# Depositional architecture and post-depositional alteration of the Toutunhe Formation (J_2_*t*) in the Louzhuangzi area, Southern Junggar Basin: Implications for uranium mineralization

**DOI:** 10.1371/journal.pone.0351337

**Published:** 2026-06-16

**Authors:** Qing Wang, Fengjun Nie, Fei Xia, Xin Zhang, Weiwei Jia, Kegai Lu, Xiao Sun, Dong-guang Yang

**Affiliations:** 1 School of Earth and Planetary Sciences, East China University of Technology, Nanchang, Jiangxi, China; 2 National Key Laboratory of Uranium Resources Exploration-Mining and Nuclear Remote Sensing, East China University of Technology, Nanchang, Jiangxi, China; 3 No. 216 Geological Party, China National Nuclear Corporation, Urumqi, Xinjiang, China; Ural Federal University named after the first President of Russia B N Yeltsin: Ural'skij federal'nyj universitet imeni pervogo Prezidenta Rossii B N El'cina, RUSSIAN FEDERATION

## Abstract

In recent years, economically significant sandstone-hosted uranium mineralization has been identified in the Louzhuangzi area along the southern margin of the Junggar Basin. However, the controls on uranium enrichment and their links to depositional architecture and post-depositional fluid processes remain insufficiently constrained. This study integrates field geological investigations, drill-core lithofacies logging, and systematic sampling with petrographic and micro-analytical techniques, including optical microscopy, scanning electron microscopy (SEM), electron probe microanalysis (EPMA), and transmission electron microscopy (TEM). The objective is to elucidate the depositional characteristics, alteration processes, and uranium occurrence mechanisms of the ore-bearing sandstones within the Toutunhe Formation (J_2_*t*). Results show that the upper member (J_2_*t*^2^) represents meandering-river deposits, whereas economically significant uranium mineralization is hosted in the lower member (J_2_*t*^1^), characterized by braided-river sandstone units with high permeability. The sandstones of the Toutunhe Formation (J_2_*t*) exhibit intense oxidation by surficial fluids, overprinted by post-mineralization hydrothermal alteration and sulfide-forming alteration associated with reducing fluids. Uranium is closely associated with pyrite, organic matter, and clay minerals. Uranium minerals are dominated by coffinite and pitchblende (~68%), with UO₂ contents of 52.89–86.07%. Minor Ti-bearing uranium phases (~16%), interpreted as possible brannerite, contain 38.01–41.46% UO₂ and 31.67–36.09% TiO₂, while nanoscale uranium minerals (~16%) show UO₂ contents of 9.15–60.28%. These results indicate that uranium mineralization was controlled by the coupling of braided-channel architecture and multi-stage fluid processes. Uranium was initially precipitated from oxidized fluids and subsequently modified and preserved by later thermal and reducing fluids, highlighting the importance of multi-fluid interactions in sandstone-hosted uranium systems.

## 1. Introduction

With the wide application of the in-situ leaching uranium mining technology, sandstone-type uranium deposit has become the main target of uranium resource exploration in China [1]. Sandstone-type uranium deposits in northern China are mainly distributed in Mesozoic-Cenozoic foreland basins, such as Ili, Turpan-Hami, and Songliao basins, as well as the Ordos Basin [2–5]. Their formation is generally controlled by a combination of uranium source, depositional architecture of host sandstones, redox conditions, diagenetic evolution, and regional tectonic framework [6–8].

Among these factors, depositional controls play a fundamental role in governing uranium mineralization. The geometry, connectivity, and permeability of fluvial and deltaic sandstone units determine the migration pathways of ore-forming fluids and the spatial distribution of uranium accumulation [1,6]. In particular, braided river and delta plain systems commonly provide favorable reservoir conditions due to their laterally continuous and highly permeable channel sandstones [9]. Furthermore, the distribution of reductants, such as organic matter and pyrite, which are strongly facies-dependent, exerts a first-order control on uranium precipitation at redox interfaces [7,10,11].

The Junggar Basin is one of the important basins in Xinjiang region, northern China where sandstone-type uranium deposits have been conducted [12]. Some promising uranium mineralizations have been discovered in Jurassic, Cretaceous, and Paleogene sandstones in the Dingshan area of the northern basin, the Kamusite area of the eastern basin, and along the southern margin of the basin, attracting considerable attention from the uranium exploration industry [13]. However, the uranium mineralization with economic values has been discovered only in the Jurassic Toutunhe Formation (J_2_*t*) of the Louzhuangzi area, southern margin of the basin at present, and a medium-sized uranium mineral area has been confirmed [14]. Previous studies suggest that the southern margin of the basin experienced prolonged interaction with uranium-bearing oxidizing fluids under relatively stable tectonic conditions during the Late Cretaceous to Paleogene [15–17]. The Middle Jurassic Toutunhe Formation (J_2_*t*), characterized by thick braided river delta plain sandstones, constitutes the principal host of economic uranium mineralization in the Louzhuangzi area [18, 19]. Uranium mineralization is spatially associated with interlayer oxidation zones and occurs preferentially within permeable sandstones enriched in organic matter and pyrite [20]. In addition, increasing evidence indicates that hydrocarbon-related reduction and deep-seated fluid activity may have played a significant role in uranium enrichment and preservation, particularly in the development of grayish-white alteration zones [14,20,21].

Currently, two competing genetic hypotheses currently frame the metallogenic theory of sandstone-type uranium deposits, resulting in pronounced academic divergence. The traditional interlayer oxidation-zone model emphasizes the lateral migration of surficial, oxygen-bearing, uranium-rich fluids through permeable sandstones, with uranium precipitation occurring at redox interfaces [1,6,10]. In contrast, the deep-fluid exhalation or superimposed mineralization model proposes that deep-seated fluids—such as hydrocarbons and thermal fluids—migrate upward, supplying reducing agents that facilitate uranium precipitation or exert superimposed modification on pre-existing mineralization [3,22,23]. This interpretation has gained widespread acceptance in the Ordos Basin [24–26], the Songliao Basin [27, 28], and the Erlian Basin [29–32].

In the Louzhuangzi uranium deposit, previous studies have documented favorable geological conditions for mineralization, including well-developed braided river sandstone units and interlayer oxidation zones [18]. Nevertheless, interpretations remain inconsistent. Some researchers attribute uranium enrichment primarily to depositional facies variations and oxidation front migration [18, 19], whereas others emphasize the critical role of hydrocarbon-related reduction and deep fluid activity [14,20,21]. This divergence reflects a broader uncertainty regarding the relative importance of depositional versus fluid-driven controls in uranium mineralization. Notably, previous research has primarily emphasized macroscopic geological characteristics, while micro- to nanoscale petrographic and mineralogical investigations of ore-bearing sandstone units remain insufficiently constrained, limiting a comprehensive understanding of uranium mineralization processes.

To address these issues, the objectives of this study are explicitly defined as follows: (i) to characterize the depositional architecture and structural features of the host sandstone units; (ii) to identify mineralogical and textural characteristics using multiscale analytical techniques, including scanning electron microscopy (SEM), electron probe microanalysis (EPMA), and focused ion beam–transmission electron microscopy (FIB–TEM); (iii) to constrain the evolution of metallogenic fluids; and (iv) to establish a coupled genetic model linking depositional processes with fluid activity.

Through this integrated framework, this study aims to provide new insights into the sedimentation–fluid coupling mechanism of uranium mineralization in the Toutunhe Formation along the southern margin of the Junggar Basin, and to offer an improved conceptual model for sandstone-hosted uranium systems in comparable sedimentary basins.

## 2. Geological background

**Fig 1:**
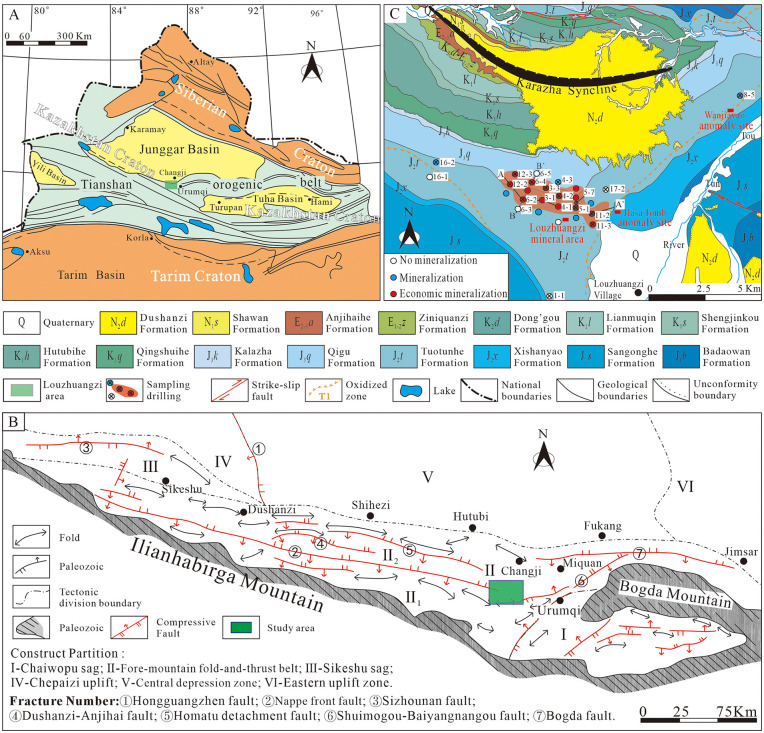
Geological map of the study area. **(A)** Tectonic location sketch of the Junggar Basin (modified from [21,44]); **(B)** Division and distribution of major tectonic units on the southern margin of the Junggar Basin (modified from [15,21]); **(C)** Geological map of Louzhuangzi area (modified from [20]). (A) and **(B)** Reprinted from [Wang, **Q.**, Nie, **F.**J, Yang, **Y.**P, Xian, **H.**Y, Xia, **F.**, Yan, **Z.**B, et al. Brannerite identification in the Louzhuangzi sedimentary U deposit and implications for deposit genesis or post-ore alteration. 2025; 106908] under a CC BY license, with permission from [Ore Geology Reviews], original copyright [2025]. **(C)** Reprinted from [Jia **W.**W, Wang **G.**R, Tang **X.**F, Huang S, Yan **J.**J. Discussion on the relationship of different types of alteration zones to the uranium mineralization in Toutunhe Formation of Louzhuangzi area, Southern Margin of Junggar Basin. 2023; 40(2):152–161.] under a CC BY license, with permission from [World Nuclear Geoscience], original copyright [2023].

The Junggar Basin is located in the eastern segment of the Kazakhstan Plate and is bounded by the Siberian Plate to the north and the Tarim Plate to the south (Fig 1A). It represents a typical intracontinental composite foreland basin formed through multiple phases of tectonic superposition since the Paleozoic [33–37]. Since the Late Paleozoic, the region has undergone a series of tectonic events, including ocean basin closure, terrane accretion, and intracontinental reworking. During the early Mesozoic, pre-existing Paleozoic tectonic frameworks were reactivated. Subsequently, the Cenozoic collision between the Indian and Eurasian plates triggered significant uplift of the Tianshan Mountains and intense N–S compressional deformation, leading to the development of the present-day foreland fold–thrust belt and associated piedmont depression system along the northern Tianshan [38–43].

The architecture of sandstone bodies, the development of oxidizing fluid pathways, and depositional heterogeneity along the southern margin of the Junggar Basin are the combined result of tectonic uplift, climatic aridification, and basin infilling processes [43]. During the Jurassic, a continental depositional system dominated by alluvial plains, lacustrine environments, and deltaic settings developed between the southern Junggar Basin and the northern Tianshan orogenic belt. In the Early to Middle Jurassic, relatively humid climatic conditions prevailed, promoting the widespread formation of coal-bearing strata and highly permeable braided fluvial sandstone units [35,45]. These provided both migration pathways and reducing agents for subsequent sandstone-hosted uranium mineralization. From the Late Middle Jurassic to Late Jurassic, localized uplift of the Tianshan Mountains and enhanced sediment recycling occurred, accompanied by a regional climatic transition from humid to semi-arid and arid conditions. During deposition of the Qigu Formation, coal seams decreased, whereas red beds and paleosols became more abundant, indicating increasingly oxidizing conditions within the basin [35,43,46].

During the Late Jurassic to Early Cretaceous, the Kalaza Formation and the Tugulu Group were widely deposited in the Junggar, Tarim, and other peri-Tianshan basins, characterized by alluvial fans, seasonal flood deposits, and aeolian dune systems [35,40].

Since the Cenozoic, the collision between the Indian and Eurasian plates has led to strong intracontinental reactivation and crustal shortening within the Tianshan orogenic belt. As a result, a series of thrust faults, fold–thrust belts, and piedmont depression systems developed along the southern margin of the Junggar Basin [35,40,47,48]. Under the combined influence of plate collision, intracontinental adjustment, and far-field tectonic effects, the present-day structural–geomorphological framework of “three mountain belts enclosing two basins” was established, separating the Junggar Basin from the uranium-bearing Turpan–Hami Basin and Yili Basin (Fig 1A) [47,49].

Meso–Cenozoic tectonic uplift not only modified the hydrogeological conditions of the basin but also facilitated the recharge of oxygenated meteoric waters along piedmont outcrop zones and high-permeability sandstone units into the basin interior. This process provided a critical driving mechanism for the migration of redox fronts, uranium mobilization, transport, and precipitation in sandstone-hosted uranium systems [15,16,49–51].

**Fig 2:**
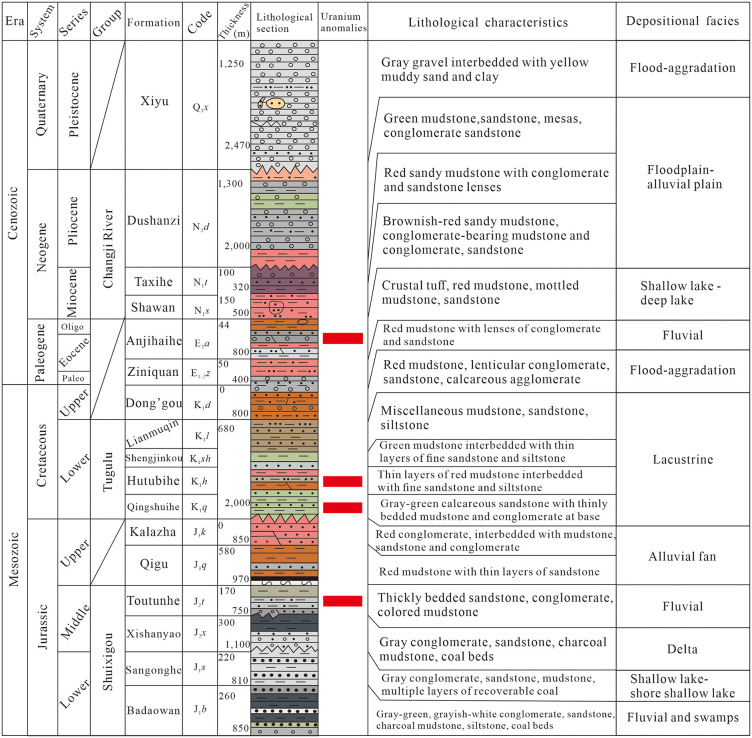
Stratigraphic column of the Louzhuangzi area (modified from internal report, CNNC Geological Party No. 216, 2022).

The Meso–Cenozoic strata are well exposed along the southern margin of the Junggar Basin, among which the Jurassic, Cretaceous, and Paleogene successions constitute the principal target horizons for sandstone-hosted uranium exploration (Fig 2). The Louzhuangzi area is located within a secondary tectonic unit along the southern margin of the basin (Fig 1B) and belongs to the frontal zone of the northern Tianshan foreland fold–thrust belt [15]. In recent years, the Louzhuangzi uranium district has been discovered within the foreland fold–thrust belt at the junction between the eastern Yilianhabirga Mountains and the western segment of the Bogda Mountains tectonic belt (Haojiagou uplift) (Fig 1B) [14,21]. Together with previously identified uranium occurrences and anomalies such as Hasa Tomb area and Wanjayao area, this newly discovered district defines a promising sandstone-hosted uranium mineralization belt with significant exploration potential [13,20] (Fig 1C).

## 3. Materials and methods

### 3.1. Fieldwork and sampling

Field investigations were conducted in the Louzhuangzi uranium deposit on the southern margin of the Junggar Basin. An integrated approach, including stratigraphic logging, outcrop observation, and systematic core description, was employed to characterize depositional facies, alteration features, and mineralization-related lithological variations. Sampling was restricted to the Middle Jurassic Toutunhe Formation (J_2_*t*), with particular emphasis on grayish-white altered sandstones hosting economically significant uranium mineralization, thereby ensuring direct relevance to the research objectives. Core materials were obtained from exploration drill holes provided by the CNNC No. 216 Geological Party, and only fresh, unweathered, and lithologically intact intervals were selected for analysis.To ensure spatial representativeness, a systematic sampling strategy integrating both vertical and lateral controls was adopted. Laterally, sampling covered the western, central, and eastern sectors of the alteration zone across the deposit (Fig 1C). Vertically, samples were collected from depths ranging from 24.84 m to 871.50 m, encompassing the entire grayish-white alteration interval within the Toutunhe Formation (J_2_*t*). This design enables characterization of depth-dependent mineralogical variations as well as lateral heterogeneity. A total of 52 drill core samples (6–8 cm in diameter and ~10–15 cm in length) were collected, of which 46 were impregnated with resin and prepared as polished thin sections for petrographic analysis. The 20 sandstone samples listed in S2 Table represent the analytical subset used to illustrate the micro-petrographic characteristics discussed in this study.

In addition, representative drill holes (ZK12−2, ZK6−2, ZK3−1, ZK4−1, ZK5−1, ZK5−7, and ZK11−2) were selected for detailed intra-formational and profile-scale sedimentological analysis (Figs 7–9). The depositional facies were preliminarily identified in terms of integrative lithological subdivision, depositional cycle features, color variations and sedimentary structures of sandstones in the Toutunhe Formation (J_2_*t*).

### 3.2. Mineralogical analysis methods

#### Optical petrography.

A total of 14 polished thin sections were prepared from representative samples (S2 Table) and examined using a Zeiss AxioScope.A1 transmitted–reflected digital polarizing microscope. Petrographic analysis focused on mineral assemblages, textural relationships, and diagenetic features, following standard petrographic classification schemes. In addition, the detrital components of sandstones (n = 68) from the Toutunhe Formation (J_2_*t*) in the Louzhuangzi area were identified using a polarizing microscope (Zeiss AxioScope A1). Modal composition was determined by point-counting, whereby the areal proportions of quartz, feldspar, and lithic fragments were statistically quantified within the field of view. The relative percentages of each clastic component were subsequently calculated. The resulting compositional data (S1 Table) were projected onto the Folk’s ternary diagram for sandstone classification.

#### Scanning electron microscopy (SEM).

Eighteen sandstone samples were prepared as polished thin sections (n = 12) and solid block specimens (n = 6), followed by carbon coating prior to analysis. All samples were examined using a FEI Nova Nano SEM 450 equipped with an energy-dispersive X-ray spectroscopy (EDS) system. SEM observations were conducted under high-vacuum conditions at an accelerating voltage of approximately 15–20 kV. This analysis was used to (i) identify uranium-bearing mineral phases, (ii) characterize microtextures and alteration features, and (iii) resolve mineral assemblages at the micrometer scale.

#### Electron microprobe analysis (EPMA).

Quantitative compositional data (S3 Table) of uranium minerals and titanium-bearing phases in uranium ore samples (n = 4) were obtained using a JEOL JXA-8100 electron probe microanalyzer (EPMA) equipped with an Inca Energy EDS. Analytical conditions included 15 kV accelerating voltage and 10 nA beam current, and spot diameter ≤ 2 μm. The counting time was 5 s per element. X-ray lines analyzed included: SiKα, AlKα, MgKα, KKα, NaKα, YLα, CaKα, PKα, FeKα, TiKα, ZrKα, PbMα, MnKα, UMα, ThMα, and CeLα. The following mineral standards were used for calibration: Jadeite (Si, Al, Na), Sanidine (K), Olivine (Mg, Fe), Plagioclase (Ca), Rutile (Ti), Zircon (Zr), Cubic zirconia (Y), Monazite (P, Ce), Uraninite (Pb, U, Th), Rhodonite (Mn).

#### FIB-TEM sample preparation and transmission electron microscopy.

At the Guangzhou Institute of Geochemistry, a focused ion beam (FIB) technique was employed to prepare an electron-transparent foil (~100 nm thick) from Ti–U mineral phases identified in a polished thin section of uranium ore sample 22ZGE001 from drill hole ZK12−2. The foil was subsequently analyzed using a Thermo Scientific Talos F200S transmission electron microscope (200 kV) equipped with an energy-dispersive X-ray spectroscopy (EDS) system. This analysis enabled nanoscale characterization of brannerite-like Ti–U mineral assemblages.

#### Analytical facilities.

Except for FIB sample preparation and TEM-EDS analysis, all other experiments were conducted at the State Key Laboratory of Nuclear Resources and Environment, East China University of Technology.

## 4. Results

### 4.1. Petrographic features of the Toutunhe Formation sandstones

**Fig 3:**
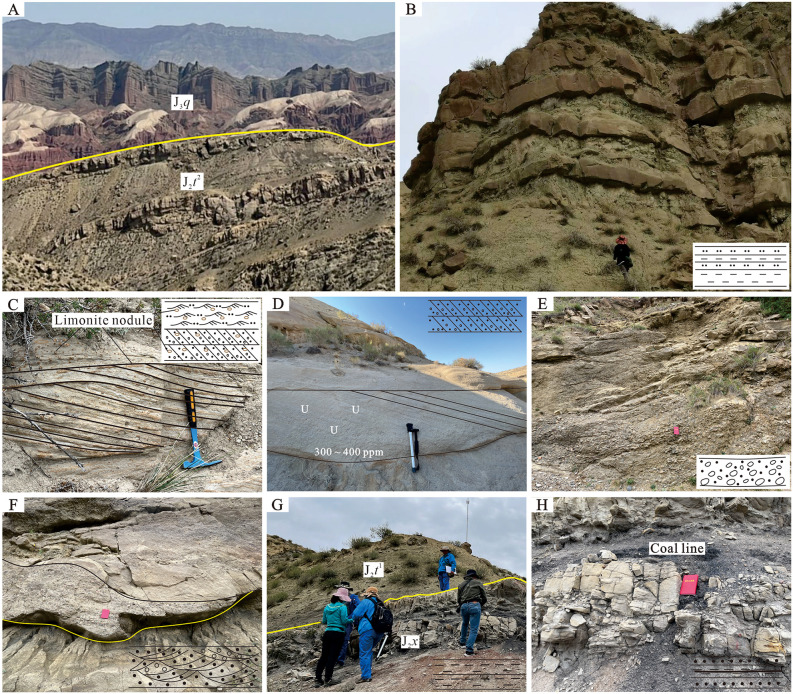
Petrographic photographs of outcrops of the Toutunhe Formation (J₂t) in the Louzhuangzi area. **(A–C)** Outcrops of the Upper Member of the Toutunhe Formation (J_2_*t*^2^): fine–medium grained sandstone with parallel bedding **(A)**, massive mudstone **(B)**, fine–medium grained sandstone with horizontal bedding **(B)**, and fine sandstone with flaser bedding and planar cross-bedding **(C)**. **(D–F)** Outcrops of the Lower Member of the Toutunhe Formation (J_2_*t*^1^): sandstone with large to giant planar cross-bedding **(D)**, massive conglomerate **(E)**, and glutenite and coarse sandstone with trough cross-bedding **(F)**. **(G)** Stratigraphic contact between the Lower Member of the Toutunhe Formation (J_2_*t*^1^) and the Xishanyao Formation (J_2_*x*). **(H)** Outcrop of the Xishanyao Formation (J_2_*x*): siltstone with parallel bedding and coal seams.

In the Toutunhe Formation (J_2_*t*), mudstone is commonly interbedded with sandstone and displays a wide range of colors. In addition to the dominant grayish-green mudstone, brownish-red to red varicolored mudstones are also present (Fig 7A). The mudstones are predominantly characterized by massive bedding (Figs 3A, B). Sandstones encountered in drill cores are typically grayish white to gray (Fig 7B), whereas yellowish and locally dark purple sandstones occur in outcrop (Fig 3B). In terms of grain size, medium- to coarse-grained sandstones are dominant, followed by medium-, fine-grained sandstones and siltstones. Sedimentary structures commonly developed in the sandstones include flaser bedding (Fig 3C), parallel bedding (Fig 3A), horizontal bedding (Fig 3B), and small- to medium-scale planar cross-bedding (single sets <10 cm; Figs 3C, 12B).

Gravel-bearing sandstones, glutenites, and conglomerates identified in drill cores are generally grayish white (Fig 7B) and contain abundant carbonaceous debris and pyrite (Figs 12J–L), with locally developed clay alteration (Fig 12K). In outcrop, these coarse-grained deposits commonly exhibit large- to giant-scale planar cross-bedding (single sets >10 cm; Fig 3D), massive bedding (Fig 3E), trough cross-bedding (Fig 3F), and scour surfaces.

**Fig 4:**
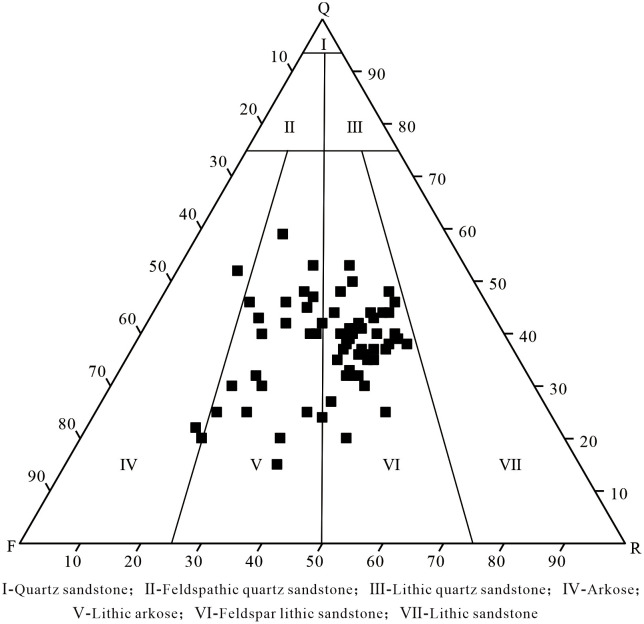
Sandstone classification diagram of the Toutunhe Formation (J_2_*t*) in the Louzhuangzi area (According to [52]).

Studies on the components of the Toutunhe Formation (J_2_*t*) sandstone clastics show that the percentage contents of the debris and feldspar in the sandstone are relatively high. Specifically, quartz accounts for 18% to 59%, averaging 38%; feldspar accounts for 15% to 50%, averaging 26%; debris accounts for 10% to 60%, averaging 35%. The compositional maturity index Q/(F + R) of the sandstone ranges from 0.22 to 1.44, averaging 0.65 (S1 Table), which is relatively low. Projection of the modal data onto the Folk’s ternary diagram indicates that the sandstones of the Lower Member of Toutunhe Formation (J_2_*t*^1^) are predominantly classified as feldspar lithic sandstone, followed by lithic feldspar sandstone, and the feldspar sandstone is only rarely observed (Fig 4).

**Fig 5:**
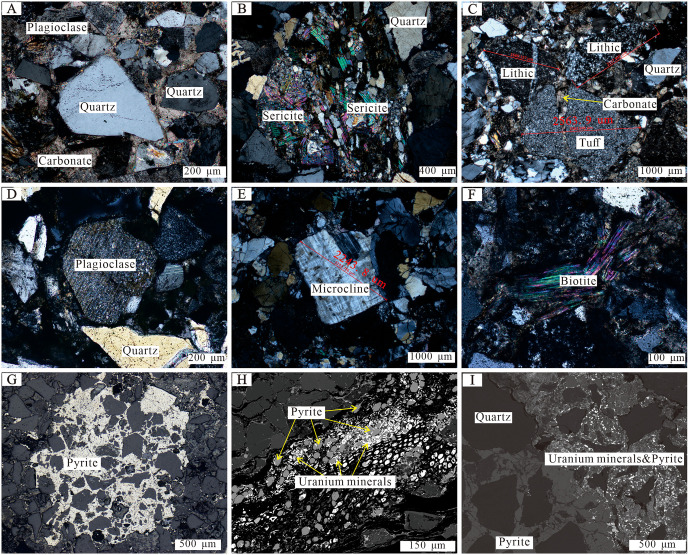
Microscopic petrographic characteristics of sandstone samples from the Toutunhe Formation (J_2_*t*) in the Louzhuangzi area. **(A)** Sharp-edged quartz clasts cemented by basal carbonate cement, ZK5−1, 22ZGE033, 148.50 m, transmitted light (+); **(B)** Clastic grains and clay matrix, ZK6−2, 22ZGE016, 477.30 m, transmitted light (+); **(C)** Pink crystalline quartz and feldspar filling between detrital grains, ZK6−2, 22ZGE016, 477.30 m, transmitted light (+); **(D)** Illitized plagioclase with cloudy surface, ZK6−2, 22ZGE063, 478.60 m, transmitted light (+); **(E)** Microcline with lattice twinning, ZK6−2, 22ZGE014, 483.47 m, transmitted light (+); **(F)** Bent and crushed sericitized biotite, ZK12−2, 22ZGE001, 672.48 m, transmitted light (+); **(G)** Pyrite cementation, ZK4−2, 23ZGE026, 398.20 m, reflected light; **(H)** Fine-grained pyrite and uranium minerals filling cellular cavities of carbonaceous debris, ZK4−2, 23ZGE026, 398.20 m, BSE image; **(I)** Pyrite and uranium minerals interstitially filling detrital pores, ZK12−3, 23ZGE028, 798.50 m, BSE image.

**Fig 6:**
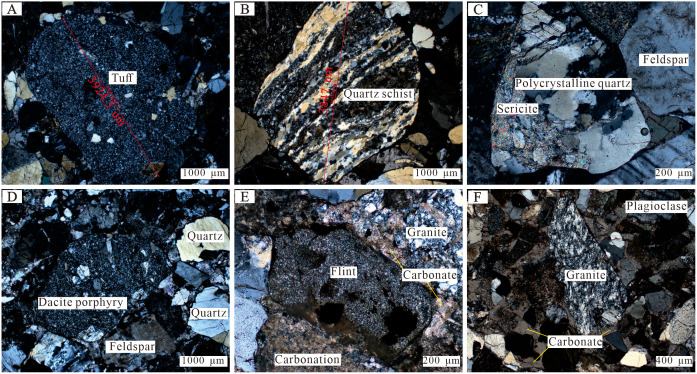
Microscopic detrital characteristics of sandstone samples from the Toutunhe Formation (J_2_*t*) in the Louzhuangzi area. **(A)** Cryptocrystalline tuff detritus, ZK6−2, 22ZGE016, 477.30 m, transmitted light (+); **(B)** Quartz schist detritus with platy structure, ZK6−2, 22ZGE015, 479.30 m, transmitted light (+); **(C)** Sericitization of sharp-edged polycrystalline quartz, ZK6−2, 22ZGE016, 477.30 m, transmitted light (+); **(D)** Dacite porphyry detritus with porphyritic texture, ZK6−2, 22ZGE026, 398.80 m, transmitted light (+); **(E)** Dissolution pits developed in cryptocrystalline flint detritus, ZK6−2, 22ZGE026, 398.80 m, transmitted light (+); **(F)** Granite detritus cemented by carbonate minerals, ZK11−2, 22ZGE034, 24.84 m, transmitted light (+).

The content of sandstone clastics ranges from 70% to 89%, averaging 82%, with quartz, feldspar and debris as the main components. The sorting property of the clastic particles is mostly poor, and the roundness is mainly sub-angular to angular, with only a small amount of debris being sub-round to round (Figs 5 and 6). The clastic particles are relatively dispersed and generally do not contact with each other (Figs 5A-F), with the particle diameters being up to 2600 μm, mostly between 500 and 1500 μm, averaging about 800 μm. The structural type is mainly coarse-giant grained sandy structure, followed by fine-medium grained sandy structure. The interstitial materials between the clastic particles are mainly composed of matrix and cements, with a content of approximately 20% to 30%. The matrix content is relatively high, with the components being mainly fine silty quartz and feldspar particles (Figs 5B, C, E), followed by a small amount of clay mineral, such as illite, sericite (Fig. 5B), and kaolinite, smectite, chlorite (Figs 10E, F and 15, 16). In the uranium mineralized sandstone samples, finely dispersed uranium minerals and fine-grained pyrite are commonly observed in the matrix (Figs 5H, I and 10A-C), and associated with clay minerals (Figs 10E, F, 16A-C) and carbonaceous debris (Fig 5H). The cementing materials are mainly composed of carbonate and clay minerals (Figs 5A and 6F), followed by gelatinous pyrite (Figs 5G and I). The main cementing types are mostly basal cementation, with matrix-supported structures (Figs 5A-F). These clastic particles features indicate that both the compositional maturity and the structural maturity of the sandstone are generally low.

The relative content of quartz ranges from 22% to 59%, averaging 40%, with single crystal quartz being the main component, accounting for about 65% of the total amount of quartz, followed by polycrystalline quartz, accounting for about 35%. The quartz is generally characterized by dirty and rough surface, with poor roundness, and commonly shows angular and sub-angular shapes (Figs 5A-D and 6C, D). The feldspar content ranges from 11% to 36%, averaging 21%, with microcline and plagioclase being the main components. The microcline is mainly the characterized by lattice twin crystal structures, with the twin crystal bands usually having a spindle-shaped lattice pattern under the microscope. (Fig 5E). The plagioclase mainly shows the characteristics of polysynthetic twin texture, with serimicization usually occurring on the surface (Fig 5D), and compaction and crushing of altered biotite occasionally being observed (Fig 5F).

The debris particles have relatively large diameters and good roundness being sub-round and round, with cement and detrital matrix usually observed on the edge. The debris can be classified into the following 3 types according to the rock property in the source area: igneous rock debris, sedimentary rock debris and metamorphic rock debris. The commonly observed tuff debris (Fig 6A), dacite porphyry debris (Fig 6D), and granite debris (Figs 6E, F) are all the products of fragmentation and deposition of the intermediate acidic igneous rocks and intrusive rocks. The sedimentary rock debris is composed mainly by siliceous rock debris (Fig 6C), while flint debris (Fig 6E) and quartz schist debris (Fig 6B) constitute the main types of the metamorphic rock debris. The rhyolite spherulitic texture, almond shaped body, is clearly visible in the igneous rock debris (Fig 6A), indicating that the weathering of the pro-rock is relatively intense. Porphyritic texture is identified in part of the acidic volcanic rock debris (Fig 6D), with felsic mineral as the main composition of the phenocryst and cryptocrystalline quartz as the matrix. In some thin slices of rock samples, the epigranite debris can be found, the granitic texture composed of quartz and feldspar is clearly visible, whereas most of the mica-type minerals are absent (Figs 6E, F). The metamorphic rock is mainly composed of the quartzite debris that accounts for 90% of the metamorphic rock debris, mainly in the forms of polycrystalline quartz debris (Fig 6C) and flint debris (Fig 6E), followed by the quartz schist debris (Fig 6B), with obvious platy structures.

### 4.2. Sandstone depositional characteristics

**Fig 7:**
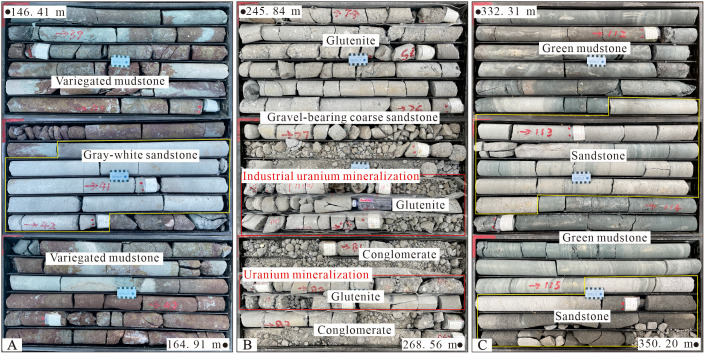
Representative drill (ZK5-7) core photographs from the Toutunhe Formation (J_2_*t*) in the Louzhuangzi area. **(A)** Meandering river facies: thick mudstone interbedded with thin sandstone of the Upper Member of Toutunhe Formation (J_2_*t*^2^); **(B)** Gravelly braided river facies: thick sand body of the Lower Member of Toutunhe Formation (J_2_*t*^1^); **(C)** Sandy braided river facies: floodplain mudstone and crevasse splay sandstone at the base of the Lower Member of Toutunhe Formation (J_2_*t*^1^).

**Fig 8:**
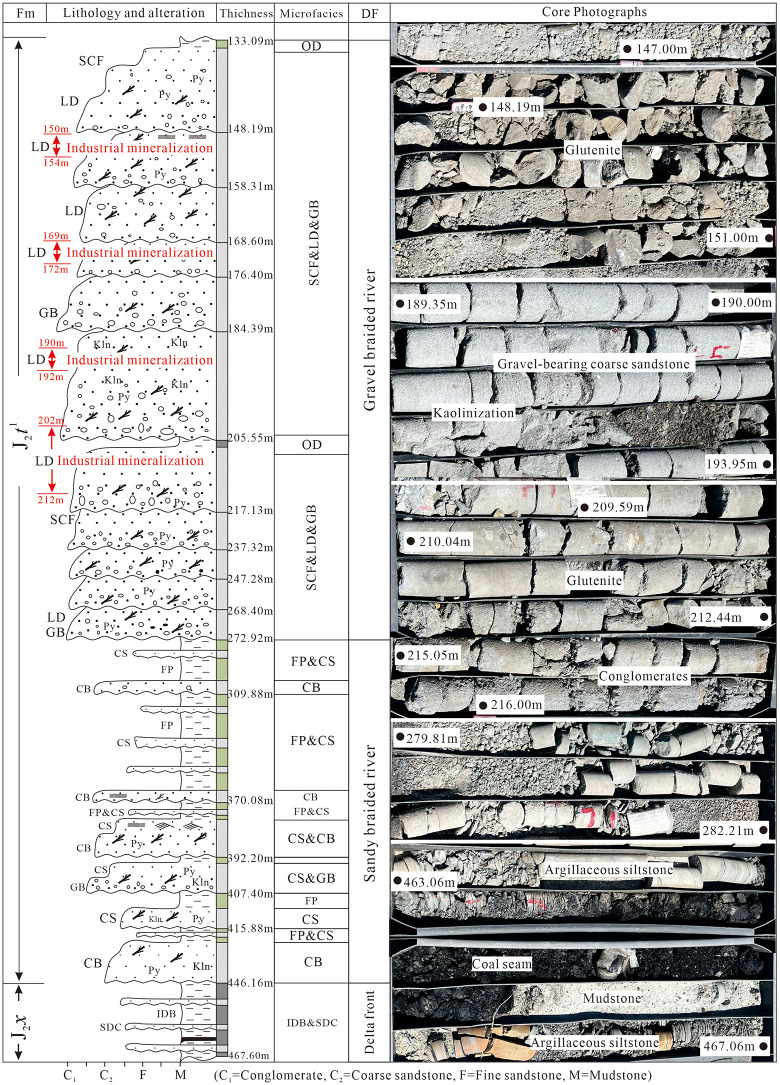
Interpretative stratigraphic column of drill hole ZK5−1 in the Louzhuangzi area. (Abbreviation: Fm = Formation; DF = Depositional Facies; OD = Overbank Deposit; SCF = Sandy Channel Fill; LD = Lag Deposit; GB = Gravel Bar; FP = Floodplain; CS = Crevasse Splay; CB = Channel Bar; IDB = Interdistributary Bay; SDC = Subaqueous Distributary Channel).

Based on the vertical depositional cycle characteristics of the Toutunhe Formation (J_2_*t*) sandstone units, it can be divided into upper and lower sections [18–20]. The Upper Member (J_2_*t*^2^) consists of relatively thick purple-red to dark purple mudstone interbedded with thin grayish-white siltstone and fine sandstone layers (Fig 7A). Depositional microfacies including floodplain, natural levee, point bar, and channel-fill deposits are recognized (Fig 8). Sedimentary structures observed in the Upper Member (J_2_*t*^2^) include massive bedding in mudstone (Fig 3B), flaser bedding in fine sandstone (Fig 3C), horizontal bedding in mudstone and sandstone (Fig 3B), parallel bedding in sandstone (Fig 3A), and small- to medium-scale planar cross-bedding (Figs 3C and 12B).

The ore-bearing sandstone units in the Lower Member of the Toutunhe Formation (J_2_*t*^1^) are composed predominantly of thick grayish-white coarse sandstone, glutenite, and conglomerate, interbedded with thin layers of grayish-green mudstone (Fig 7B). Depositional microfacies, including sandy channel-fill deposits, lag deposits, and gravel bars, are well developed (Fig 8). Glutenite exhibiting trough cross-bedding (Fig 3F) and conglomerate with massive bedding (Fig 3E) are also commonly observed.

At the base of the Lower Member (J_2_*t*^1^), gray to grayish-white coarse sandstone and medium- to fine-grained sandstone are interbedded with green mudstone (Fig 7C). Depositional microfacies such as floodplain deposits, crevasse splays, and channel bars are identified (Fig 8). Sedimentary structures including massive bedding, small-scale planar cross-bedding, climbing ripple lamination, and scour surfaces are well developed, indicating variable hydrodynamic conditions during deposition (Fig 8).

Up-section from the Lower to the Upper Member, the proportion of thick conglomerate and coarse sandstone decreases, whereas grayish-white siltstone and fine sandstone become increasingly abundant. In addition, multiple gypsum layers occur within the succession (Figs 12E, I).

### 4.3. Uranium mineralization characteristics

**Fig 9:**
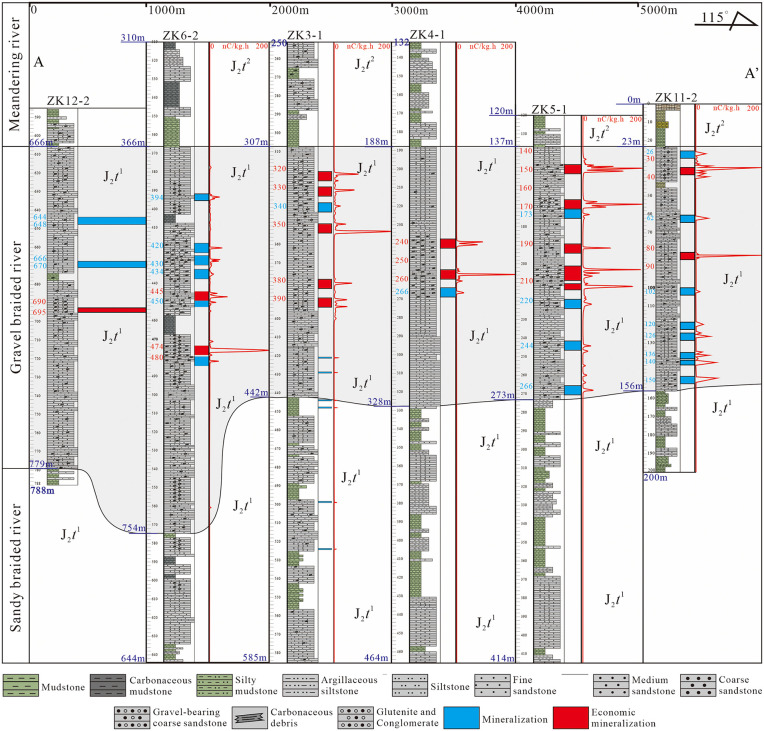
Profile of the A-A’ drill hole in Louzhuangzi area. The top boundary of the sandstone interval within the Lower Member of the Toutunhe Formation (J_2_*t*^1^) is horizontally leveled. The profile location is shown in Fig 1C.

The uranium mineralization in the Louzhuangzi area occurs in the sandstones of the Lower Member of the Toutunhe Formation (J_2_*t*^1^) (Figs 8, 9). The classification of depositional facies based on outcrops and borehole strata shows that the Lower Member of the Toutunhe Formation (J_2_*t*^1^) contains sandstones deposited in braided-river delta plain, sandy braided-river, and gravelly braided-river facies (Figs 7-9). The uranium mineralization zone, large in scale, is distributed along the direction of the sandstone trend from east to west, being about 5.5 km long from east to west and about 800 m–1,100 m in width (Fig 1C), controlled mainly by the depositional sandstones of braided river delta plain deposition (the main channel of gravelly braided river) (Fig 14). The A-A ‘borehole profile (Fig 9) exposes multiple layers of uranium bodies and uranium mineralized bodies in the depths ranging from 32.05 m to 695.55 m, with the depth being deeper in the west and shallower in the east (approaching the eroded surface). The thickness of the economic ore bodies is generally between 0.8 m and 5.6 m, controlled by a set of gravelly braided river depositional sandstones, which are thick and large, with coarse sandstone, glutenite, and conglomerate being interbedded frequently, and are loose to relatively loose in structure, with good permeability, connectivity and stratification, and rich in carbonized plant detritus. The gravelly braided river channel lag deposits is the main position for the formation of uranium mineralization (Fig 8). The thick mudstone layer deposited in the meandering river environment in the Upper Member of the Toutunhe Formation (J_2_*t*^2^) and the mud-bearing sandstone at the bottom of the Lower Member (J_2_*t*^1^) act as the top and bottom plates of the aquiclude, respectively, and form a sandstone structure favorable for uranium mineralization together with the thick gravel-bearing coarse sandstone, glutenite, and conglomerate layers in the middle and upper sections of the Lower Member (J_2_*t*^1^) (Fig 9).

### 4.4. Uranium occurrence and mineral assemblages

In the uranium ore samples from the Toutunhe Formation (J_2_*t*) of the Louzhuangzi area, uranium occurs mainly in three types of state including the independent uranium mineral, unclassified nano-uranium minerals and the isomorphism substitution mineral.

#### 4.4.1. Independent uranium mineral.

The independent uranium minerals mainly include coffinite and pitchblende, with a small amount of brannerite-like.

**Fig 10:**
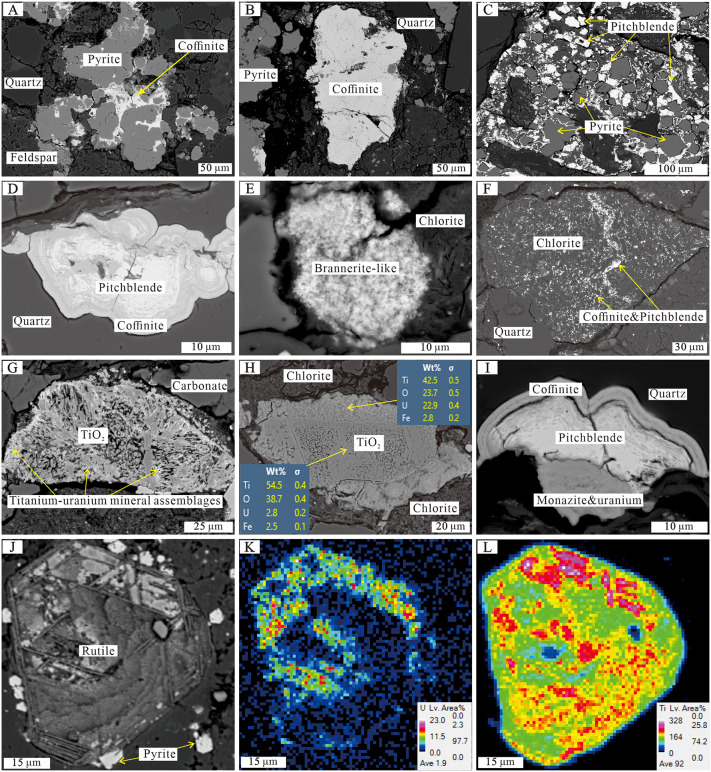
Occurrence characteristics of uranium minerals in sandstones of the Toutunhe Formation (J_2_*t*) in the Louzhuangzi area (BSE images). **(A)** Colloidal coffinite associated with pyrite, ZK11−3, 23ZGE022, 119.52 m; **(B)** Massive coffinite associated with fine-grained pyrite, ZK12−3, 23ZGE028, 798.50 m; **(C)** Massive pitchblende associated with pyrite, ZK12−3, 23ZGE028, 798.50 m; **(D)** Banded pitchblende coexisting with coffinite in a ring-shaped distribution, ZK4−2, 23ZGE026, 398.20 m; **(E)** Nanoscale titanium–uranium aggregates associated with chlorite, ZK3−3, 23ZGE027, 753.10 m; **(F)** Nanoscale uranium particles adsorbed on clay minerals, ZK12−3, 23ZGE028, 798.50 m; **(G)** Nanoscale uranium minerals adsorbed at the margins of titanium oxides, ZK4−2, 23ZGE026, 398.20 m; **(H)** Nanoscale uranium minerals adsorbed within dissolution pores of titanium oxides, ZK12−3, 23ZGE028, 798.50 m; **(I)** Uranium-rich monazite associated with banded pitchblende, ZK4−2, 23ZGE026, 398.20 m; **(J)** Uranium-rich rutile, ZK4−2, 23ZGE026, 398.20 m; **(K–L)** Spatial distribution of U (K) and Ti (L) elemental mappings in uranium-rich rutile via electron probe microanalysis.

Electron probe analyses indicate that the diameters of the coffinite particles generally range from 5 μm to 30 μm, with these particles mostly being distributed among the clastic particles in gel-like forms, and coexisting with gelatinous pyrite (Fig 10A); the finely dispersed coffinite particles associated with chlorite are commonly observed (Figs 10F and 16), and the coffinite particles are also commonly observed occurring in ring-shaped distribution on the quartz surface and coexist with pitchblende (Figs 10D, I); massive coffinite particles (with particle diameter up to 200 μm) can occasionally be observed in the matrix (Fig 10B). The coffinite is generally rich in Y_2_O_3_, CaO, P_2_O_5_ and FeO, with the total amount of these components accounting for 87.51% to 97.14%, averaging 91.81%. Specifically, the contents of SiO_2_, UO_2_, Y_2_O_3_, CaO, P_2_O_5_, FeO range from 9.33% to 18.23%, 52.89% to 66.88%, 0.50% to 7.33%, 0.62% to 7.21%, 1.06% to 6.44%, and 0.09% to 4.59%, respectively, with the average contents of 14.31%, 61.33%., 3.40%., 3.42%, 3.43%, and 1.55%, respectively. Additionally, the coffinite also contains a small amount of impurities such as TiO_2_, ZrO_2_, PbO, and MnO (S3 Table).

Pitchblende particles are relatively fine, generally ranging from 5 μm to 20 μm in diameters, and its content in the independent uranium minerals is lower than that of coffinite. The composition and appearance characteristics of pitchblende can basically be determined by electron microscope. Under the electron microscope, the massive pitchblende particles can be observed to appear in the matrix among the clastic particles, often associated with clay minerals and fine-grained pyrite (Fig 10C), and the baited pitchblende can also be commonly observed to be wrapped by the in ring-shaped distribution of coffinite in the quartz dissolution pits (Figs 10D, I). The pitchblende is characterized by a lower SiO_2_ content and a higher UO_2_ content. The total amount of the pitchblende chemical components accounts for 86.29% to 95.38%, with an average of 90.63%. In which, SiO_2_ content ranges from 1.05% to 5.76%, averaging 2.36%. and UO_2_ content ranges from 75.50% to 86.07%, averaging 81.06%. Additionally, the pitchblende also contains a small amount of impurities such as Y_2_O_3_, CaO, P_2_O_5_, and FeO (S3 Table).

**Fig 11:**
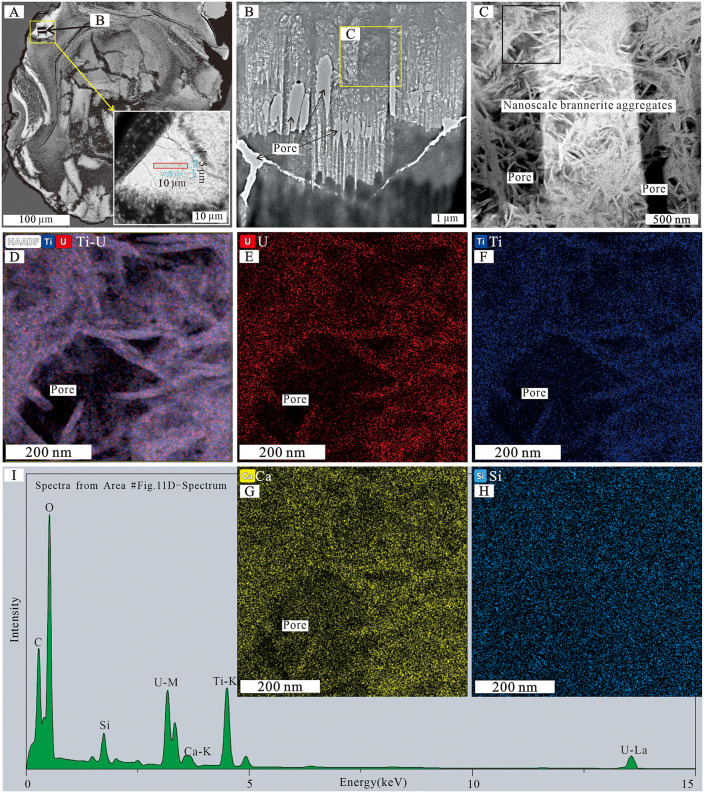
FIB and TEM images of titanium‑bearing uranium minerals (ZK12−2, 22ZGE001, 672.48 m). **(A)** Backscattered electron (BSE) image of titanium-bearing uranium mineral obtained via FIB-SEM; **(B)** TEM image of the ultrathin foil of titanium-bearing uranium mineral. The ultrathin foil was extracted vertically downward from the yellow rectangular area in panel A, with a thickness < 100 nm; **(C)** HAADF-STEM image of titanium-bearing uranium mineral; (D–I) STEM-EDS elemental distribution maps of Ti–U **(D)**, U **(E)**, Ti **(F)**, Ca **(G)**, Si **(H)**, and corresponding energy spectrum (I) for the black rectangular area in panel **C.**

Besides coffinite and pitchblende, titanium-containing uranium minerals are also commonly found in the independent uranium minerals. This type of uranium mineral usually fills among the clastic particles in the form of disseminated mineral aggregates (Fig 11A), or associated with chlorite (Fig 10E). Under the transmission electron microscope, it can be observed that the titanium-containing uranium mineral particles present a bright-white, complexly interwoven acicular aggregate morphology (Figs 11B, C). The micro-zone element area distribution image shows that this type of uranium-containing mineral is rich in Ti and U, with the distribution of the two elements being relatively uniform (Figs 11D-F). When combined with the micro-zone area-scanning element energy spectrum image (Fig 11I), it can be confirmed that this type of uranium mineral is a collection of nano-scale brannerite-like aggregate, rather than nanoscale coffinite or pitchblende associated with titanium oxides. The difference between the contents of TiO_2_ and UO_2_ in the brannerite-like is small and tends to be stable, with the average contents of TiO_2_ and UO_2_ being 33.20% and 39.62%, respectively. The contents of SiO_2_, CaO and ZrO_2_ in the brannerite-like are relatively high, with the average contents of SiO_2_, CaO, and ZrO_2_ being 6.48%, 1.74%, 2.88%, respectively. Additionally, a small amount of impurities such as Al_2_O_3_ and FeO also exist in the brannerite-like (S3 Table).

Some analyses are of relatively poor quality, with total oxide contents below 85 wt%. This suggests that some mineral phases may contain volatile components or undetected constituents (e.g., structural water or beam‑sensitive phases), and that amorphous domains may also be present, which complicate quantitative analysis—for example, the auto‑oxidation of U⁴⁺ to U⁶⁺ [53].

#### 4.4.2. Unclassified nano-uranium minerals.

Under scanning electron microscope, the nano-scale uranium minerals adsorbed in chlorite are commonly observed (Figs 10F and 16), and the nano-scale uranium mineral are also commonly observed associated with loose and porous titanium oxides (Figs 10G, H). The uranium minerals are adsorbed at the edges or in the dissolution pores of this type of titanium oxides, and eventually formed a mixed mineral phase containing uranium and titanium, and occurring in the sandstones in the form of titanium-uranium “mixture”. This titanium-uranium “mixture” phase mineral is rich in the sandstone-type uranium deposit in the southern Songliao Basin, and can be observed under a transmission electron microscope. It is the nanoscale uranium mineral (coffinite, pitchblende) adsorbed at the edges or in the dissolution pores of the titanium oxides, rather than the brannerite-like described in previous studies. The nano-scale uranium minerals is characterized by relatively high content of TiO_2_, with a large difference in UO_2_ contents. The content of UO_2_ adsorbed in the pores or at the edges of the titanium oxides is relatively low, whereas the content of UO_2_ adsorbed in chlorite is relatively high. The total chemical component amount of the nano-scale uranium minerals ranges from 92.05% to 95.65%, with an average of 93.88%. Specifically, the contents of UO_2_, TiO_2_, FeO, SiO_2_ range from 9.15% to 60.68%, 19.05% to 67.00%, 0.13% to 4.21% and 2.25% to 11.57%, respectively, averaging 31.98%, 45.82%, 2.33% and 7.41%, respectively; the average contents of CaO, Al_2_O_3_ and ZrO_2_ are 1.80%, 1.67% and 1.13%, respectively. Additionally, the nano-scale uranium minerals also contain a small amount of impurities such as Y_2_O_3_ and P_2_O_5_ (S3 Table).

#### 4.4.3. Isomorphism substitution mineral.

The minerals such as uranium-rich rutile (Fig 10J) and monazite (Fig 10I) can be occasionally observed under the microscope in the uranium ore samples from the Louzhuangzi area. The distribution of uranium in these minerals is extremely uneven (Figs 10K, L). These uranium-containing minerals may have been formed through the isomorphism substitution of Ti^4+^ and REE^3+^ by uranium in the source rocks, and then occurred in the sandstones as a result of source rock weathering and crushing, and sediment transportation and deposition. During the uranium mineralization process, uranium-rich rutile and monazite, due to the later-stage fluid alteration effect, can serve as potential uranium sources in depositional sandstone units, and can promote the formation of uranium mineralization.

### 4.5. Alteration features

#### 4.5.1. Interlayer oxidation.

**Fig 12:**
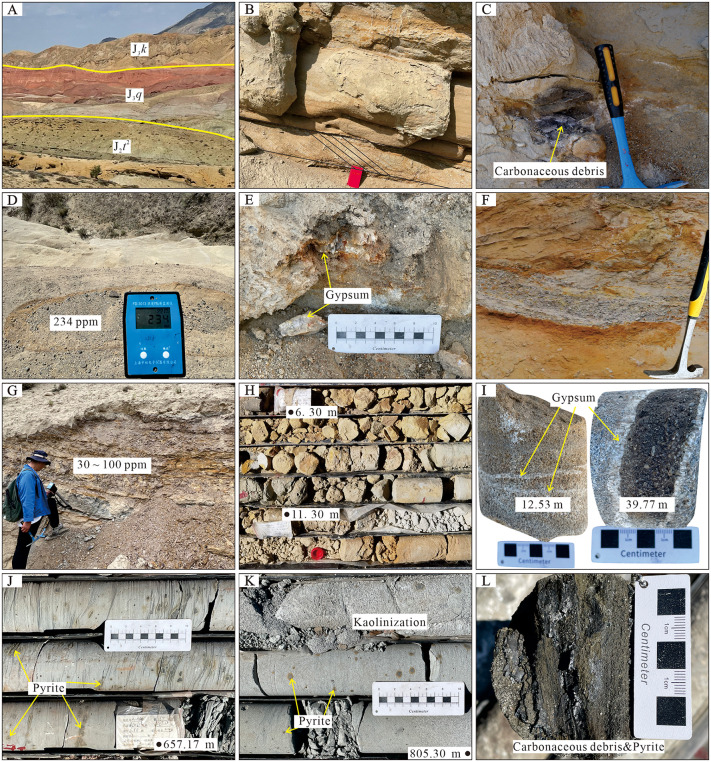
Epigenetic alteration characteristics of sandstones from the Toutunhe Formation (J_2_*t*) in the Louzhuangzi area. **(A–D)** Field characteristics of sand-yellow oxidized sandstone from the Upper Member of the Toutunhe Formation (J_2_*t*^2^) at the Wanjiayao mineralization site: **(A)** Oxidized sandstone outcrop; **(B)** Medium to large planar cross-bedding developed in sandstone; **(C)** Incompletely oxidized carbonized plant detritus and sporadic layered gypsum; **(D)** Uranium anomalies developed in gravel-bearing coarse sandstone. **(F–G)** Incompletely oxidized gray residues (F) and uranium anomalies (G) within sand-yellow oxidized sandstone of the Lower Member of the Toutunhe Formation (J_2_*t*^1^) at the Hasa Tomb anomaly site. (H–I) Oxidized sandstone (H) and multi-layered gypsum (I) of the Upper Member of the Toutunhe Formation (J_2_*t*^2^) from drill hole ZK11−3. **(J)** Laminated carbonized plant detritus and disseminated pyrite in grayish-white altered sandstone of the Lower Member of the Toutunhe Formation (J_2_*t*^1^) from drill hole ZK6−4. **(K)** Kaolinite aggregates and pyrite nodules developed in grayish-white altered sandstone of the Lower Member of the Toutunhe Formation (J_2_*t*^1^) from drill hole ZK12−3. **(L)** Laminated carbonaceous debris and pyrite occurring at a depth of 758.24 m in drill hole ZK4−3, Lower Member of the Toutunhe Formation (J_2_*t*^1^).

At the Wanjiayao mineral occurrence, located northeast of the Louzhuangzi area, brownish-yellow discoloration is observed in outcrops of sandstones from the Upper Member of the Toutunhe Formation (J_2_*t*^2^) (Figs 12A, B). Relatively large fragments of carbonaceous debris occur locally and show no obvious color contrast with the surrounding matrix (Fig 12C). Uranium anomalies are identified within brownish-yellow glutenite (Fig 12D), and the underlying gypsum layers locally exhibit partial discoloration and textural modification (Fig 12E).

At the Hasa Tomb anomaly site, east of the Louzhuangzi area, sandstones of the Lower Member (J_2_*t*^1^) display heterogeneous brownish-yellow staining and mottled coloration (Fig 12F). Some stained surfaces are partially covered by Quaternary surficial deposits; after removal of the cover, the discoloration remains clearly visible and is spatially associated with zones of elevated uranium mineralization (Fig 12G).

Further east of the Louzhuangzi area, an interlayer oxidation zone in the Upper Member (J_2_*t*^2^) is intersected by drill hole ZK11−3 at a depth of approximately 40.9 m. The oxidized sandstones contain multiple gypsum layers (Fig 12I). Except for the intervals intersected by drill holes ZK11−2 and ZK11−3, most sandstones from the Toutunhe Formation (J_2_*t*) encountered in other drill holes are gray to grayish-white (Figs 7B, 8, 9) and contain abundant carbonized plant detritus and pyrite (Figs 12J–L). Microscopic observations reveal residual traces of ilmenite oxidation and alteration (Figs 17A, B).

**Fig 13:**
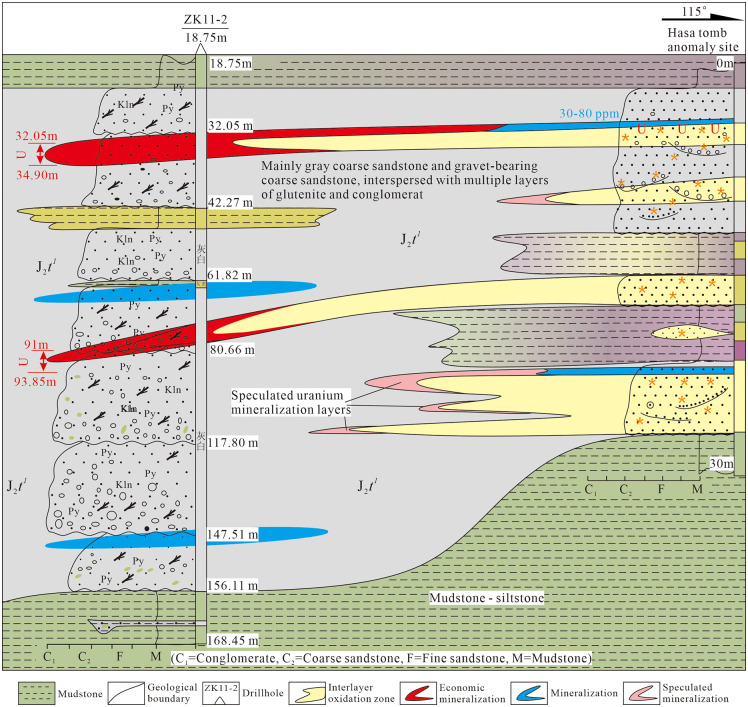
Profile of interlayer oxidation zones and uranium mineralization zones in sandstones of the Toutunhe Formation (J_2_*t*) from drill hole ZK11−2 to the Hasa Tomb (the top boundary of sandstone intervals is horizontally leveled).

**Fig 14:**
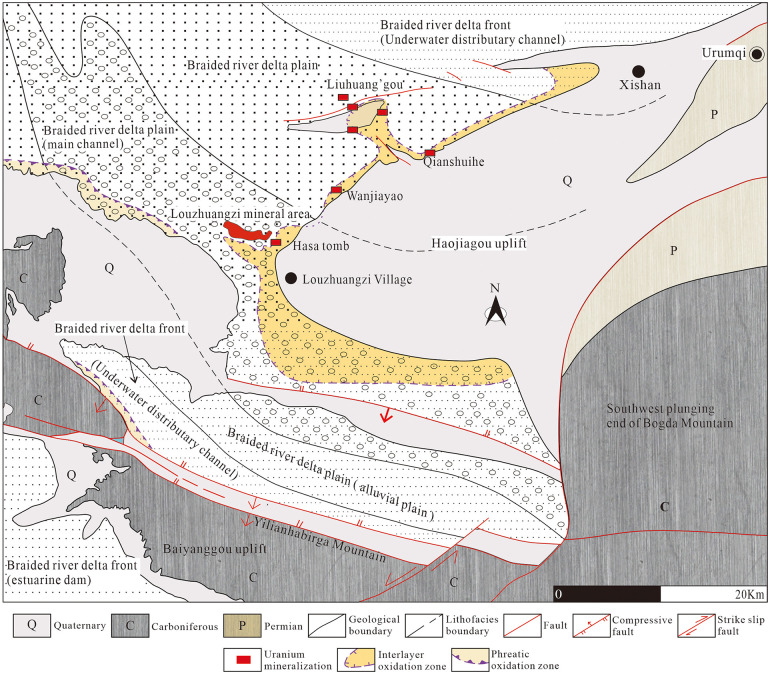
Distribution of depositional facies and interlayer oxidation zones in the Lower Member of Toutunhe Formation (J_2_*t*^1^), Louzhuangzi area (modified from [18]). Reprinted from [Lu **K.**G, Du M, Sun X, Jia **W.**W, Wang **S.**Y. Metallogenic controlling factors and genetic analysis of sandstone uranium deposit in the lower member of Toutunhe Formation in Louzhuangzi area, Southern Junggar Basin. 2023; 39(4):507–521.] under a CC BY license, with permission from [Uranium Geology], original copyright [2023].

Field investigations indicate that the interlayer oxidation zone extends in a belt-like pattern from the Hasa Tomb anomaly site eastward through Wanjiayao, Liuhuang’gou, and Qianshuihe. Based on outcrop and drill hole constraints, the total length of the oxidation front is approximately 20 km (Fig 14). Drill hole ZK11−2 shows that uranium-mineralized sandstones in the Lower Member (J_2_*t*^1^) correspond spatially to oxidized sandstones at the Hasa Tomb anomaly site. Each mineralized interval and uranium anomaly identified in this drill hole is aligned with a corresponding segment of the interlayer oxidation zone located to the east (Fig 13). The interlayer oxidation zone is spatially associated with the Haojiagou Uplift and shows an overall trend subparallel to the uplift zone (Fig 14) (Lu, 2023).

#### 4.5.2. Clayification.

**Fig 15:**
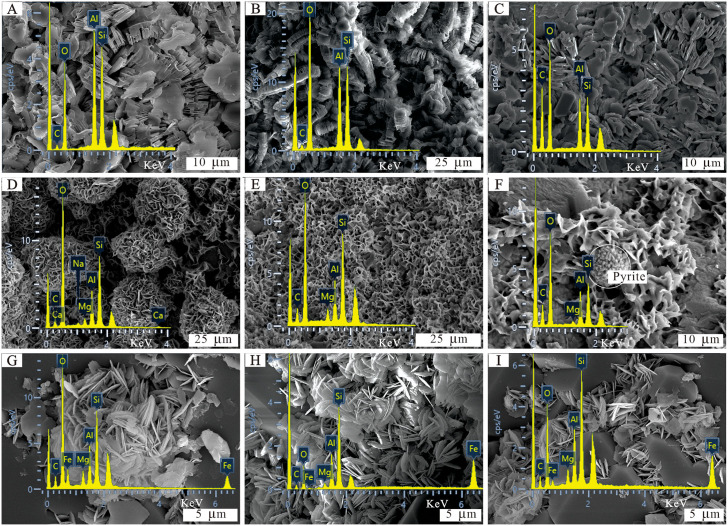
Clay mineral characteristics of sandstones in the Lower Member of the Toutunhe Formation (J_2_*t*^1^), Louzhuangzi area (BSE images). **(A)** Intergranular layered kaolinite, ZK16−2, 23DJA-9, 705.70 m; **(B)** Intergranular vermiform kaolinite, ZK16−2, 23DJA-6, 654.60 m; **(C)** Intergranular platy kaolinite, ZK12−3, 23DJA-27, 641.70 m; **(D)** Spherical smectite mixed-layer minerals on grain surfaces, ZK4−3, 23DJA-7, 710.40 m; **(E)** Cellular smectite mixed-layer minerals on grain surfaces, ZK12−3, 23DJA-15, 871.50 m; **(F)** Strawberry-like pyrite encapsulated by flaky smectite mixed-layer minerals, ZK12−2, 23DJA-4, 680.70 m; (G–I) Intergranular flaky chlorite aggregates, ZK16−2, 23DJA-9, 705.70 **m.**

**Fig 16:**
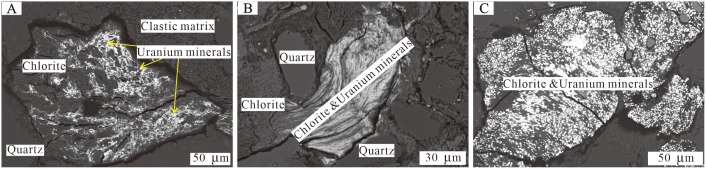
Association of uranium minerals and clay minerals in sandstones of the Lower Member of the Toutunhe Formation (J_2_*t*^1^), Louzhuangzi area (BSE images). **(A)** Uranium minerals adsorbed on chlorite, ZK3−3, 23ZGe027, 753.10 m; **(B)** Uranium minerals associated with chloritized biotite, ZK4−2, 23ZGE026, 398.20 m; **(C)** Uranium minerals associated with chlorite, ZK12−3, 23ZGE028, 798.50 **m.**

The gray to grayish-white sandstone alteration zone in the Lower Member of the Toutunhe Formation (J_2_*t*^1^) in the Louzhuangzi area (Figs 7B, 8, 9) shows a close spatial association with uranium mineralization. The ore-bearing sandstones are predominantly composed of glutenite and exhibit well-developed clay alteration (Figs 8, 15–16).

Scanning electron microscopy reveals abundant clay minerals within the altered sandstones. The dominant clay mineral assemblage includes kaolinite (Figs 15A–C), smectite (Figs 15D–F), and chlorite (Figs 15G–I), with minor sericite (Figs 5B, 6C). These clay minerals commonly occur as pore-filling, grain-coating, and aggregate phases. Uranium-bearing phases are observed to occur in close association with clay minerals, forming flake-like and locally massive aggregates (Fig 16).

#### 4.5.3. Ti-Fe mineral alteration.

**Fig 17:**
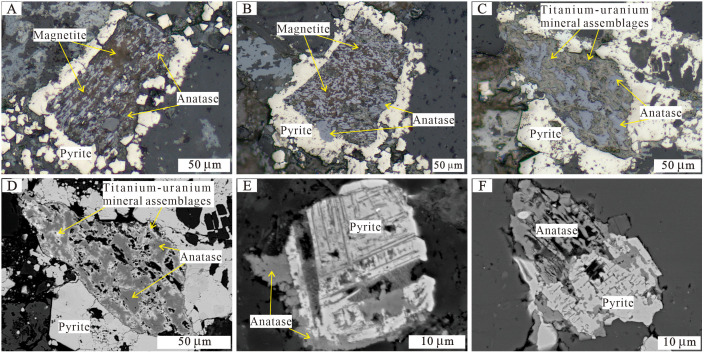
Alteration characteristics of titanium oxides in sandstones of the Lower Member of the Toutunhe Formation (J_2_*t*^1^), Louzhuangzi area. **(A–C)** Assemblage of anatase and magnetite enveloped by colloform pyrite (reflected light); **(D)** Uranium-bearing oxides enveloped by colloform pyrite; **(E–F)** Intergrown association of anatase and pyrite (BSE image).

Ti-Fe mineral assemblages are commonly observed in thin sections of gray to grayish-white sandstones from the Lower Member of the Toutunhe Formation (J_2_*t*^1^). These particles are predominantly angular to sub-angular in morphology (Figs 17A, B), with a smaller proportion showing sub-rounded to rounded shapes (Figs 17C, D). Many grains exhibit textural features indicative of alteration, including anatase and magnetite commonly occur together (Figs 17A, B), and pyrite is frequently observed along grain margins or surrounding these assemblages (Figs 17A-D). In some cases, titanium oxide particles are partially or completely replaced by pyrite, while relic crystal structures are locally preserved (Figs 17E, F). Uranium-bearing phases are commonly observed in close spatial association with altered titanium oxides. These phases occur along grain edges or within pore spaces and are locally surrounded by fine-grained or colloform pyrite (Figs 17C, D).

**Fig 18:**
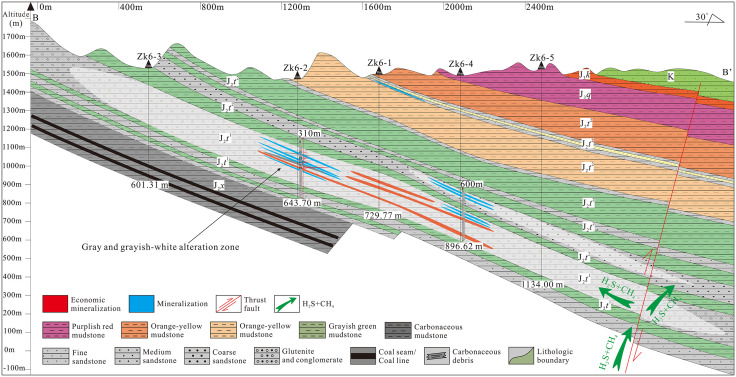
Profile of the B-B’ drill hole in Louzhuangzi area. The profile position is shown in Fig 1C (modified from [14,20]).

Near-surface oxidized sandstones are only encountered in the eastern part of the Louzhuangzi area in drill holes ZK11−2 and ZK11−3 along the trend of the uranium ore bodies. In contrast, sandstones intersected by drill holes up to ~6 km west of the ore zone are consistently gray to grayish-white (Figs 7B, 8, 9, 18). Petrographic observations indicate that the ore-bearing sandstones are characterized by widespread sulfide mineral development, particularly pyrite (Fig 17).

## 5. Discussion

### 5.1. Constraints of depositional architecture on uranium mineralization

In the Upper Member (J_2_*t*^2^), thick mudstone intervals with massive bedding (Fig 3B) indicate deposition under low-energy conditions, such as floodplains or abandoned channels, where fine-grained sediments settle from suspension under weak hydrodynamic conditions [54–56]. Flaser bedding in fine sandstone (Fig 3C) reflects alternating traction and suspension processes under fluctuating flow regimes, suggesting intermittent current activity and sediment reworking [13,57,58]. Horizontal bedding in mudstone and sandstone (Fig 3B) is characteristic of low-energy, laterally extensive sheet-like deposition in floodplain or overbank settings [54,59]. Parallel bedding in sandstone (Fig 3A) indicates deposition under relatively higher flow conditions within the upper flow regime, associated with planar laminar flow [60, 61]. Small- to medium-scale planar cross-bedding (Figs 3C and 12B) records bedform migration and lateral sediment transport under persistent current activity, consistent with fluvial systems of moderate hydrodynamic energy [62, 63].

In the Lower Member (J_2_*t*^1^), the dominance of thick coarse sandstone, glutenite, and conglomerate (Fig 7B), together with the development of channel-fill, lag-deposit, and gravel-bar microfacies (Fig 8), indicates deposition in a braided-river system characterized by high-energy channel processes [64, 65]. Large-scale planar cross-bedding in glutenite (Fig 3D) reflects downstream-accreting barforms and rapid sediment accumulation under strong current conditions [64–66]. Trough cross-bedding in glutenite (Fig 3F) and massive-bedded conglomerate (Fig 3E) further indicate channel erosion followed by rapid infill, typical of gravelly braided fluvial systems [56,67]. The sandy-dominated association at the base of the Lower Member (J_2_*t*^1^), associated with climbing ripples and scour surfaces (Fig 8), suggest deposition in a sandy braided-river environment under moderate to high flow conditions [68].

The upward decrease in conglomerate and coarse sandstone, accompanied by an increase in siltstone and fine sandstone from the Lower to the Upper Member, reflects a progressive reduction in hydrodynamic energy [13,62]. The occurrence of multiple gypsum layers (Figs 12E, I) further indicates progressively increasing aridity and evaporitic conditions, reflecting a transition toward a more restricted and shallow depositional environment [43,69,70].

Uranium mineralization in the Lower Member Toutunhe Formation (J_2_*t*^1^) in the Louzhuangzi area is primarily controlled by braided channel depositional architecture and subsequent fluid-induced alteration [18]. Matrix-rich braided channel lag deposits constitute the principal ore- bearing facies [19]. These facies-controlled sandstones are characterized by high primary permeability and enhanced geochemical reactivity along grain–matrix interfaces, thereby providing favorable pathways for the migration of uranium-bearing oxidizing fluids and the development of localized reduction fronts [71–74].

### 5.2. Sandstone alteration conditions and the response to uranium mineralization

A well-defined redox zonation characterizes the Louzhuangzi deposit, with oxidized sandstones in the eastern sector grading into reduced sandstones in the western sector. This pattern reflects the development of an interlayer oxidation front and exerts a first-order control on the spatial distribution of uranium mineralization [18] (Figs 13, 14). The ore-bearing sandstones of the Toutunhe Formation (J_2_*t*) display well-developed interlayer permeability architectures [21], facilitating fluid migration. Their low compositional maturity, characterized by abundant feldspar and lithic fragments, renders them highly reactive to chemical weathering and diagenetic alteration [75–77]. Although compaction reduces primary porosity, alteration preserves reactive grain boundaries [73]. Under oxidizing to weakly acidic conditions, dissolution of unstable components releases Ca, Na, and K, promoting secondary porosity and authigenic clay mineral formation (kaolinite, smectite, chlorite). These processes enhance fluid–rock interaction and generate abundant adsorption and retention sites for uranium [78, 79].

During mineralization, oxidized, uranium-bearing fluids preferentially migrated through permeable sandstones and underwent enrichment upon encountering reductive and/or adsorptive components, including carbonaceous debris preserved during sandstone deposition (Fig 5H), as well as fine-grained pyrite (Figs 5H, 5I, 10A-10C), porous anatase (Figs 10G, 10H, 17D), and clay minerals (Figs 10F, 16A-C) formed during diagenesis [10,22,80,81]. In the Lower Member of Toutunhe Formation (J_2_*t*^1^) sandstones, the abundance of porous TiO_2_ and clay minerals markedly increases reactive surface area, promoting surface-mediated adsorption and nucleation of uranium. This leads to characteristic uranium associations with Ti oxides (Fig 10G–H, 10J; 17C, D) and clay minerals [10,21,82–87] (Figs 10E–F; 16). However, the presence of nanoscale brannerite-like aggregates (Figs 10E, 11) indicates that the initial redox-controlled uranium mineralization was subsequently modified by hydrothermal fluids [88].

**Fig 19:**
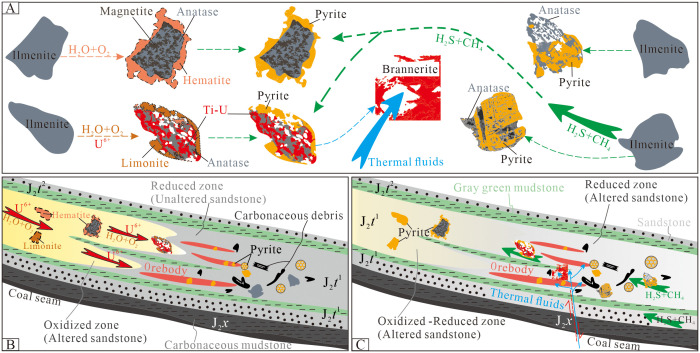
Schematic diagram of uranium mineralization model in the Louzhuangzi area. **(A)** Types of mineral alteration under different fluid environments; **(B)** Mineralization induced by interlayer oxidation fluids; **(C)** Thermal fluid superimposed transformation coupled with hydrocarbon reduction.

Additionally, the alteration of Fe–Ti minerals provide a sensitive record of fluid evolution. The transformation of ilmenite to anatase and magnetite (Figs 17A–D) reflects the influence of oxidizing fluids during diagenetic to post-diagenetic stages [10,81]. The development of colloform pyrite rims along altered Ti oxides (Figs 17A–D), together with their intimate association with anatase (Figs 17E, F), indicates the post-ore influx of strongly reducing fluids (e.g., H_2_S and/or CH_4_) into the sandstone [89]. The ingress of these reducing fluids triggered localized re-reduction and bleaching of the Toutunhe Formation (J_2_*t*) sandstones (Figs 18, 19), effectively inhibiting uranium remobilization and oxidative degradation, thereby enhancing ore preservation [14,20,21].

Overall, uranium mineralization in the Louzhuangzi deposit reflects a tightly coupled sediment–alteration–fluid system. Low compositional maturity provides a reactive substrate, diagenetic alteration generates porosity and adsorption interfaces, and redox zonation dictates sites of uranium precipitation. The superposition of multi-stage fluids—including surficial oxidizing uranium-bearing fluids, hydrothermal fluids, and deep-seated reducing fluids—collectively governs uranium enrichment, modification, and preservation [7,21–23,27,30,79,90–94] (Fig 19). The proposed multi-stage coupled metallogenic model (Fig 19) underscores the integrated control of sedimentation, alteration, and fluid evolution. However, the temperature, sources, and spatiotemporal evolution of these fluids remain to be constrained by further fluid inclusion and stable isotope analyses.

## 6. Conclusions

The uranium mineralization in the Louzhuangzi area, hosted within sandstone of the Toutunhe Formation (J_2_*t*), is dominated by lithic arkose and feldspathic litharenite with low compositional maturity, which reflects a proximal depositional setting. Uranium mineralization is preferentially developed within permeable braided-channel sandstone units, underscoring the primary control exerted by depositional architecture on uranium accumulation. At the microscale, uranium minerals show a close spatial association with organic matter, pyrite, titanium oxide particles, and clay minerals, indicating that multiple reductants and adsorption interfaces collectively govern uranium precipitation through coupled redox–adsorption processes. Uranium predominantly occurs as pitchblende, coffinite, and Ti–U mineral aggregates, including nanocrystalline brannerite-like phases. These mineralogical and textural features record a multi-stage fluid evolution, involving the migration of early oxidizing uranium-bearing fluids, subsequent hydrothermal overprinting, and localized reduction associated with hydrocarbon-bearing fluids. Such multi-fluid interaction reflects the integrated influence of surface-derived and deep-seated fluids, which plays a critical role in uranium mobilization and enrichment.

Overall, this study highlights the significance of multi-fluid coupling and nanoscale mineralization processes in sandstone-hosted uranium systems, and points to the need for future work aimed at quantitatively constraining fluid sources and timing, as well as elucidating the formation mechanisms of nanocrystalline brannerite-like aggregates, to further refine genetic models.

## Supporting information

S1 Table Statistical table of clastic composition of Toutunhe Formation (J_2_*t*) sandstone (n = 46) in Louzhuangzi area. (DOCX)

S2 Table Basic information of major sandstone samples for microscopic lithofacies analysis. (DOCX)

S3 Table Analysis results of chemical components in uranium-rich minerals in Louzhuangzi area by electron probe (Wt %). (DOCX)

S1 File Highlights. (DOCX)
