# Supplementary material for: Depositional architecture and post-depositional alteration of the Toutunhe Formation (J2t) in the Louzhuangzi area, Southern Junggar Basin: Implications for uranium mineralization
Source: PLoS One. 2026 Jun 16;21(6):e0351337. doi: 10.1371/journal.pone.0351337 (PMC13271488; doi:10.1371/journal.pone.0351337)
Supplement: S1 Table — (DOCX) [file pone.0351337.s001.docx]

Table1 Statistical table of clastic composition of Toutunhe Formation (J_2_*t*) sandstone (n=68) in Louzhuangzi area

| Sample number | Drill | Depth (m) | Lithology | Q | F | R | Q\F+R |
| --- | --- | --- | --- | --- | --- | --- | --- |
| 22ZGE001 | ZK12-2 | 672.48 | Grey gravelly coarse sandstone | 39 | 26 | 35 | 0.64 |
| 22ZGE002 |  | 668.50 | gray conglomerate | 38 | 20 | 42 | 0.61 |
| 22ZGE003 |  | 641.64 | Grey gravelly coarse sandstone | 35 | 25 | 40 | 0.54 |
| 22ZGE004 |  | 765.60 | Grey gravelly coarse sandstone | 53 | 19 | 28 | 1.13 |
| 22ZGE005 |  | 755.00 | Gray coarse sandstone | 44 | 26 | 30 | 0.79 |
| 22ZGE006 |  | 736.20 | Grey medium sandstone | 45 | 30 | 25 | 0.82 |
| 22ZGE007 |  | 713.50 | Gray fine sandstone | 52 | 38 | 10 | 1.08 |
| 22ZGE008 |  | 694.50 | Grey gravelly medium-coarse sandstone | 33 | 29 | 38 | 0.49 |
| 22ZGE009 |  | 690.45 | Grey medium sandstone | 40 | 31 | 29 | 0.67 |
| 22ZGE010 |  | 669.20 | Grey medium-fine sandstone | 46 | 39 | 15 | 0.85 |
| 22ZGE011 |  | 657.00 | Gray coarse sandstone | 38 | 27 | 35 | 0.61 |
| 22ZGE012 |  | 645.74 | Grey gravelly coarse sandstone | 37 | 23 | 40 | 0.59 |
| 22ZGE013 |  | 624.65 | Grey medium-fine sandstone | 43 | 39 | 18 | 0.75 |
| 22ZGE014 | ZK6-2 | 483.47 | Grey gravelly coarse sandstone | 41 | 24 | 35 | 0.69 |
| 22ZGE015 |  | 479.30 | Grey gravelly coarse sandstone | 37 | 21 | 42 | 0.59 |
| 22ZGE016 |  | 477.30 | Grey gravelly coarse sandstone | 36 | 25 | 39 | 0.56 |
| 22ZGE021 |  | 486.08 | Grey gravelly coarse sandstone | 35 | 24 | 41 | 0.54 |
| 22ZGE022 |  | 484.98 | Gray coarse sandstone | 37 | 28 | 35 | 0.59 |
| 22ZGE023 |  | 476.20 | Gray coarse sandstone | 40 | 27 | 33 | 0.67 |
| 22ZGE024 |  | 475.70 | Gray medium-coarse sandstone | 40 | 32 | 28 | 0.67 |
| 22ZGE026 |  | 398.80 | Grey gravelly coarse sandstone | 48 | 15 | 37 | 0.92 |
| 22ZGE027 |  | 393.10 | Grey medium-fine sandstone | 53 | 25 | 22 | 1.13 |
| 22ZGE028 |  | 395.40 | Grey medium sandstone | 48 | 23 | 29 | 0.92 |
| 22ZGE063 |  | 478.60 | Grey gravelly coarse sandstone | 44 | 17 | 39 | 0.79 |
| 22ZGE029 | ZK5-1 | 214.12 | Gray medium-coarse sandstone | 40 | 25 | 35 | 0.67 |
| 22ZGE030 |  | 200.74 | Gray coarse sandstone | 43 | 20 | 37 | 0.75 |
| 22ZGE031 |  | 193.00 | Grey medium sandstone | 47 | 28 | 25 | 0.89 |
| 22ZGE032 |  | 168.60 | Gray coarse sandstone | 44 | 20 | 36 | 0.79 |
| 22ZGE033 |  | 148.50 | Grey gravelly coarse sandstone | 40 | 18 | 42 | 0.67 |
| 22ZGE034 | ZK11-2 | 24.84 | Gray coarse sandstone | 32 | 29 | 39 | 0.47 |
| 22ZGE035 |  | 39.97 | Grey fine conglomerate | 39 | 18 | 43 | 0.64 |
| 22ZGE036 | ZK3-1 | 388.20 | Grey gravelly coarse sandstone | 37 | 25 | 38 | 0.59 |
| 22ZGE037 |  | 350.10 | Grey gravelly coarse sandstone | 41 | 23 | 36 | 0.69 |
| 22ZGE038 |  | 424.43 | Grey gravelly coarse sandstone | 40 | 21 | 39 | 0.67 |
| 22ZGE039 |  | 414.77 | Grey medium-fine sandstone | 42 | 29 | 29 | 0.72 |
| 22ZGE040 |  | 473.56 | Grey gravelly coarse sandstone | 46 | 15 | 39 | 0.85 |
| 22ZGE041 | ZK5-4 | 181.13 | Grey gravelly coarse sandstone | 44 | 18 | 38 | 0.79 |
| 22ZGE042 |  | 167.50 | Grey fine sandstone | 59 | 27 | 14 | 1.44 |
| 22ZGE045 | ZK8-5 | 103.38 | Gray coarse sandstone | 41 | 25 | 34 | 0.69 |
| 22ZGE046 |  | 89.45 | Grey fine conglomerate | 38 | 17 | 45 | 0.61 |
| 22ZGE047 |  | 61.33 | Gray coarse sandstone | 36 | 26 | 38 | 0.56 |
| 22ZGE048 |  | 30.57 | Grayish-green fine sandstone | 43 | 39 | 18 | 0.75 |
| 22ZGE025 | ZK1-1 | 209.96 | Grey medium-fine sandstone | 42 | 35 | 23 | 0.72 |
| 23ZGE001 | ZK17-2 | 153.7 | Grayish-white fine sandstone | 33 | 20 | 47 | 0.49 |
| 23ZGE002 |  | 153.92 | Grayish-white fine sandstone | 40 | 32 | 28 | 0.67 |
| 23ZGE003 |  | 155.62 | Grayish-white coarse sandstone | 35 | 15 | 50 | 0.54 |
| 23ZGE004 |  | 152.43 | Grayish-white medium sandstone | 38 | 32 | 30 | 0.61 |
| 23ZGE005 |  | 151.92 | Grayish-white coarse sandstone | 25 | 45 | 30 | 0.33 |
| 23ZGE006 |  | 151.62 | Grayish-white gravelly coarse sandstone | 20 | 20 | 60 | 0.25 |
| 23ZGE007 |  | 147.42 | Grayish-white coarse sandstone | 38 | 24 | 38 | 0.61 |
| 23ZGE008 |  | 43.60 | Grayish-white coarse sandstone | 42 | 30 | 28 | 0.72 |
| 23ZGE009 |  | 38.70 | Grayish-white medium sandstone | 30 | 50 | 20 | 0.43 |
| 23ZGE010 |  | 40.20 | Grayish-white fine sandstone | 25 | 30 | 45 | 0.33 |
| 23ZGE011 |  | 27.90 | Grayish-green fine sandstone | 48 | 25 | 27 | 0.92 |
| 23ZGE014 |  | 945.73 | Grayish-white coarse sandstone | 35 | 35 | 30 | 0.54 |
| 23ZGE015 |  | 933.14 | Grayish-white medium sandstone | 23 | 32 | 45 | 0.3 |
| 23ZGE017 |  | 799.50 | Grayish-white coarse sandstone | 20 | 25 | 55 | 0.25 |
| 23ZGE018 |  | 754.80 | Grayish-white gravelly coarse sandstone | 20 | 20 | 60 | 0.25 |
| 23ZGE019 | ZK6-4 | 654.61 | Grayish-white coarse sandstone | 44 | 20 | 36 | 0.79 |
| 23ZGE020 | ZK11-3 | 135.52 | Grayish-white coarse sandstone | 25 | 25 | 50 | 0.33 |
| 23ZGE021 |  | 132.85 | Grayish-white gravelly coarse sandstone | 20 | 30 | 50 | 0.25 |
| 23ZGE022 |  | 119.52 | Grayish-white gravelly coarse sandstone | 18 | 22 | 60 | 0.22 |
| 23ZGE024 |  | 79.20 | Grayish-white gravelly coarse sandstone | 38 | 27 | 35 | 0.61 |
| 23ZGE025 |  | 40.90 | Grayish-white coarse sandstone | 35 | 25 | 40 | 0.54 |
| 23ZGE026 | ZK4-2 | 398.20 | rey white coarse sandstone | 48 | 29 | 23 | 0.92 |
| 23ZGE027 | ZK3-3 | 753.10 | Grey white fine sandstone | 42 | 23 | 35 | 0.72 |
| 23ZGE016 | ZK12-3 | 838.64 | Grey gravel-bearing coarse sandstone | 20 | 40 | 40 | 0.25 |
| 23ZGE028 |  | 798.50 | Grey gravel-bearing coarse sandstone | 46 | 33 | 21 | 0.85 |
| Minimum |  |  |  | 18 | 15 | 10 | 0.22 |
| Maximum |  |  |  | 59 | 50 | 60 | 1.44 |
| Average |  |  |  | 38 | 26 | 35 | 0.65 |

Q - Quartz; F - Feldspar; R - Rock fragments; Q/(F+R) - Compositional maturity index
