# Supplementary material for: Depositional architecture and post-depositional alteration of the Toutunhe Formation (J2t) in the Louzhuangzi area, Southern Junggar Basin: Implications for uranium mineralization
Source: PLoS One. 2026 Jun 16;21(6):e0351337. doi: 10.1371/journal.pone.0351337 (PMC13271488; doi:10.1371/journal.pone.0351337)
Supplement: S2 Table — (DOCX) [file pone.0351337.s002.docx]

Table 1. Basic information of major sandstone samples for microscopic lithofacies analysis

| Sample number | Drill hole | Sampling depth / (m) | Lithology description | Mineralogical analysis methods | | |
| --- | --- | --- | --- | --- | --- | --- |
| 23DJA-6 | ZK16-2 | 654.60 | Grey glutenite | / | SEM | / |
| 23DJA-9 |  | 705.70 |  | / |  | / |
| 22ZGE001 | ZK12-2 | 672.48 | Grey gravel-bearing coarse sandstone | Optical petrography |  | FIB-TEM |
| 23DJA-4 |  | 680.70 |  |  |  | / |
| 23ZGE016 | ZK12-3 | 838.64 |  | Optical petrography |  | / |
| 23ZGE028 |  | 798.50 |  |  |  | EPMA |
| 23DJA-15 |  | 871.50 | Grey glutenite | / |  | / |
| 23DJA-27 |  | 641.70 |  | / |  | / |
| 22ZGE014 | ZK6-2 | 483.47 | Grey gravel-bearing coarse sandstone | Optical petrography | / | / |
| 22ZGE015 |  | 479.30 |  |  | / | / |
| 22ZGE016 |  | 477.30 |  |  | SEM | / |
| 22ZGE026 |  | 398.80 |  |  |  | / |
| 22ZGE063 |  | 478.60 |  |  |  | / |
| 23ZGE027 | ZK3-3 | 753.10 | Grey white fine sandstone |  |  | EPMA |
| 23ZGE026 | ZK4-2 | 398.20 | Grey white coarse sandstone |  |  | EPMA |
| 23DJA-7 | ZK4-3 | 710.40 | Grey gravel-bearing coarse sandstone | / |  | / |
| 22ZGE033 | ZK5-1 | 148.50 |  | Optical petrography |  | / |
| 22ZGE034 | ZK11-2 | 24.84 | Grey coarse sandstone |  |  | / |
| 23ZGE022 | ZK11-3 | 119.52 | Grey white gravel-bearing coarse sandstone |  |  | EPMA |
| 23ZGE024 |  | 79.20 |  |  |  | / |
