# Supplementary material for: Depositional architecture and post-depositional alteration of the Toutunhe Formation (J2t) in the Louzhuangzi area, Southern Junggar Basin: Implications for uranium mineralization
Source: PLoS One. 2026 Jun 16;21(6):e0351337. doi: 10.1371/journal.pone.0351337 (PMC13271488; doi:10.1371/journal.pone.0351337)
Supplement: S3 Table — (DOCX) [file pone.0351337.s003.docx]

| **Table 2. Analysis results of chemical components in uranium-rich minerals in Louzhuangzi area by electron probe (Wt %).** | | | | | | | | | | | | | | | | |
| --- | --- | --- | --- | --- | --- | --- | --- | --- | --- | --- | --- | --- | --- | --- | --- | --- |
| **Test point** | **SiO_2_** | **Al_2_O_3_** | **MgO** | **Na_2_O** | **Y_2_O_3_** | **CaO** | **P_2_O_5_** | **FeO** | **TiO_2_** | **ZrO_2_** | **PbO** | **MnO** | **UO_2_** | **Total** | **Mineral type** |  |
| 23ZGE022-01 | 18.23 | 0.22 | 0.03 | 0.11 | 0.50 | 3.88 | 1.26 | 0.09 | 0.12 | 0.09 | 0.05 | 0.02 | 66.44 | 91.03 | coffinite |  |
| 23ZGE022-02 | 9.33 | 0.99 | 0.19 | 0.11 | 0.64 | 4.30 | 2.12 | 0.46 | 2.86 | 4.43 | 0.11 | 0.15 | 66.88 | 92.57 | coffinite |  |
| 23ZGE022-03 | 16.39 | 1.24 | 0.19 | 0.01 | 0.92 | 4.88 | 2.79 | 0.62 | 0.28 | 7.24 | 0.05 | 0.16 | 61.58 | 96.36 | coffinite |  |
| 23ZGE022-04 | 11.54 | 0.06 | 0.05 | 0.07 | 0.60 | 4.42 | 3.51 | 1.47 | 0.58 | 3.13 | 0.05 | 0.07 | 66.71 | 92.26 | coffinite |  |
| 23ZGE028-01 | 15.98 | 1.59 | 0.14 | 0.03 | 0.97 | 4.49 | 2.60 | 0.20 | 0.30 | 7.17 | 0.01 | 0.11 | 58.27 | 91.85 | coffinite |  |
| 23ZGE028-02 | 13.27 | 1.08 | 0.13 | 0.05 | 0.72 | 4.26 | 2.65 | 0.30 | 1.73 | 6.75 | 0.05 | 0.01 | 59.24 | 90.23 | coffinite |  |
| 23ZGE028-03 | 14.33 | 1.21 | 0.05 | 0.21 | 4.93 | 7.21 | 5.91 | 0.21 | 0.07 | 0.98 | 0.00 | 0.12 | 54.04 | 89.27 | coffinite |  |
| 23ZGE028-04 | 15.09 | 0.05 | 0.49 | 0.03 | 7.17 | 0.62 | 1.31 | 1.06 | 0.00 | 1.16 | 0.09 | 0.00 | 60.54 | 87.61 | coffinite |  |
| 23ZGE026-01 | 14.28 | 0.05 | 0.39 | 0.08 | 6.21 | 0.65 | 1.06 | 2.66 | 0.00 | 1.28 | 0.06 | 0.02 | 60.99 | 87.72 | coffinite |  |
| 23ZGE026-02 | 16.20 | 0.28 | 1.87 | 0.07 | 7.33 | 0.83 | 1.24 | 1.00 | 0.00 | 0.49 | 0.18 | 0.00 | 58.07 | 87.56 | coffinite |  |
| 23ZGE026-01 | 16.54 | 5.38 | 0.19 | 0.17 | 4.69 | 3.88 | 5.53 | 0.89 | 0.10 | 2.22 | 0.01 | 0.00 | 52.89 | 92.50 | coffinite |  |
| 23ZGE026-03 | 14.26 | 0.24 | 0.09 | 0.03 | 3.24 | 3.39 | 5.30 | 4.59 | 0.21 | 0.08 | 0.08 | 0.00 | 65.62 | 97.14 | coffinite |  |
| 23ZGE026-04 | 11.71 | 0.20 | 0.02 | 0.07 | 3.20 | 3.76 | 4.68 | 4.56 | 0.08 | 0.17 | 0.09 | 0.00 | 65.70 | 94.23 | coffinite |  |
| 23ZGE026-05 | 12.42 | 0.19 | 0.07 | 0.01 | 3.96 | 2.67 | 5.25 | 3.51 | 0.07 | 0.05 | 0.06 | 0.06 | 65.86 | 94.16 | coffinite |  |
| 23ZGE026-06 | 16.46 | 1.68 | 0.21 | 0.08 | 4.36 | 4.21 | 6.44 | 1.84 | 0.48 | 0.82 | 0.00 | 0.06 | 60.37 | 97.01 | coffinite |  |
| 23ZGE026-07 | 13.00 | 0.39 | 0.06 | 0.02 | 5.03 | 1.27 | 3.21 | 1.33 | 3.49 | 1.39 | 0.20 | 0.02 | 58.12 | 87.51 | coffinite |  |
| X̄（averages） | 14.31 | 0.93 | 0.26 | 0.07 | 3.40 | 3.42 | 3.43 | 1.55 | 0.65 | 2.34 | 0.07 | 0.05 | 61.33 | 91.81 |  |  |
| 23ZGE028-05 | 4.34 | 0.02 | 0.03 | 0.19 | 0.26 | 3.45 | 0.36 | 0.33 | 1.88 | 0.69 | 0.06 | 0.36 | 82.87 | 94.83 | pitchblende |  |
| 23ZGE028-06 | 1.20 | 0.00 | 0.04 | 0.10 | 0.00 | 3.56 | 0.30 | 0.32 | 0.35 | 0.31 | 5.21 | 0.39 | 83.60 | 95.38 | pitchblende |  |
| 23ZGE028-07 | 5.76 | 1.72 | 0.24 | 0.27 | 0.00 | 2.79 | 0.17 | 0.92 | 0.64 | 1.16 | 0.02 | 0.47 | 75.50 | 89.66 | pitchblende |  |
| 23ZGE028-08 | 1.95 | 0.02 | 0.09 | 0.28 | 1.52 | 2.39 | 0.05 | 0.62 | 0.00 | 0.03 | 0.05 | 0.54 | 83.33 | 90.85 | pitchblende |  |
| 23ZGE028-09 | 2.75 | 0.00 | 0.07 | 0.55 | 1.81 | 1.21 | 0.30 | 0.45 | 0.00 | 0.00 | 0.00 | 0.19 | 84.25 | 91.58 | pitchblende |  |
| 23ZGE026-08 | 2.40 | 0.03 | 0.06 | 0.49 | 1.30 | 1.74 | 0.14 | 0.71 | 0.00 | 0.16 | 0.05 | 0.38 | 84.67 | 92.12 | pitchblende |  |
| 23ZGE026-09 | 1.73 | 0.01 | 0.05 | 0.26 | 1.18 | 1.86 | 0.08 | 0.60 | 0.00 | 0.05 | 0.01 | 0.46 | 86.07 | 92.33 | pitchblende |  |
| 23ZGE026-10 | 1.05 | 0.00 | 0.00 | 0.06 | 0.36 | 3.99 | 0.25 | 1.18 | 0.25 | 0.63 | 1.26 | 0.73 | 76.82 | 86.57 | pitchblende |  |
| 23ZGE026-11 | 1.11 | 0.00 | 0.00 | 0.09 | 0.36 | 3.55 | 0.24 | 1.04 | 0.57 | 0.56 | 1.47 | 0.67 | 77.06 | 86.72 | pitchblende |  |
| 23ZGE026-12 | 1.30 | 0.01 | 0.01 | 0.07 | 0.33 | 3.07 | 0.22 | 0.94 | 0.94 | 0.79 | 1.28 | 0.90 | 76.44 | 86.29 | pitchblende |  |
| X̄（averages） | 2.36 | 0.18 | 0.06 | 0.24 | 0.71 | 2.76 | 0.21 | 0.71 | 0.46 | 0.44 | 0.94 | 0.51 | 81.06 | 90.63 |  |  |
| 23ZGE027-01 | 6.30 | 0.83 | 0.16 | 0.09 | 0.02 | 2.19 | 0.16 | 0.41 | 33.49 | 2.23 | 0.01 | 0.32 | 39.81 | 86.01 | brannerite |  |
| 23ZGE027-02 | 9.18 | 2.30 | 0.51 | 0.08 | 0.09 | 2.92 | 0.16 | 0.70 | 31.97 | 3.37 | 0.06 | 0.55 | 38.01 | 89.88 | brannerite |  |
| 23ZGE027-03 | 6.88 | 0.83 | 0.06 | 0.30 | 0.03 | 1.07 | 0.17 | 2.05 | 31.67 | 2.64 | 0.00 | 0.24 | 40.40 | 86.33 | brannerite |  |
| 23ZGE027-04 | 5.74 | 0.36 | 1.62 | 0.06 | 0.43 | 0.59 | 0.12 | 0.45 | 33.34 | 1.75 | 0.03 | 0.19 | 39.33 | 84.01 | brannerite |  |
| 23ZGE026-13 | 6.62 | 1.06 | 0.23 | 0.06 | 0.07 | 3.03 | 0.16 | 0.54 | 32.67 | 3.43 | 0.00 | 0.63 | 38.72 | 87.22 | brannerite |  |
| 23ZGE026-14 | 4.14 | 0.74 | 0.09 | 0.34 | 0.12 | 0.65 | 0.12 | 3.99 | 36.09 | 3.86 | 0.00 | 0.24 | 41.46 | 91.83 | brannerite |  |
| X̄（averages） | 6.48 | 1.02 | 0.45 | 0.16 | 0.13 | 1.74 | 0.15 | 1.36 | 33.21 | 2.88 | 0.02 | 0.36 | 39.62 | 87.55 |  |  |
| 23ZGE026-15 | 8.69 | 2.37 | 0.34 | 0.22 | 0.15 | 1.43 | 0.07 | 4.21 | 67.00 | 0.91 | 0.05 | 0.22 | 9.15 | 94.81 | Unidentified nano-uranium minerals |  |
| 23ZGE026-16 | 4.25 | 1.07 | 0.31 | 0.20 | 0.00 | 2.86 | 0.27 | 2.43 | 21.24 | 2.10 | 0.00 | 0.23 | 60.68 | 95.65 |  |  |
| 23ZGE026-17 | 10.30 | 0.11 | 0.02 | 0.07 | 2.43 | 3.60 | 2.07 | 2.45 | 19.05 | 0.10 | 0.02 | 0.10 | 53.24 | 93.55 |  |  |
| 23ZGE028-10 | 2.25 | 0.00 | 0.38 | 0.04 | 0.17 | 0.63 | 0.10 | 0.13 | 60.33 | 1.60 | 0.00 | 0.12 | 26.29 | 92.05 |  |  |
| 23ZGE028-11 | 11.57 | 4.80 | 0.82 | 0.14 | 0.01 | 0.46 | 0.03 | 2.42 | 61.47 | 0.93 | 0.06 | 0.09 | 10.55 | 93.34 |  |  |
| X̄（averages） | 7.41 | 1.67 | 0.37 | 0.13 | 0.55 | 1.80 | 0.51 | 2.33 | 45.82 | 1.13 | 0.03 | 0.15 | 31.98 | 93.88 |  |  |
