# Supplementary material for: Depositional architecture and post-depositional alteration of the Toutunhe Formation (J2t) in the Louzhuangzi area, Southern Junggar Basin: Implications for uranium mineralization
Source: PLoS One. 2026 Jun 16;21(6):e0351337. doi: 10.1371/journal.pone.0351337 (PMC13271488; doi:10.1371/journal.pone.0351337)
Supplement: S1 File — (DOCX) [file pone.0351337.s004.docx]

**Highlights**

- **The ore-controlling sandstone units depositional characteristics of the Upper and Lower Member of Toutunhe Formaiton (J_2_*t*) is determined.**
- **The occurrence state of uranium in the Louzhuangzi area is identified, and the occurrence form of brannerite-like in sandstone-type uranium deposit is confirmed.**
- **A coupling effect of the epigenetic alteration induced by the oxygen- and uranium- containing fluids, the superimposed reformation of the thermal fluids and the oil and gas reduction on uranium mineralization is proposed, revealing a potentially multi-stage alteration induced uranium mineralization mechanism.**
