# Supplementary material for: Depositional architecture and post-depositional alteration of the Toutunhe Formation (J2t) in the Louzhuangzi area, Southern Junggar Basin: Implications for uranium mineralization
Source: PLoS One. 2026 Jun 16;21(6):e0351337. doi: 10.1371/journal.pone.0351337 (PMC13271488; doi:10.1371/journal.pone.0351337)
Supplement: Attachment — Submitted filename: PONE-D-25-34881_R2_reviewer_EJA.pdf [file pone.0351337.s008.pdf]

PLOS One

Sedimentary architecture and post-depositional alteration of the Toutunhe Formation (J2t) in the Louzhuangzi area, Southern Junggar Basin: Implications for uranium mineralization

--Manuscript Draft--

**Manuscript Number:**	PONE-D-25-34881R2
**Article Type:**	Research Article
**Full Title:**	Sedimentary architecture and post-depositional alteration of the Toutunhe Formation (J2t) in the Louzhuangzi area, Southern Junggar Basin: Implications for uranium mineralization
**Short Title:**	Sedimentary architecture and post-depositional alteration of the Toutunhe Formation (J2t)
**Corresponding Author:**	Qing Wang<br>East China University of Technology<br>南昌, CHINA
**Keywords:**	Sandstone-type uranium deposits, Braided river delta, Hydrocarbon-mediated reduction, FIB-TEM/SEM analysis, Toutunhe Formation, Redox transition zone, Northwest China.
**Abstract:**	In recent years, some uranium mineralization with economic values has been discovered in the Louzhuangzi area, southern margin of the Junggar Basin, and a mediu-sized uranium mineral area has been confirmed. To investigate the depositional environment and alteration characteristics of the ore-bearing target horizon—the Toutunhe Formation (J2t)—in this mining area, this study is based on detailed field geological investigations, drill-core logging, and systematic sampling. A preliminary assessment of the depositional environment and uranium mineralization characteristics of the target sandstone units was carried out. Petrographic features of the ore-bearing sandstones were examined using polarized light microscopy and scanning electron microscopy (SEM). Furthermore, electron probe microanalysis (EPMA) and transmission electron microscopy (TEM) were integrated to systematically characterize the mineralogical composition and modes of occurrence of uranium minerals.<br>The results indicate that the upper member of the target horizon (J2t2) exhibits typical meandering river depositional characteristics, manifested by “thick mudstone layers interbedded with thin sandstone layers.” In contrast, economically significant uranium mineralization is hosted within the lower member (J2t1), which displays braided river depositional features characterized by “thick sandstone layers interbedded with thin mudstone layers.” The ore-bearing sandstones show characteristics of proximal-source deposition and have undergone pronounced supergene alteration and post-diagenetic modification. The spatial distribution of uranium is closely associated with pyrite, organic matter, and clay minerals. Uranium minerals are predominantly coffinite and pitchblende, with UO_2 contents ranging from 52.89 % to 86.07 %. A minor proportion of uranium minerals, with UO_2 contents of 38.01 %–41.46 % and TiO_2 contents of 31.67 %–36.09 %, are tentatively interpreted as possible brannerite. In addition, unidentified nano-scale uranium minerals exhibit UO_2 contents ranging from 9.15 % to 60.28 %. Finally, based on the occurrence characteristics of uranium and the results of alteration mineralogical analyses, a conceptual model for uranium mineralization in the Louzhuangzi deposit is proposed. The formation and preservation of uranium mineralization in the target horizon are interpreted as the combined result of surficial and ascending fluid processes, likely governed by three principal factors: (1) the architectural framework of braided-channel sandstones, which provided favorable permeable pathways for fluid migration; (2) infiltration of surficial oxygen-bearing, uranium-rich fluids that initiated uranium mineralization, subsequently overprinted by post-ore thermal fluid modification; and (3) the action of deep-seated reducing fluids, which inhibited remobilization and oxidative destruction of uranium mineralization, thereby contributing to its preservation and stabilization.
**Order of Authors:**	Qing Wang
	Fengjun Nie

Powered by Editorial Manager® and ProduXion Manager® from Aries Systems Corporation

	Fei Xia
	Xin Zhang
	Weiwei Jia
	Kegai Lu
	Xiao Sun
	Guang Dong Yang
**Response to Reviewers:**	Dear Reviewers, Thank you very much for your constructive suggestions on our submitted manuscript. Your feedback has been invaluable in improving its quality. In the resubmitted manuscript, we have made the corresponding revisions based on your suggestions, which are explained in the Cover Letter. Detailed revisions are included in the Revised Manuscript with Track Changes. Specific responses to the revisions are provided in the Response to Reviewers.
**Additional Information:**	
**Question**	**Response**
**Financial Disclosure**<br><br>Enter a financial disclosure statement that describes the sources of funding for the work included in this submission. Review the submission guidelines for detailed requirements. View published research articles from *PLOS ONE* for specific examples.<br><br>This statement is required for submission and **will appear in the published article** if the submission is accepted. Please make sure it is accurate.<br><br>**Funded studies**<br>Enter a statement with the following details:<br>• Initials of the authors who received each award<br>• Grant numbers awarded to each author<br>• The full name of each funder<br>• URL of each funder website<br>• Did the sponsors or funders play any role in the study design, data collection and analysis, decision to publish, or preparation of the manuscript?<br><br>Did you receive funding for this work?	Yes
Please add funding details.<br>as follow-up to "**Financial Disclosure**<br><br>Enter a financial disclosure statement that describes the sources of funding for the	National Natural Science Foundation of China (No. U2244205 and No. U2067202) and the Joint Innovation Fund Project of China Uranium Industry Company Limited - State Key Laboratory of Nuclear Resources and Environment, East China University of Technology (2023NRE-LH-10).

Powered by Editorial Manager® and ProduXion Manager® from Aries Systems Corporation

work included in this submission. Review the submission guidelines for detailed requirements. View published research articles from *PLOS ONE* for specific examples.<br><br>This statement is required for submission and **will appear in the published article** if the submission is accepted. Please make sure it is accurate.<br><br>**Funded studies**<br>Enter a statement with the following details:<br>• Initials of the authors who received each award<br>• Grant numbers awarded to each author<br>• The full name of each funder<br>• URL of each funder website<br>• Did the sponsors or funders play any role in the study design, data collection and analysis, decision to publish, or preparation of the manuscript?<br><br>Did you receive funding for this work?"	
Please select the country of your main research funder (please select carefully as in some cases this is used in fee calculation).<br>as follow-up to "**Financial Disclosure**<br><br>Enter a financial disclosure statement that describes the sources of funding for the work included in this submission. Review the submission guidelines for detailed requirements. View published research articles from *PLOS ONE* for specific examples.<br><br>This statement is required for submission and **will appear in the published article** if the submission is accepted. Please make sure it is accurate.	CHINA - CN

**Funded studies**<br>Enter a statement with the following details:<br>• Initials of the authors who received each award<br>• Grant numbers awarded to each author<br>• The full name of each funder<br>• URL of each funder website<br>• Did the sponsors or funders play any role in the study design, data collection and analysis, decision to publish, or preparation of the manuscript?<br><br>Did you receive funding for this work?"	
**Competing Interests**<br><br>Use the instructions below to enter a competing interest statement for this submission. On behalf of all authors, disclose any competing interests that could be perceived to bias this work—acknowledging all financial support and any other relevant financial or non-financial competing interests.<br><br>This statement is required for submission and **will appear in the published article** if the submission is accepted. Please make sure it is accurate and that any funding sources listed in your Funding Information later in the submission form are also declared in your Financial Disclosure statement.<br><br>View published research articles from *PLOS ONE* for specific examples.	The authors have declared that no competing interests exist.

Powered by Editorial Manager® and ProduXion Manager® from Aries Systems Corporation

**NO authors have competing interests**<br><br>Enter: *The authors have declared that no competing interests exist.*<br><br>**Authors with competing interests**<br><br>Enter competing interest details beginning with this statement:<br><br>*I have read the journal's policy and the authors of this manuscript have the following competing interests: [insert competing interests here]*<br><br>* typeset	
**Ethics Statement**<br><br>Enter an ethics statement for this submission. This statement is required if the study involved:<br><br>• Human participants<br>• Human specimens or tissue<br>• Vertebrate animals or cephalopods<br>• Vertebrate embryos or tissues<br>• Field research<br><br>Write "N/A" if the submission does not require an ethics statement.<br><br>General guidance is provided below. Consult the submission guidelines for detailed instructions. **Make sure that all information entered here is included in the Methods section of the manuscript.**	N/A

**Format for specific study types**<br><br>**Human Subject Research (involving human participants and/or tissue)**<br>• Give the name of the institutional review board or ethics committee that approved the study<br>• Include the approval number and/or a statement indicating approval of this research<br>• Indicate the form of consent obtained (written/oral) or the reason that consent was not obtained (e.g. the data were analyzed anonymously)<br><br>**Animal Research (involving vertebrate animals, embryos or tissues)**<br>• Provide the name of the Institutional Animal Care and Use Committee (IACUC) or other relevant ethics board that reviewed the study protocol, and indicate whether they approved this research or granted a formal waiver of ethical approval<br>• Include an approval number if one was obtained<br>• If the study involved *non-human primates*, add *additional details* about animal welfare and steps taken to ameliorate suffering<br>• If anesthesia, euthanasia, or any kind of animal sacrifice is part of the study, include briefly which substances and/or methods were applied<br><br>**Field Research**<br><br>Include the following details if this study involves the collection of plant, animal, or other materials from a natural setting:<br>• Field permit number<br>• Name of the institution or relevant body that granted permission	
**Data Availability**<br><br>Authors are required to make all data underlying the findings described fully available, without restriction, and from the time of publication. PLOS allows rare exceptions to address legal and ethical concerns. See the PLOS Data Policy and FAQ for detailed information.	Yes - all data are fully available without restriction

A Data Availability Statement describing where the data can be found is required at submission. Your answers to this question constitute the Data Availability Statement and **will be published in the article**, if accepted.<br><br>**Important:** Stating 'data available on request from the author' is not sufficient. If your data are only available upon request, select 'No' for the first question and explain your exceptional situation in the text box.<br><br>Do the authors confirm that all data underlying the findings described in their manuscript are fully available without restriction?	
**Describe where the data may be found in full sentences. If you are copying our sample text, replace any instances of XXX with the appropriate details.**<br><br>• If the data are **held or will be held in a public repository**, include URLs, accession numbers or DOIs. If this information will only be available after acceptance, indicate this by ticking the box below. For example: *All XXX files are available from the XXX database (accession number(s) XXX, XXX.*).<br>• If the data are all contained **within the manuscript and/or Supporting Information files**, enter the following: *All relevant data are within the manuscript and its Supporting Information files.*<br>• If neither of these applies but you are able to provide **details of access elsewhere**, with or without limitations, please do so. For example:<br><br>*Data cannot be shared publicly because of [XXX]. Data are available from the XXX Institutional Data Access / Ethics Committee (contact via XXX) for researchers who meet the criteria for access to confidential data.*<br><br>*The data underlying the results presented in the study are available from (include the name of the third party*	All relevant data are within the manuscript and its Supporting Information files.

and contact information or URL).<br>• This text is appropriate if the data are owned by a third party and authors do not have permission to share the data.<br><br>* typeset	
Additional data availability information:	Tick here if the URLs/accession numbers/DOIs will be available only after acceptance of the manuscript for publication so that we can ensure their inclusion before publication.

Powered by Editorial Manager® and ProduXion Manager® from Aries Systems Corporation

Revised Manuscript with No changes Marked Click here to access/download;Manuscript;Manuscript-Revised Manuscript with No changes Marked.docx

Sedimentary architecture and post-depositional alteration of the Toutunhe Formation (J_2t) in the Louzhuangzi area, Southern Junggar Basin: Implications for uranium mineralization

Qing Wang [1*], Fengjun Nie [1,2*], Fei Xia[1,2], Xin Zhang [1], Weiwei Jia [3], Kegai Lu [3], Xiao Sun [3], Dong-guang Yang[2]

[1] School of Earth and Planetary Sciences, East China University of Technology, Nanchang, Jiangxi, China;

[2] National Key Laboratory of Uranium Resources Exploration-Mining and Nuclear Remote Sensing, East China University of Technology, Nanchang, Jiangxi, China;

[3] No. 216 Geological Party, China National Nuclear Corporation, Urumqi, Xinjiang, China.

* Corresponding author

E-mail: niefj@263.net (FN); destinw@126.com (QW)

Abstract

In recent years, some uranium mineralization with economic values has been discovered in the Louzhuangzi area, southern margin of the Junggar Basin, and a mediu-sized uranium mineral area has been confirmed. To investigate the depositional environment and alteration characteristics of the ore-bearing target horizon—the Toutunhe Formation (J_2t)—in this mining area, this study is based on detailed field geological investigations, drill-core logging, and systematic sampling. A preliminary assessment of the depositional environment and uranium mineralization characteristics of the target sandstone units was carried out. Petrographic features of the ore-bearing

sandstones were examined using polarized light microscopy and scanning electron microscopy (SEM). Furthermore, electron probe microanalysis (EPMA) and transmission electron microscopy (TEM) were integrated to systematically characterize the mineralogical composition and modes of occurrence of uranium minerals.

The results indicate that the upper member of the target horizon (J_2t^2) exhibits typical meandering river depositional characteristics, manifested by "thick mudstone layers interbedded with thin sandstone layers." In contrast, economically significant uranium mineralization is hosted within the lower member (J_2t^1), which displays braided river depositional features characterized by "thick sandstone layers interbedded with thin mudstone layers." The ore-bearing sandstones show characteristics of proximal-source deposition and have undergone pronounced supergene alteration and post-diagenetic modification. The spatial distribution of uranium is closely associated with pyrite, organic matter, and clay minerals. Uranium minerals are predominantly coffinite and pitchblende, with UO_2 contents ranging from 52.89 % to 86.07 %. A minor proportion of uranium minerals, with UO_2 contents of 38.01 %–41.46 % and TiO_2 contents of 31.67 %–36.09 %, are tentatively interpreted as possible brannerite. In addition, unidentified nano-scale uranium minerals exhibit UO_2 contents ranging from 9.15 % to 60.28 %.

Finally, based on the occurrence characteristics of uranium and the results of alteration mineralogical analyses, a conceptual model for uranium mineralization in the Louzhuangzi deposit is proposed. The formation and preservation of uranium mineralization in the target horizon are interpreted as the combined result of surficial

and ascending fluid processes, likely governed by three principal factors: (1) the architectural framework of braided-channel sandstones, which provided favorable permeable pathways for fluid migration; (2) infiltration of surficial oxygen-bearing, uranium-rich fluids that initiated uranium mineralization, subsequently overprinted by post-ore thermal fluid modification; and (3) the action of deep-seated reducing fluids, which inhibited remobilization and oxidative destruction of uranium mineralization, thereby contributing to its preservation and stabilization.

1. Introduction

With the wide application of the in-situ leaching uranium mining technology, sandstone-type uranium deposit has become the main target of uranium resource exploration in China. Sandstone-type uranium deposits in northern China are mainly distributed in Mesozoic-Cenozoic foreland basins, such as Ili, Turpan-Hami, and Songliao basins, as well as the Ordos Basin [1-4]. Uranium source, depositional environment of host sandstones, reduction capacity of sandstones, epigenetic alteration, and regional tectonic activity are the main factors controlling the uranium mineralization [5-8]. The Junggar Basin is one of the important basins in Xinjiang region, northern China where sandstone-type uranium deposits have been conducted. Some promising uranium mineralizations have been discovered in Jurassic, Cretaceous, and Paleogene sandstones in the Dingshan area of the northern basin, the Kamusite area of the eastern basin, and along the southern margin of the basin, attracting considerable attention from the uranium exploration industry [9]. However, the uranium

mineralization with economic values has been discovered only in the Jurassic Toutunhe Formation (J_2t) of the Louzhuangzi area, southern margin of the basin at present, and a medium-sized uranium mineral area has been confirmed.

The sediment source of the Jurassic strata in the southern margin of the Junggar Basin mainly comes from the Northern Tianshan Mountains. The uranium-enriched geological bodies of the Northern Tianshan Mountains, together with conglomerate layers at the base of the Lower Cretaceous Qingshuihe Formation, provide important uranium sources for mineralization. In addition, relatively stable structural areas constitute favorable sites for uranium mineralization [3, 10]. The southern margin of the Junggar Basin was in a tectonically stable state during the period from the Late Cretaceous to Paleogene. The Middle and Lower Jurassic strata experienced long-term interaction with uranium- and oxygen-bearing fluids, leading to the formation of significant uranium mineralization [11-12]. Of which, the thick and large braided river facies sandstones in the Middle Jurassic Toulunhe Formation acts as the host rock of the sandstone-type uranium deposit [9]. The uranium mineralization in the Louzhuangzi mineral area is controlled by the interlayer oxidation zone that develops from the Haojiagou Uplift to the west and northwest directions [13]. The mineralization occurs in the depositional sandstones of the braided river delta plain facies in the Lower member of the Toutunhe Formation (J_2t^1), with the sandstones being stable, thick and large, good in permeability, and rich in pyrite and organic matter [14]. The uranium mineralization area can be classified into the yellow alteration zone and the grayish-white alteration zone. The development of the economic uranium deposit is controlled

by the grayish-white alteration zone resulting from the reduction and bleaching effect of oil and gas in the later stage, in which, the native oxidation alteration zone was reduced to the grayish-white zone due to oil and gas exudation [15-16]. In addition to the local oil and gas exudation that participated in uranium mineralization, the hydrocarbons migrated from other locations were also involved in uranium mineralization [16]. The main occurrence forms of uranium in the Louzhuangzi mineral area include pitchblende, coffinite and titanium-uranium mineral assemblages [15]. In the Toutunhe Formation (J_2t) of the Liuhuang'gou ore site, northeastern the Louzhuangzi mineral area, uranium mostly occurs in organic matter in irregular, grape-like, gelatinous and kidney-like forms, or coexists with clay mineral and pyrite. Uranium at this ore site is mainly hosted by pitchblende and unidentified uranium minerals enriched in SiO_2, P_2O_5, and Y_2O_3, with subordinate brannerite and adsorbed uranium [17].

Currently, two competing genetic hypotheses currently frame the metallogenic theory of sandstone-type uranium deposits, resulting in pronounced academic divergence. The traditional interlayer oxidation-zone model emphasizes the lateral migration of surficial, oxygen-bearing, uranium-rich fluids through permeable sandstones, with uranium precipitation occurring at redox interfaces [18-19]. In contrast, the deep-fluid exhalation or superimposed mineralization model proposes that deep-seated fluids—such as hydrocarbons and thermal fluids—migrate upward, supplying reducing agents that facilitate uranium precipitation or exert superimposed modification on pre-existing mineralization [2, 20-21]. This interpretation has gained

widespread acceptance in the Ordos Basin [22-24], the Songliao Basin [25-26], and the Erlian Basin [27-30].

Previous studies have identified the fundamental geological conditions of uranium mineralization in the Louzhuangzi mining area. Some researchers interpret the mineralization as the product of a single-stage surficial interlayer oxidation process, with the oxidation front acting as the principal ore-controlling factor [13], and argue that uranium enrichment is primarily governed by variations in sedimentary facies [14]. At the same time, other scholars emphasize the critical role of hydrocarbon reduction and thermal-fluid overprinting, proposing that the grayish-white alteration zones are closely linked to deep-seated fluid activity and represent a necessary condition for the formation of economically viable uranium mineralization [15-16, 31]. This divergence in interpretation has resulted in an unresolved understanding of the sedimentary–alteration coupling mechanism controlling uranium mineralization in the Toutunhe Formation along the southern margin of the Junggar Basin. In particular, the temporal and spatial relationships among braided river sandstone architecture, permeability evolution, interlayer oxidation zones, and hydrocarbon-related reduction alteration remain insufficiently constrained. Furthermore, the nature of the metallogenic fluids and the mechanisms of superimposed transformation are still unclear, including the timing, intensity, and relative contributions of surficial oxygen-bearing fluids and deep-seated reducing fluids to uranium precipitation and preservation.

Uranium mineralization in sandstone-type deposits is intrinsically linked to the sedimentary architecture of host sandstones and their subsequent fluid-driven alteration.

The uranium endowment of stratigraphic units is largely governed by dominant sedimentary facies, which are themselves controlled by paleoclimatic conditions and depositional environments [32]. In addition, the alteration characteristics of sandstones provide critical insights into the genetic type and evolution of uranium mineralization.

To elucidate the controlling effects of sandstone sedimentation and post-depositional alteration on uranium mineralization in the Toutunhe Formation (J_2t) along the southern margin of the Junggar Basin, this study integrates sedimentology, petrography, and alteration mineralogy within a multiscale analytical framework. Macroscopic sedimentological observations—including detailed field investigations and petrographic analysis—are coupled with micro- to nanoscale mineralogical characterization using scanning electron microscopy (SEM), electron microprobe analysis (EMPA), and focused ion beam–transmission electron microscopy (FIB–TEM). Based on these integrated investigations, we propose a conceptual model of coupled mineralization control involving braided-river facies sandstone architecture and multi-stage metallogenic fluid processes. This model provides a necessary reference framework for uranium exploration in the Junggar Basin and in other analogous sedimentary basins.

2. Geological background

The Junggar Basin is located on the Kazakhstan, Plate between the Siberian Plate and the Tarim Plate, and is a foreland basin in the plate collision orogenic belt. From the Cenozoic era, the Indian Plate collided with the Eurasian Plate and subducted, causing the Tianshan Orogenic Belt to be subjected to north-south compression [33-36].

During regional compression, the Tarim stable block was located near the center of the compressive regime, resulting in progressive thickening of its lithospheric mantle. In contrast, the Tianshan Orogenic Belt acted as a buffer and stress-release zone, where crustal shortening and uplift gradually intensified. Through prolonged geological evolution, these processes ultimately shaped the present tectonic and geomorphic framework of the Junggar Basin, which is now separated from the Tuha and Ili basins by intervening mountain ranges. [37] (Fig 1A).

The study area is located in the level-II tectonic province at the southern margin of the Junggar Basin (Fig 1B), belonging to the front of the fore-mountain fold-and-thrust belt of the Northern Tianshan Mountains. A thrust fault belt associated with folding structures about 300km long from east to west and nearly 50-80km wide from north to south develops from the fore-mountain fold belt of the northern Tianshan Mountains to the center of the basin [12] (Fig 1B). In recent years, the Louzhuangzi uranium mineral area has been discovered in the intersection and subduction site of the N-WW oriented Yilianhabirga Mountains and the western sloping end of the N-E oriented Bogda Mountain tectonic belt (Haojiagou Uplift) (Fig 1B) in the fore-mountain fold-and-thrust belt of the Northern Tianshan Mountains. This newly discovered uranium mineral area, together with the previously found uranium mineral sites such as Hasa tomb and Wanjiayao, and the uranium anomaly sites, forms a prospective sandstone-type uranium mineralization zone [9, 15] (Fig 1C).

Fig.1 (A) Sketch map showing tectonic location of Junggar Basin (modified from [38]); (B) Geological sketch map of Louzhuangzi area (modified from [15]); (C) Division and distribution of

the main tectonic units at southern margin of Junggar Basin (modified from [12]).

The Cenozoic strata in the southern margin of the Junggar Basin are completely exposed, of which the Jurassic, Cretaceous and Paleogene systems constitute the main horizons for exploring the sandstone-type uranium deposits (Fig 2). The Jurassic strata are widely developed in the Louzhuangzi area (Fig 1C), and the strata revealed by outcrop profile and drilling data from top to bottom include the Karaza Formation (J_3k), Qigu Formation (J_3q), Toutunhe Formation (J_2t), Xishanyao Formation (J_2x), Sangonghe Formation (J_1s), and Badaowan Formation (J_1b). Based on the previous comprehensive study on the sedimentary formation, lithology combination and logging curve characteristics of the Junggar Basin, the Jurassic system has been divided into the river depositional system, the delta depositional system and the lake depositional system [39]. The Karaza Formation (J_3k) and the Qigu Formation (J_3q) are alluvial fan facies deposits, the Toutunhe Formation (J_2t) is a fluvial facies deposit, the Xishanyao Formation (J_2x) is a delta facies deposit, the Sangonghe Formation (J_1s) is a shallow lake – shore shallow lake subfacies deposit, and the Badaowan Formation (J_1b) is a fluvial and swamp facies deposit (Fig 2). Among which, the Toutunhe Formation with fluvial facies deposits is the main uranium-bearing horizon in the Louzhuangzi mineral area (Figs 1C and 2).

Fig. 2 Stratigraphic column of the Louzhuangzi area (modified after an internal report from Geological Party No. 216, CNNC, 2022).

3. Materials and methods

3.1 Fieldwork and sampling

Field investigations were conducted in the Louzhuangzi area on the southern margin of the Junggar Basin. Stratigraphic logging, outcrop observation, and core description were used to define depositional facies and alteration features. Sampling was restricted to the Toutunhe Formation (J_2t), with particular emphasis on the grayish-white alteration sandstones hosting economic uranium mineralization, thereby ensuring close consistency between sampled materials and the research focus on sandstone alteration and its control on uranium mineralization. Core samples were preferentially selected from lithologically intact and unweathered intervals.

To capture the vertical (depth-related) and lateral (lithology-related) mineralogical variations within the grayish-white alteration zone, samples were collected from different depths and lithologies. Sampling sites were systematically arranged from west to east across the Louzhuangzi deposit, spanning the full distribution range of the grayish-white alteration zone within the Toutunhe Formation. Laterally, the western, central, and eastern key sectors of the alteration belt were covered; vertically, sampling depths ranged from 24.84 m to 871.50 m. This strategy enables a comprehensive characterization of the spatial distribution and vertical evolution of the alteration zone and ensures robust spatial representativeness.

Representative drill holes were selected for detailed characterization of internal alteration features, clarification of the relationship between alteration intensity and lithology, and targeted fine-scale sampling. The sample list (Table 1) provided in this

study includes the principal specimens involved in the analytical work and does not represent the complete inventory of systematically collected samples.

3.2 Mineralogical analysis

Thin sections were prepared for petrographic observation under transmitted light using a Zeiss AxioScope.A1 digital polarizing microscope. Mineral assemblages, textures, and diagenetic features were identified and classified.

Scanning electron microscopy (SEM)

Selected samples were carbon-coated and analyzed using a FEI Nova Nano SEM 450 equipped with an energy-dispersive X-ray spectroscopy (EDS) system. SEM was used to identify uranium-bearing mineral phases, textural relationships, and alteration rims.

Electron microprobe analysis (EPMA)

Quantitative compositional data of uranium minerals and Ti-bearing phases were obtained using a JEOL JXA-8100 EPMA with Inca Energy EDS. Analytical conditions included 15 kV accelerating voltage and 10 nA beam current, and spot diameter ≤ 2 μm. The counting time was 5 s per element. X-ray lines analyzed included: SiKα, AlKα, MgKα, KKα, NaKα, YLα, CaKα, PKα, FeKα, TiKα, ZrKα, PbMα, MnKα, UMα, ThMα, and CeLα. The following mineral standards were used for calibration: Jadeite (Si, Al, Na), Sanidine (K), Olivine (Mg, Fe), Plagioclase (Ca), Rutile (Ti), Zircon (Zr), Cubic zirconia (Y), Monazite (P, Ce), Uraninite (Pb, U, Th), Rhodonite (Mn). Electron microprobe analyses show that the total oxide contents range from 84.01% to 97.14%. Some analyses are of relatively poor quality, with total oxide contents below 85 wt%.

This suggests that some mineral phases may contain volatile components or undetected constituents (e.g., structural water or beam-sensitive phases), and that amorphous domains may also be present, which complicate quantitative analysis—for example, the auto-oxidation of U^{4+} to U^{6+} [40].

FIB-TEM sample preparation and transmission electron microscopy

Focused ion beam (FIB) sample preparation was conducted at the Guangzhou Institute of Geochemistry to prepare ~100 nm thick foil sections for TEM analysis. The Ti-U mineral assemblage of brannerite-like was characterized at nanometer resolution using a Thermo Scientific Talos F200S TEM (200 kV) equipped with EDS.

Except for FIB sample preparation and TEM-EDS analysis, all other experiments were conducted at the State Key Laboratory of Nuclear Resources and Environment, East China University of Technology.

4. Results

4.1 Petrographic features of the Toutunhe Formation sandstones

The detailed observation and description of the drill cores from the Toutunhe Formation (J_2t) in the study area, together with the field outcrop observation, show that the rock types developed in the Toutunhe Formation (J_2t) mainly include mudstone, fine, medium and coarse sandstone, gravel-bearing sandstone, glutenite and conglomerate. The mudstone is commonly interbedded with sandstone with rich variety of colors. Besides the common grayish green mudstone, brownish red and red-dominated varicolored ones also exist (Fig 7A). The structure type of the mudstone is dominated

by massive beddings (Figs 3A and B). The sandstone revealed by drilling is normally grayish white or gray (Fig 7B), and the sand yellow and dark purple sandstones occasionally occur on outcrops (Fig 3B). From the perspective of grain size, the medium-coarse sandstone is dominant, followed by the medium sandstone, fine sandstone and siltstone. ~~The~~ depositional structures such as flaser bedding (Fig 3C), parallel bedding (Fig 3A), horizontal bedding (Fig 3B), and small to medium (with the single-layer system < 10 cm) planar cross bedding (Figs 3C and 12B) are commonly developed in the sandstone. The gravel-bearing sandstone, glutenite and conglomerate revealed by drilling are grayish white (Fig 7B), containing a considerable amount of carbonaceous debris and pyrite (Figs 12J-L), with clayization developed (Figs 12K). The depositional structures such as large to giant (with the single-layer system > 10 cm) planar cross bedding (Fig 3D), massive bedding (Fig 3E), trough cross bedding (Fig 3F) and surface of scour are developed commonly in the outcrops. The components of the conglomerate mainly include silicite, granite and tuff with poor sorting, and generally being sub-angular to sub-round in roundness.

Fig. 3 Petrographic photographs of Toutunhe Formation (J_2t) outcrops in Louzhuangzi area: (A-C) Sandstone outcrop of the Upper Member, Toutunhe Formation (J_2t^2), showing fine-medium sandstone with parallel beddings (A), mudstone with massive beddings (B), fine-medium sandstone with horizontal beddings (B), and fine sandstone with flaser beddings and planar cross beddings (C); (D-F) Sandstone outcrop of the Lower Member, Toutunhe Formation (J_2t^1), showing sandstone with large to giant planar cross beddings (D), conglomerate with massive beddings (E), and glutenite and coarse sandstone with trough cross beddings (F); (G) Interface between the Lower Member of the

Toutunhe Formation (J_2t^1) and the Xishanyao Formation (J_2x); (H) Sandstone outcrop of the Xishanyao Formation (J_2x), showing siltstone with parallel beddings and coal lines.

Table 1. Property of sandstone samples from Toutunhe Formation (J_2t) of Louzhuangzi area, southern margin of Junggar Basin.

Sample number	Drill hole	Sampling depth / (m)	Lithology description
**23DJA-6**	ZK16-2	654.60	Grey glutenite
**23DJA-9**		705.70	
**22ZGE001**	ZK12-2	672.48	Grey gravel-bearing coarse sandstone
**23DJA-4**		680.70	
**23ZGE016**	ZK12-3	838.64	
**23ZGE028**		798.50	
**23DJA-15**		871.50	Grey glutenite
**23DJA-27**		641.70	
**22ZGE014**	ZK6-2	483.47	Grey gravel-bearing coarse sandstone
**22ZGE015**		479.30	
**22ZGE016**		477.30	
**22ZGE026**		398.80	
**22ZGE063**		478.60	
**23ZGE027**	ZK3-3	753.10	Grey white fine sandstone

**23ZGE026**	ZK4-2	398.20	Grey white coarse sandstone
**23DJA-7**	ZK4-3	710.40	Grey gravel-bearing
**22ZGE033**	ZK5-1	148.50	coarse sandstone
**22ZGE034**	ZK11-2	24.84	Grey coarse sandstone
**23ZGE022**	ZK11-3	119.52	Grey white gravel-bearing coarse sandstone
**23ZGE024**		79.20	

The components of the sandstone clasitcs in the Toutunhe Formation (J_2t), Louzhuangzi area have been identified by using a polarizing microscope, the statistical analysis of the area proportions of quartz, feldspar and debris under the field of view has been performed, and the percentage content of each type of clastics components has been determined. When the statistical data are projected into the Folk's ternary diagram for sandstone classification, it can be seen that the type of sandstone is mainly feldspar lithic sandstone, followed by lithic feldspar sandstone, and the feldspar sandstone is just rarely found (Fig 4). Studies on the components of the sandstone clastics show that the percentage contents of the debris and feldspar in the sandstone are relatively high. Specifically, quartz accounts for 18% to 59%, averaging 38%; feldspar accounts for 15% to 50%, averaging 26%; debris accounts for 10% to 60%, averaging 35%. The compositional maturity index Q/(F+R) of the sandstone ranges from 0.22 to 1.44, averaging 0.65, which is relatively low.

Fig. 4 Classification diagram of sandstones in Toutunhe Formation (J_2t), Louzhuangzi area

(According to [41]).

The content of sandstone clastics ranges from 70% to 89%, averaging 82%, with quartz, feldspar and debris as the main components. The sorting property of the clastic particles is mostly poor, and the roundness is mainly sub-angular to angular, with only a small amount of debris being sub-round to round (Figs 5 and 6). The clastic particles are relatively dispersed and generally do not contact with each other (Figs 5A-F), with the particle diameters being up to 2600 μm, mostly between 500 and 1500 μm, averaging about 800 μm. The structural type is mainly coarse-giant grained sandy structure, followed by fine-medium grained sandy structure. The interstitial materials between the clastiic particles are mainly composed of matrix and cements, with a content of approximately 20% to 30%. The matrix content is relatively high, with the components being mainly fine silty quartz and feldspar particles (Figs 5B, C, E), followed by a small amount of clay mineral, such as illite, sericite (Fig. 5B), and kaolinite, smectite, chlorite (Figs 10E, F and 15, 16). In the uranium mineralized sandstone samples, finely dispersed uranium minerals and fine-grained pyrite are commonly observed in the matrix, and associated with clay minerals and carbonaceous debris (Figs 5H, I and 10A-C). The cementing materials are mainly composed of carbonate and clay minerals (Figs 5A and 6F), followed by gelatinous pyrite (Figs 5G and I). The main cementing types are mostly basal cementation, with matrix-supported structures (Figs 5A-F). These clastic particles features indicate that both the compositional maturity and the structural maturity of the sandstone are generally low.

Fig. 5 Petrographic characteristics of sandstone samples from Toutunhe Formation (J_2t) in Louzhuangzi area under microscope: (A) Sharp-edged quartz clasts cemented by basal carbonate cementation, ZK5-1, 22ZGE033, 148.50 m, (+); (B) Clastics and clay matrix, ZK6-2, 22ZGE016, 477.30m, (+); (C) Pink crystalline quartz and feldspar matrix filled among debris particles, ZK6-2, 22ZGE016, 477.30m, (+); (D) Illitizated plagioclase with relatively dirty surface, ZK6-2, 22ZGE063, 478.60m, (+); (E) Lattice twin crystal microcline, ZK6-2, 22ZGE014, 483.47m, (+); (F) Bending and crushing of serimicized biotite, ZK12-2, 22ZGE001, 672.48m, (+); (G) Pyrite cementation, ZK4-2, 23ZGE026, 398.20m (reflected light); (H) Fine-grained pyrite and uranium minerals filled the cell cavities of carbonaceous debris, ZK4-2, 23ZGE026, 398.20 m, (BSE image); (I) Pyrite and uranium minerals filled among the clastic particles, ZK12-3, 23ZGE028, 798.50 m, (BSE image).

The relative content of quartz ranges from 22% to 59%, averaging 40%, with single crystal quartz being the main component, accounting for about 65% of the total amount of quartz, followed by polycrystalline quartz, accounting for about 35%. The quartz is generally characterized by dirty and rough surface, with poor roundness, and commonly shows angular and sub-angular shapes (Figs 5A-D and 6C, D). The feldspar content ranges from 11% to 36%, averaging 21%, with microcline and plagioclase being the main components. The microcline is mainly the characterized by lattice twin crystal structures, with the twin crystal bands usually having a spindle-shaped lattice pattern under the microscope. (Fig 5E). The plagioclase mainly shows the characteristics of polysynthetic twin texture, with serimicization usually occurring on

the surface (Fig 5D), and compaction and crushing of altered biotite occasionally being observed (Fig 5F).

The debris particles have relatively large diameters and good roundness being sub-round and round, with cement and detrital matrix usually observed on the edge. The debris can be classified into the following 3 types according to the rock property in the source area: igneous rock debris, sedimentary rock debris and metamorphic rock debris. The commonly observed tuff debris (Fig 6A), dacite porphyry debris (Fig 6D), and granite debris (Figs 6E, F) are all the products of fragmentation and deposition of the intermediate acidic igneous rocks and intrusive rocks. The sedimentary rock debris is composed mainly by siliceous rock debris (Fig 6C), while flint debris (Fig 6E) and quartz schist debris (Fig 6B) constitute the main types of the metamorphic rock debris. The rhyolite spherulitic texture, almond shaped body, is clearly visible in the igneous rock debris (Fig 6A), indicating that the weathering of the pro-rock is relatively intense. Porphyritic texture is identified in part of the acidic volcanic rock debris (Fig 6D), with felsic mineral as the main composition of the phenocryst and cryptocrystalline quartz as the matrix. In some thin slices of rock samples, the epigranite debris can be found, the granitic texture composed of quartz and feldspar is clearly visible, whereas most of the mica-type minerals are absent (Figs 6E, F). The metamorphic rock is mainly composed of the quartzite debris that accounts for 90% of the metamorphic rock debris, mainly in the forms of polycrystalline quartz debris (Fig 6C) and flint debris (Fig 6E), followed by the quartz schist debris (Fig 6B), with obvious platy structures.

Fig. 6 Debris characteristics of sandstone samples from Toutunhe Formation (J_2t) in Louzhuangzi area under microscope: (A) Cryptocrystalline tuff debris, ZK6-2, 22ZGE016, 477.30 m, (+); (B) Platy-structure quartz schist debris, ZK6-2, 22ZGE015, 479.30m, (+); (C) Sericitization of sharp edged polycrystalline quartz, ZK6-2, 22ZGE016, 477.30m, (+); (D) Dacite porphyry debris with porphyritic texture, ZK6-2, 22ZGE026, 398.80m, (+); (E) Dissolution pit visible in cryptocrystalline flint debris, ZK6-2, 22ZGE026, 398.80m, (+); (F) Carbonate-cemented granite debris, ZK11-2, 22ZGE034, 24.84 m, (+).

4.2 Sandstone depositional characteristics

The Toutunhe Formation (J_2t) can be divided vertically into upper and lower members. The Upper Member (J_2t^2) consists of relatively thick purple-red to dark purple mudstone interbedded with thin grayish-white siltstone and fine sandstone layers (Fig 7A). Depositional microfacies including floodplain, natural levee, point bar, and channel-fill deposits are recognized. Sedimentary structures observed in the Upper Member include massive bedding in mudstone (Fig 3B), flaser bedding in fine sandstone (Fig 3C), horizontal bedding in mudstone and sandstone (Fig 3B), parallel bedding in sandstone (Fig 3A), and small- to medium-scale planar cross-bedding (Figs 3C and 12B).

Fig. 7 Photographs of representative core samples from Toutunhe Formation (J_2t) exposed by the economic uranium mineralization drill hole of ZK5-7 in Louzhuangzi area: (A) Meandering river facies ' thick mud with thin sand from the Upper Member of the Toutunhe Formation (J_2t^2); (B) Gravelly braided river facies thick sand body from the Lower Member of the

Toutunhe Formation (J_2t^1); (C) Sandy braided river facies flood mud and crevasse splay sand from the bottom of the Lower Member of the Toutunhe Formation (J_2t^1) .

The ore-bearing main sandstones in the Lower Member of the Toutunhe Formation (J_2t^1) is composed of the thick, grayish-white coarse sandstone, glutenite, and conglomerate interspersed by the thin layers of grayish-green mudstone on the profile (Fig 7B), with the depositional microfacies such as sandy channel fill, lag deposit and gravel bar developed (Fig 8). The Glutenite with trough cross-bedding (Fig 3F) and conglomerate with massive bedding (Fig 3E) also occur. At the base of the Lower Member (J_2t^1), gray to grayish-white coarse sandstone and medium–fine sandstone interbedded with green mudstone are observed (Fig 7C). Depositional microfacies including floodplain deposits, crevasse splays, and channel bars are identified (Fig 8), and sedimentary structures such as massive bedding, small-scale planar cross-bedding, climbing ripple bedding, and scour surfaces are common (Fig 8).

Up-section from the Lower to the Upper Member (J_2t^1), thick conglomerate and coarse sandstone layers decrease, whereas grayish-white siltstone and fine sandstone layers become more abundant. In addition, multiple gypsum layers are present within the succession (Figs 12E, I).

Fig. 8 Interpretation of the core profile of drill hole ZK5-1 in Louzhuangzi area. (Abbreviation: Fm=Formation; DF= Depositional Facies; OD= Overbank Deposit; SCF= Sandy Channel Fill; LD= Lag Deposit; GB= Gravel Bar; FP= Floodplain; CS= Crevasse Splay; CB= Channel Bar; IDB= Interdistributary Bay; SDC= Subaqueous Distributary Channel).

4.3 Uranium mineralization characteristics

The uranium mineralization in the Louzhuangzi area occurs in the sandstones of the Lower Member of the Toutunhe Formation (J_2t^1). The classification of depositional facies based on outcrops and borehole strata, together with previous studies, shows that the Lower Member of the Toutunhe Formation (J_2t^1) contains sandstones deposited in braided-river delta plain, sandy braided-river, and gravelly braided-river facies (Figs 7-9). The uranium mineralization zone, large in scale, is distributed along the direction of the sandstone trend from east to west, being about 5.5 km long from east to west and about 800 m – 1,100 m in width (Fig 1C), controlled mainly by the depositional sandstones of braided river delta plain deposition (the main channel of gravelly braided river) (Fig 14). The A-A 'borehole profile (Fig 9) exposes multiple layers of uranium bodies and uranium mineralized bodies in the depths ranging from 32.05 m to 695.55 m, with the depth being deeper in the west and shallower in the east (approaching the eroded surface). The thickness of the economic ore bodies is generally between 0.8 m and 5.6 m, controlled by a set of gravelly braided river depositional sandstones, which are thick and large, with coarse sandstone, glutenite, and conglomerate being interbedded frequently, and are loose to relatively loose in structure, with good permeability, connectivity and stratification, and rich in carbonized plant detritus. The gravelly braided river channel lag deposits is the main position for the formation of uranium mineralization (Fig 8). The thick mudstone layer deposited in the meandering river environment in the Upper Member of the Toutunhe Formation (J_2t^2) and the mud-bearing sandstone at the bottom of the Lower Member (J_2t^1) act as the top and bottom

plates of the aquiclude, respectively, and form a sandstone structure favorable for uranium mineralization together with the thick gravel-bearing coarse sandstone, glutenite, and conglomerate layers in the middle and upper sections of the Lower Member (J_2t^1) (Fig 9).

Fig. 9 Sketch diagram showing the A-A' profile in Louzhuangzi area (In the Lower Member of the Toutunhe Formation (J_2t^1), the top roof of the sandstone has been leveled. The profile position is shown in Figure 1C).

4.4 Uranium occurrence and mineral assemblages

In the uranium ore samples from the Toutunhe Formation (J_2t) of the Louzhuangzi area, uranium occurs mainly in three types of state including the independent uranium mineral, unclassified nano-uranium minerals and the isomorphism substitution mineral.

4.4.1 Independent uranium mineral

The independent uranium minerals mainly include coffinite and pitchblende, with a small amount of brannerite-like.

Electron probe analyses indicate that the diameters of the coffinite particles generally range from 5 μm to 30 μm, with these particles mostly being distributed among the clastic particles in gel-like forms, and coexisting with gelatinous pyrite (Fig 10A); the finely dispersed coffinite particles associated with chlorite are commonly observed (Figs 10F and 16), and the coffinite particles are also commonly observed occurring in ring-shaped distribution on the quartz surface and coexist with pitchblende (Figs 10D, I); massive coffinite particles (with particle diameter up to 200 μm) can

occasionally be observed in the matrix (Fig 10B). The coffinite is generally rich in Y_2O_3, CaO, P_2O_5 and FeO, with the total amount of these components accounting for 87.51% to 97.14%, averaging 91.81%. Specifically, the contents of SiO_2, UO_2, Y_2O_3, CaO, P_2O_5, FeO range from 9.33% to 18.23%, 52.89% to 66.88%, 0.50% to 7.33%, 0.62% to 7.21%, 1.06% to 6.44%, and 0.09% to 4.59%, respectively, with the average contents of 14.31%, 61.33%., 3.40%., 3.42%, 3.43%, and 1.55%, respectively. Additionally, the coffinite also contains a small amount of impurities such as TiO_2, ZrO_2, PbO, and MnO (Table 2).

Fig. 10 Occurrence characteristics of uranium in sandstones of Toutunhe Formation (J_2t) in Louzhuangzi area (BSE images), including (A) Gelatinous coffinite associated with pyrite, ZK11-3, 23ZGE022, 119.52m; (B) Massive coffinite associated with fine-grained pyrite, ZK12-3, 23ZGE028, 798.50m; (C) Massive pitchblende associated with pyrite, ZK12-3, 23ZGE028, 798.50 m; (D) Baited pitchblende coexisted with coffinite with ring-shaped distribution; ZK4-2, 23ZGE026, 398.20 m; (E) Nanoscale titanium-uranium aggregates associated with chlorite, ZK3-3, 23ZGE027, 753.10m; (F) Nanoscale uranium particles adsorbed in clay minerals, ZK12-3, 23ZGE028, 798.50m; (G) Nanoscale uranium minerals adsorbed at the edge of titanium oxides, ZK4-2, 23ZGe026, 398.20m; (H) Nanoscale uranium minerals adsorbed in the dissolution pores of titanium oxides, ZK12-3, 23ZGE028, 798.50 m; (I) Uranium-rich monazite associated with baited pitchblende, ZK4-2, 23ZGE026, 398.20m; (J) Uranium-rich rutile, ZK4-2, 23ZGE026, 398.20 m; (K-L) Area distribution of U(K) and Ti (L) elements in uranium-rich rutile obtained by electron probe.

Pitchblende particles are relatively fine, generally ranging from 5 μm to 20 μm in diameters, and its content in the independent uranium minerals is lower than that of

coffinite. The composition and appearance characteristics of pitchblende can basically be determined by electron microscope. Under the electron microscope, the massive pitchblende particles can be observed to appear in the matrix among the clastic particles, often associated with clay minerals and fine-grained pyrite (Fig 10C), and the baited pitchblende can also be commonly observed to be wrapped by the in ring-shaped distribution of coffinite in the quartz dissolution pits (Figs 10D, I). The pitchblende is characterized by a lower SiO_2 content and a higher UO_2 content. The total amount of the pitchblende chemical components accounts for 86.29% to 95.38%, with an average of 90.63%. In which, SiO_2 content ranges from 1.05% to 5.76%, averaging 2.36%. and UO_2 content ranges from 75.50% to 86.07%, averaging 81.06%. Additionally, the pitchblende also contains a small amount of impurities such as Y_2O_3, CaO, P_2O_5, and FeO (Table 2).

Fig. 11 Images showing the characteristics of titanium-containing uranium minerals under FIB and TEM, including (A) Backscattered electron (BSE) image of titanium-containing uranium mineral under focused ion beam (FIB) scanning electron microscope; (B) Image of the transparent foil of titanium-containing uranium mineral under transmission electron microscope (The transparent foil was taken vertically downward at the yellow rectangular position in Figure A, with the thickness <100 nm); (C) HAADF-STEM image of titanium-containing uranium mineral; (D-I) STEM-EDS-derived area distribution of Ti-U(D), U(E), Ti(F), Ca(G) and Si(H) elements and energy spectra (I) of the black rectangular area in Figure C.

Besides coffinite and pitchblende, titanium-containing uranium minerals are also commonly found in the independent uranium minerals. This type of uranium mineral

usually fills among the clastic particles in the form of disseminated mineral aggregates (Fig 11A), or associated with chlorite (Fig 10E). Under the transmission electron microscope, it can be observed that the titanium-containing uranium mineral particles present a bright-white, complexly interwoven acicular aggregate morphology (Figs 11B, C). The micro-zone element area distribution image shows that this type of uranium-containing mineral is rich in Ti and U, with the distribution of the two elements being relatively uniform (Figs 11D-F). When combined with the micro-zone area-scanning element energy spectrum image (Fig 11I), it can be confirmed that this type of uranium mineral is a collection of nano-scale brannerite-like aggregate, rather than nanoscale coffinite or pitchblende associated with titanium oxides. The difference between the contents of TiO_2 and UO_2 in the brannerite-like is small and tends to be stable, with the average contents of TiO_2 and UO_2 being 33.20% and 39.62%, respectively. The contents of SiO_2, CaO and ZrO_2 in the brannerite-like are relatively high, with the average contents of SiO_2, CaO, and ZrO_2 being 6.48%, 1.74%, 2.88%, respectively. Additionally, a small amount of impurities such as Al_2O_3 and FeO also exist in the brannerite-like (Table 2).

4.4.2 Unclassified nano-uranium minerals

Under scanning electron microscope, the nano-scale uranium minerals adsorbed in chlorite are commonly observed (Figs 10F and 16), and the nano-scale uranium mineral are also commonly observed associated with loose and porous titanium oxides (Figs 10G, H). The uranium minerals are adsorbed at the edges or in the dissolution pores of this type of titanium oxides, and eventually formed a mixed mineral phase

containing uranium and titanium, and occurring in the sandstones in the form of titanium-uranium "mixture". This titanium-uranium "mixture" phase mineral is rich in the sandstone-type uranium deposit in the southern Songliao Basin, and can be observed under a transmission electron microscope. It is the nanoscale uranium mineral (coffinite, pitchblende) adsorbed at the edges or in the dissolution pores of the titanium oxides, rather than the brannerite-like described in previous studies. The nano-scale uranium minerals is characterized by relatively high content of TiO_2, with a large difference in UO_2 contents. The content of UO_2 adsorbed in the pores or at the edges of the titanium oxides is relatively low, whereas the content of UO_2 adsorbed in chlorite is relatively high. The total chemical component amount of the nano-scale uranium minerals ranges from 92.05% to 95.65%, with an average of 93.88%. Specifically, the contents of UO_2, TiO_2, FeO, SiO_2 range from 9.15% to 60.68%, 19.05% to 67.00%, 0.13% to 4.21% and 2.25% to 11.57%, respectively, averaging 31.98%, 45.82%, 2.33% and 7.41%, respectively; the average contents of CaO, Al_2O_3 and ZrO_2 are 1.80%, 1.67% and 1.13%, respectively. Additionally, the nano-scale uranium minerals also contain a small amount of impurities such as Y_2O_3 and P_2O_5 (Table 2).

4.4.3 Isomorphism substitution mineral

The minerals such as uranium-rich rutile (Fig 10J) and monazite (Fig 10I) can be occasionally observed under the microscope in the uranium ore samples from the Louzhuangzi area. The distribution of uranium in these minerals is extremely uneven (Figs 10K, L). These uranium-containing minerals may have been formed through the isomorphism substitution of Ti^{4+} and REE^{3+} by uranium in the source rocks, and then

occurred in the sandstones as a result of source rock weathering and crushing, and sediment transportation and deposition. During the uranium mineralization process, uranium-rich rutile and monazite, due to the later-stage fluid alteration effect, can serve as potential uranium sources in depositional sandstone units, and can promote the formation of uranium mineralization.

Table 2. Analysis results of chemical components in uranium-rich minerals in Louzhuangzi area by electron probe (Wt %).

Test point	SiO_2	Al_2O_3	MgO	Na_2O	Y_2O_3	CaO	P_2O_5	FeO	TiO_2	ZrO_2	PbO	MnO	UO_2	Total	Mineral type
23ZGE022-01	18.23	0.22	0.03	0.11	0.50	3.88	1.26	0.09	0.12	0.09	0.05	0.02	66.44	91.03	coffinite
23ZGE022-02	9.33	0.99	0.19	0.11	0.64	4.30	2.12	0.46	2.86	4.43	0.11	0.15	66.88	92.57	coffinite
23ZGE022-03	16.39	1.24	0.19	0.01	0.92	4.88	2.79	0.62	0.28	7.24	0.05	0.16	61.58	96.36	coffinite
23ZGE022-04	11.54	0.06	0.05	0.07	0.60	4.42	3.51	1.47	0.58	3.13	0.05	0.07	66.71	92.26	coffinite
23ZGE028-01	15.98	1.59	0.14	0.03	0.97	4.49	2.60	0.20	0.30	7.17	0.01	0.11	58.27	91.85	coffinite
23ZGE028-02	13.27	1.08	0.13	0.05	0.72	4.26	2.65	0.30	1.73	6.75	0.05	0.01	59.24	90.23	coffinite
23ZGE028-03	14.33	1.21	0.05	0.21	4.93	7.21	5.91	0.21	0.07	0.98	0.00	0.12	54.04	89.27	coffinite
23ZGE028-04	15.09	0.05	0.49	0.03	7.17	0.62	1.31	1.06	0.00	1.16	0.09	0.00	60.54	87.61	coffinite
23ZGE026-01	14.28	0.05	0.39	0.08	6.21	0.65	1.06	2.66	0.00	1.28	0.06	0.02	60.99	87.72	coffinite
23ZGE026-02	16.20	0.28	1.87	0.07	7.33	0.83	1.24	1.00	0.00	0.49	0.18	0.00	58.07	87.56	coffinite
23ZGE026-01	16.54	5.38	0.19	0.17	4.69	3.88	5.53	0.89	0.10	2.22	0.01	0.00	52.89	92.50	coffinite
23ZGE026-03	14.26	0.24	0.09	0.03	3.24	3.39	5.30	4.59	0.21	0.08	0.08	0.00	65.62	97.14	coffinite
23ZGE026-04	11.71	0.20	0.02	0.07	3.20	3.76	4.68	4.56	0.08	0.17	0.09	0.00	65.70	94.23	coffinite
23ZGE026-05	12.42	0.19	0.07	0.01	3.96	2.67	5.25	3.51	0.07	0.05	0.06	0.06	65.86	94.16	coffinite

23ZGE026-06	16.46	1.68	0.21	0.08	4.36	4.21	6.44	1.84	0.48	0.82	0.00	0.06	60.37	97.01	coffinite
23ZGE026-07	13.00	0.39	0.06	0.02	5.03	1.27	3.21	1.33	3.49	1.39	0.20	0.02	58.12	87.51	coffinite
$\bar{X}$（averages）	14.31	0.93	0.26	0.07	3.40	3.42	3.43	1.55	0.65	2.34	0.07	0.05	61.33	91.81	
23ZGE028-05	4.34	0.02	0.03	0.19	0.26	3.45	0.36	0.33	1.88	0.69	0.06	0.36	82.87	94.83	pitchblende
23ZGE028-06	1.20	0.00	0.04	0.10	0.00	3.56	0.30	0.32	0.35	0.31	5.21	0.39	83.60	95.38	pitchblende
23ZGE028-07	5.76	1.72	0.24	0.27	0.00	2.79	0.17	0.92	0.64	1.16	0.02	0.47	75.50	89.66	pitchblende
23ZGE028-08	1.95	0.02	0.09	0.28	1.52	2.39	0.05	0.62	0.00	0.03	0.05	0.54	83.33	90.85	pitchblende
23ZGE028-09	2.75	0.00	0.07	0.55	1.81	1.21	0.30	0.45	0.00	0.00	0.00	0.19	84.25	91.58	pitchblende
23ZGE026-08	2.40	0.03	0.06	0.49	1.30	1.74	0.14	0.71	0.00	0.16	0.05	0.38	84.67	92.12	pitchblende
23ZGE026-09	1.73	0.01	0.05	0.26	1.18	1.86	0.08	0.60	0.00	0.05	0.01	0.46	86.07	92.33	pitchblende
23ZGE026-10	1.05	0.00	0.00	0.06	0.36	3.99	0.25	1.18	0.25	0.63	1.26	0.73	76.82	86.57	pitchblende
23ZGE026-11	1.11	0.00	0.00	0.09	0.36	3.55	0.24	1.04	0.57	0.56	1.47	0.67	77.06	86.72	pitchblende
23ZGE026-12	1.30	0.01	0.01	0.07	0.33	3.07	0.22	0.94	0.94	0.79	1.28	0.90	76.44	86.29	pitchblende
$\bar{X}$（averages）	2.36	0.18	0.06	0.24	0.71	2.76	0.21	0.71	0.46	0.44	0.94	0.51	81.06	90.63	
23ZGE027-01	6.30	0.83	0.16	0.09	0.02	2.19	0.16	0.41	33.49	2.23	0.01	0.32	39.81	86.01	Brannerite-like
23ZGE027-02	9.18	2.30	0.51	0.08	0.09	2.92	0.16	0.70	31.97	3.37	0.06	0.55	38.01	89.88	Brannerite-like

22ZGE027-03	6.88	0.83	0.06	0.30	0.03	1.07	0.17	2.05	31.67	2.64	0.00	0.24	40.40	86.33	Brannerite-like
22ZGE027-04	5.74	0.36	1.62	0.06	0.43	0.59	0.12	0.45	33.34	1.75	0.03	0.19	39.33	84.01	Brannerite-like
23ZGE026-13	6.62	1.06	0.23	0.06	0.07	3.03	0.16	0.54	32.67	3.43	0.00	0.63	38.72	87.22	Brannerite-like
23ZGE026-14	4.14	0.74	0.09	0.34	0.12	0.65	0.12	3.99	36.09	3.86	0.00	0.24	41.46	91.83	Brannerite-like
$\bar{X}$（averages）	6.48	1.02	0.45	0.16	0.13	1.74	0.15	1.36	33.21	2.88	0.02	0.36	39.62	87.55	
23ZGE026-15	8.69	2.37	0.34	0.22	0.15	1.43	0.07	4.21	67.00	0.91	0.05	0.22	9.15	94.81	
23ZGE026-16	4.25	1.07	0.31	0.20	0.00	2.86	0.27	2.43	21.24	2.10	0.00	0.23	60.68	95.65	
23ZGE026-17	10.30	0.11	0.02	0.07	2.43	3.60	2.07	2.45	19.05	0.10	0.02	0.10	53.24	93.55	Unidentified nano-uranium minerals
23ZGE028-10	2.25	0.00	0.38	0.04	0.17	0.63	0.10	0.13	60.33	1.60	0.00	0.12	26.29	92.05	
23ZGE028-11	11.57	4.80	0.82	0.14	0.01	0.46	0.03	2.42	61.47	0.93	0.06	0.09	10.55	93.34	
$\bar{X}$（averages）	7.41	1.67	0.37	0.13	0.55	1.80	0.51	2.33	45.82	1.13	0.03	0.15	31.98	93.88	

4.5 Alteration features

4.5.1 Interlayer oxidation

In the Wanjiayao mineral site, located northeast of the Louzhuangzi mineral area, brownish-yellow discoloration is clearly observed on outcrops within sandstones of the Upper Member of the Toutunhe Formation (J_2t^2) (Figs 12A, B). In addition, relatively large fragments of carbonaceous debris are occasionally present and show no obvious color change relative to the surrounding matrix (Fig 12C). Uranium anomalies are detected within brownish-yellow glutenite (Fig 12D), and gypsum layers beneath the glutenite locally display partial discoloration and textural alteration (Fig 12E). At the Hasa Tomb anomaly site, east of the Louzhuangzi mineral area, the sandstone of the Lower Member of the Toutunhe Formation (J_2t^1) exhibits heterogeneous brownish-yellow staining and mottled coloration (Fig 12F). Some stained surfaces are partially covered by Quaternary surficial deposits. After removal of the surficial cover, brownish-yellow staining remains clearly visible and is spatially associated with zones of elevated uranium mineralization (Fig 12G). Also, in the east of the Louzhuangzi mineral area, an interlayer oxidation zone has been exposed in the Upper Member of the Toutunhe Formation (J_2t^2) by the economic uranium mineralization drill hole of ZK11-3, with the buried depth being about 40.9m, and multiple layers of gypsum being present in the oxidized sandstone (Fig 12I). Except for the sandstone exposed by the drill holes of ZK11-2 and ZK11-3, the sandstones exposed by the other holes drilled to the Toutunhe Formation (J_2t) are all grayish to grayish-white (Figs 7B, 8 and 9) and reducing sandstones, rich in carbonized plant detritus and pyrite (Figs 12J-L), but the

residual traces of ilmenite oxidation and alteration can still be observed under the microscope (Figs 17A, B).

Fig. 12 Epigenetic alteration characteristics of sandstones in Toutunhe Formation (J_2t), Louzhuangzi area, including (A-D) Sand-yellow oxidized sandstone outcrop (A); medium to large planar cross bedding developed in the sandstone (B); incompletely oxidized carbonized plant detritus (C) and layered gypsum (E) occasionally observed; uranium anomaly commonly observed in gravel-bearing coarse sandstone (D) of the Upper Member of the Toutunhe Formation (J_2t^2) at Wanjiayao mineralization site; (F-G) Incompletely oxidized gray residues in the sand-yellow oxidized sandstone (F); uranium anomalies observed in the oxidized sandstone (G) of the Lower Member of the Toutunhe Formation (J_2t^1) at Hasa tomb anomaly site; (H-I) Oxidized sandstone (H) and multiple layers of gypsum (I) of the Upper Member of the Toutunhe Formation (J_2t^2) exposed by drill hole ZK11-3; (J) Laminated carbonized plant detritus and pyrite distribution observed in the grayish-white altered sandstones in the Lower Member of the Toutunhe Formation (J_2t^1) exposed by drill hole ZK 6-4; (K) Kaolin and pyrite nodules developed in the grayish-white altered sandstones in the Lower Member of the Toutunhe Formation (J_2t^1) exposed by drill hole ZK 12-3; (L) Laminated carbonized plant detritus and pyrite distribution exposed by drill hole ZK4-3 in depth of 758.24m.

Field geological investigation found that the interlayer oxidation zone in the Louzhuangzi mineral area extends in a belt-like pattern from the Hasa tomb anomaly site in the east of this mineral area continuously to the Wanjiayao, Liuhuang'gou and Qianshuihe mineralization sites, with the total length of the front line of the oxidation zone controlled by outcrops and drill holes being approximately 20km (Fig 14). The

drill hole ZK11-2 reveals that the uranium mineralized sandstone in the Lower Member of the Toutunhe Formation (J_2t^1) is closely related to the oxidized sandstone at the Hasa tomb anomaly site, with every ore layer, every uranium mineralized body, and every uranium anomaly exposed by this drill hole in the Lower Member of the Toutunhe Formation (J_2t^1) having the corresponding interlayer oxidation zone at the Hasa tomb anomaly site in east of this drill hole (Fig 13). The field geological investigation and the analysis of the information revealed by the drill hole show that the uranium mineralization in the Louzhuangzi area is closely related to the interlayer oxidation zone, and specifically, it is mainly controlled by the interlayer oxidation of epigenetic oxygen- and uranium- containing fluids. The development of the interlayer oxidation zone is mainly controlled by the Haojiagou Uplift, with the front line of the oxidation zone being basically parallel to the Haojiagou Uplift zone (Fig 14).

Fig. 13 Profile of interlayer oxidation zones and uranium mineralization zones in sandstones of Toutunhe Formation (J_2t) in drill hole ZK 11-2 at Hasa tomb anomaly site (with sandstone top roof being leveled).

Fig. 14 Distribution of depositional facies and oxidation zones in the Lower Member of Toutunhe Formation (J_2t^1), Louzhuangzi area (modified from [13]).

4.5.2 Clayification

The gray and grayish-white sandstone alteration zone in the Lower Member of the Toutunhe Formation (J_2t^1) of the Louzhuangzi area (Figs 7B, 8, and 9) and the uranium mineralization zone are closely related in space. The ore-bearing sandstones are mostly glutenite in lithology, and has undergone relatively strong clayification (Figs 8, 15 and

16). Large amounts of clay minerals have been identified in the sandstones occurring in the gray and grayish-white alteration zone by using a scanning electron microscope, with the lithologies being mainly kaolinite (Figs 15A-C), smectite (Figs 15D-F), and chlorite (Figs 15G-I), followed by sericite (Figs 5B and 6C). Clay minerals have excellent adsorption capability and high surface activity, and can selectively adsorb the oxygen- and uranium- containing fluids migrating in the sandstone pores, leading to the enrichment and precipitation of uranium elements on the surface of clay minerals, and eventually forming uranium-rich flake-like and massive clay aggregates (Fig 16).

Fig. 15 Characteristics of clay minerals in sandstones in Lower Member of Toutunhe Formation (J_2t^1), Louzhuangzi area (BSE images), including (A) Intergranular layered kaolinite, ZK16-2, 23DJA-9, 705.70m; (B) Intergranular worm-like kaolinite, ZK16-2, 23 dJA-6, 654.60m; (C) Intergranular plate-like kaolinite, ZK12-3, 23 dJA-27, 641.70m; (D) Spherical smectite on the particle surface , ZK4-3, 23DJA-7, 710.40m; (E) Cellular smectite on the particle surface , ZK12-3, 23 DJA-15,871.50m; (F) The strawberry pyrite Encapsulated by flake-like smectite, ZK12-2, 23DJA-4, 680.70m; (G-I) Intergranular flake-like chlorite aggregate, ZK16-2, 23DJA-9, 705.70m.

Fig. 16 Association of uranium minerals and clay minerals in sandstones in Lower Member of Toutunhe Formation (J_2t^1), Louzhuangzi area (BSE images), including (A) Uranium mineral adsorbed in chlorite, ZK3-3, 23ZGe027, 753.10m; (B) Uranium mineral associated with chloritized biotite, ZK4-2, 23ZGE026, 398.20m; (C) Uranium minerals associated with chlorite, ZK12-3, 23ZGE028, 798.50m.

4.5.3 Sulfurization

Titanium oxide particles are commonly observed in thin sections of the gray and

grayish-white sandstones of the Lower Member of the Toutunhe Formation (J_2t^1). These particles display clear features of epigenetic alteration and are predominantly angular to sub-angular in morphology (Figs 17A, B). A smaller proportion exhibits circular to sub-circular shapes (Figs 17C, D), suggesting modification by epigenetic processes and indicating that these titanium oxides represent altered products of proximal deposits. The early deposited ilmenite oxides were eventually transformed to the combination assemblage of anatase and magnetite due to the iron exsolution in the epigenetic alteration process, with pyrite enveloped at the edge of the mineral assemblage (Figs 17A, B). The uranium minerals adsorbed at the edge of altered titanium oxides were eventually transformed to the uranium-bearing phases rich in iron, titanium, and sulfur, with the periphery of these uranium-bearing phases commonly enveloped by gelatinous pyrite (Figs 17C, D). Under the effect of strong sulfurization, the ilmenite was metasomatized by pyrite, and formed the combination assemblage of anatase and pyrite, but the lattice twin structure of ilmenite still remains (Figs 17E, F).

Fig. 17 Alteration characteristics of titanium oxides in sandstones in Lower Member of Toutunhe Formation (J_2t^1), Louzhuangzi area. (A-C) Anatase and magnetite mineral assemblage enveloped by gelatinous pyrite (reflected light); (D) uranium-bearing oxides enveloped by gelatinous pyrite; (E-F) Interwoven association of anatase and pyrite (BSE image).

In addition, the near surface oxidized sandstones are only exposed in the eastern of the Louzhuangzi mineral area by drill holes ZK11-2 and ZK11-3 along the trend of the uranium ore body. Within a range of 6km to the west of the Louzhuangzi mineral area, the target sandstones exposed by drill holes are all gray or grayish white (Figs 7B,

8, 9 and 18). The rock slice identification under the microscope show that the ore-bearing sandstones have undergone intense sulfurization (Fig 17). Moreover, the petroleum geological information indicates that the oil and gas activities in the deep layers of the study area have been frequent, suggesting that the intense sulfurization alteration in the gray and grayish-white sandstones were highly likely formed by the reduction and bleaching effects due to the large amount of H_2S and CH_4 gases released from the deep oil and gas leakage, which eventually determined the spatial distribution of uranium mineralization [15].

Fig. 18 Schematic diagram of the B-B ' drill hole profile in Louzhuangzi area. The profile position is shown in Figure 1C (modified from [15-16]).

5. Discussion

5.1 Depositional environment and facies interpretation

In the Upper Member (J_2t^2), thick mudstone intervals with massive bedding (Fig 3B) are consistent with deposition under low-energy conditions such as floodplains or abandoned channels, where sediment settling dominates under weak flow conditions [42]. Flaser bedding in fine sandstone (Fig 3C) suggests intermittent traction and suspension deposition under fluctuating flow, consistent with sandy sediment migration on channel bottoms [9, 43]. Horizontal bedding in mudstone and sandstone (Fig 3B) is typical of low-energy sheet-like sedimentation in floodplain or onlap settings where flow is weak and deposition is laterally extensive [44]. Parallel bedding in sandstone (Fig 3A) indicates deposition under a higher-flow regime with planar laminar flow conditions [45]. Small- to medium-scale planar cross-bedding (Figs 3C and 12B)

reflects bedform migration and lateral sediment transport under persistent flow, compatible with a meandering-river setting characterized by relatively moderate hydrodynamic energy [46].

In the Lower Member (J_2t^1), the dominance of thick coarse sandstone, glutenite, and conglomerate (Fig 7B), together with the development of channel-fill, lag-deposit, and gravel-bar microfacies (Fig 8), indicates deposition in a braided-river system with higher-energy channel processes. Large-scale planar cross-bedding in glutenite (Fig 3D) is consistent with downstream-aggrading braided flow and rapid sediment accumulation [47]. Trough cross-bedding in glutenite (Fig 3F) and massive-bedded conglomerate (Fig 3E) further suggest channel erosion and subsequent infill, typical of gravelly braided fluvial deposition under strong hydrodynamic conditions [48]. The sandy-dominated association at the base of the Lower Member, with climbing ripples and scour surfaces (Fig 8), is consistent with a sandy braided-river environment with moderate hydrodynamic energy.

The upward decrease in conglomerate and coarse sandstone and the increase in siltstone and fine sandstone from the Lower to the Upper Member [9], together with the appearance of multiple gypsum layers (Figs 12E, I), suggest an overall upward shift toward more arid conditions and reduced hydrodynamic energy, accompanied by progressive shallowing of the water body through time [49].

5.2 Sandstone alteration conditions and the response to uranium mineralization

Uranium mineralization in the Toutunhe Formation (J_2t) of the Louzhuangzi area

is primarily controlled by braided channel sedimentary architecture and subsequent fluid-induced alteration. Matrix-rich braided channel lag deposits constitute the principal ore- bearing facies. These facies-controlled sandstones are characterized by high primary permeability and enhanced geochemical reactivity along grain–matrix interfaces, thereby providing favorable pathways for the migration of uranium-bearing oxidizing fluids and the development of localized reduction fronts [5, 50].

The ore-bearing sandstones exhibit relatively low compositional maturity and contain abundant unstable or labile components, including feldspar and lithic fragments, which are highly susceptible to chemical weathering and diagenetic alteration [51]. Although burial compaction reduced the initial porosity, reactive detrital grain boundaries were largely preserved, particularly in zones where feldspar and lithic fragments underwent alteration. Labile components—especially feldspar and volcanic or metamorphic lithic fragments—are prone to alteration under oxidizing or acidic conditions, releasing Ca, Na, and K, and potentially providing ion-exchange surfaces capable of temporarily adsorbing or immobilizing uranium during fluid migration [52]. Moreover, alteration of feldspar and lithic fragments may generate secondary porosity and authigenic clay minerals (e.g., kaolinite, montmorillonite, and chlorite), which can serve as adsorption sites for uranium or participate in redox buffering processes [53].

5.3 Reduction–precipitation of uranium and superimposed transformation

A close spatial association is observed between uranium minerals and pyrite, titanium oxides, and organic matter within the ore- bearing sandstones. Pyrite and

organic matter act as effective reducing agents, forming localized redox-buffering zones capable of reducing mobile U^{6+} to immobile U^{4+} [16, 54-56]. Titanium oxides exhibit strong surface complexation capacity for U^{4+}. Microstructural and compositional evidence supports their dual role as both adsorption substrates and mineralization nuclei [57], and they may further promote recrystallization under fluctuating redox conditions and temperature variations [31, 58]. The recrystallized Ti–U mineral aggregates are interpreted as nanocrystalline brannerite-like phases. Although further confirmation by X-ray diffraction (XRD) is required, the brannerite-like interpretation is supported by nanoscale mineral structural evidence [31].

5.4 Fluid activity and conceptual model

Uranium mineralization in the Louzhuangzi area is closely related to interlayer oxidation driven by surficial oxygen-bearing, uranium-bearing fluids [13]. However, the occurrence of nano-scale brannerite-like aggregates and pronounced sulfidation features in the analyzed samples suggests that the grayish-white ore- bearing sandstones may have experienced superimposed transformation by thermal fluids. Under the influence of deep-seated reducing fluids, such as H_2S- or CH_4-bearing fluids, localized re-reduction of the sandstones likely occurred, thereby inhibiting the remobilization and oxidative destruction of uranium mineralization [20-21, 23-24, 31, 59-60]. Based on the current understanding, this study proposes a conceptual model of coupled mineralization control involving braided fluvial sedimentary sandstones and multi-stage metallogenic fluid processes (Fig 19). This model highlights the coupling relationship between sandstone sedimentation, post-depositional alteration, and

uranium mineralization. Nevertheless, due to the lack of fluid inclusion microthermometry and stable isotope constraints, the timing, temperature, and sources of the involved fluids remain to be further verified by future investigations.

Fig. 19 Schematic diagram of the uranium mineralization model in the Louzhuangzi area, including (A) Types of mineral alteration in different fluid environments; (B) Interlayer oxidizing fluid mineralization; (C) Thermal fluid superposition transformation with oil and gas reduction.

6. Conclusions

(1) Uranium mineralization in the Louzhuangzi area is predominantly developed within thick, coarse-grained braided channel sandstones of the lower member of the Toutunhe Formation (J_2t^1).

(2) Pyrite, titanium oxides, clay minerals, and organic matter are spatially associated with uranium minerals, acting as local reducing agents and/or adsorption sites that facilitate the reduction of U^{6+} to U^{4+} and subsequent precipitation.

(3) Uranium mainly occurs in the form of pitchblende, coffinite, and Ti–U aggregates, including brannerite-like nano-scale particles.

(4) The alteration of the ore-bearing sandstones reflects at least three stages of fluid activity: (i) interlayer oxidation by surficial oxygen-bearing, uranium-rich fluids; (ii) post-mineralization superimposed transformation by thermal fluids; and (iii) localized reduction induced by hydrocarbon influx.

Corresponding Author

Fengjun Nie — School of Earth Sciences, East China University of Technology,

Nanchang, Jiangxi, China; Email: niefj@263.net.

Author Information

Qing Wang — School of Earth Sciences, East China University of Technology, Nanchang, Jiangxi, China; Email: destinw@126.com.

Author Contributions

Conceptualization: Qing Wang, Fengjun Nie.

Data curation: Qing Wang, Xin Zhang.

Formal analysis: Fengjun Nie, Qing Wang, Xin Zhang.

Funding acquisition: Fengjun Nie, Dong-guang Yang.

Investigation: Qing Wang, Fengjun Nie, Xin Zhang, Weiwei Jia, Kegai Lu, Xiao Sun.

Methodology: Fengjun Nie, Kegai Lu.

Project administration: Fengjun Nie, Fei Xia.

Resources: Fengjun Nie, Weiwei Jia, Kegai Lu, Xiao Sun.

Writing – original draft: Qing Wang.

Writing – review & editing: Qing Wang, Fei Xia.

Data Availability

All data generated or analyzed during this study are attached.

Declaration of Competing Interest

The authors declare that they have no known competing financial interests or

personal relationships that could have appeared to influence the work reported in this paper.

Funding Projects: In this study, the field geological investigation and sample preparation were financially supported by the National Natural Science Foundation of China (No. U2244205 and No. U2067202). The laboratory analytical work was supported by the Joint Innovation Fund Project of China Uranium Industry Company Limited – State Key Laboratory of Nuclear Resources and Environment, East China University of Technology (2023NRE-LH-10).

Acknowledgments: We thank the PLOS ONE editors for their support, all contributors to fieldwork and laboratory analyses, and the reviewers for their constructive suggestions that significantly enhanced this manuscript.

References

1. Cai, Y. Q., Zhang, J. D., Li, Z. Y., Guo, Q. Y., Song, J. Y., Fan, H. H., Liu, W. S., Qi, F. C., Zhang, M. L. 2015. Outline of uranium resources characteristics and metallogenetic regularity in China. Acta Geologica Sinica 89(6):1051–1069. https://kns.cnki.net/KCMS/detail/detail.aspx?dbcode=CJFQ&dbname=CJFDLAST2015&filename=DZXE201506005. [in Chinese]
2. Zhang, J. D. 2016. Innovation and development of metallogenic theory for sandstone type uranium deposit in China. Uranium Geology 32(6):321–332. doi:

10. 3969/j issn 1000-0658. 2016. 06. 001. [in Chinese]

3. Geng, Y. Y., Liu, Z. Y., He, Z. B., Huang, S. H., Guo, Q., Xu, Q., Song, J. Y. 2020. Provenance and ore-forming tectonic setting of sandstone-type uranium deposits in the southern margin of Junggar Basin——Insights from U-Pb ages and Hf isotopes of detrital zircons. Geological Review 66(2):393–409. doi: 10.16509/j.georeview.2020.02.010. [in Chinese]

4. Cheng, Y. H., Jin, R. S., Cuney, M., Petrov, V. A., Miao, P. S. 2024. The strata constraint on large scale sandstone-type uranium mineralization in Meso-Cenozoic basins, northern China. Acta Geologica Sinica 98(7):1953–1976. doi: 10.19762/j.cnki.dizhixuebao.2023299. [in Chinese]

5. Huang, S. J. 1994. Formation conditions and prospecting criteria for sandstone uranium deposits of interlayered oxidation type. Uranium Geology (1):6–13. https://kns.cnki.net/KCMS/detail/detail.aspx?dbcode=CJFQ&dbname=CJFD9495&filename=YKDZ401.001. [in Chinese]

6. Chen, Z. Y. 2002. Regional distribution regularity of sandstone uranium deposits in Asian continent and prospecting strategy for sandstone uranium deposits in China. Uranium Geology (3):129–137. https://kns.cnki.net/KCMS/detail/detail.aspx?dbcode=CJFQ&dbname=CJFD2002&filename=YKDZ200203000. [in Chinese]

7. Wang, G. 2002. Mountain–basin formation and sandstone-type uranium mineralization in Xinjiang. Xinjiang Geology 20(2):110–114. https://kns.cnki.net/KCMS/detail/detail.aspx?dbcode=CJFQ&dbname=CJFD200

2&filename=XJDI200202006. [in Chinese]

8. Chen, D. S., Li, S. X., Cai, Y. Q. 2006. Overview of the researches on sedimentary environment for sandstone-type uranium deposits in the Mesozoic–Cenozoic basins of China. Acta Sedimentologica Sinica 24(2):223–228. https://kns.cnki.net/KCMS/detail/detail.aspx?dbcode=CJFQ&dbname=CJFD2006&filename=CJXB200602008. [in Chinese]

9. Wang, G.R., Jia, W. W., Du, M., Huang, S., Lu, K.G., Sun, X. 2021. Relationship between jurassic braided river depositional system and uranium mineralization in the Southern Margin of Junggar Basin. Uranium Geology 37(3) :342–54. doi: 10.3969/j.issn.1000-0658.2021.37.037. [in Chinese]

10. Geng Y.Y, Liu Z.Y, Huang S.H, He Z.B. Metallogenic environment and prospecting direction of sandstone-type uranium deposits in the Toutunhe area, southern margin of Junggar Basin. Uranium Geology. 2022;38(2):194–206. https://kns.cnki.net/KCMS/detail/detail.aspx?dbcode=CJFQ&dbname=CJFDLAST2022&filename=YKDZ202202004. [in Chinese]

11. Wu Y.P. The uranium mineralization characteristics of the Liuhuang'gou area in the southern margin of Junggar Basin [dissertation]. Beijing: China University of Geosciences (Beijing); 2014. [in Chinese]

12. Chen, Z.L., Lu, K.G., Wang, G., Chen, B.L., Wang, G.R., Zheng, E.J., Cui, L.L., Ding, W.J. 2010. Characteristics of Cenozoic structural movements in southern margin of Junggar basin and its relationship to the mineralization of sandstone-type uranium deposits, Acta Petrologica Sinica, 26(2): 457-470.

https://kns.cnki.net/KCMS/detail/detail.aspx?dbcode=CJFQ&dbname=CJFD2010&filename=YSXB201002012. [in Chinese]

13. Lu, K.G., Du, M., Sun, X., Jia, W. W., Wang, S.Y. 2023. Metallogenic controlling factors and genetic analysis of sandstone uranium deposit in the Lower Member of Toutunhe Formation in Louzhuangzi area, Southern Junggar Basin. Uranium Geology 39(4): 507–21. doi: 10.3969/j.issn.1000-0658.2023.39.043. [in Chinese].
14. Huang, S., Jia, W. W., Yan, J. J. 2023. Discussion on the relationship between sand body characteristics and uranium mineralization in the Lower Member of Toutunhe Formation in Louzhuangzi area, Southern Margin of Junggar Basin. Uranium Geology (4): 522–32. doi:10.3969/j.issn.1000-0658.2023.39.044. [in Chinese]
15. Jia W.W., Wang, G.R., Tang, X.F., Huang, S., Yan, J. J. 2023. Discussion on the relationship of different types of alteration zones to the uranium mineralization in Toutunhe Formation of Louzhuangzi area, Southern Margin of Junggar Basin. World Nuclear Geoscience 40(2):152–61. doi:10.3969/j.issn.1672-0636.2023.02.002. [in Chinese]
16. He, Z.B., Hu, B.Q., Qiu, L.F., Wang, Y., Chen, H., Jia, W.W., Li, Y.F., Ji, H.L., Zhu, M.H. 2024. The role of hydrocarbons in the formation of uranium mineralization, Louzhuangzi district, Southern Junggar Basin (China). Minerals 14(7):709. doi: 10.3390/min14070709.
17. Yang, Z. H., Dong, Y., Liu, C., Liu, M., Zhu, Y. S., Zhou, Y. 2022. Study on the occurrence state of uranium in the Toutunhe Formation of Middle Jurassic in Liuhuang'gou region, southern margin of Junggar Basin. Uranium Geology

38(6):1032–1042. doi:10.3969/j.issn.1000-0658.2022.38.091. [in Chinese]

18. Finch, W. I. 1967. Geology of epigenetic uranium deposits in sandstone in the United States. U.S. Geological Survey Professional Paper 538, 121 p. doi: 10.3133/pp538.

19. Reynolds, R. L., Goldhaber, M. B. 1978. Origin of a South Texas roll-type uranium deposit: I. Alteration of iron-titanium oxide minerals. Economic Geology 73(8):1677–1689. doi: 10.2113/gsecongeo.73.8.1677.

20. Nie, F. J., Yan, Z. B., Xia, F., He, J. F., Zhang, C. Y., Feng, Z. B., Zhang, X., Yang, D. G., Chen, M. Y., Tan, S. J., Zhang, J., Kang, S. H., Ning, J., Yang, J. X., Shen, K. F., Cai, J. F. 2021. Two-stage and two-mode uranium mineralization for sandstone-type uranium deposits. Acta Geoscientica Sinica 42(6):823–848. doi: 10.3975/cagsb.2021.112201. [in Chinese]

21. Yan, Z. B., Zhang, W. W., Zhang, C. Y., Xia, F., Nie, F. J., Wu, X. Y. 2023. The influence of fluid activity from deep basin on the metallogenic process of sandstone type uranium deposits. Acta Geologica Sinica 97(12):4131–4149. doi: 10.19762/j.cnki.dizhixuebao.2023169. [in Chinese]

22. Liu, Y., Zhao, J. H., Jiao, Y. Q., Liu, Z. R., Zhou, M. F. 2023. Constraints of in-situ S-isotopic compositions of pyrite on the genesis of the Bayinqinggeli sandstone-hosted uranium deposit, Ordos Basin, northern China. Ore Geology Reviews 161:105608. doi: 10.1016/j.oregeorev.2023.105608.

23. Liu, C. Y., Zhang, L., Huang, L., Wu, B. L., Wang, J. Q., Zhang, D. D., Tan, C. Q., Ma, Y. P., Zhao, J. S. 2024. Novel metallogenic model of sandstone-type uranium

deposits: Mineralization by deep organic fluids. Earth Science Frontiers 31(1):368–383. doi: 10.13745/j.esf.sf.2024.1.7. [in Chinese]

24. Sun, D., Li, H. M., Xia, F., Nie, F. J., Huang, G. W., Zhang, Z. B., Meng, F. M., Pan, J. Y., Hu, Y. J. 2024. Provenance and tectonic setting of the Lower Cretaceous Huanhe Formation in the northwestern Ordos Basin and its implications for uranium mineralization. ACS Omega 9(3):3324–3341. doi: 10.1021/acsomega.3c06163.

25. Nie, F. J., Yan, Z. B., Xia, F., Li, M. G., Lu, Y. Y., Cai, J. F., Guo, F. N., Ning, J. 2017. Hot fluid flows in the sandstone-type uranium deposit in the Kailu basin, Northeast China. Geological Bulletin of China 36(10):1850–1866. [in Chinese]

26. Qin, M. K., Huang, S. H., Liu, J. L., Liu, Z. Y., Guo, Q., Jia, L. C., Jiang, W. J. 2021. Hydrothermal alteration and its superimposed enrichment for the Qianjiadian tabular-type uranium deposit in the southwestern Songliao Basin. Minerals 12(1):52. doi: 10.3390/min12010052.

27. Li, Z. Y., Liu, W. S., Li, W. T., Li, X. D., Qin, M. K., Cai, Y. Q., Zhang, Y. L., et al. 2022. Exudative metallogeny of the Hadatu sandstone-type uranium deposit in the Erlian Basin, Inner Mongolia. Geology in China 49(4):1009–1047. doi: 10.12029/gc20220401. [in Chinese]

28. Liu, B., Hao, P., Zhang, Z. L., Guo, X. J., Zhang, P. F., Shi, Z. Q. 2023. Geology, alteration system and uranium mineralization of Tamusu large uranium deposit, China. Ore Geology Reviews 158:105488. doi: 10.1016/j.oregeorev.2023.105488.

29. Liu, W. S., Li, Z. Y., Liu, C. H., Ji, H. W., Li, X. D. 2024. Exudative metallogeny

and model of Saihan Gaobi sandstone-type uranium deposit in Erlian Basin. Uranium Geology 40(1):129–142. Doi: 10.3969/j.issn.1000-0658.2024.40.011. [in Chinese].

30. Tang, C., Xu, Z. L., Duan, M., Meng, L. S., Liu, H. J., Wei, J. L., Zhang, C., Zhao, L. J. 2025. Genetic model of the Luhai sandstone-type uranium deposit in the Erlian Basin, Inner Mongolia. Minerals 15(3):294. doi: 10.3390/min15030294.

31. Wang, Q., Nie, F. J., Yang, Y. P., Xian, H. Y., Xia, F., Yan, Z. B., Zhang, X., Yang, D. G., Jia, W. W., Meng, F. M., Sun, D., Yan, J. J., Huang, S. 2025. Brannerite identification in the Louzhuangzi sedimentary U deposit and implications for deposit genesis or post-ore alteration. Ore Geology Reviews 106908. doi: 10.1016/j.oregeorev.2025.106908.

32. Hu, X. W., Yang, X. Y., Ren, Y. S., Wu, Z. J., Du, G. F., Huang, T. T. 2020. Controls of sedimentary environment and tectonic evolution on sandstone-type uranium mineralization in the Junggar Basin. Geotectonica et Metallogenia 44(4):725–741. doi: 10.16539/j.ddgzyckx.2020.04.013. [in Chinese]

33. Wu, Z.H., Jiang, W., Zhou, J.R., Li, J.S. 2001. Thermal-chronological dating on the thermal history of plutons and tectonic-landform evolution of the central tibetan plateau. Acta Geologica Sinica (4):468–76. [in Chinese]

34. Fang, S.H., Guo, Z. J., Wu, C. D., Zhang, Z. C., Wang, M. N., Yuan, Q. D. 2006. Jurassic clastic composition in the Southern Junggar Basin, Northwest China: implications for basin-range pattern and tectonic attributes. Acta Geologica Sinica (2):196–209. [in Chinese]

35. Fang, S.H., Song, Y., Jia, C.Z., Guo, Z.J., Zhang, Z.C., Liu, L.J. 2007. Timing of Cenozoic intense deformation at the north margin of Tianshan and its implications for petroleum accumulation. Earth Science Frontiers 14(2) :205-214. [in Chinese]

36. Han, B.F., Guo, Z.J., Zhang, Z.Ch., Zhang, L.Z., Chen, J.F., Song, B. 2010. Age, geochemistry, and tectonic implications of a late Paleozoic stitching pluton in the North Tian Shan suture zone, western China. GSA Bulletin 122(3–4):627–40. doi: 10.1130/B26491.1.

37. Li, W.H., Xu, G. Z., Quan, J. P. 2010. Late modification of the intermountain basins of the Tien Shan orogenic belt and sandstone-type uranium mineralization. Mineral Deposits 29(S1):141–42. doi: 10.16111/j.0258-7106.2010.s1.081. [in Chinese]

38. Xiao, X.C., Tang, Y.Q., Wang, J., Gao, J., Zhao, M. 1994. Blueschist belts and their tectonic implications of the S. Tianshan Mts., N. W. China. Acta Geoscientia Sinica (Z2). [in Chinese]

39. Guan, X.T., Wu, C.D., Wu, J., Zhou, J.Q., Jiao, Y., Zhou, R., Yu, Q.S. 2020. Sedimentary sequence and depositional environment evolution of Upper Jurassic-Lower Cretaceous strata in the Southern Margin of Junggar Basin. Xinjiang Petroleum Geology 41(1):67–79. doi: 10.7657/XJPG20200109. [in Chinese]

40. Wang, D.Y., Fu, Y.Q., 1981. Uranium Mineralogy (in Chinese). Atomic Energy Press, Beijing, p. 109.

41. Folk， R.L. 1968. Petrology of Sedimentary Rocks[Z]. U.S.A: Hemphill Publishingc，Austin，1968:170.

42. Yin, Z.Q., Qin, X. G., Wu, J. S., Ning, B. 2009. The multimodal grain-size

distribution characteristics of loess desert lake and river sediments in some areas of Northern China. Acta Sedimentologica Sinica 27(2):343–51. Doi: 10.14027/j.cnki.cjxb.2009.02.009. [in Chinese]

43. Wang H.W. Sedimentary microfacies and evolutionary simulation of a meandering river in the NmIII oil group, Yangerzhuang Oilfield [dissertation]. Qingdao: China University of Petroleum (East China); 2024. [in Chinese]

44. Tang, Y.N., Hu, C.L., Dan, S.H., Han, C.C., Liu, Z.M. 2022. Depositional model for the early triassic braided river delta and controls on oil reservoirs in the eastern Junggar Basin, Northwestern China. Minerals 12(11):1409. doi: 10.3390/min12111409.

45. Liu, S., Li, J.B., Jin, F.B., Liu, H.F., Wang, M., Cheng, Y.L., Zhong, X.H., Lu, H. 2024. Genesis of efficient reservoirs in mixed sedimentary setting: A case study of Benxi Formation in the Gaoqiao area, Ordos Basin. Turkish Journal of Earth Sciences 33(2):118–45. doi: 10.55730/1300-0985.1903.

46. Wang, J., Gao, X. J., Zhou, X. M. 2019. Reservoir achitecture characterization and remaining oil distribution of meandering river. Journal of China University of Petroleum 43(3):13–24. [in Chinese]

47. Zhu, Z.J., Huang, B.H., Guo, F.S., Zheng, H.F., Jiang, Y.B. 2012. Cretaceous braided river facies sediments and Danxia landform development characteristics in Longhushan World Geopark, Jiangxi. Acta Geoscientia Sinica 33(3):379–87. doi: 10.3975/cagsb.2012.03.12. [in Chinese]

48. Liu, Y.M., Chen, R.X., Zhao, D.D., Zhang, Z.Y., Wang, J., Hou, J.G. 2022.

Sedimentary characteristics of gravelly braided rivers of the Lower Shihezi Formation in the Jin 72 well area, Dongsheng gas field. Petroleum Science Bulletin 7(4):457–74. doi: 10.3969/j.issn.2096-1693.2022.04.040. [in Chinese]

49. Jolivet, M., Bourquin, S., Heilbronn, G., Robin, C., Barrier, L., Dabard, M.P., Jia, Y.Y., De Pelsmaeker, E., Fu, B.H. 2017. The Upper Jurassic–Lower Cretaceous alluvial-fan deposits of the Kalaza Formation (Central Asia): Tectonic pulse or increased aridity? Geological Society, London, Special Publications 427(1):491–521. doi: 10.1144/SP427.6.

50. Jiao, Y. Q., Wu, L. Q., Rong, H., Zhang, F., Le, L., Song, H., Tao, Z. P., Peng, H., Sun, Y. H., Xiang, Y. 2022. Sedimentation, diagenesis and uranium mineralization: innovative discoveries and cognitive challenges in study of sandstone-type uranium deposits in China. Earth Science 47(10):3580–3602. https://doi.org/10.3799/dqkx.2022.284. [in Chinese]

51. Peng, S. L., Tian, J. P., Guo, X. Y., Jin, Y. P., Sun, B., Dou, H. X., Zhang, F., Hao, J., Hu, Y. L., Chen, L., Xu, W., Tong, Y. 2023. Characteristics and metallogenesis of ore-bearing sandstone in the Tarangaole sandstone type uranium deposit, Ordos Basin. Geology in China 50(1):264–276. doi: 10.12029/gc20200814001. [in Chinese]

52. Castillo-Oliver, M., Melgarejo, J. C., Torró, L., Villanova-de-Benavent, C., Campeny, M., Díaz-Acha, Y., Amores-Casals, S., Xu, J., Proenza, J., & Tauler, E. 2020. Sandstone-Hosted Uranium Deposits as a Possible Source for Critical Elements: The Eureka Mine Case, Castell-Estaó, Catalonia. Minerals, 10(1), 34.

https://doi.org/10.3390/min10010034.

53. Song, H., Ni, S. J., Hou, M.C., Zhang, C.J., Shi, Z.Q., Wang, G., Yang, B., Hu, Y., Chen, Y.J. 2016. The characteristic of clay minerals in sandstone-type uranium deposit in the Yili Basin, NW China and its relationship with uranium mineralization. Acta Geologica Sinica 90(12):3352–66. http://www.geojournals.cn/dzxb/ch/index.aspx. [in Chinese]

54. Spirakis, C. S. (1996). The roles of organic matter in the formation of uranium deposits in sedimentary rocks. Ore Geology Reviews, 11(1), 53 ~ 69. https://doi.org/10.1016/0169-1368(95)00015-1.

55. Bonnetti, C., Cuney, M., Michels, R., Truche, L., Malartre, F., Liu, X., Yang, J. 2015. The multiple roles of sulfate-reducing bacteria and Fe-Ti oxides in the genesis of the Bayinwula roll front-type uranium deposit, Erlian Basin, NE China. Economic Geology 110(4):1059–81. doi: 10.2113/econgeo.110.4.1059.

56. Cumberland, S. A., Evans, K., Douglas, G., de Jonge, M., Fisher, L., Howard, D., & Moreau, J. W. (2021). Characterisation of uranium-pyrite associations within organic-rich Eocene sediments using EM, XFM-μXANES and μXRD. Ore Geology Reviews, 133, 104051. https://doi.org/10.1016/j.oregeorev.2021.104051.

57. Yin, S., Yan, Z.B., Fu, J.L., Zhang, W., Liu, H., Xia, F., Wang, Q.F. 2024. Polymorphic transformations of titanium oxides contribute to economic uranium mineralization in sandstone. Geology. doi: 10.1130/G51982.1.

58. Chen, R., Siidra, O. I., Firsova, V. A., Arevalo-Lopez, A., Colmont, M., Ugolkov, V. L., Bocharov, V. N. 2023. The chemistry, recrystallization and thermal

expansion of brannerite from Akchatau, Kazakhstan. Materials 16(4):1719. doi: 10.3390/ma16041719.

59. Hall S.M, van Gosen B.S, Zielinski R.A. Sandstone-hosted uranium deposits of the Colorado Plateau, USA. Ore Geology Reviews. 2023;155:105353. doi:10.1016/j.oregeorev.2023.105353.

60. Qiu, L. F., Li, Z. Y., He, F., Wu, Z. Q., Liu, K. P., Zhang, L., Mao, N., Wang, T. T., Wang, L. 2025. Contribution of deep organic fluids to uranium mineralization in Cretaceous sandstones of the southwestern Ordos Basin, China. Hydrogeology Journal 33(4):925–944. doi: 10.1007/s10040-025-02912-4.

Supporting information

S1 Fig. (A) **Sketch map showing tectonic location of Junggar Basin** (modified from [38]); (B) **Geological sketch map of Louzhuangzi area** (modified from [15]); (C) **Division and distribution of the main tectonic units at southern margin of Junggar Basin** (modified from [12]).

S2 Fig. Stratigraphic column of the Louzhuangzi area (modified after an internal report from Geological Party No. 216, CNNC, 2022).

S3 Fig. Petrographic photographs of Toutunhe Formation (J_2t) outcrops in Louzhuangzi area: (A-C) Sandstone outcrop of the Upper Member, Toutunhe Formation (J_2t^2), showing fine-medium sandstone with parallel beddings (A), mudstone with massive beddings (B), fine-medium sandstone with horizontal beddings (B), and fine sandstone with flaser beddings and planar cross beddings (C); (D-F) Sandstone

outcrop of the Lower Member, Toutunhe Formation (J_2t^1), showing sandstone with large to giant planar cross beddings (D), conglomerate with massive beddings (E), and glutenite and coarse sandstone with trough cross beddings (F); (G) Interface between the Lower Member of the Toutunhe Formation (J_2t^1) and the Xishanyao Formation (J_2x); (H) Sandstone outcrop of the Xishanyao Formation (J_2x), showing siltstone with parallel beddings and coal lines.

S4 Fig Classification diagram of sandstones in Toutunhe Formation (J_2t), Louzhuangzi area (According to [41]).

S5 Fig Petrographic characteristics of sandstone samples from Toutunhe Formation (J_2t) in Louzhuangzi area under microscope: (A) Sharp-edged quartz clasts cemented by basal carbonate cementation, ZK5-1, 22ZGE033, 148.50 m, (+); (B) Clastics and clay matrix, ZK6-2, 22ZGE016, 477.30m, (+); (C) Pink crystalline quartz and feldspar matrix filled among debris particles, ZK6-2, 22ZGE016, 477.30m, (+); (D) Illitizated plagioclase with relatively dirty surface, ZK6-2, 22ZGE063, 478.60m, (+); (E) Lattice twin crystal microcline, ZK6-2, 22ZGE014, 483.47m, (+); (F) Bending and crushing of serimicized biotite, ZK12-2, 22ZGE001, 672.48m, (+); (G) Pyrite cementation, ZK4-2, 23ZGE026, 398.20m (reflected light); (H) Fine-grained pyrite and uranium minerals filled the cell cavities of carbonaceous debris, ZK4-2, 23ZGE026, 398.20 m, (BSE image); (I) Pyrite and uranium minerals filled among the clastic particles, ZK12-3, 23ZGE028, 798.50 m, (BSE image).

S6 Fig. Debris characteristics of sandstone samples from Toutunhe Formation (J_2t) in Louzhuangzi area under microscope: (A) Cryptocrystalline tuff debris, ZK6-2,

22ZGE016, 477.30 m, (+); (B) Platy-structure quartz schist debris, ZK6-2, 22ZGE015, 479.30m, (+); (C) Sericitization of sharp edged polycrystalline quartz, ZK6-2, 22ZGE016, 477.30m, (+); (D) Dacite porphyry debris with porphyritic texture, ZK6-2, 22ZGE026, 398.80m, (+); (E) Dissolution pit visible in cryptocrystalline flint debris, ZK6-2, 22ZGE026, 398.80m, (+); (F) Carbonate-cemented granite debris, ZK11-2, 22ZGE034, 24.84 m, (+).

S7 Fig. Photographs of representative core samples from Toutunhe Formation (J_2t) exposed by the economic uranium mineralization drill hole of ZK5-7 in Louzhuangzi area: (A) Meandering river facies ' thick mud with thin sand from the Upper Member of the Toutunhe Formation (J_2t^2); (B) Gravelly braided river facies thick sand body from the Lower Member of the Toutunhe Formation (J_2t^1); (C) Sandy braided river facies flood mud and crevasse splay sand from the bottom of the Lower Member of the Toutunhe Formation (J_2t^1) .

S8 Fig. Interpretation of the core profile of drill hole ZK5-1 in Louzhuangzi area. (Abbreviation: Fm=Formation; DF= Depositional Facies; OD= Overbank Deposit; SCF= Sandy Channel Fill; LD= Lag Deposit; GB= Gravel Bar; FP= Floodplain; CS= Crevasse Splay; CB= Channel Bar; IDB= Interdistributary Bay; SDC= Subaqueous Distributary Channel).

S9 Fig. Sketch diagram showing the A-A' profile in Louzhuangzi area (In the Lower Member of the Toutunhe Formation (J_2t^1), the top roof of the sandstone has been leveled. The profile position is shown in Figure 1C).

S10 Fig. Occurrence characteristics of uranium in sandstones of Toutunhe Formation (J_2t) in Louzhuangzi area (BSE images), including (A) Gelatinous coffinite associated with pyrite, ZK11-3, 23ZGE022, 119.52m; (B) Massive coffinite associated with fine-grained pyrite, ZK12-3, 23ZGE028, 798.50m; (C) Massive pitchblende associated with pyrite, ZK12-3, 23ZGE028, 798.50 m; (D) Baited pitchblende coexisted with coffinite with ring-shaped distribution; ZK4-2, 23ZGE026, 398.20 m; (E) Nanoscale titanium-uranium aggregates associated with chlorite, ZK3-3, 23ZGE027, 753.10m; (F) Nanoscale uranium particles adsorbed in clay minerals, ZK12-3, 23ZGE028, 798.50m; (G) Nanoscale uranium minerals adsorbed at the edge of titanium oxides, ZK4-2, 23ZGE026, 398.20m; (H) Nanoscale uranium minerals adsorbed in the dissolution pores of titanium oxides, ZK12-3, 23ZGE028, 798.50 m; (I) Uranium-rich monazite associated with baited pitchblende, ZK4-2, 23ZGE026, 398.20m; (J) Uranium-rich rutile, ZK4-2, 23ZGE026, 398.20 m; (K-L) Area distribution of U(K) and Ti (L) elements in uranium-rich rutile obtained by electron probe.

S11 Fig. Images showing the characteristics of titanium-containing uranium minerals under FIB and TEM (ZK1202, 22ZGE001, 672.48 m), including (A) Backscattered electron (BSE) image of titanium-containing uranium mineral under focused ion beam (FIB) scanning electron microscope; (B) Image of the transparent foil of titanium-containing uranium mineral under transmission electron microscope (The transparent foil was taken vertically downward at the yellow rectangular position in Figure A, with the thickness <100 nm); (C) HAADF-STEM image of titanium-

containing uranium mineral; (D-I) STEM-EDS-derived area distribution of Ti-U(D), U(E), Ti(F), Ca(G) and Si(H) elements and energy spectra (I) of the black rectangular area in Figure C.

S12 Fig. Epigenetic alteration characteristics of sandstones in Toutunhe Formation (J_2t), Louzhuangzi area, including (A-D) Sand-yellow oxidized sandstone outcrop (A); medium to large planar cross bedding developed in the sandstone (B); incompletely oxidized carbonized plant detritus (C) and layered gypsum (E) occasionally observed; uranium anomaly commonly observed in gravel-bearing coarse sandstone (D) of the Upper Member of the Toutunhe Formation (J_2t^2) at Wanjiayao mineralization site; (F-G) Incompletely oxidized gray residues in the sand-yellow oxidized sandstone (F); uranium anomalies observed in the oxidized sandstone (G) of the Lower Member of the Toutunhe Formation (J_2t^1) at Hasa tomb anomaly site; (H-I) Oxidized sandstone (H) and multiple layers of gypsum (I) of the Upper Member of the Toutunhe Formation (J_2t^2) exposed by drill hole ZK11-3; (J) Laminated carbonized plant detritus and pyrite distribution observed in the grayish-white altered sandstones in the Lower Member of the Toutunhe Formation (J_2t^1) exposed by drill hole ZK 6-4; (K) Kaolin and pyrite nodules developed in the grayish-white altered sandstones in the Lower Member of the Toutunhe Formation (J_2t^1) exposed by drill hole ZK 12-3; (L) Laminated carbonized plant detritus and pyrite distribution exposed by drill hole ZK4-3 in depth of 758.24m.

S13 Fig. Profile of interlayer oxidation zones and uranium mineralization zones in sandstones of Toutunhe Formation (J_2t) in drill hole ZK 11-2 at Hasa tomb anomaly site (with sandstone top roof being leveled).

S14 Fig. Distribution of depositional facies and oxidation zones in the Lower Member of Toutunhe Formation (J_2t^1), Louzhuangzi area (modified from [13]).

S15 Fig. Characteristics of clay minerals in sandstones in Lower Member of Toutunhe Formation (J_2t^1), Louzhuangzi area (BSE images), including (A) Intergranular layered kaolinite, ZK16-2, 23DJA-9, 705.70m; (B) Intergranular worm-like kaolinite, ZK16-2, 23 dJA-6, 654.60m; (C) Intergranular plate-like kaolinite, ZK12-3, 23 dJA-27, 641.70m; (D) Spherical smectite mixed layer mineral on the particle surface , ZK4-3, 23DJA-7, 710.40m; (E) Cellular smectite mixed layer mineral on the particle surface , ZK12-3, 23 DJA-15,871.50m; (F) The strawberry pyrite Encapsulated by flake-like smectite mixed layer mineral, ZK12-2, 23DJA-4, 680.70m; (G-I) Intergranular flake-like chlorite aggregate, ZK16-2, 23DJA-9, 705.70m.

S16 Fig. Association of uranium minerals and clay minerals in sandstones in Lower Member of Toutunhe Formation (J_2t^1), Louzhuangzi area (BSE images), including (A) Uranium mineral adsorbed in chlorite, ZK3-3, 23ZGe027, 753.10m; (B) Uranium mineral associated with chloritized biotite, ZK4-2, 23ZGE026, 398.20m; (C) Uranium minerals associated with chlorite, ZK12-3, 23ZGE028, 798.50m.

S17 Fig. Alteration characteristics of titanium oxides in sandstones in Lower Member of Toutunhe Formation (J_2t^1), Louzhuangzi area. (A-C) Anatase and magnetite mineral assemblage enveloped by gelatinous pyrite (reflected light); (D)

uranium-bearing oxides enveloped by gelatinous pyrite; (E-F) Interwoven association of anatase and pyrite (BSE image).

S18 Fig. Schematic diagram of the B-B ' drill hole profile in Louzhuangzi area. The profile position is shown in Figure 1C (modified from [15-16]).

S19 Fig. Schematic diagram of the uranium mineralization model in the Louzhuangzi area, including (A) Types of mineral alteration in different fluid environments; (B) Interlayer oxidizing fluid mineralization; (C) Thermal fluid superposition transformation with oil and gas reduction.

S1 Table. Property of sandstone samples from Toutunhe Formation (J_2t) of Louzhuangzi area, southern margin of Junggar Basin.

S2 Table. Analysis results of chemical components in uranium-rich minerals in Louzhuangzi area by electron probe (Wt %).

Click here to access/download;Figure;S1_Fig.tif

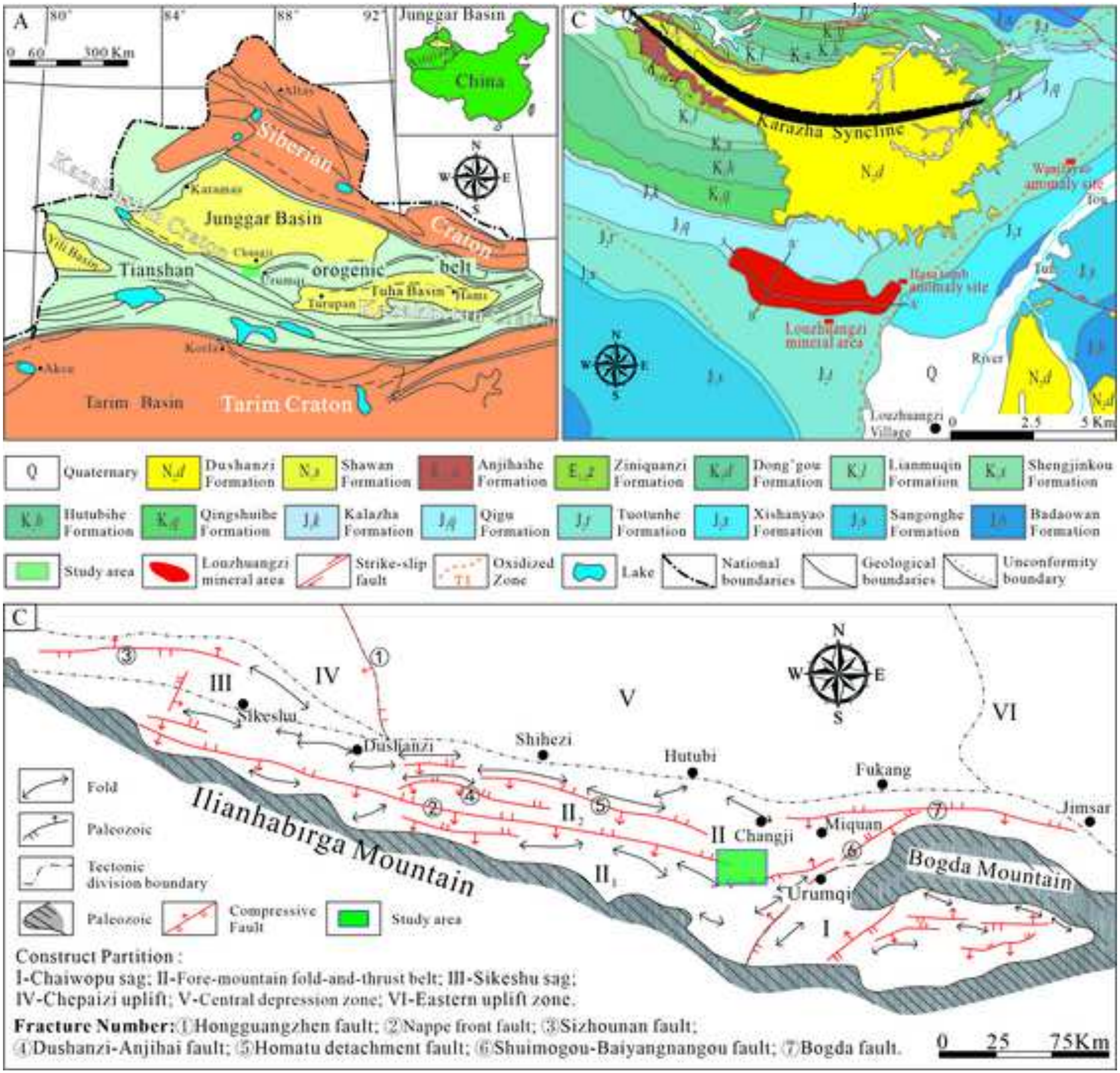

Click here to access/download;Figure;S2_Fig.tif

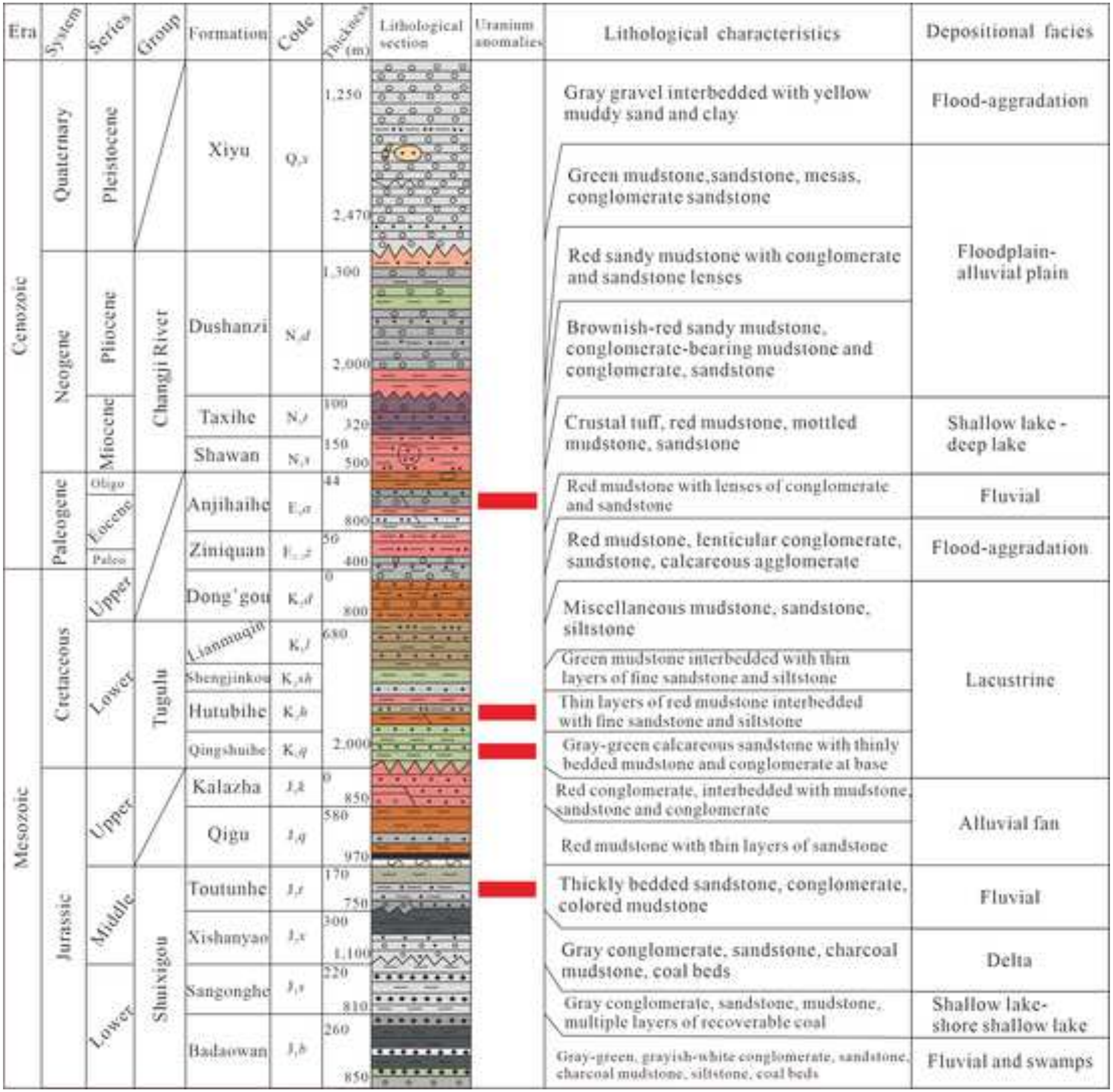

Click here to access/download;Figure;S3_Fig.tif

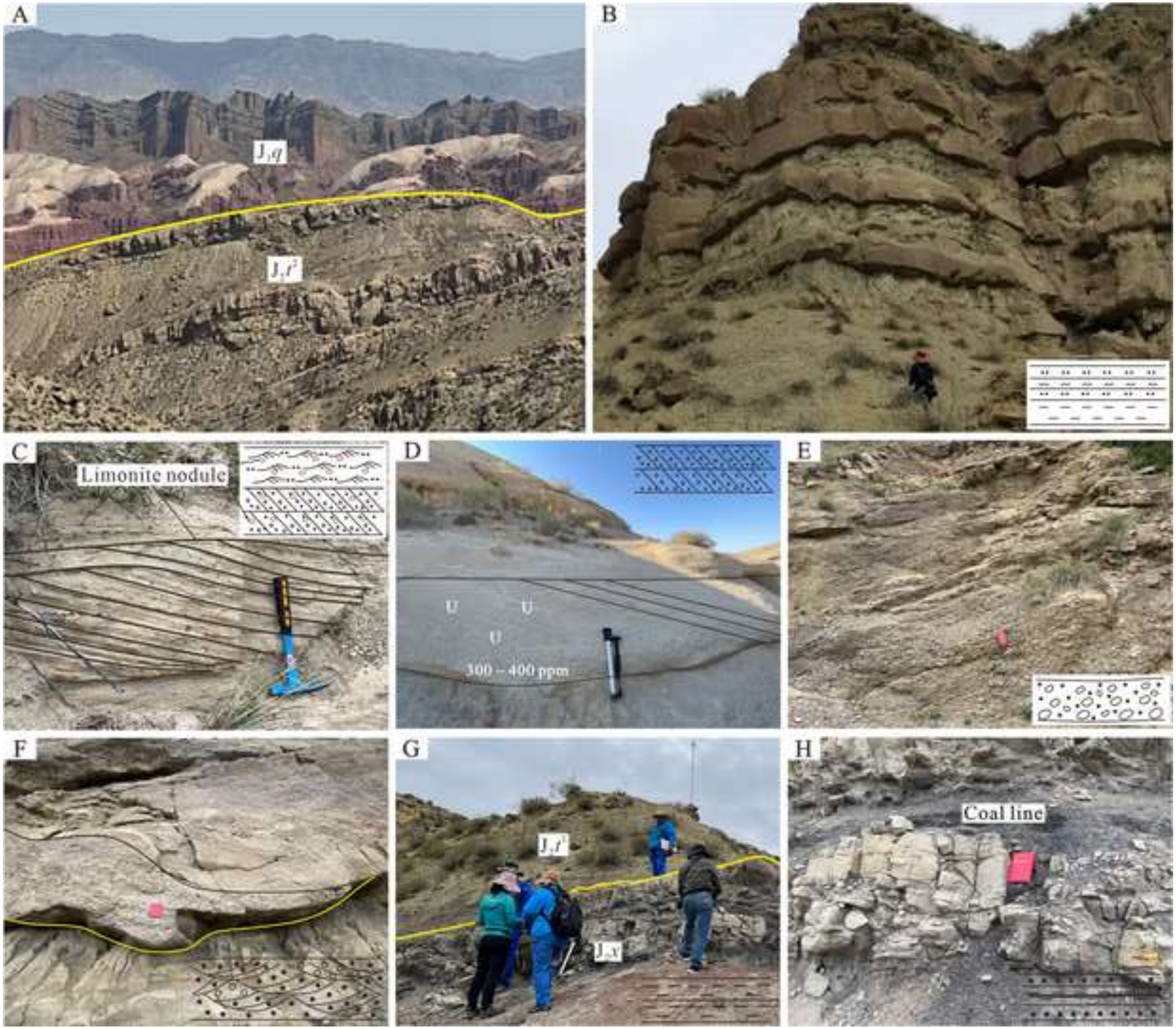

Click here to access/download;Figure;S4_Fig.tif

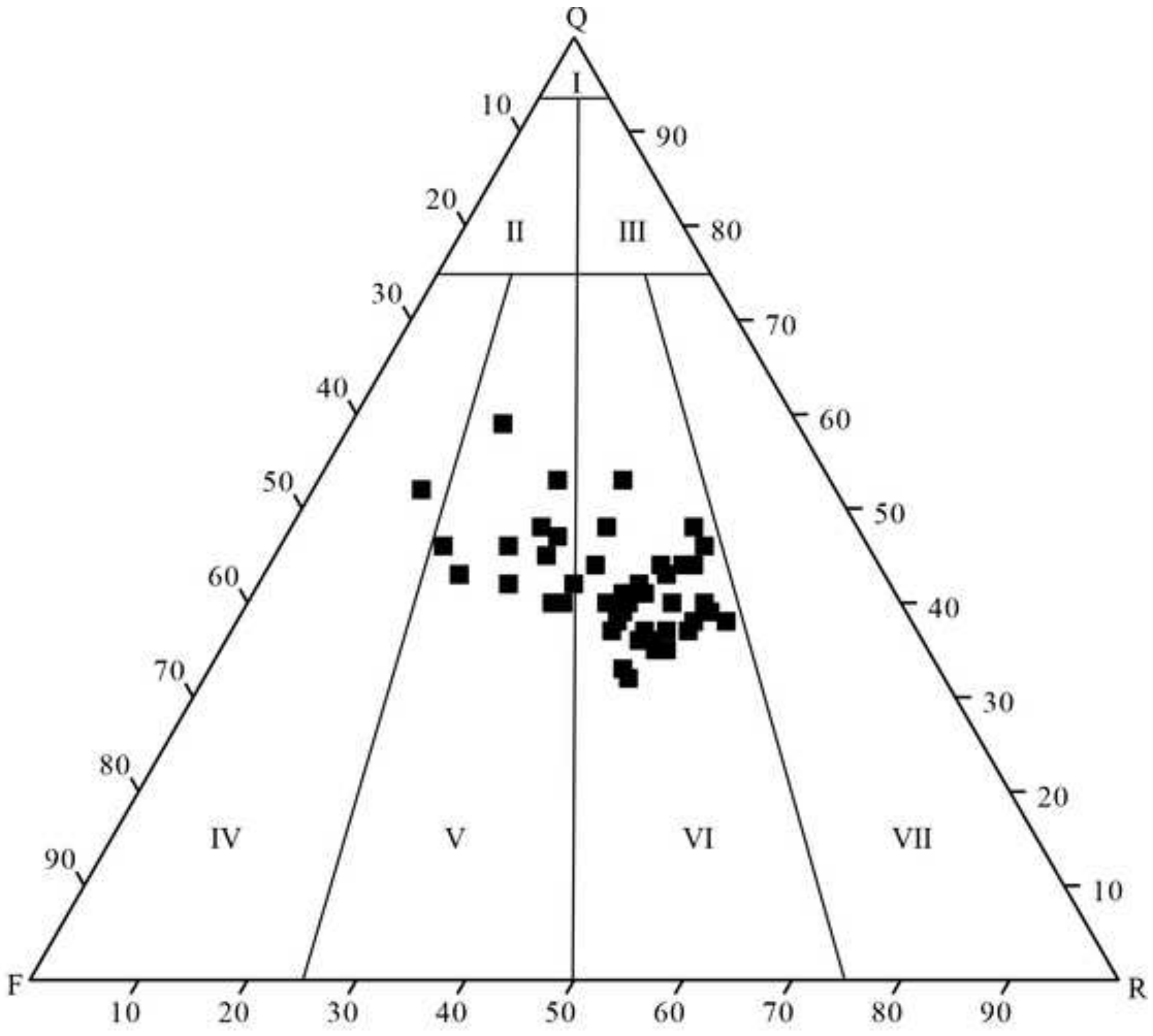

I-Quartz sandstone; II-Feldspathic quartz sandstone; III-Lithic quartz sandstone; IV-Arkose; V-Lithic arkose; VI-Feldspar lithic sandstone; VII-Lithic sandstone

Click here to access/download;Figure;S5_Fig.tif

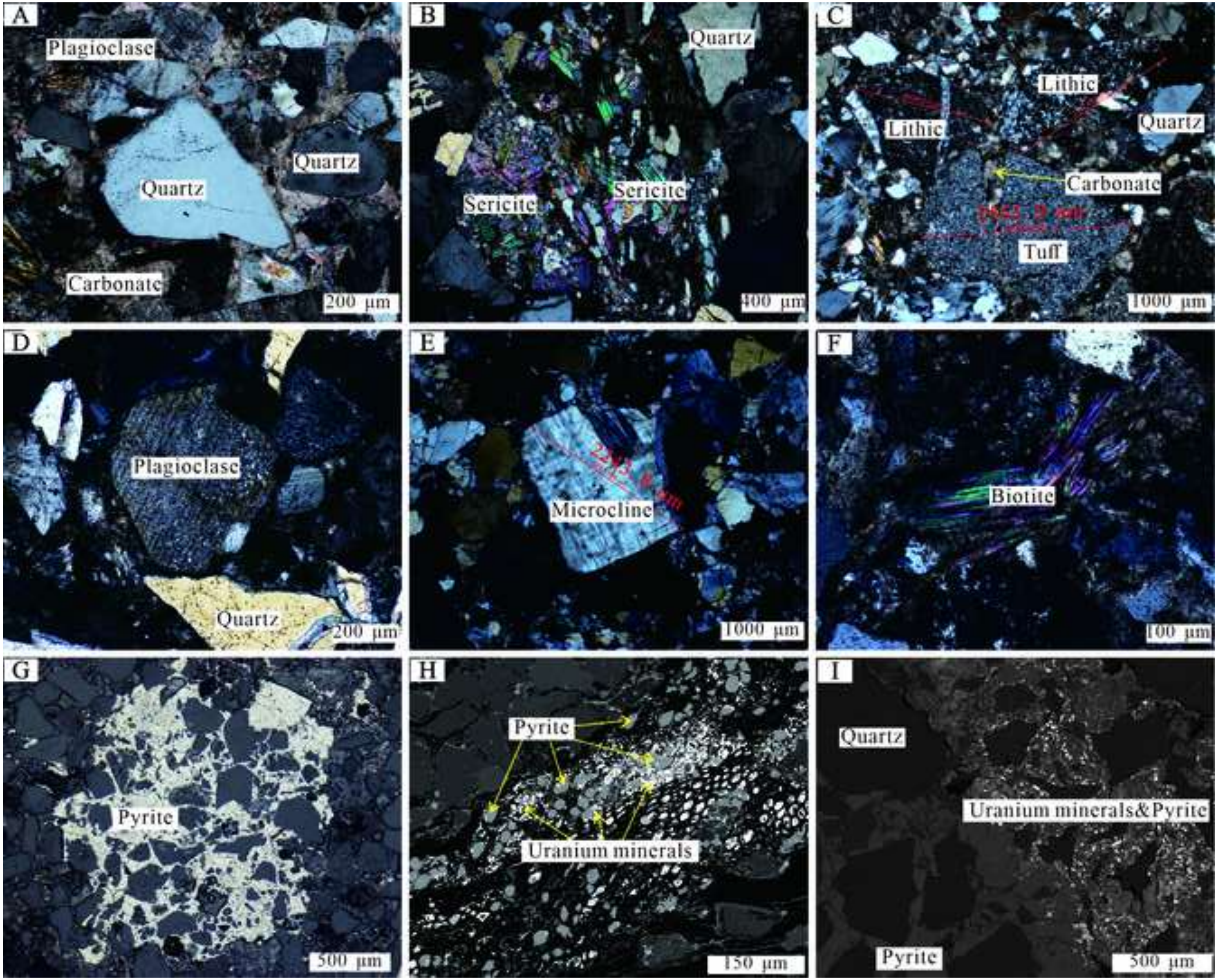

Click here to access/download;Figure;S6_Fig.tif

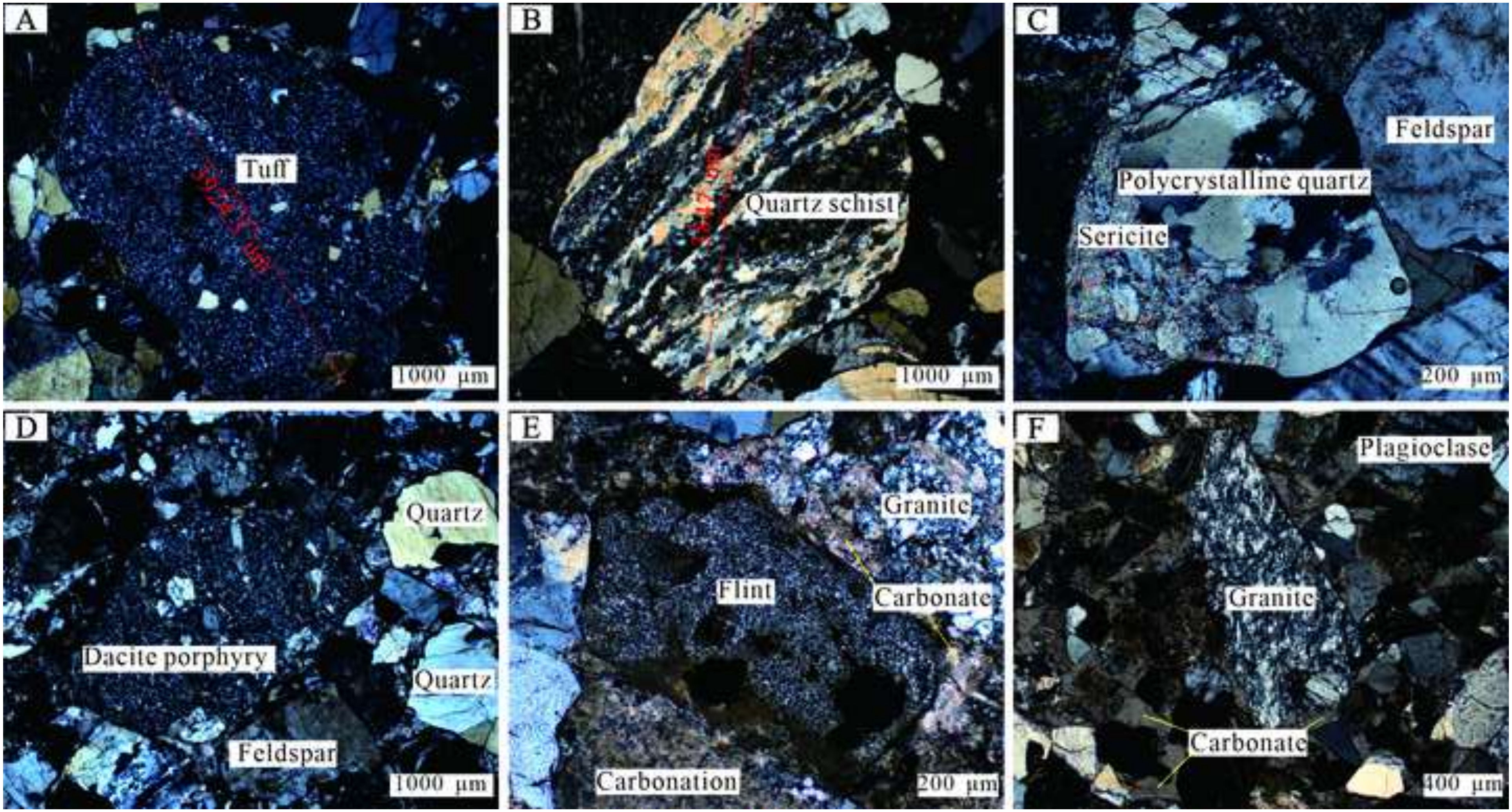

Click here to access/download;Figure;S7_Fig.tif

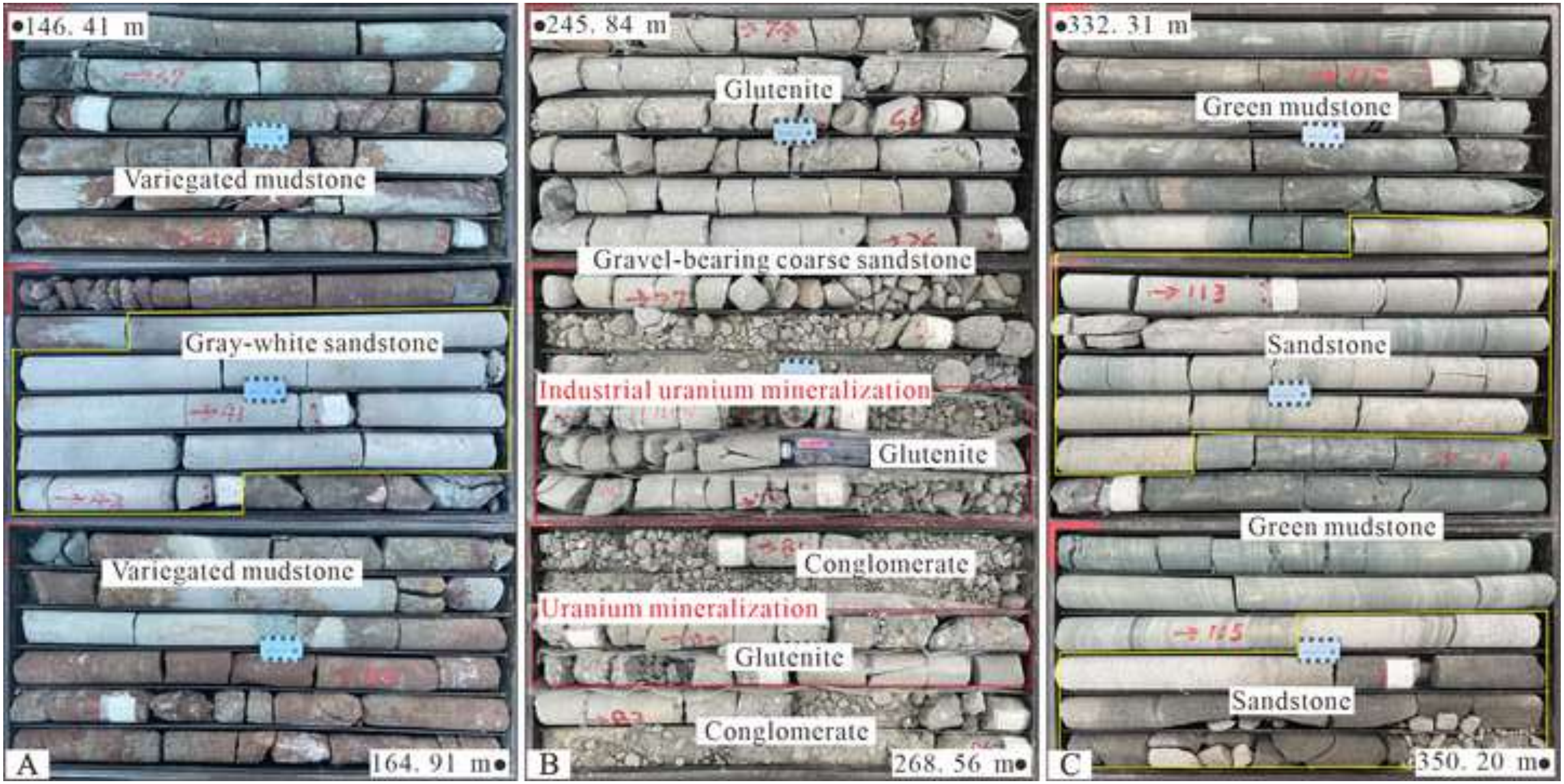

Click here to access/download;Figure;S8_Fig.tif

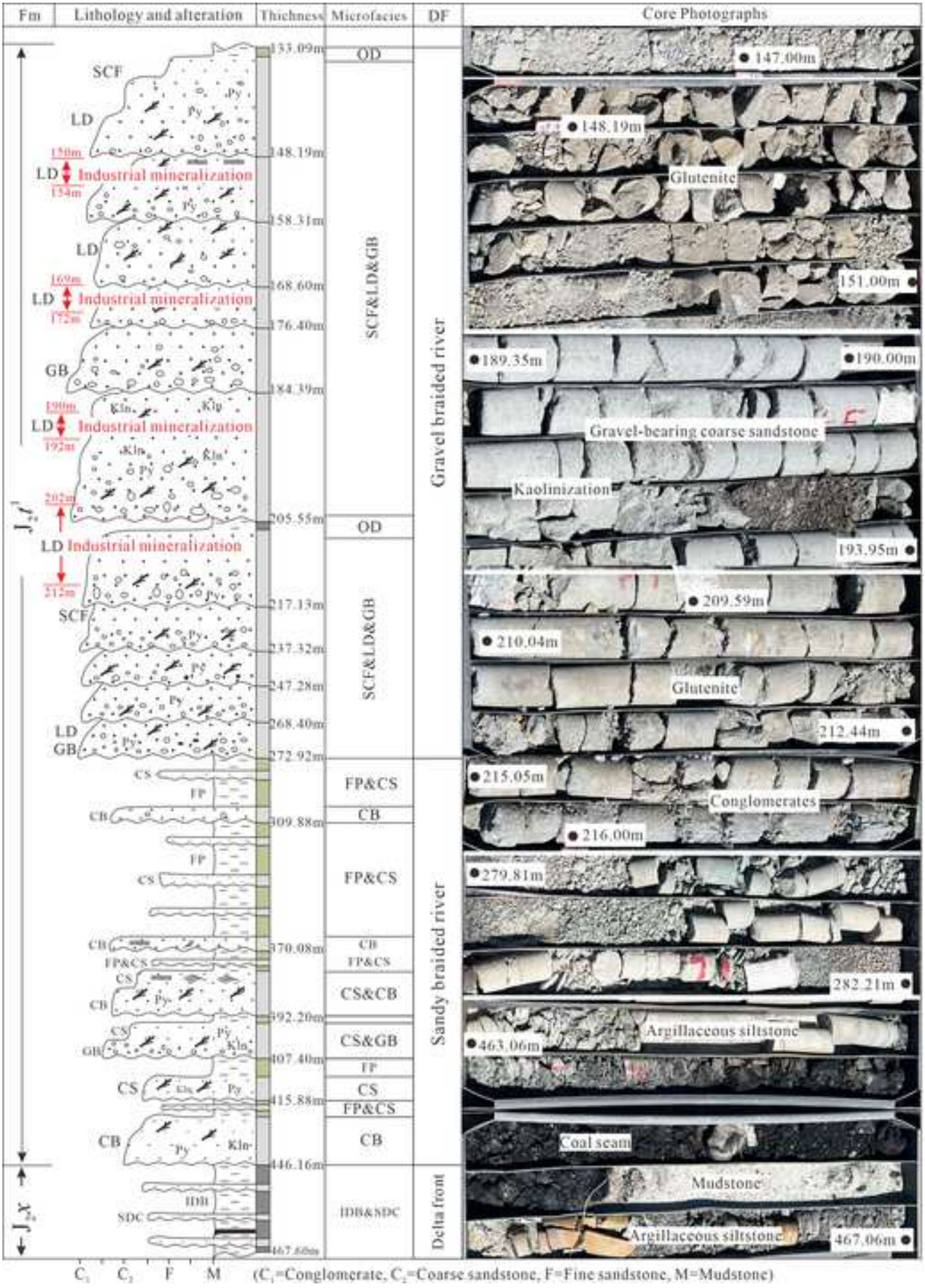

Click here to access/download;Figure;S9_Fig.tif

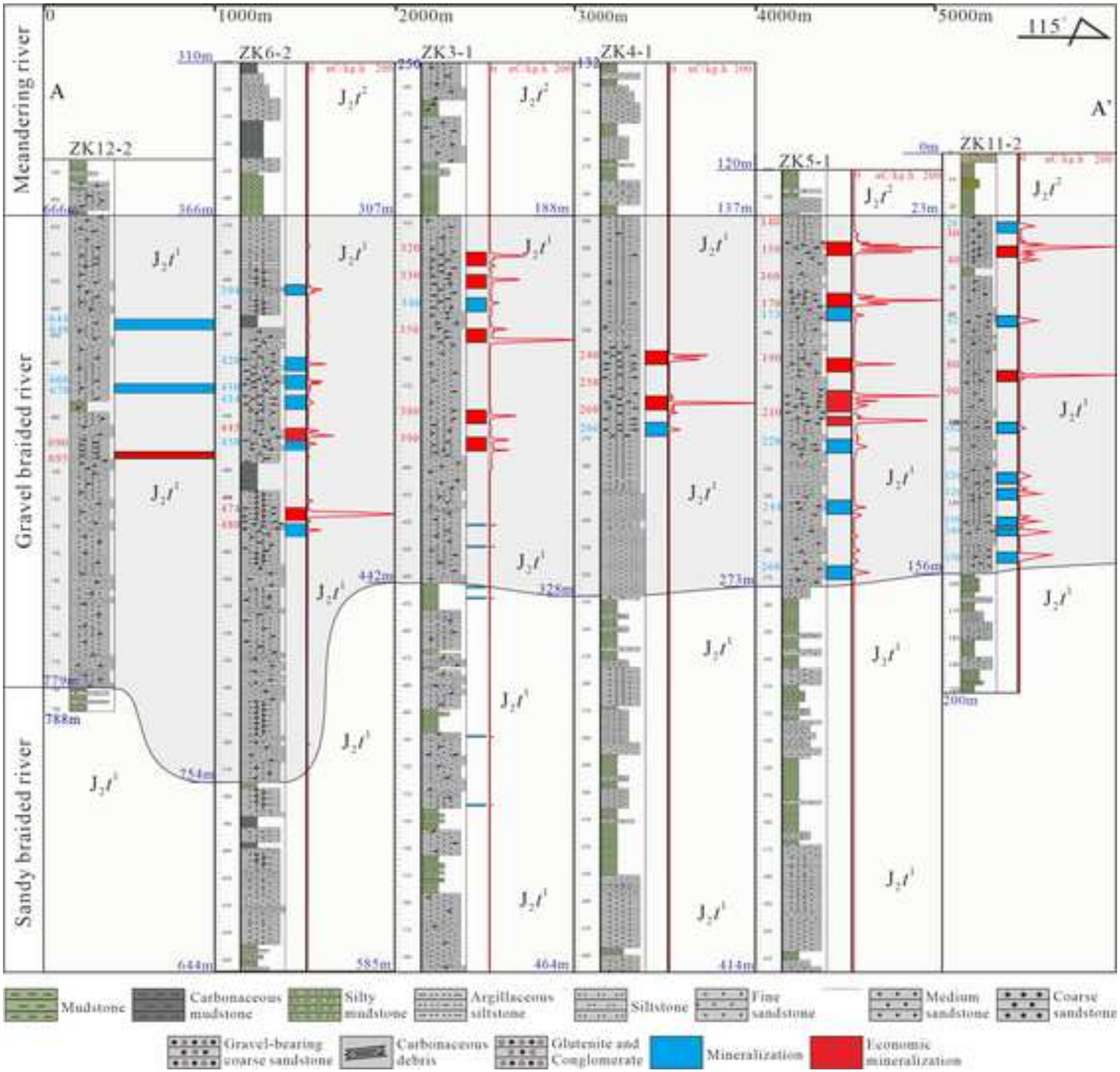

Click here to access/download;Figure;S10_Fig.tif

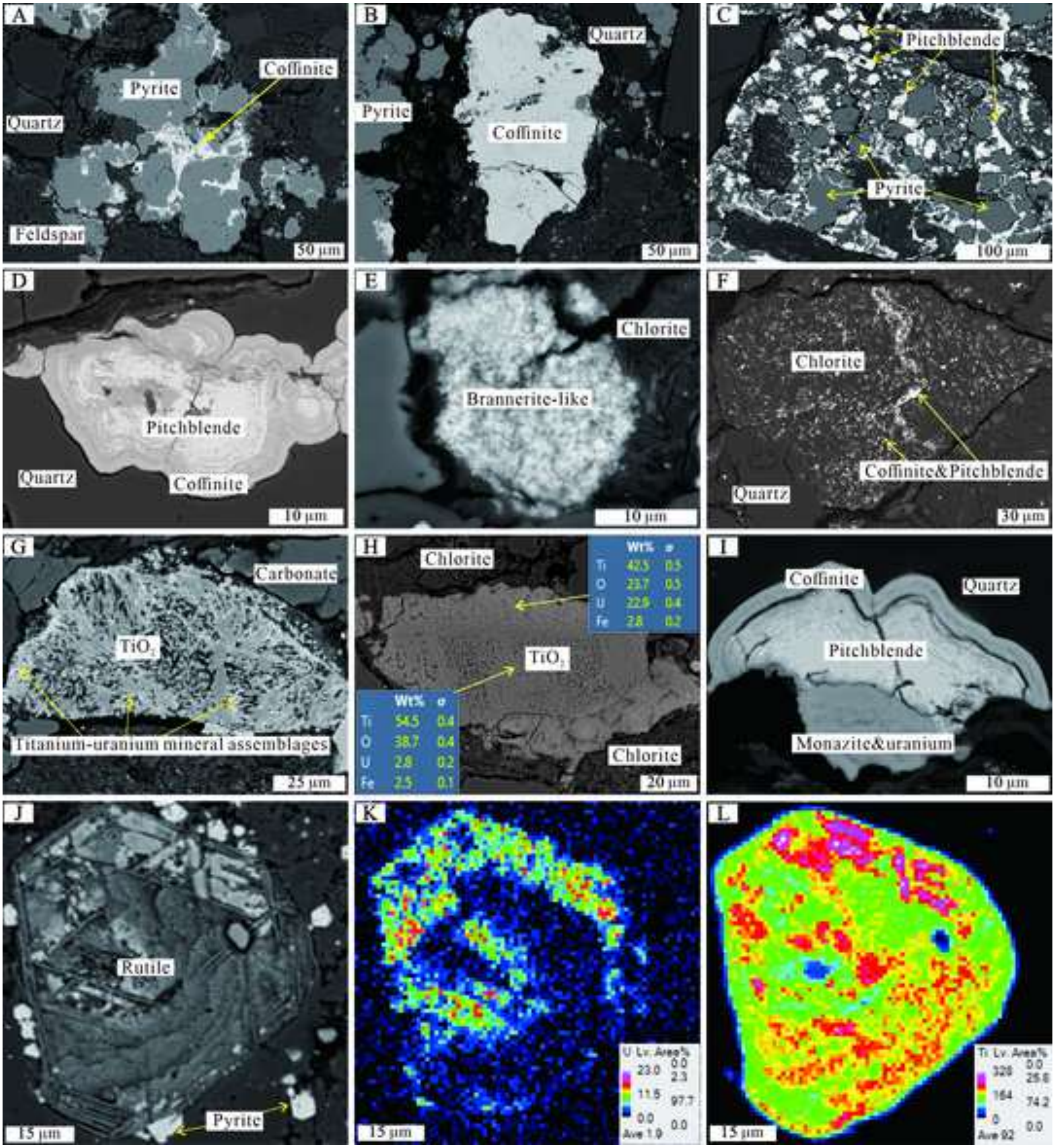

Click here to access/download;Figure;S11_Fig.tif

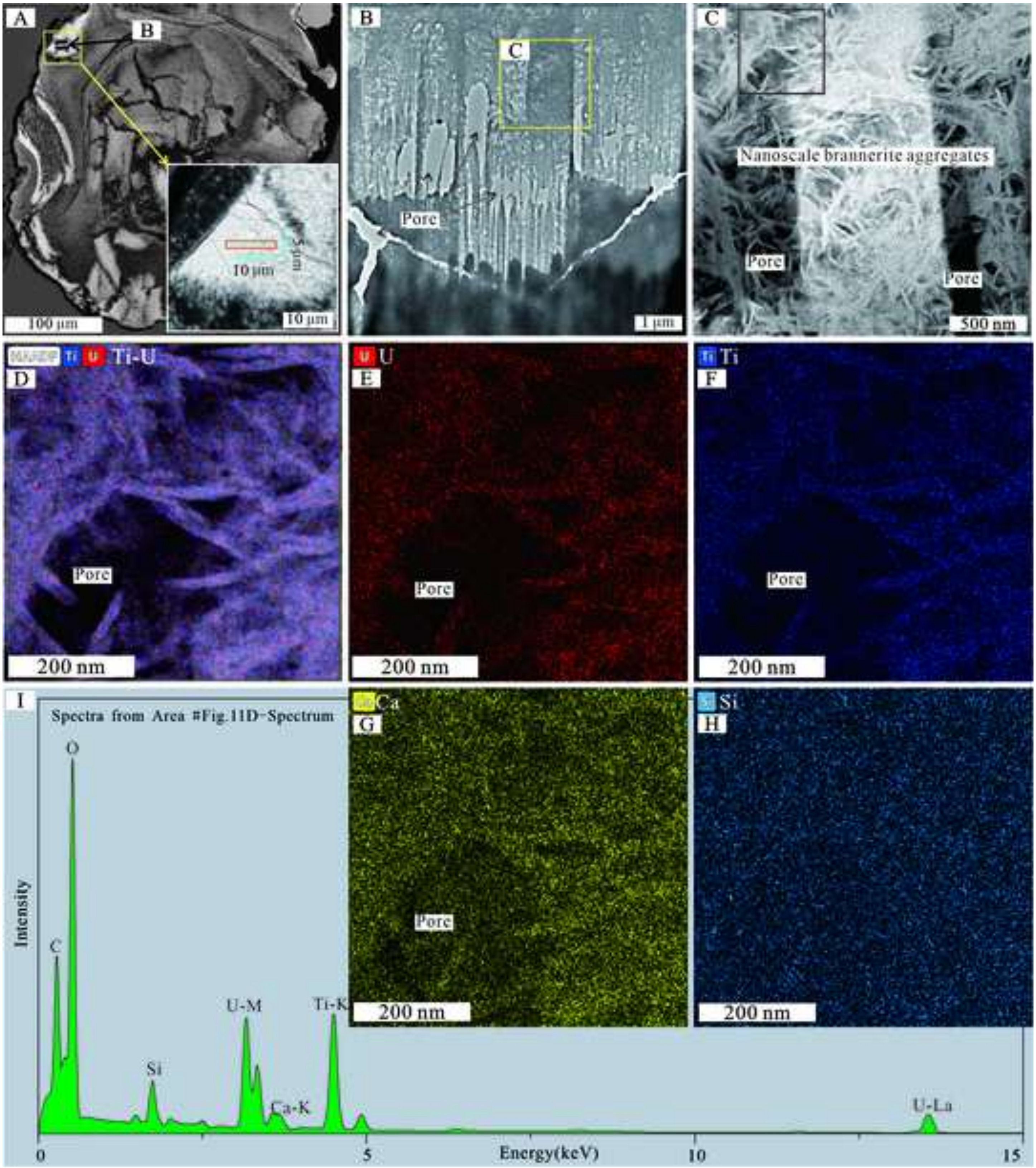

Click here to access/download;Figure;S12_Fig.tif

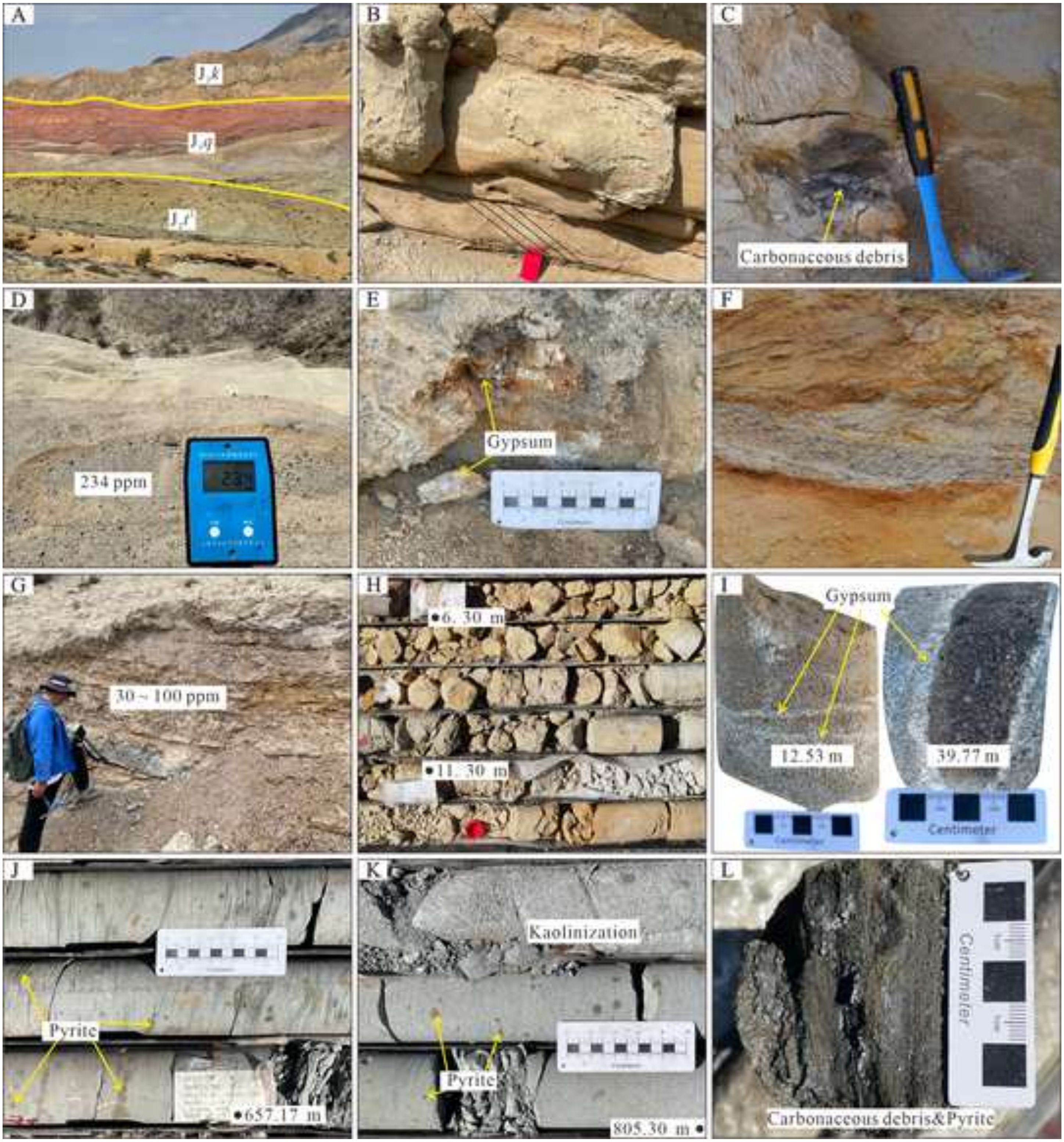

Click here to access/download;Figure;S13_Fig.tif

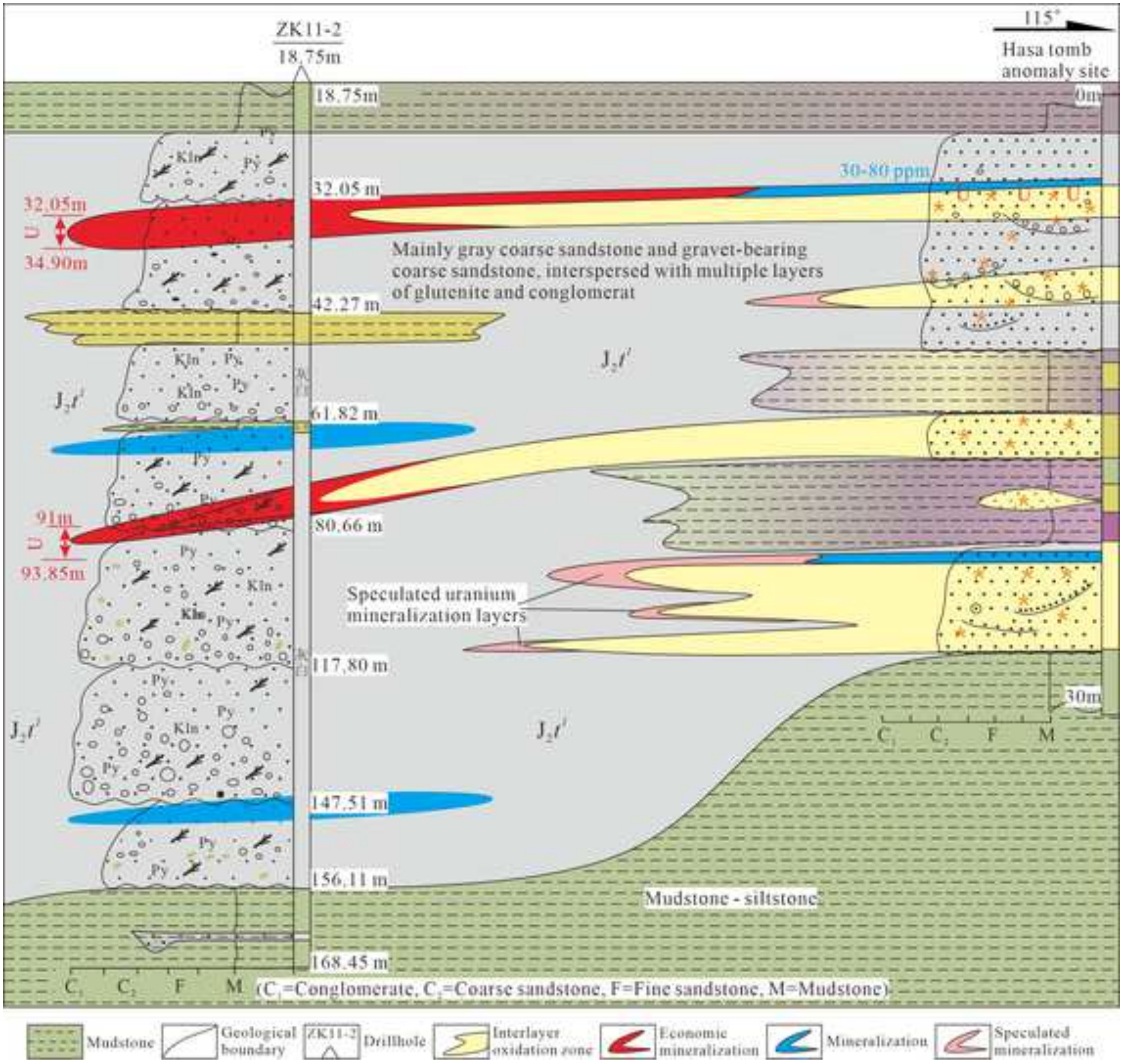

Click here to access/download;Figure;S14_Fig.tif

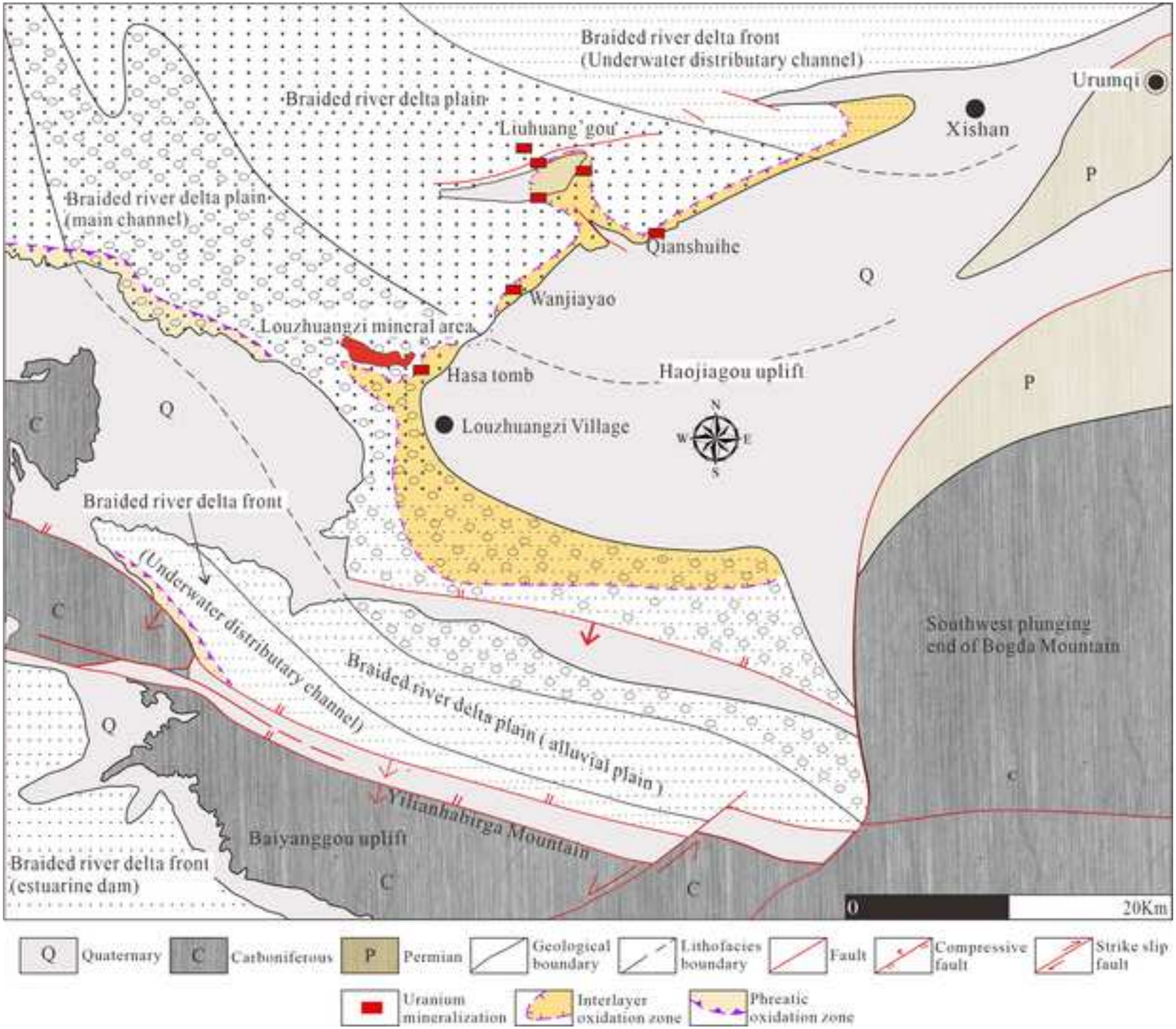

Click here to access/download;Figure;S15_Fig.tif

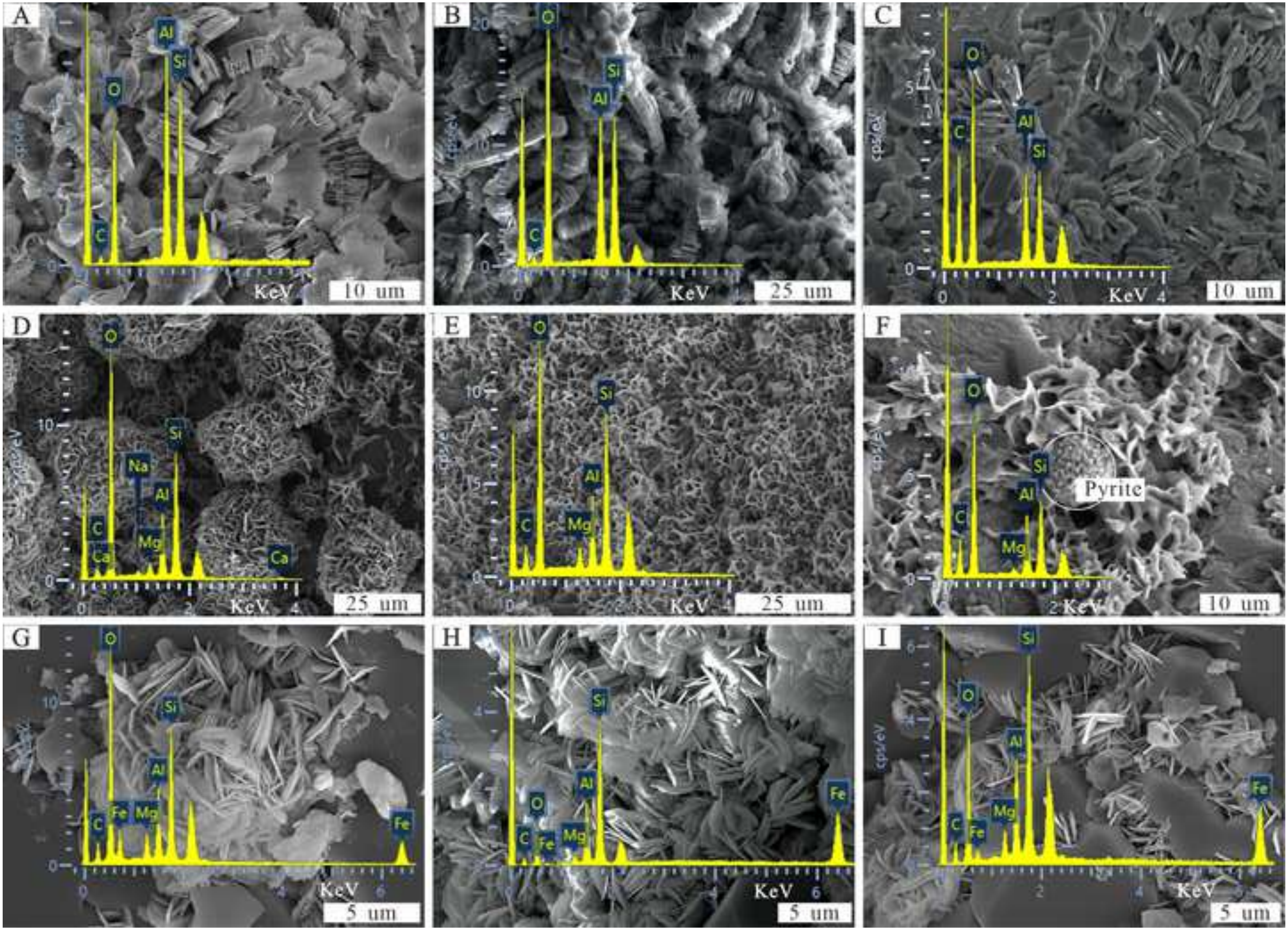

Click here to access/download;Figure;S16_Fig.tif

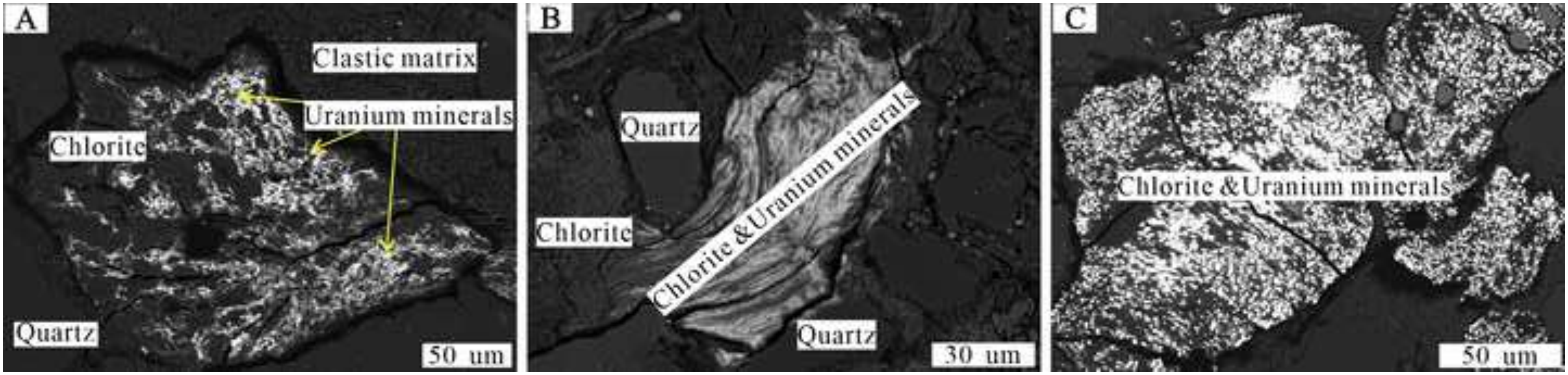

Click here to access/download;Figure;S17_Fig.tif

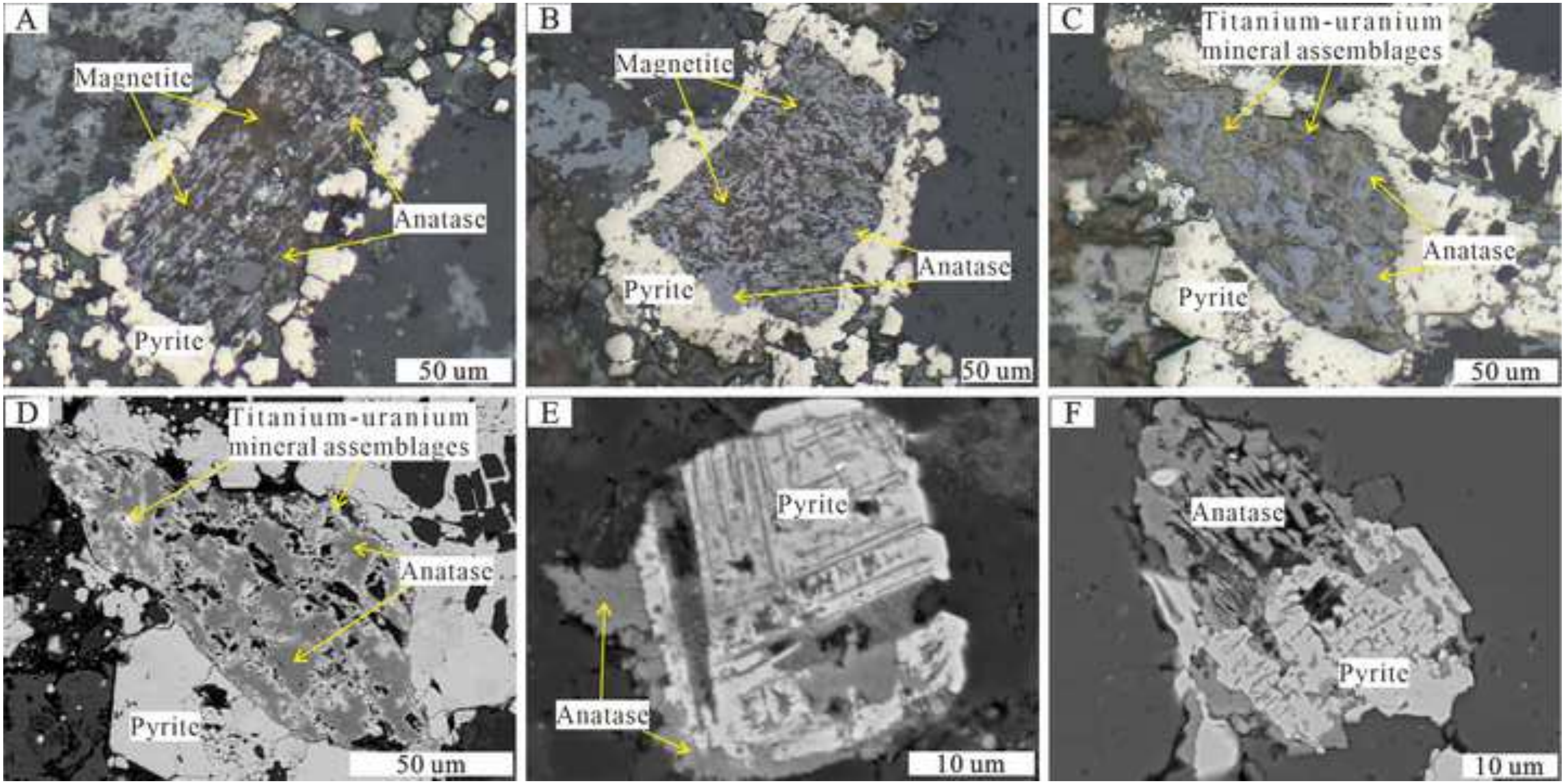

Click here to access/download;Figure;S18_Fig.tif

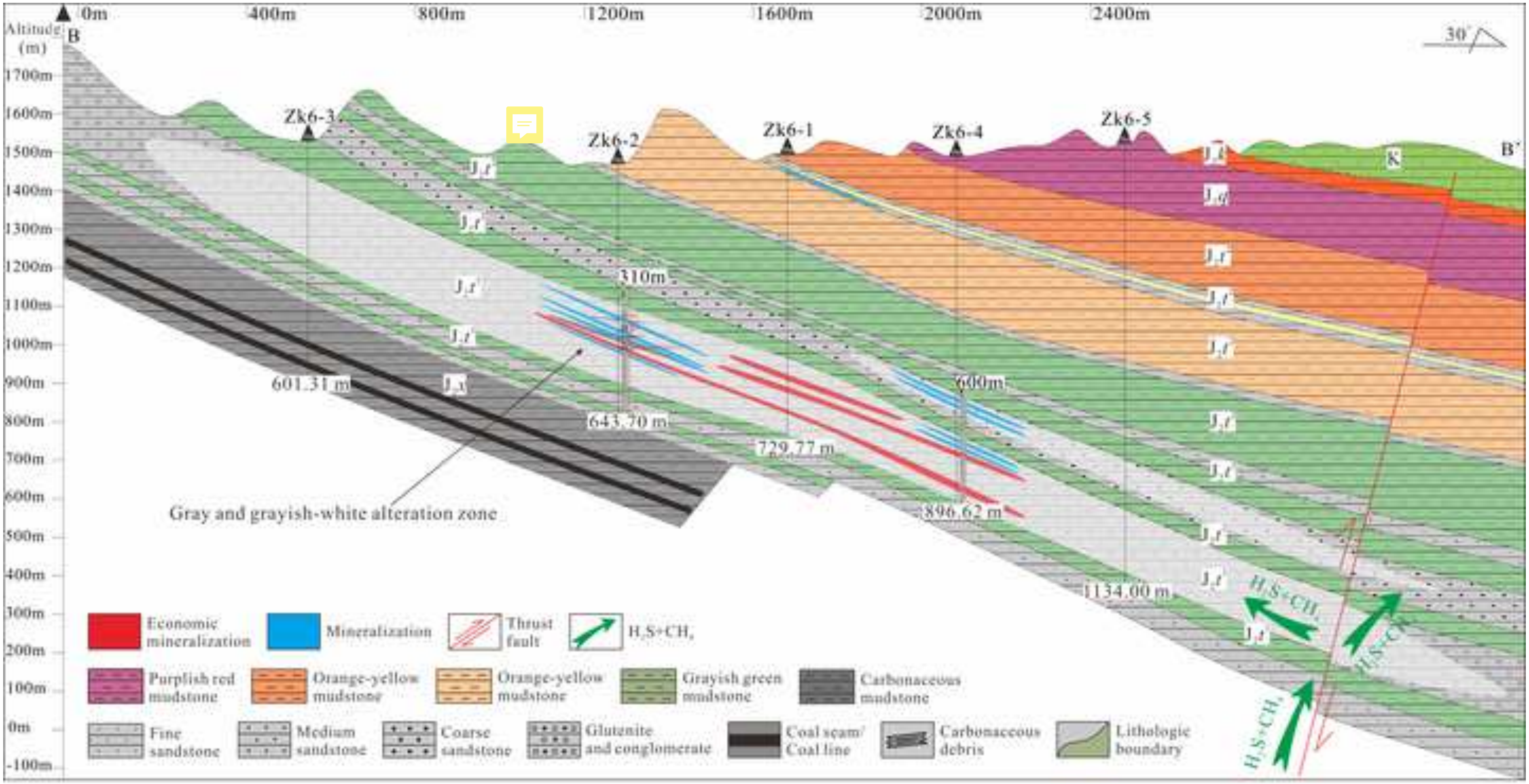

Click here to access/download;Figure;S19_Fig.tif

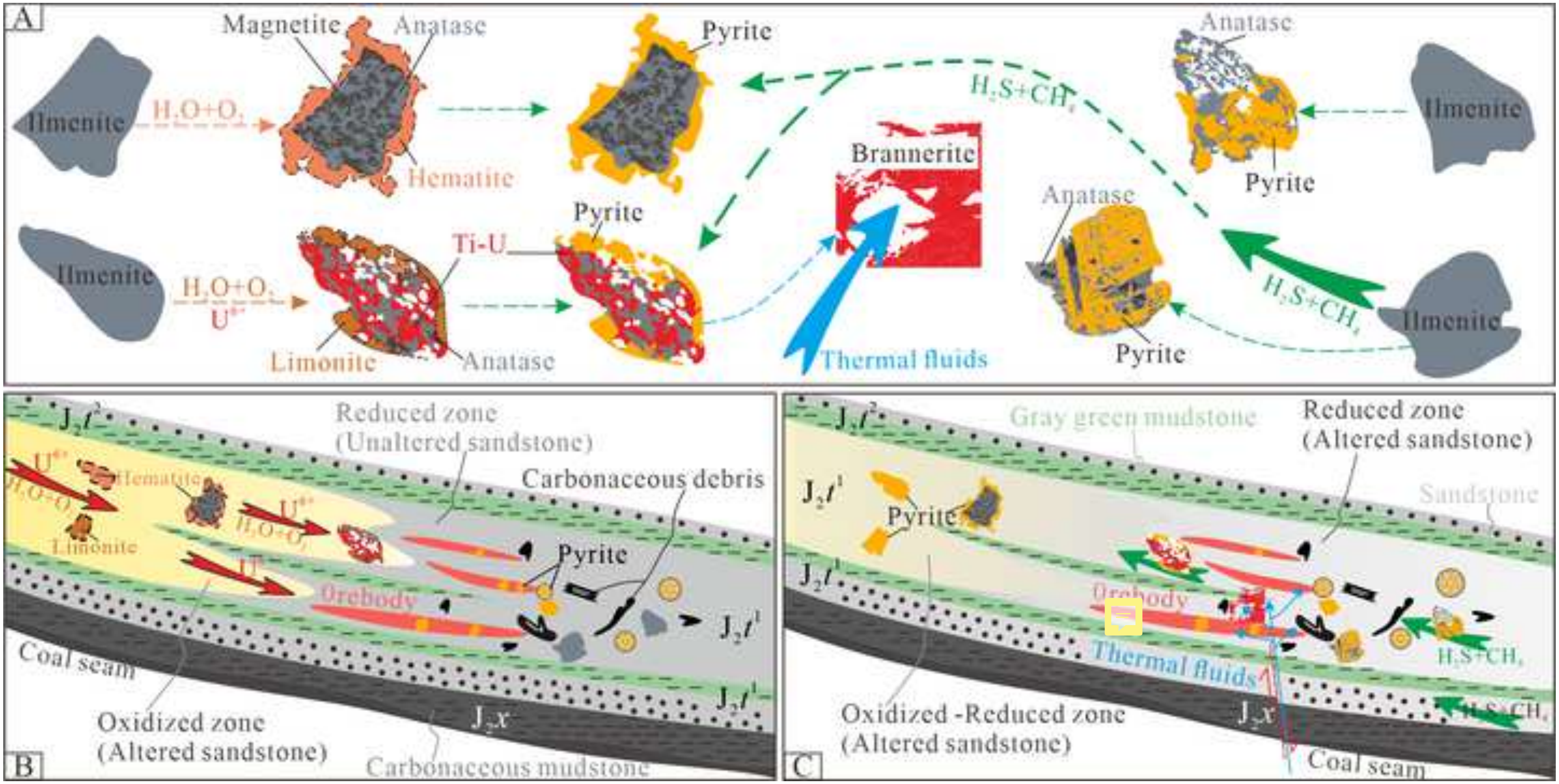

Click here to access/download;Figure;Field and indoor analysis images.tif

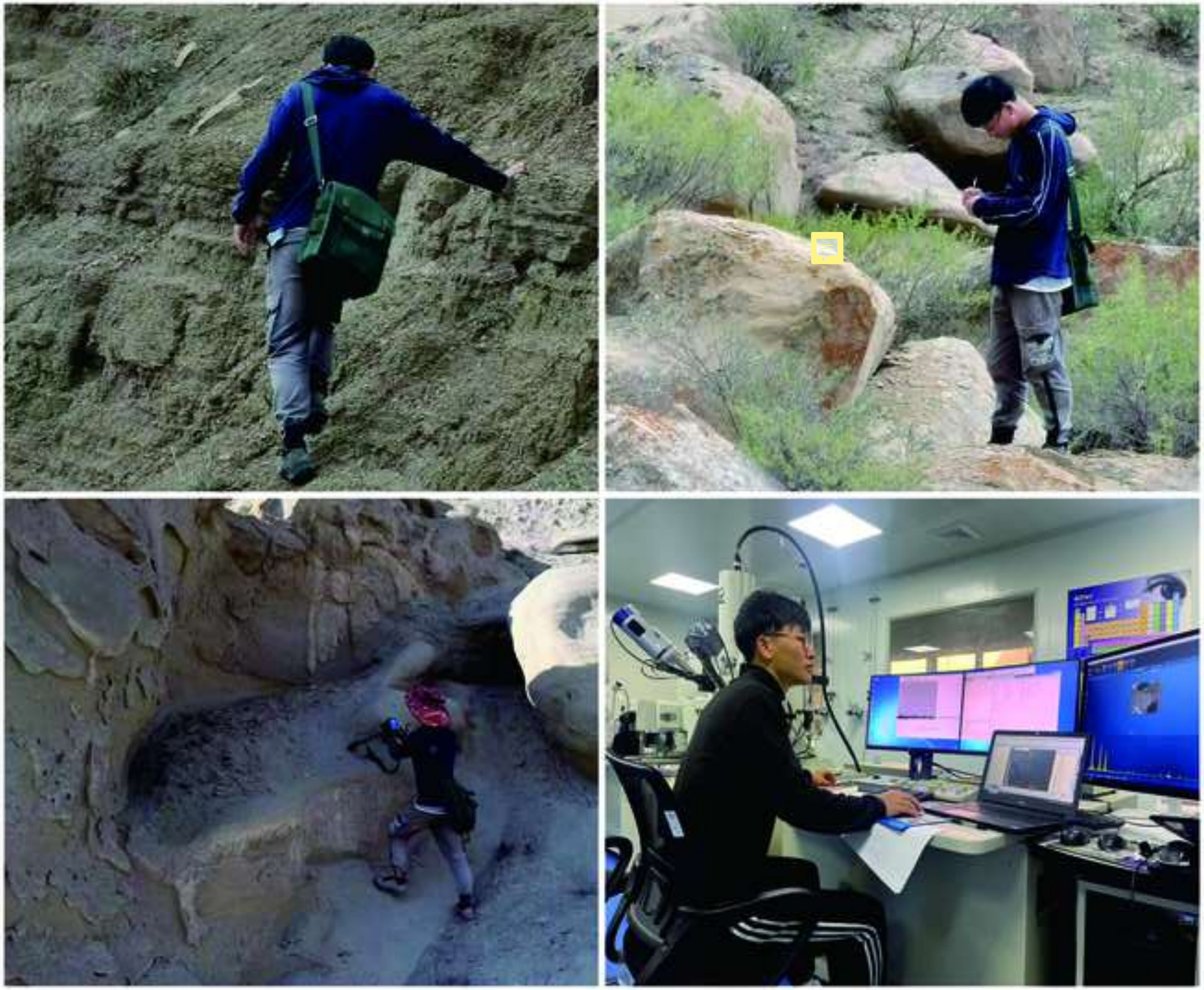

Figure

Click here to access/download;Figure;WQ-Figure-PLOS ONE.docx

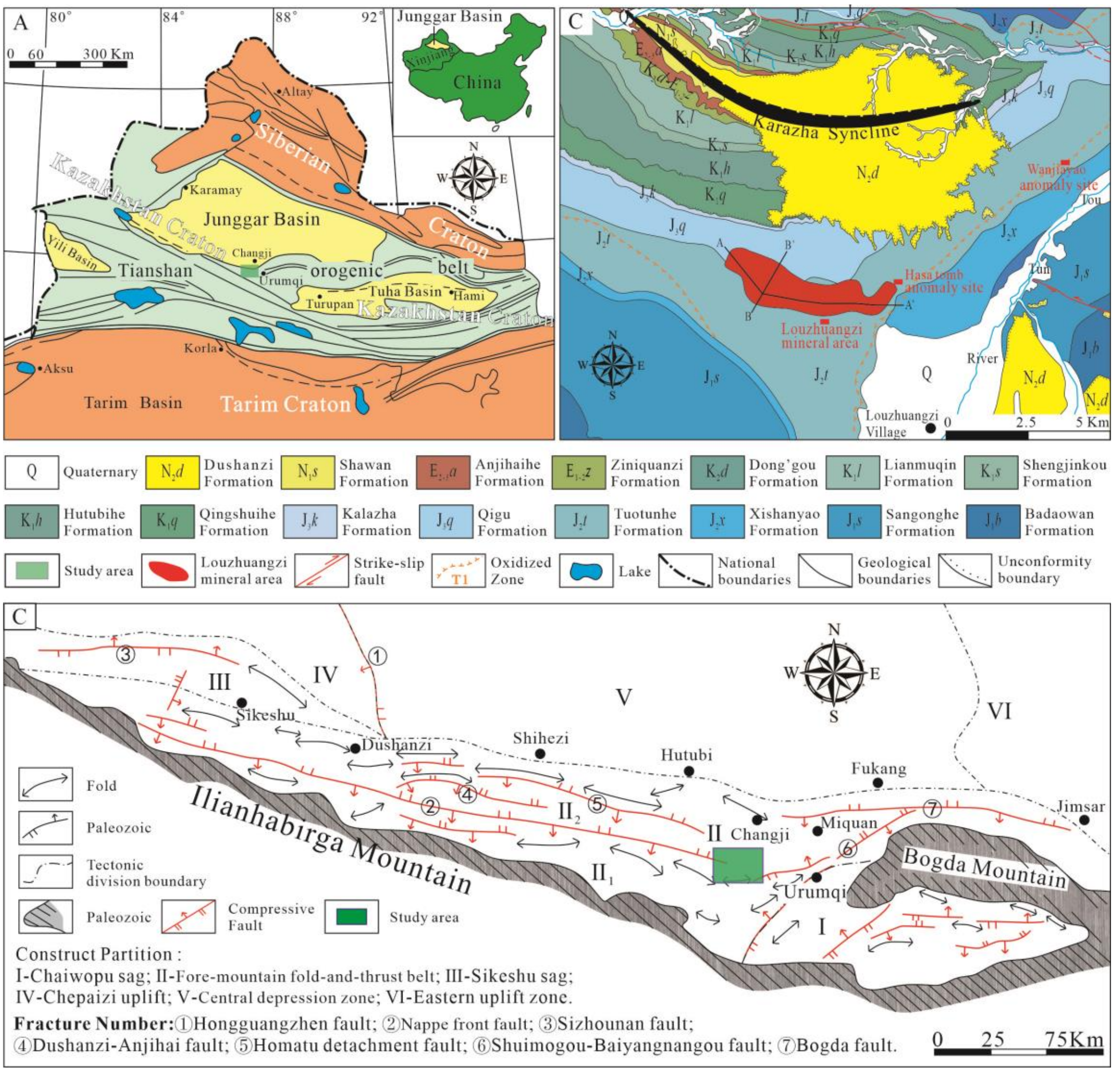

Fig.1 (A) Sketch map showing tectonic location of Junggar Basin (modified from [38]); (B) Geological sketch map of Louzhuangzi area (modified from [15]); (C) Division and distribution of the main tectonic units at southern margin of Junggar Basin (modified from [12]).

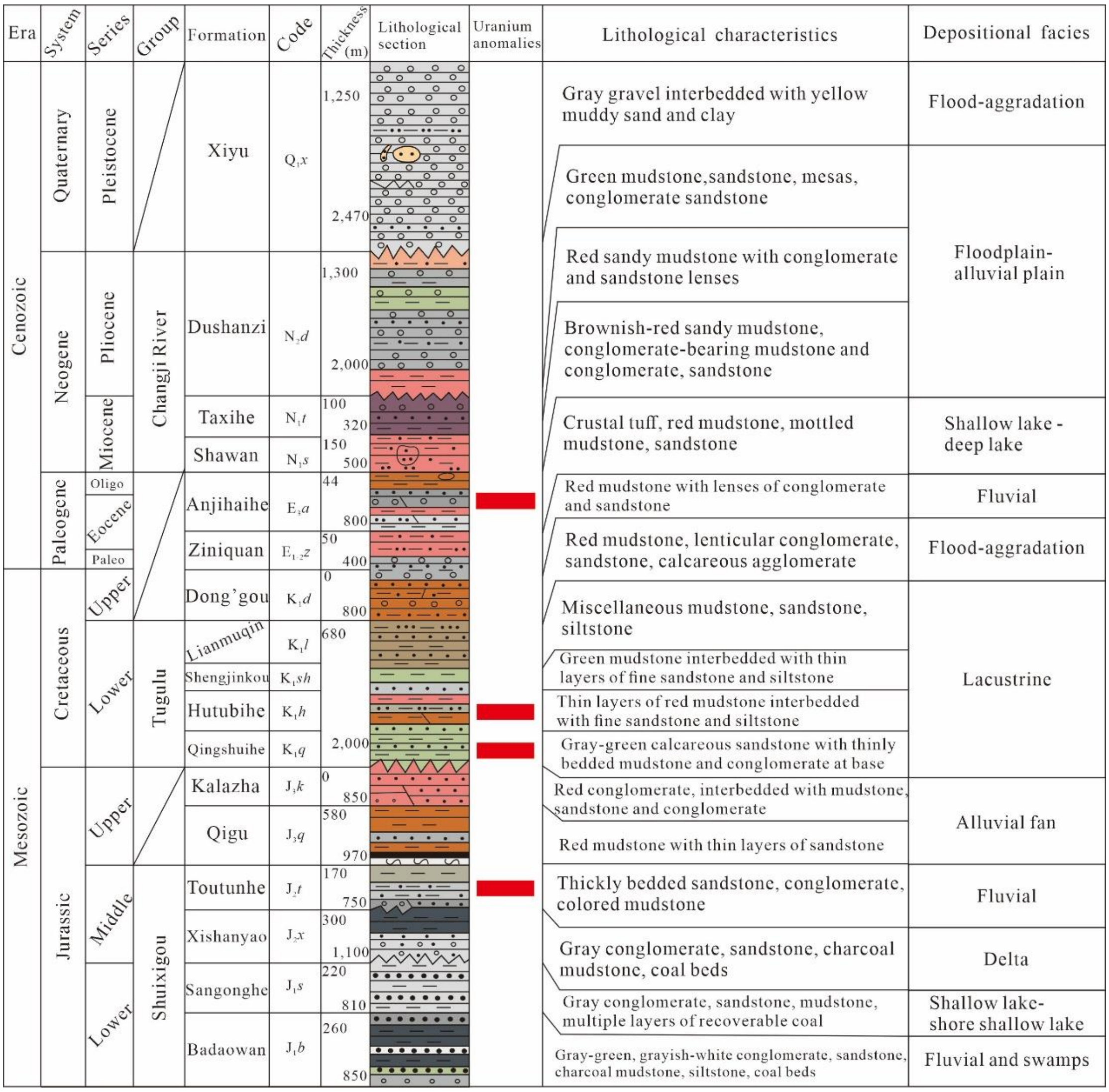

Fig. 2 Stratigraphic column of the Louzhuangzi area (modified after an internal report from Geological Party No. 216, CNNC, 2022).

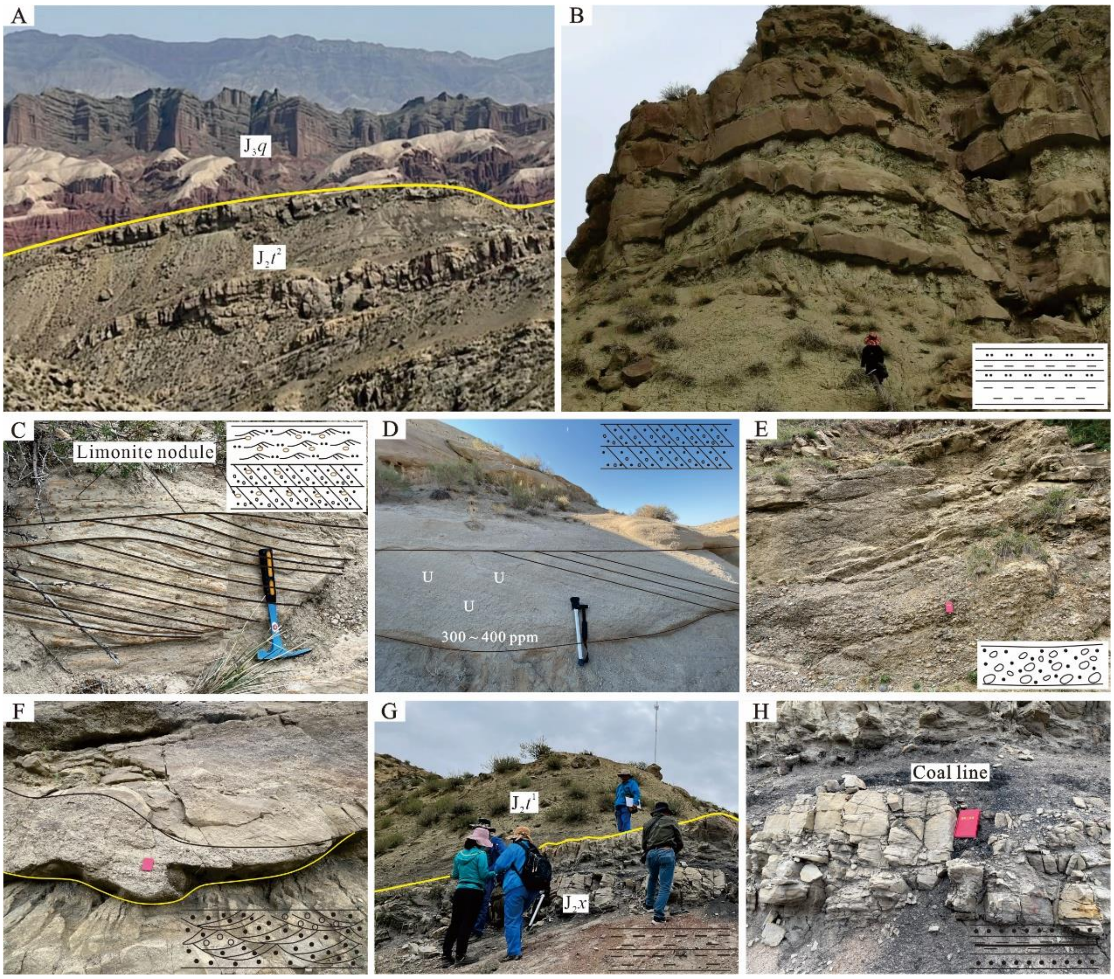

Fig. 3 Petrographic photographs of Toutunhe Formation (J_2t) outcrops in Louzhuangzi area: (A-C) Sandstone outcrop of the Upper Member, Toutunhe Formation (J_2t^2), showing fine-medium sandstone with parallel beddings (A), mudstone with massive beddings (B), fine-medium sandstone with horizontal beddings (B), and fine sandstone with flaser beddings and planar cross beddings (C); (D-F) Sandstone outcrop of the Lower Member, Toutunhe Formation (J_2t^1), showing sandstone with large to giant planar cross beddings (D), conglomerate with massive beddings (E), and glutenite and coarse sandstone with trough cross beddings (F); (G) Interface between the Lower Member of the Toutunhe Formation (J_2t^1) and the Xishanyao Formation (J_2x); (H) Sandstone outcrop of the Xishanyao Formation (J_2x), showing siltstone with parallel beddings and coal lines.

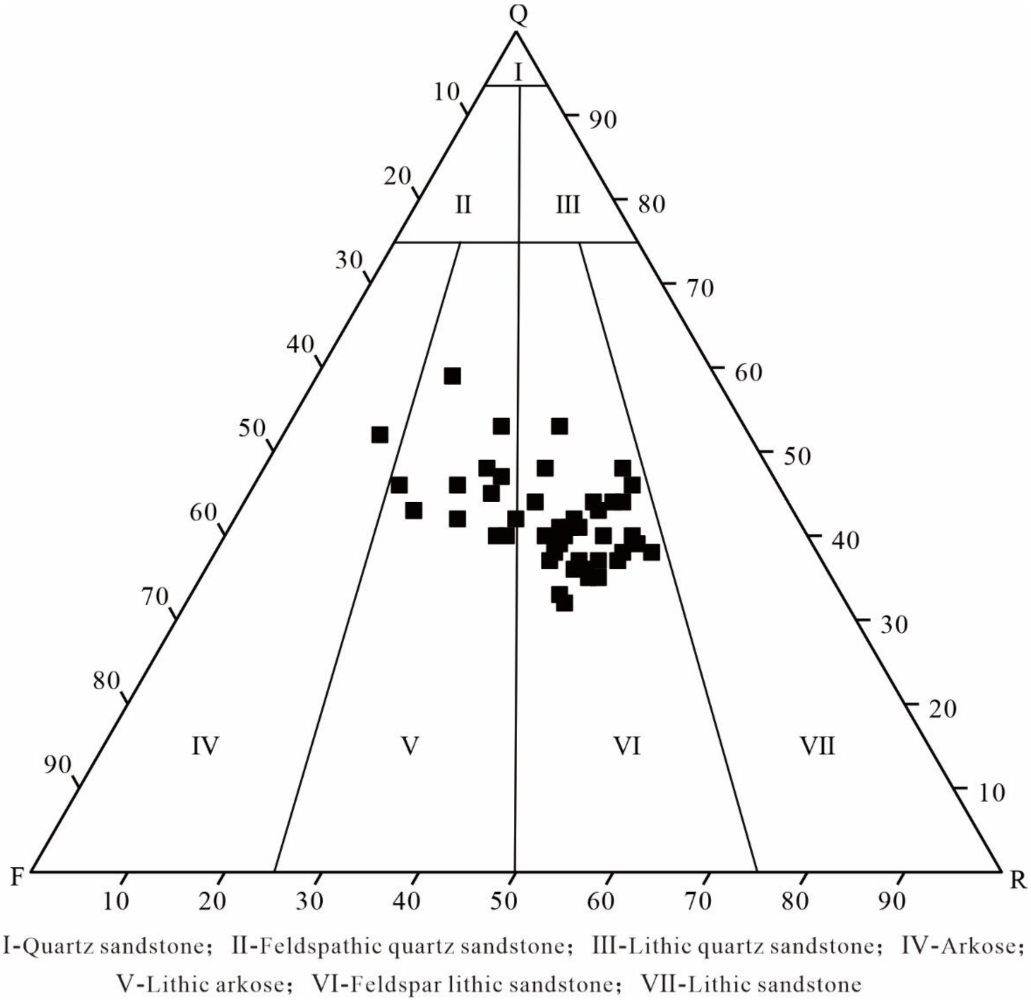

Fig. 4 Classification diagram of sandstones in Toutunhe Formation (J_2t), Louzhuangzi area (According to [41]).

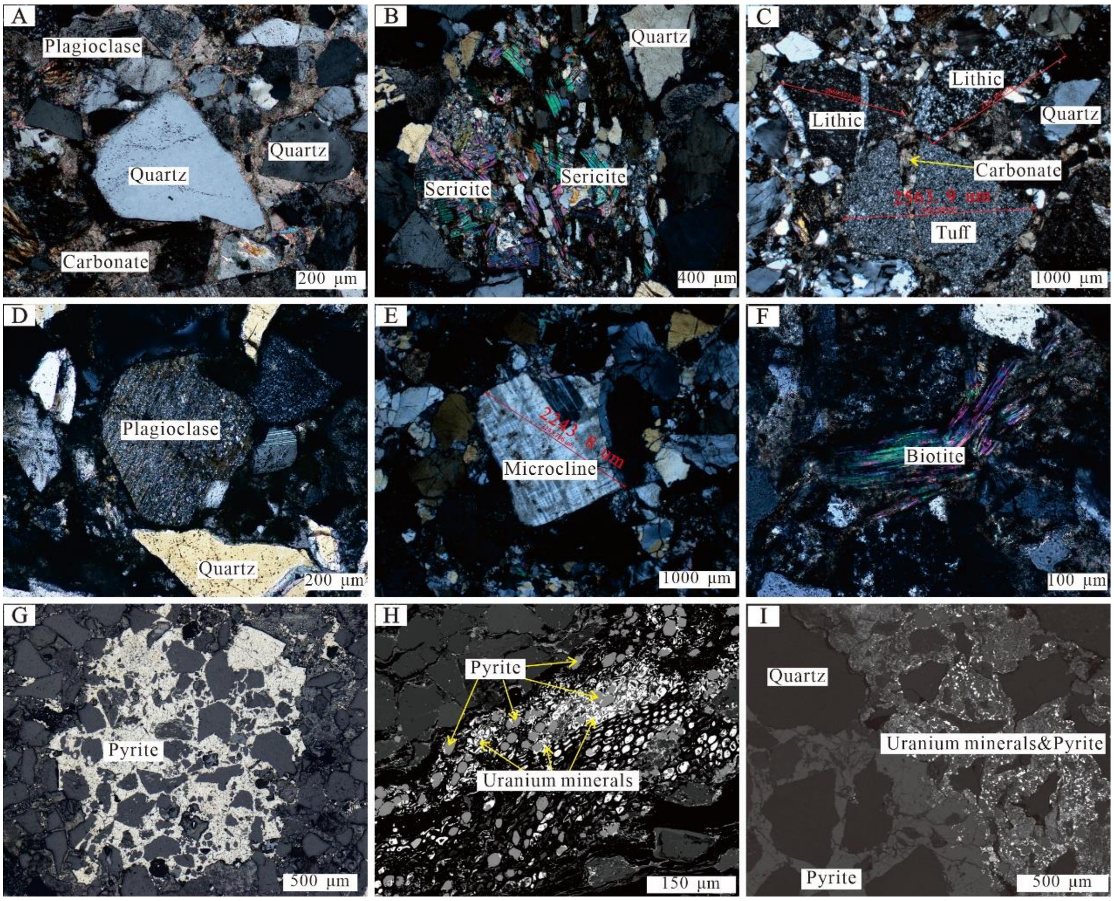

Fig. 5 Petrographic characteristics of sandstone samples from Toutunhe Formation (J_2t) in Louzhuangzi area under microscope: (A) Sharp-edged quartz clasts cemented by basal carbonate cementation, ZK5-1, 22ZGE033, 148.50 m, (+); (B) Clastics and clay matrix, ZK6-2, 22ZGE016, 477.30m, (+); (C) Pink crystalline quartz and feldspar matrix filled among debris particles, ZK6-2, 22ZGE016, 477.30m, (+); (D) Illitizated plagioclase with relatively dirty surface, ZK6-2, 22ZGE063, 478.60m, (+); (E) Lattice twin crystal microcline, ZK6-2, 22ZGE014, 483.47m, (+); (F) Bending and crushing of serimicized biotite, ZK12-2, 22ZGE001, 672.48m, (+); (G) Pyrite cementation, ZK4-2, 23ZGE026, 398.20m (reflected light); (H) Fine-grained pyrite and uranium minerals filled the cell cavities of carbonaceous debris, ZK4-2, 23ZGE026, 398.20 m, (BSE image); (I) Pyrite and uranium minerals filled among the clastic particles, ZK12-3, 23ZGE028, 798.50 m, (BSE image).

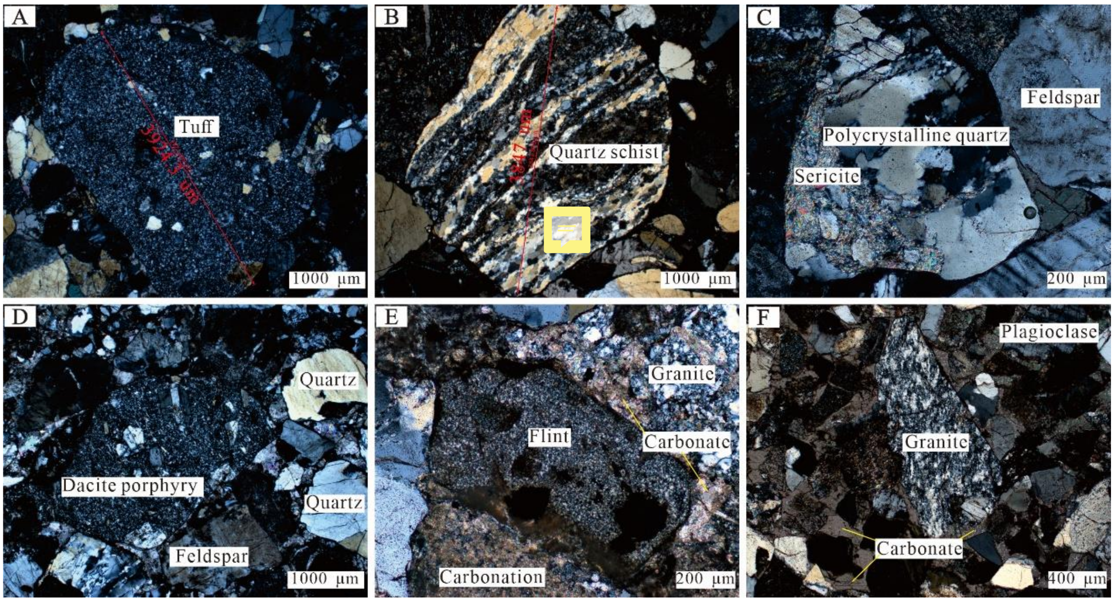

Fig. 6 Debris characteristics of sandstone samples from Toutunhe Formation (J_2t) in Louzhuangzi area under microscope: (A) Cryptocrystalline tuff debris, ZK6-2, 22ZGE016, 477.30 m, (+); (B) Platy-structure quartz schist debris, ZK6-2, 22ZGE015, 479.30m, (+); (C) Sericitization of sharp edged polycrystalline quartz, ZK6-2, 22ZGE016, 477.30m, (+); (D) Dacite porphyry debris with porphyritic texture, ZK6-2, 22ZGE026, 398.80m, (+); (E) Dissolution pit visible in cryptocrystalline flint debris, ZK6-2, 22ZGE026, 398.80m, (+); (F) Carbonate-cemented granite debris, ZK11-2, 22ZGE034, 24.84 m, (+).

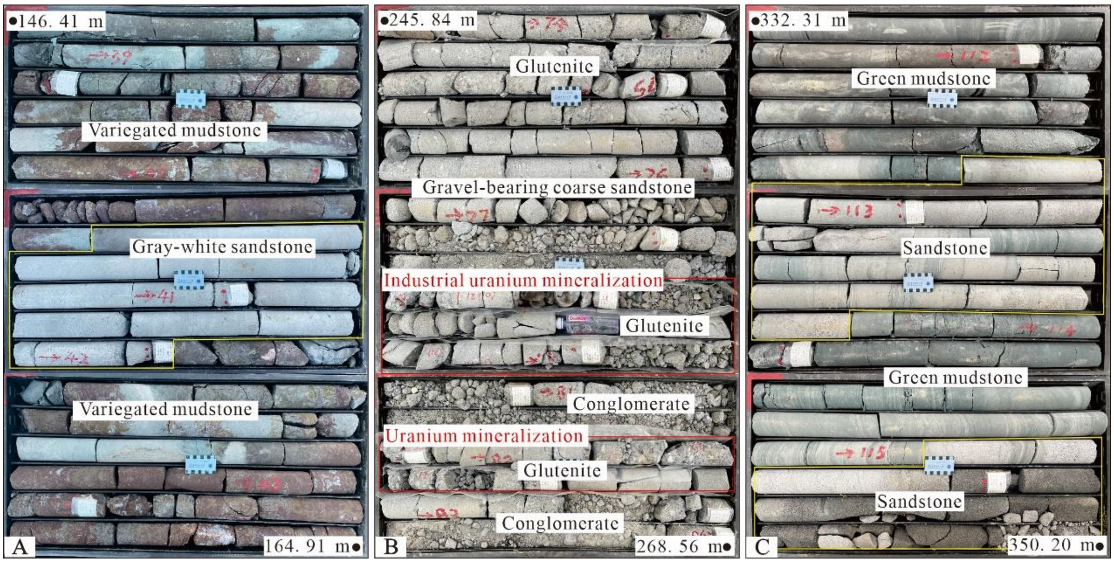

Fig. 7 Photographs of representative core samples from Toutunhe Formation (J_2t) exposed by the economic uranium mineralization drill hole of ZK5-7 in Louzhuangzi area: (A) Meandering river facies ' thick mud with thin sand from the Upper Member of the Toutunhe Formation (J_2t^2); (B) Gravelly braided river facies thick sand body from the Lower Member of the Toutunhe Formation (J_2t^1); (C) Sandy braided river facies flood mud and crevasse splay sand from the bottom of the Lower Member of the Toutunhe Formation (J_2t^1) .

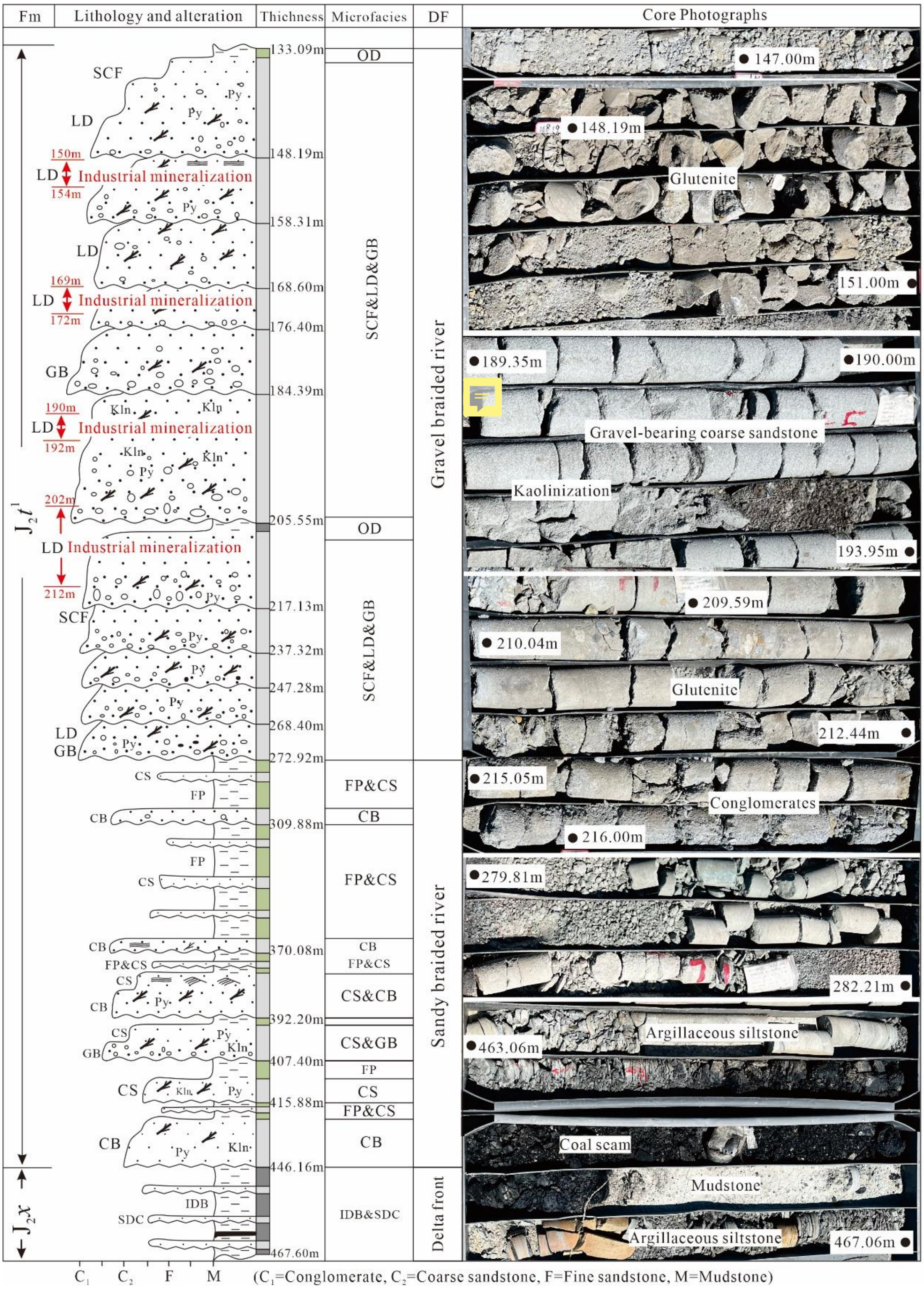

Fig. 8 Interpretation of the core profile of drill hole ZK5-1 in Louzhuangzi area. (Abbreviation: Fm=Formation; DF= Depositional Facies; OD= Overbank Deposit; SCF= Sandy Channel Fill; LD=

Lag Deposit; GB= Gravel Bar; FP= Floodplain; CS= Crevasse Splay; CB= Channel Bar; IDB= Interdistributary Bay; SDC= Subaqueous Distributary Channel).

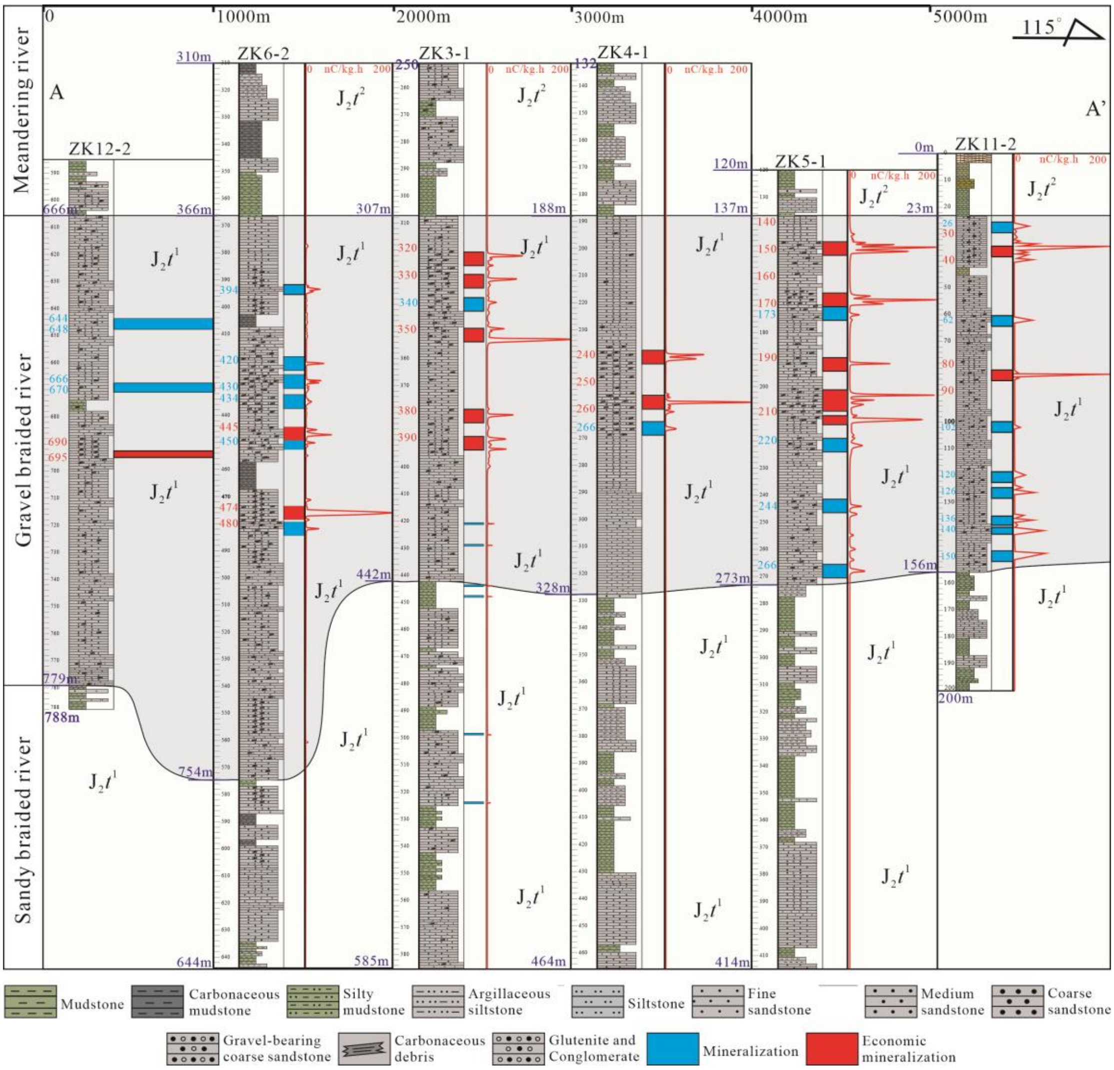

Fig. 9 Sketch diagram showing the A-A' profile in Louzhuangzi area (In the Lower Member of the Toutunhe Formation (J_2t^1), the top roof of the sandstone has been leveled. The profile position is shown in Figure 1C).

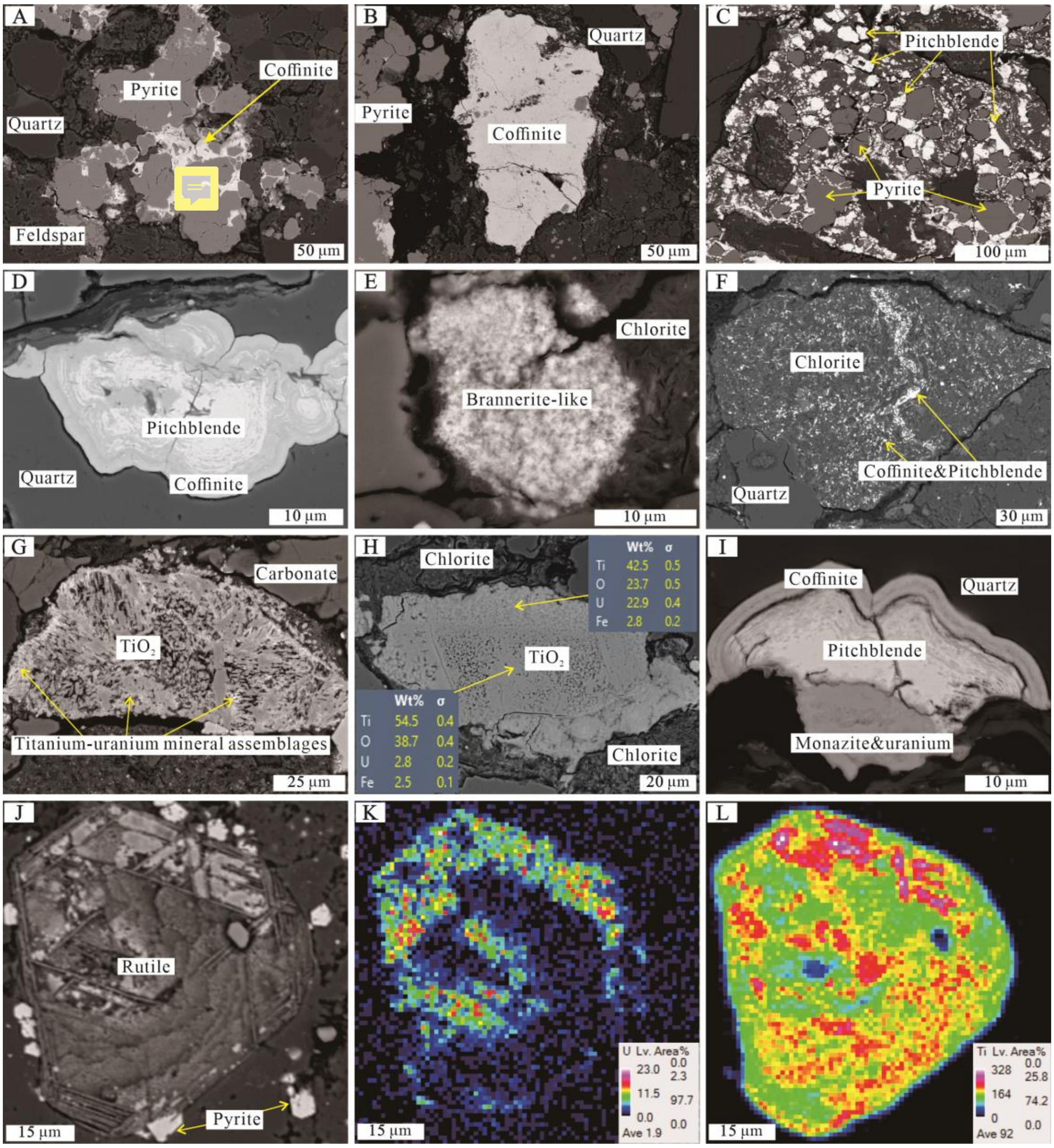

Fig. 10 Occurrence characteristics of uranium in sandstones of Toutunhe Formation (J_2t) in Louzhuangzi area (BSE images), including (A) Gelatinous coffinite associated with pyrite, ZK11-3, 23ZGE022, 119.52m; (B) Massive coffinite associated with fine-grained pyrite, ZK12-3, 23ZGE028, 798.50m; (C) Massive pitchblende associated with pyrite, ZK12-3, 23ZGE028, 798.50 m; (D) Baited pitchblende coexisted with coffinite with ring-shaped distribution; ZK4-2, 23ZGE026, 398.20 m; (E) Nanoscale titanium-uranium aggregates associated with chlorite, ZK3-3, 23ZGE027, 753.10m; (F) Nanoscale uranium particles adsorbed in clay minerals, ZK12-3, 23ZGE028, 798.50m;

(G) Nanoscale uranium minerals adsorbed at the edge of titanium oxides, ZK4-2, 23ZGe026, 398.20m; (H) Nanoscale uranium minerals adsorbed in the dissolution pores of titanium oxides, ZK12-3, 23ZGE028, 798.50 m; (I) Uranium-rich monazite associated with baited pitchblende, ZK4-2, 23ZGE026, 398.20m; (J) Uranium-rich rutile, ZK4-2, 23ZGE026, 398.20 m; (K-L) Area distribution of U(K) and Ti (L) elements in uranium-rich rutile obtained by electron probe.

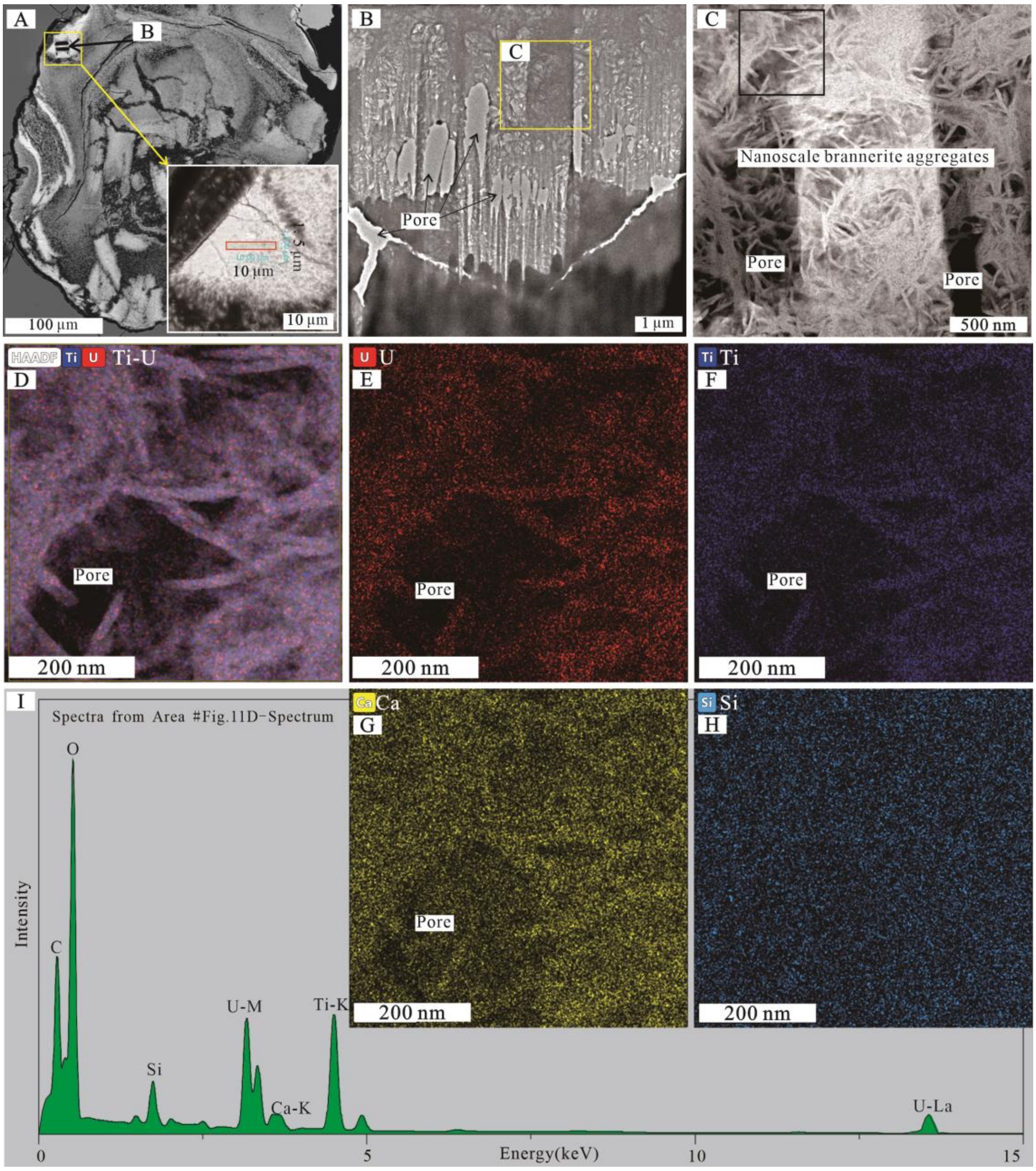

Fig. 11 Images showing the characteristics of titanium-containing uranium minerals under FIB and TEM, including (A) Backscattered electron (BSE) image of titanium-

containing uranium mineral under focused ion beam (FIB) scanning electron microscope; (B) Image of the transparent foil of titanium-containing uranium mineral under transmission electron microscope (The transparent foil was taken vertically downward at the yellow rectangular position in Figure A, with the thickness <100 nm); (C) HAADF-STEM image of titanium-containing uranium mineral; (D-I) STEM-EDS-derived area distribution of Ti-U(D), U(E), Ti(F), Ca(G) and Si(H) elements and energy spectra (I) of the black rectangular area in Figure C.

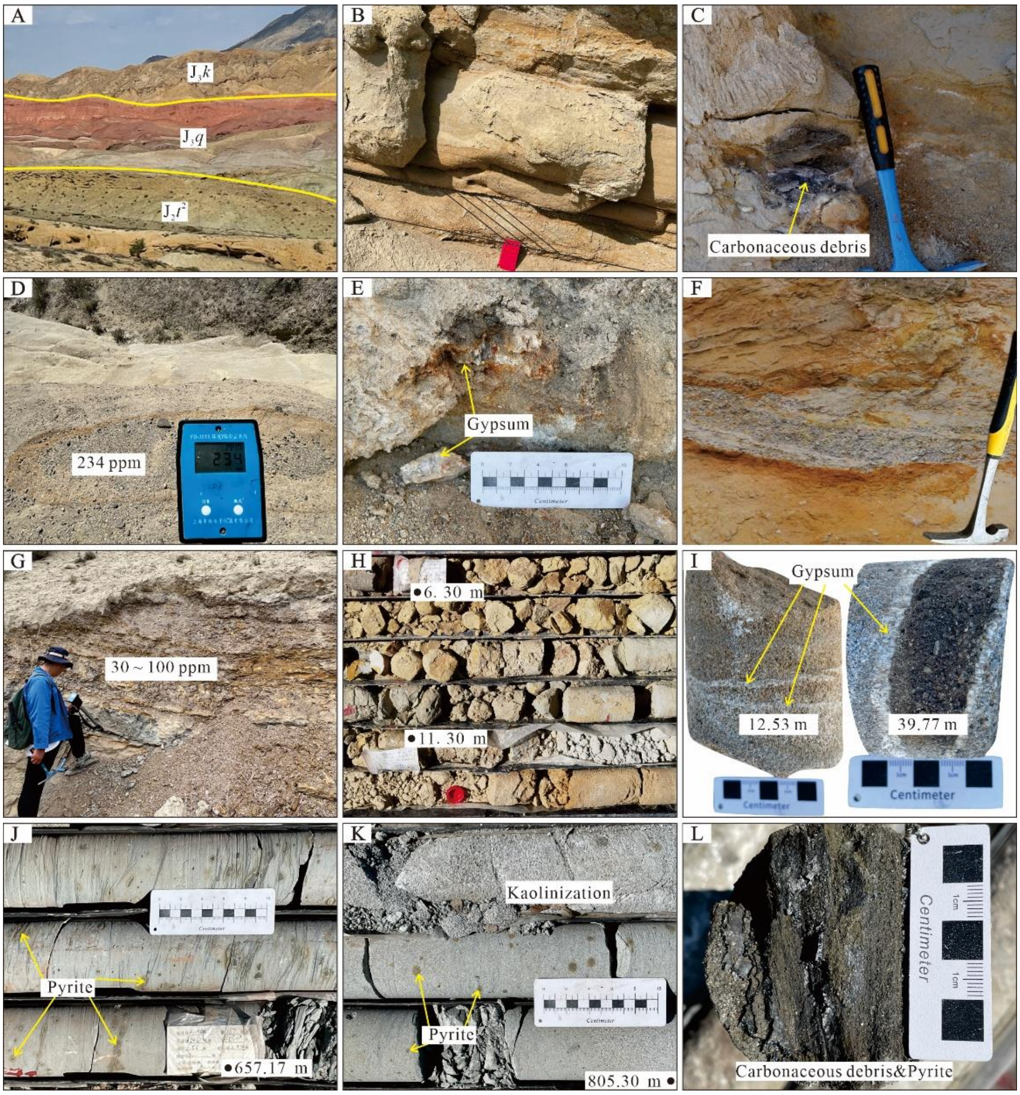

Fig. 12 Epigenetic alteration characteristics of sandstones in Toutunhe Formation (J_2t), Louzhuangzi area, including (A-D) Sand-yellow oxidized sandstone outcrop (A); medium to large planar cross bedding developed in the sandstone (B); incompletely oxidized carbonized plant detritus (C) and layered gypsum (E) occasionally observed; uranium anomaly commonly observed in gravel-bearing coarse sandstone (D) of the Upper Member of the Toutunhe Formation (J_2t^2) at Wanjiayao mineralization site; (F-G) Incompletely oxidized gray residues in the sand-yellow oxidized sandstone (F); uranium anomalies observed in the oxidized sandstone (G) of the Lower Member of the Toutunhe Formation (J_2t^1) at Hasa tomb anomaly site; (H-I) Oxidized sandstone (H) and multiple layers of gypsum (I) of the Upper Member of the Toutunhe Formation (J_2t^2) exposed by drill hole ZK11-3; (J) Laminated carbonized plant detritus and pyrite distribution observed in the grayish-white altered sandstones in the Lower Member of the Toutunhe Formation (J_2t^1) exposed by drill hole ZK 6-4; (K) Kaolin and pyrite nodules developed in the grayish-white altered sandstones in the Lower Member of the Toutunhe Formation (J_2t^1) exposed by drill hole ZK 12-3; (L) Laminated carbonized plant detritus and pyrite distribution exposed by drill hole ZK4-3 in depth of 758.24m.

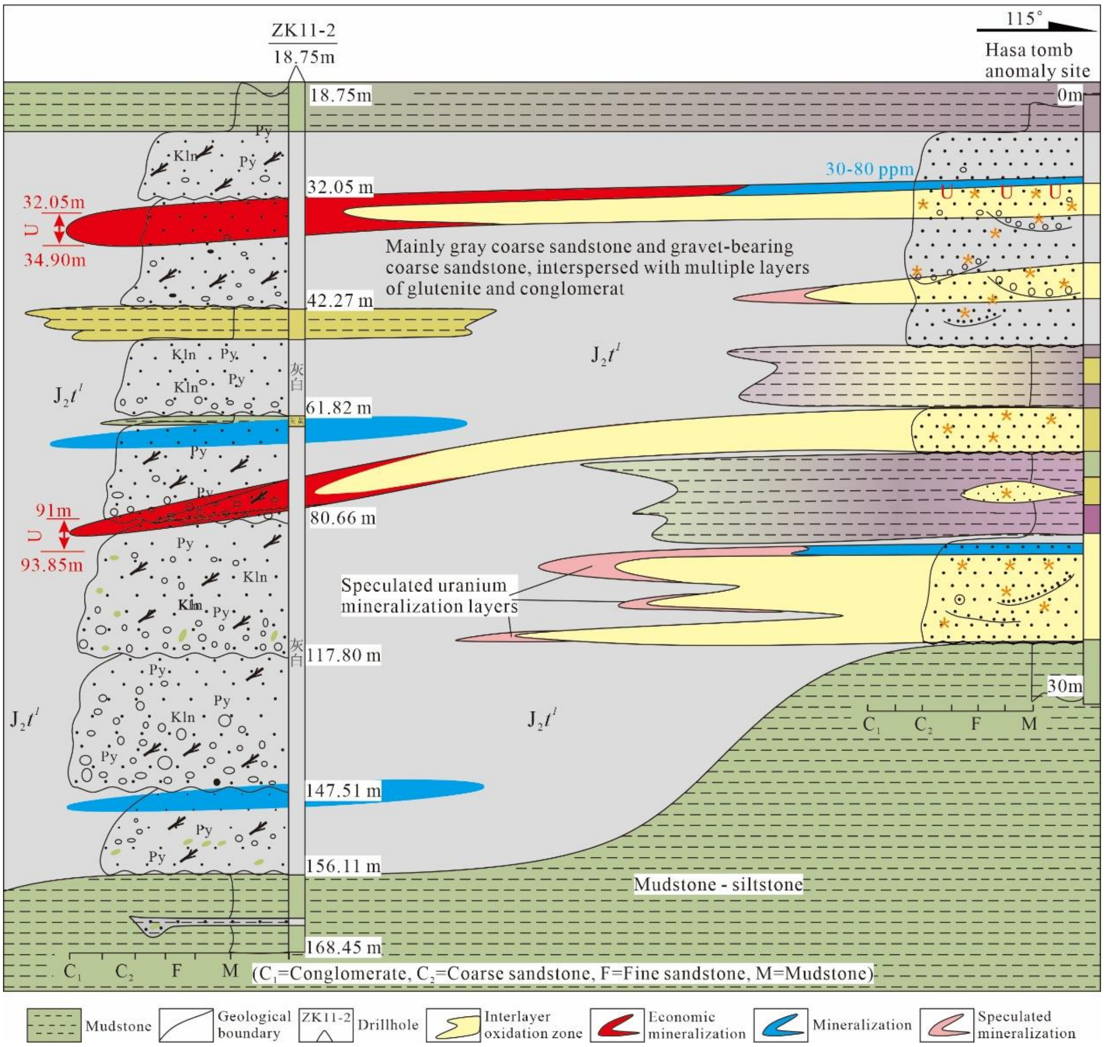

Fig. 13 Profile of interlayer oxidation zones and uranium mineralization zones in sandstones of Toutunhe Formation (J_2t) in drill hole ZK 11-2 at Hasa tomb anomaly site (with sandstone top roof being leveled).

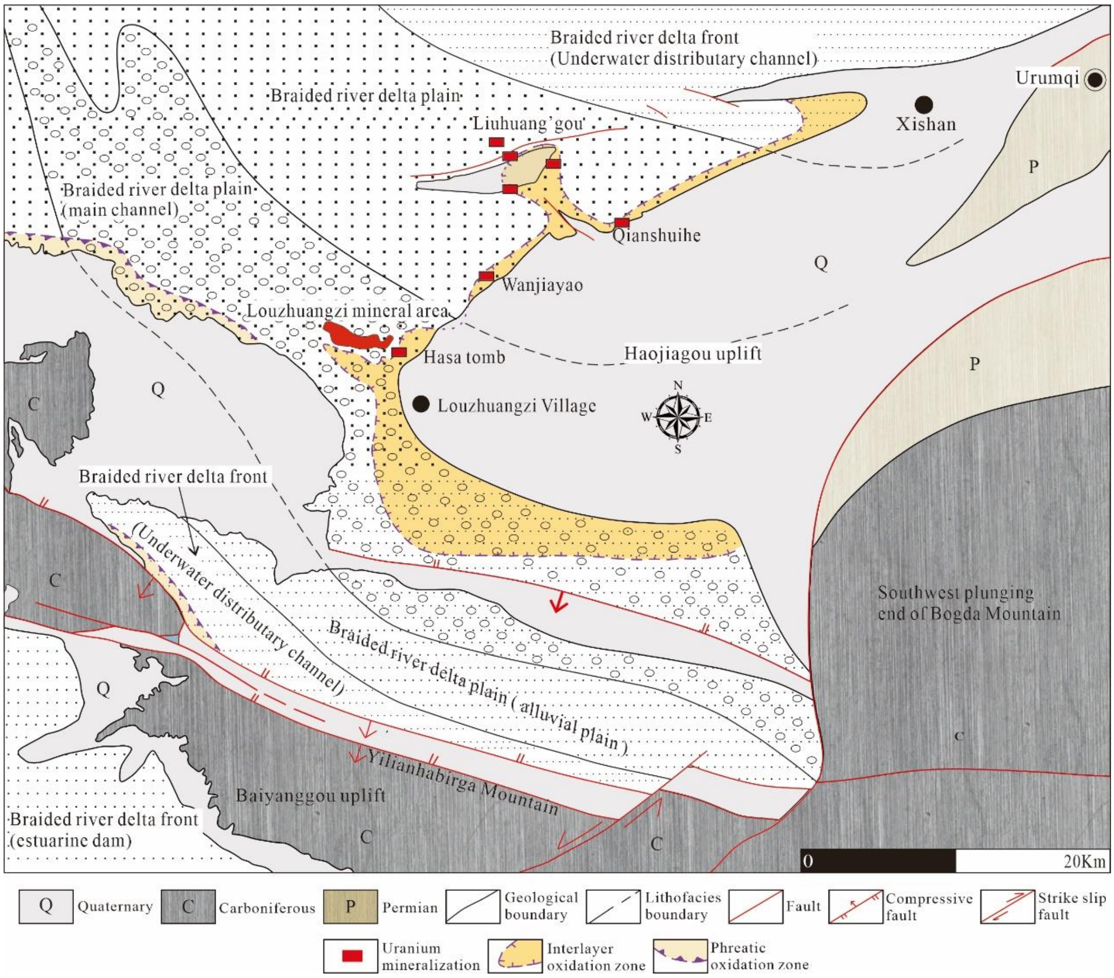

Fig. 14 Distribution of depositional facies and oxidation zones in the Lower Member of Toutunhe Formation (J_2t^1), Louzhuangzi area (modified from [13]).

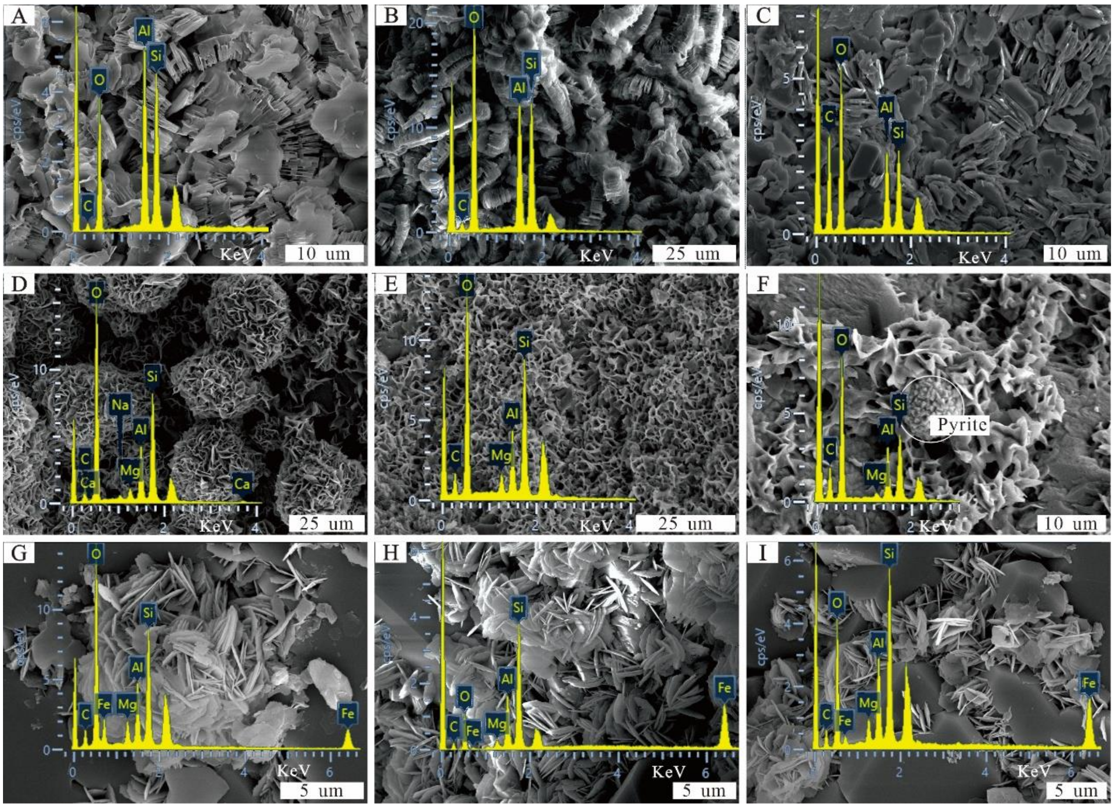

Fig. 15 Characteristics of clay minerals in sandstones in Lower Member of Toutunhe Formation (J_2t^1), Louzhuangzi area (BSE images), including (A) Intergranular layered kaolinite, ZK16-2, 23DJA-9, 705.70m; (B) Intergranular worm-like kaolinite, ZK16-2, 23 dJA-6, 654.60m; (C) Intergranular plate-like kaolinite, ZK12-3, 23 dJA-27, 641.70m; (D) Spherical smectite on the particle surface , ZK4-3, 23DJA-7, 710.40m; (E) Cellular smectite on the particle surface , ZK12-3, 23 DJA-15,871.50m; (F) The strawberry pyrite Encapsulated by flake-like smectite, ZK12-2, 23DJA-4, 680.70m; (G-I) Intergranular flake-like chlorite aggregate, ZK16-2, 23DJA-9, 705.70m.

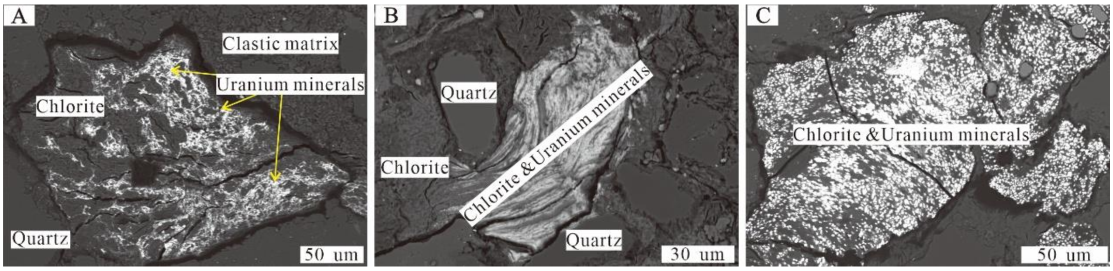

Fig. 16 Association of uranium minerals and clay minerals in sandstones in Lower

Member of Toutunhe Formation (J_2t^1), Louzhuangzi area (BSE images), including (A) Uranium mineral adsorbed in chlorite, ZK3-3, 23ZGe027, 753.10m; (B) Uranium mineral associated with chloritized biotite, ZK4-2, 23ZGE026, 398.20m; (C) Uranium minerals associated with chlorite, ZK12-3, 23ZGE028, 798.50m.

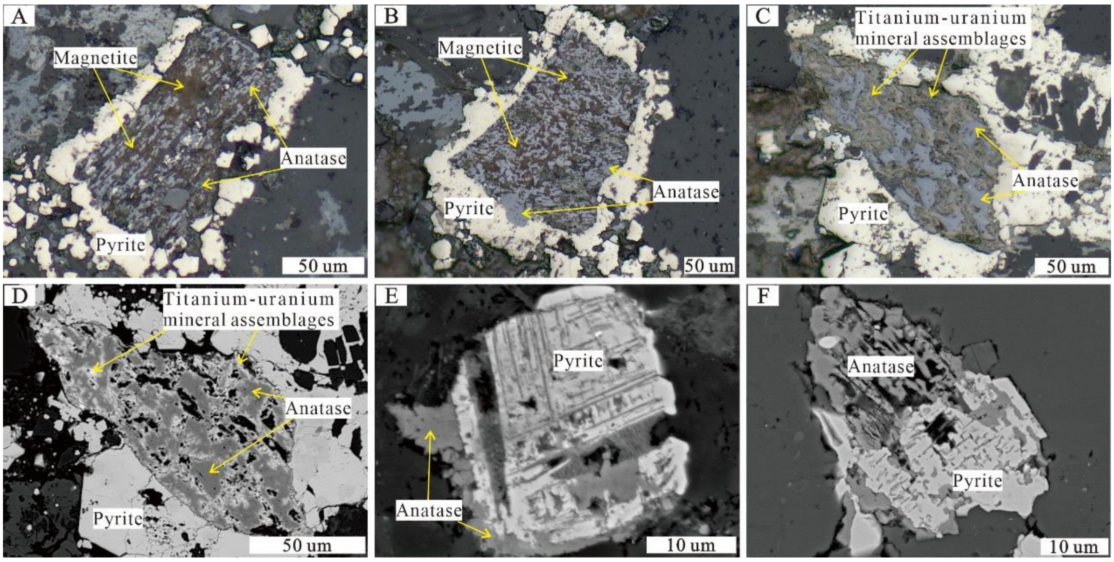

Fig. 17 Alteration characteristics of titanium oxides in sandstones in Lower Member of Toutunhe Formation (J_2t^1), Louzhuangzi area. (A-C) Anatase and magnetite mineral assemblage enveloped by gelatinous pyrite (reflected light); (D) uranium-bearing oxides enveloped by gelatinous pyrite; (E-F) Interwoven association of anatase and pyrite (BSE image).

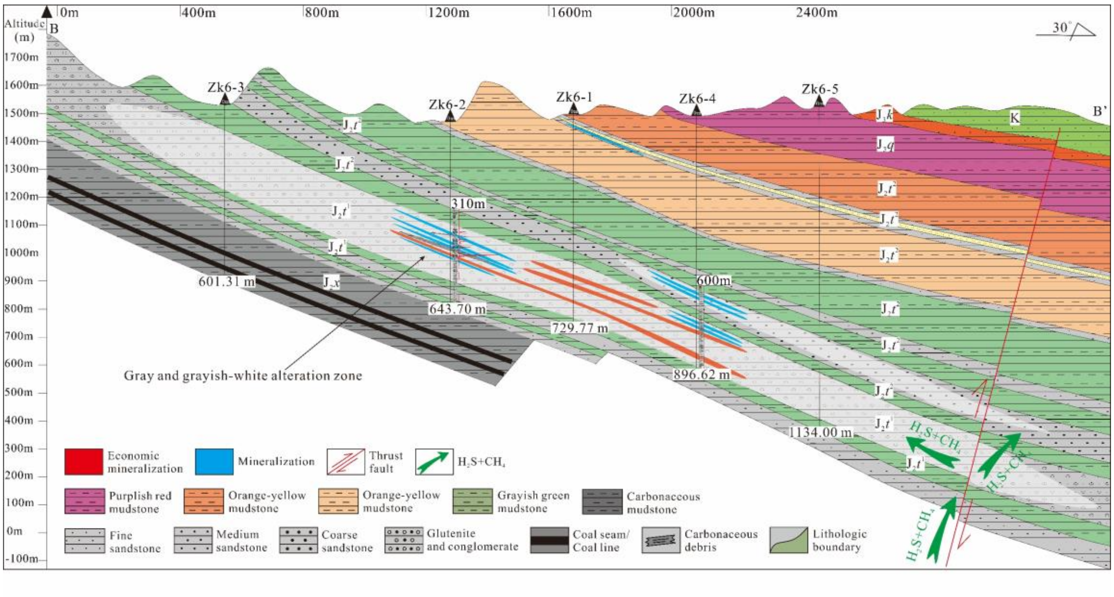

Fig. 18 Schematic diagram of the B-B ' drill hole profile in Louzhuangzi area. The profile position is shown in Figure 1C (modified from [15-16]).

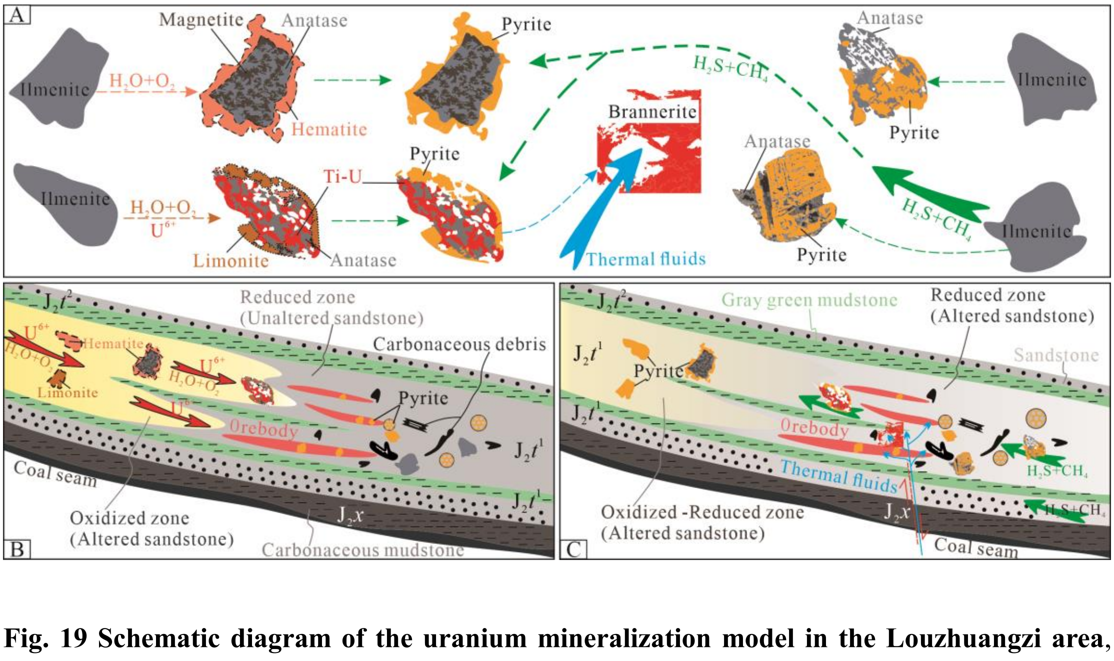

Fig. 19 Schematic diagram of the uranium mineralization model in the Louzhuangzi area, including (A) Types of mineral alteration in different fluid environments; (B) Interlayer oxidizing fluid mineralization; (C) Thermal fluid superposition transformation with oil and gas reduction.

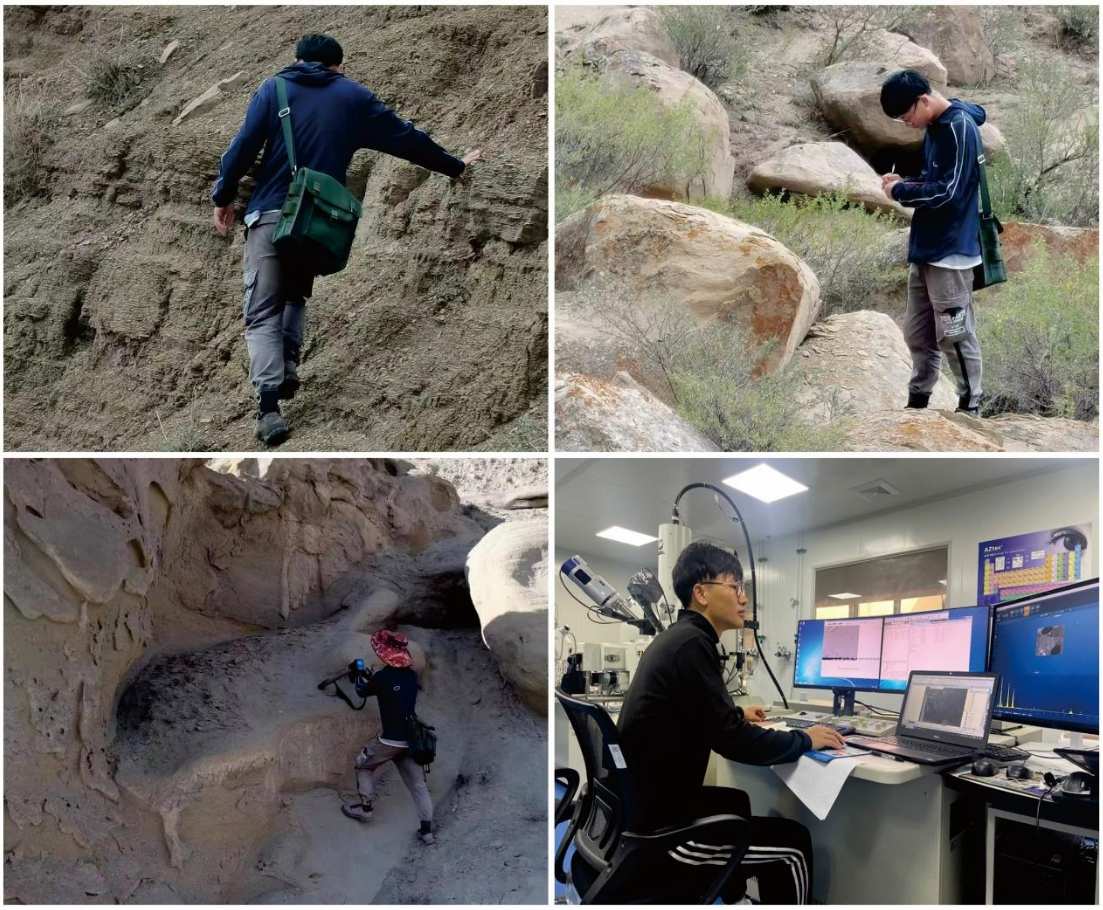

Images of the author's field geological survey and laboratory analysis work.

Click here to access/download
Supporting Information
Supporting information.docx

S1 Table. Sample Information

Click here to access/download
Supporting Information
S1 Table. Sample Information.docx

S2 Table. Electron probe microanalysis data

Click here to access/download

Supporting Information

S2 Table. Electron probe microanalysis data.docx

Click here to access/download
Supporting Information
Highlights.docx

Click here to access/download
Supporting Information
Declaration-of-competing-interests.docx

Manuscript

Click here to access/download
Supporting Information
Revised Manuscript with No changes Marked.docx

Revised Manuscript with Track Changes

Sedimentary architecture and post-depositional alteration of the Toutunhe Formation (J_2t) in the Louzhuangzi area, Southern Junggar Basin: Implications for uranium mineralization~~Control of deposition-epigenetic alteration of sandbody on uranium mineralization in Toutunhe Formation, Louzhuangzi area, Southern Margin of Junggar Basin, China~~

Formatted: Font: Italic

Qing Wang [1*], Fengjun Nie [1,2*], Fei Xia[1,2], Xin Zhang [1], Weiwei Jia [3], Kegai Lu [3], Xiao Sun [3], Dong-guang Yang[2]

[1] School of Earth and Planetary Sciences, East China University of Technology, Nanchang, Jiangxi, China;

[2] National Key Laboratory of Uranium Resources Exploration-Mining and Nuclear Remote Sensing, East China University of Technology, Nanchang, Jiangxi, China;

[3] No. 216 Geological Party, China National Nuclear Corporation, Urumqi, Xinjiang, China.

* Corresponding author

E-mail: niefj@263.net (FN); destinw@126.com (QW)

Abstract

In recent years, some uranium mineralization ~~shows~~ with ~~industrial~~economic values ~~have~~ has been discovered in the Louzhuangzi area, southern margin of the Junggar Basin, and a mediu~~m~~-sized uranium mineral area has been confirmed. To investigate the depositional environment and alteration characteristics of the ore-bearing target horizon—the Toutunhe Formation (J_2t)—in this mining area, this study is based on detailed field geological investigations, drill-core logging, and systematic

Formatted: Font: Italic

sampling. A preliminary assessment of the depositional environment and uranium mineralization characteristics of the target sandstone units was carried out. Petrographic features of the ore-bearing sandstones were examined using polarized light microscopy and scanning electron microscopy (SEM). Furthermore, electron probe microanalysis (EPMA) and transmission electron microscopy (TEM) were integrated to systematically characterize the mineralogical composition and modes of occurrence of uranium minerals.

The results indicate that the upper member of the target horizon (J_2t^2) exhibits typical meandering river depositional characteristics, manifested by "thick mudstone layers interbedded with thin sandstone layers." In contrast, economically significant uranium mineralization is hosted within the lower member (J_2t^1), which displays braided river depositional features characterized by "thick sandstone layers interbedded with thin mudstone layers." The ore-bearing sandstones show characteristics of proximal-source deposition and have undergone pronounced supergene alteration and post-diagenetic modification. The spatial distribution of uranium is closely associated with pyrite, organic matter, and clay minerals. Uranium minerals are predominantly coffinite and pitchblende, with UO_2 contents ranging from 52.89 % to 86.07 %. A minor proportion of uranium minerals, with UO_2 contents of 38.01 %–41.46 % and TiO_2 contents of 31.67 %–36.09 %, are tentatively interpreted as possible brannerite. In addition, unidentified nano-scale uranium minerals exhibit UO_2 contents ranging from 9.15 % to 60.28 %.

Finally, based on the occurrence characteristics of uranium and the results of

Formatted: Font: Italic

Formatted: Superscript

Formatted: Font: Italic

Formatted: Superscript

alteration mineralogical analyses, a conceptual model for uranium mineralization in the Louzhuangzi deposit is proposed. The formation and preservation of uranium mineralization in the target horizon are interpreted as the combined result of surficial and ascending fluid processes, likely governed by three principal factors: (1) the architectural framework of braided-channel sandstones, which provided favorable permeable pathways for fluid migration; (2) infiltration of surficial oxygen-bearing, uranium-rich fluids that initiated uranium mineralization, subsequently overprinted by post-ore thermal fluid modification; and (3) the action of deep-seated reducing fluids, which inhibited remobilization and oxidative destruction of uranium mineralization, thereby contributing to its preservation and stabilization.

However, the sandbody depositional environment controlling the uranium deposit remains still unclear, and the properties of the ore-forming fluids are also controversial, mainly due to the lack of systematic field geological survey and detailed altered mineral research. In this study, the depositional characteristics of the sandbodies in the Toutunhe Formation (J_2t) has been revealed through field geological investigation, collation of drill core data and petrographic analysis; the occurrence state of uranium and alteration characteristics of minerals coexisting with uranium has been explored by using scanning electron microscope (SEM), electron probe (EMPA), and focused ion beam scanning electron microscopy (FIB) - transmission electron microscopy (TEM). Comprehensive studies show that the uranium mineralization in the Toutunhe Formation (J_2t) of the Louzhuangzi area is controlled by the coupling of multiple factors such as the gravel braided river channel lag deposits, the multi-stage alteration of

~~epigenetic - thermal fluids, and the reduction of oil and gas.~~

Formatted: Font: 12 pt

1. Introduction

With the wide application of the in-situ leaching uranium mining technology, sandstone-type uranium deposit has become the main target of uranium resource exploration in China. Sandstone-type uranium deposits in northern China are mainly distributed in Mesozoic-Cenozoic foreland basins, such as Ili, Turpan-Hami, and Songliao basins, as well as the Ordos Basin [1-4]. ~~The sandstone-type uranium deposits in northern China are mainly found in the Mesozoic and Cenozoic fore-mountain basins, such as the southern margins of the Ili, Turpan-Hami, and Songliao Basins, and the northern part of the Ordos Basin [1].~~ Uranium source, depositional environment of host ~~sandbodies~~sandstones, reduction capacity of ~~sandbodies~~sandstones, epigenetic alteration, and regional tectonic activity are the main factors controlling the uranium mineralization [5-8~~1-5~~]. The Junggar Basin is one of the important basins in Xinjiang region, northern China where sandstone-type uranium deposits have been conducted. Some promising uranium mineralizations have been discovered in Jurassic, Cretaceous, and Paleogene sandstones in the Dingshan area of the northern basin, the Kamusite area of the eastern basin, and along the southern margin of the basin, attracting considerable attention from the uranium exploration industry [9]. ~~Some promising uranium mineralization has been discovered in the Jurassic, Cretaceous and Paleogene sandstones in the Dingshan area of the northern basin, the Kamusite area of the eastern basin and the southern margin of the basin, which has attracted high attention from the industry [6].~~ However, the uranium mineralization with ~~industrial~~economic values has

been discovered only in the Jurassic Toutunhe Formation (J_2t) of the Louzhuangzi area, southern margin of the basin at present, and a medium-sized uranium mineral area has been confirmed.

The sediment source of the Jurassic strata in the southern margin of the Junggar Basin mainly comes from the Northern Tianshan Mountains. The uranium-enriched geological bodies of the Northern Tianshan Mountains, together with conglomerate layers at the base of the Lower Cretaceous Qingshuihe Formation, provide important uranium sources for mineralization. In addition, relatively stable structural areas constitute favorable sites for uranium mineralization [3, 10]. ~~The uranium-enriched geological bodies of the Northern Tianshan Mountains and the conglomerate layers in the bottom of the Lower Cretaceous Qingshuihe Formation provide uranium source for the uranium mineralization, and the relatively stable structure areas are the favorable locations for the mineralization of uranium [1, 7].~~ The southern margin of the Junggar Basin was in a tectonically stable state during the period from the Late Cretaceous to Paleogene~~ period;~~. The Middle and Lower Jurassic strata experienced long-term interaction with uranium- and oxygen-bearing fluids, leading to the formation of significant uranium mineralization [11-12].~~the Middle and Lower Jurassic series received long-term modification by the uranium- and oxygen- containing water, resulting in the formation of a large amount of uranium mineralization [8-9].~~ Of which, the thick and large braided river facies ~~sandbodies~~sandstones in the Middle Jurassic Toulunhe Formation acts as the host rock of the sandstone-type uranium deposit [9~~6~~]. The uranium mineralization in the Louzhuangzi mineral area is controlled by the

interlayer oxidation zone that develops from the Haojiagou Uplift to the west and northwest directions [13~~10~~]. The mineralization occurs in the depositional ~~sandbodies~~sandstones of the braided river delta plain facies in the Lower member of the Toutunhe Formation (J_2t^1), with the ~~sandbodies~~sandstones being stable, thick and large, good in permeability, and rich in pyrite and organic matter [14~~11~~]. The uranium mineralization area can be classified into the yellow alteration zone and the grayish-white alteration zone. The development of the ~~industrial~~economic uranium deposit is controlled by the grayish-white alteration zone resulting from the reduction and bleaching effect of oil and gas in the later stage, in which, the native oxidation alteration zone was reduced to the grayish-white zone due to oil and gas exudation [15-16~~12-13~~]. In addition to the local oil and gas exudation that participated in uranium mineralization, the hydrocarbons migrated from other locations were also involved in uranium mineralization [16~~13~~]. The main occurrence forms of uranium in the Louzhuangzi mineral area include pitchblende, coffinite and titanium-uranium mineral assemblages [15~~12~~]. In the Toutunhe Formation (J_2t) of the Liuhuang'gou ore site, northeastern the Louzhuangzi mineral area, uranium mostly occurs in organic matter in irregular, grape-like, gelatinous and kidney-like forms, or coexists with clay mineral and pyrite. Uranium at this ore site is mainly hosted by pitchblende and unidentified uranium minerals enriched in SiO_2, P_2O_5, and Y_2O_3, with subordinate brannerite and adsorbed uranium [17].~~The components of uranium in this ore site are dominated by pitchblende and the unknown uranium minerals rich in elements such as Si, P and Y, with less brannerite and adsorbed uranium [14].~~

Currently, two competing genetic hypotheses currently frame the metallogenic theory of sandstone-type uranium deposits, resulting in pronounced academic divergence. The traditional interlayer oxidation-zone model emphasizes the lateral migration of surficial, oxygen-bearing, uranium-rich fluids through permeable sandstones, with uranium precipitation occurring at redox interfaces [18-19]. In contrast, the deep-fluid exhalation or superimposed mineralization model proposes that deep-seated fluids—such as hydrocarbons and thermal fluids—migrate upward, supplying reducing agents that facilitate uranium precipitation or exert superimposed modification on pre-existing mineralization [2, 20-21]. This interpretation has gained widespread acceptance in the Ordos Basin [22-24], the Songliao Basin [25-26], and the Erlian Basin [27-30].

Previous studies have identified the fundamental geological conditions of uranium mineralization in the Louzhuangzi mining area. Some researchers interpret the mineralization as the product of a single-stage surficial interlayer oxidation process, with the oxidation front acting as the principal ore-controlling factor [13], and argue that uranium enrichment is primarily governed by variations in sedimentary facies [14]. At the same time, other scholars emphasize the critical role of hydrocarbon reduction and thermal-fluid overprinting, proposing that the grayish-white alteration zones are closely linked to deep-seated fluid activity and represent a necessary condition for the formation of economically viable uranium mineralization [15-16, 31]. This divergence in interpretation has resulted in an unresolved understanding of the sedimentary–alteration coupling mechanism controlling uranium mineralization in the Toutunhe

Formation along the southern margin of the Junggar Basin. In particular, the temporal and spatial relationships among braided river sandstone architecture, permeability evolution, interlayer oxidation zones, and hydrocarbon-related reduction alteration remain insufficiently constrained. Furthermore, the nature of the metallogenic fluids and the mechanisms of superimposed transformation are still unclear, including the timing, intensity, and relative contributions of surficial oxygen-bearing fluids and deep-seated reducing fluids to uranium precipitation and preservation.

Uranium mineralization in sandstone-type deposits is intrinsically linked to the sedimentary architecture of host sandstones and their subsequent fluid-driven alteration. The uranium endowment of stratigraphic units is largely governed by dominant sedimentary facies, which are themselves controlled by paleoclimatic conditions and depositional environments [32]. In addition, the alteration characteristics of sandstones provide critical insights into the genetic type and evolution of uranium mineralization.

To elucidate the controlling effects of sandstone sedimentation and post-depositional alteration on uranium mineralization in the Toutunhe Formation (J_2t) along the southern margin of the Junggar Basin, this study integrates sedimentology, petrography, and alteration mineralogy within a multiscale analytical framework. Macroscopic sedimentological observations—including detailed field investigations and petrographic analysis—are coupled with micro- to nanoscale mineralogical characterization using scanning electron microscopy (SEM), electron microprobe analysis (EMPA), and focused ion beam–transmission electron microscopy (FIB–TEM). Based on these integrated investigations, we propose a conceptual model of coupled

Formatted: Font: Italic

mineralization control involving braided-river facies sandstone architecture and multi-stage metallogenic fluid processes. This model provides a necessary reference framework for uranium exploration in the Junggar Basin and in other analogous sedimentary basins.

The uranium mineralization uniquely derived from epigenetic oxidation fluid alteration and the in-situ leaching mining method of the sandstone-type uranium deposit determine the mineralization and production of uranium are closely related to the deposition-epigenetic alteration of the uranium-bearing sandbodies. The capacity of uranium mineralization in strata is controlled by the dominant sedimentary formation which in turn is controlled by paleoclimate conditions and sedimentary facies [15]. The types of uranium mineralization can be reflected by the epigenetic alteration of the uranium-bearing sandbodies.

In order to explore the controlling effect of sandbody deposition-epigenetic alteration on uranium mineralization in Toutunhe Formation (J_2t) in the southern margin of Junggar Basin, a study on the deposition-epigenetic alteration characteristics of the uranium-bearing sandbodies has been carried out guided by the uranium mineralization theory related to epigenetic oxidation fluid alteration, sedimentology and alteration mineralogy, and the depositional environment of the uranium-bearing sandbodies and the properties of the ore-forming fluids have been determined, providing a reference for determining the future uranium exploration direction in the basin.

The primary objective of this study is to elucidate the relationship between sedimentary facies architecture, post-depositional fluid alteration, and uranium

~~mineralization in the Toutunhe Formation (J_2t) of the Louzhuangzi area, southern Junggar Basin. We hypothesize that the formation and preservation of uranium mineralization are controlled by the combined effects of (1) braided channel sandbody architecture favorable for fluid migration, (2) multiple stages of epigenetic fluid alteration involving oxidizing, thermal, and reducing fluids, and (3) localized redox interfaces induced by hydrocarbons and sulfide mineralization (Fig 19). To test this hypothesis, we integrate macro-scale sedimentological observations with micro- to nano-scale mineralogical analyses using a combination of fieldwork, petrography, SEM, EPMA, and FIB–TEM techniques.~~

2. Geological background

The Junggar Basin is located on the~~lies in the~~ Kazakhstan, Plate between the Siberian Plate and the Tarim Plate, and is a ~~fore-mountain~~foreland basin in the plate collision orogenic belt. From the Cenozoic era, the Indian Plate collided with the Eurasian Plate and subducted, causing the Tianshan Orogenic Belt to be subjected to north-south compression [33-36~~16-19~~]. During regional compression, the Tarim stable block was located near the center of the compressive regime, resulting in progressive thickening of its lithospheric mantle. In contrast, the Tianshan Orogenic Belt acted as a buffer and stress-release zone, where crustal shortening and uplift gradually intensified. Through prolonged geological evolution, these processes ultimately shaped the present tectonic and geomorphic framework of the Junggar Basin, which is now separated from the Tuha and Ili basins by intervening mountain ranges.~~Since the stable block in the Tarim region was located at the center of the compression, the lithospheric mantle of~~

~~the stable block gradually thickened under compression, whereas the rock mass of the Tianshan Orogenic Belt, as the buffer and release area of the compressive stress, gradually rose and uplifted, and finally formed the current tectonic and geomorphic pattern of the Junggar Basin after long period of geological evolution, which is separated from the Tuha and Ili Basins by mountains~~ [37~~20~~] (Fig 1A).

The study area is located in the level-II tectonic province at the southern margin of the Junggar Basin (Fig 1B), belonging to the front of the fore-mountain fold-and-thrust belt of the Northern Tianshan Mountains. A thrust fault belt associated with folding structures about 300km long from east to west and nearly 50-80km wide from north to south develops from the fore-mountain fold belt of the northern Tianshan Mountains to the center of the basin [12~~9~~] (Fig 1B). In recent years, the Louzhuangzi uranium mineral area has been discovered in the intersection and subduction site of the N-WW oriented Yilianhabirga Mountains and the western sloping end of the N-E oriented Bogda Mountain tectonic belt (Haojiagou Uplift) (Fig 1B) in the fore-mountain fold-and-thrust belt of the Northern Tianshan Mountains. This newly discovered uranium mineral area, together with the previously found uranium mineral sites such as Hasa tomb and Wanjiayao, and the uranium anomaly sites, forms a prospective sandstone-type uranium mineralization zone [9, 15~~6, 12~~] (Fig 1C).

Fig.1 (A) Sketch map showing tectonic location of Junggar Basin (modified from [38~~21~~]); (B) Geological sketch map of Louzhuangzi area (modified from [15~~12~~]); (C) Division and distribution of the main tectonic units at southern margin of Junggar Basin (modified from [12~~9~~]).

The Cenozoic strata in the southern margin of the Junggar Basin are completely

exposed, of which the Jurassic, Cretaceous and Paleogene systems constitute the main horizons for exploring the sandstone-type uranium deposits (Fig 2). The Jurassic strata are widely developed in the Louzhuangzi area (Fig 1C), and the strata revealed by outcrop profile and drilling data from top to bottom include the Karaza Formation (J_3k), Qigu Formation (J_3q), Toutunhe Formation (J_2t), Xishanyao Formation (J_2x), Sangonghe Formation (J_1s), and Badaowan Formation (J_1b). Based on the previous comprehensive study on the sedimentary formation, lithology combination and logging curve characteristics of the Junggar Basin, the Jurassic system has been divided into the river depositional system, the delta depositional system and the lake depositional system [39~~22~~]. The Karaza Formation (J_3k) and the Qigu Formation (J_3q) are alluvial fan facies deposits, the Toutunhe Formation (J_2t) is a fluvial facies deposit, the Xishanyao Formation (J_2x) is a delta facies deposit, the Sangonghe Formation (J_1s) is a shallow lake – shore shallow lake subfacies deposit, and the Badaowan Formation (J_1b) is a fluvial and swamp facies deposit (Fig 2). Among which, the Toutunhe Formation with fluvial facies deposits is the main uranium-bearing horizon in the Louzhuangzi mineral area (Figs 1C and 2).

Fig. 2 Stratigraphic column of the Louzhuangzi area (modified after an internal report from Geological Party No. 216, CNNC, 2022).~~**Fig. 2 Composite stratigraphic column of Louzhuangzi area** (Modified based on the internal report from the geological party No. 216, CNNC, 2022).~~

3. Materials and methods

3.1 Fieldwork and sampling

Field investigations were conducted in the Louzhuangzi area on the southern

Formatted: Font: Not Bold

Formatted: Font: 16 pt, Font color: Auto

Formatted: Font: 16 pt

Formatted: Heading 2

margin of the Junggar Basin. Stratigraphic logging, outcrop observation, and core description were used to define depositional facies and alteration features. Sampling was restricted to the Toutunhe Formation (J_2t), with particular emphasis on the grayish-white alteration sandstones hosting economic uranium mineralization, thereby ensuring close consistency between sampled materials and the research focus on sandstone alteration and its control on uranium mineralization. Core samples were preferentially selected from lithologically intact and unweathered intervals.

To capture the vertical (depth-related) and lateral (lithology-related) mineralogical variations within the grayish-white alteration zone, samples were collected from different depths and lithologies. Sampling sites were systematically arranged from west to east across the Louzhuangzi deposit, spanning the full distribution range of the grayish-white alteration zone within the Toutunhe Formation. Laterally, the western, central, and eastern key sectors of the alteration belt were covered; vertically, sampling depths ranged from 24.84 m to 871.50 m. This strategy enables a comprehensive characterization of the spatial distribution and vertical evolution of the alteration zone and ensures robust spatial representativeness.

Representative drill holes were selected for detailed characterization of internal alteration features, clarification of the relationship between alteration intensity and lithology, and targeted fine-scale sampling. The sample list (Table 1) provided in this study includes the principal specimens involved in the analytical work and does not represent the complete inventory of systematically collected samples.~~Drill core samples were collected from the Toutunhe Formation (J_2t), with sample depths recorded in Table~~

~~1. Sampling sites were selected to represent grayish-white alteration zones of varying lithology and depth within the deposit from west to east.~~

3.2 ~~Petrographic and mineralogical~~ Mineralogical analysis

Formatted: Font: 16 pt, Font color: Auto

Formatted: Heading 2, Indent: First line: 0 ch

Formatted: Font: 16 pt, Font color: Auto

Formatted: Font: 16 pt

Thin sections were prepared for petrographic observation under transmitted light using a Zeiss AxioScope.A1 digital polarizing microscope. Mineral assemblages, textures, and diagenetic features were identified and classified.

Scanning electron microscopy (SEM)

Selected samples were carbon-coated and analyzed using a FEI Nova Nano SEM 450 equipped with an energy-dispersive X-ray spectroscopy (EDS) system. SEM was used to identify uranium-bearing mineral phases, textural relationships, and alteration rims.

Electron microprobe analysis (EPMA)

Quantitative compositional data of uranium minerals and Ti-bearing phases were obtained using a JEOL JXA-8100 EPMA with Inca Energy EDS. Analytical conditions included 15 kV accelerating voltage and 10 nA beam current~~.~~, and spot diameter ≤ 2 μm. The counting time was 5 s per element. X-ray lines analyzed included: SiKα, AlKα, MgKα, KKα, NaKα, YLα, CaKα, PKα, FeKα, TiKα, ZrKα, PbMα, MnKα, UMα, ThMα, and CeLα. The following mineral standards were used for calibration: Jadeite (Si, Al, Na), Sanidine (K), Olivine (Mg, Fe), Plagioclase (Ca), Rutile (Ti), Zircon (Zr), Cubic zirconia (Y), Monazite (P, Ce), Uraninite (Pb, U, Th), Rhodonite (Mn). Electron microprobe analyses show that the total oxide contents range from 84.01% to 97.14%. Some analyses are of relatively poor quality, with total oxide contents below 85 wt%.

This suggests that some mineral phases may contain volatile components or undetected constituents (e.g., structural water or beam-sensitive phases), and that amorphous domains may also be present, which complicate quantitative analysis—for example, the auto-oxidation of U^{4+} to U^{6+} [40].

FIB-TEM sample preparation and transmission electron microscopy

Focused ion beam (FIB) sample preparation was conducted at the Guangzhou Institute of Geochemistry to prepare ~100 nm thick foil sections for TEM analysis. The Ti-U mineral assemblage of brannerite-like was characterized at nanometer resolution using a Thermo Scientific Talos F200S TEM (200 kV) equipped with EDS.~~Thermo Scientific Talos F200S TEM with EDS (operated at 200 kV) was employed to characterize nanocrystalline and amorphous phases, crystal lattice fringes, and mineral associations at nanoscale resolution.~~

Except for FIB sample preparation and TEM-EDS analysis, all other experiments were conducted at the State Key Laboratory of Nuclear Resources and Environment, East China University of Technology.

4. Results

4.1 Petrographic features of the Toutunhe Formation sandstones

The detailed observation and description of the drill cores from the Toutunhe Formation (J_2t) in the study area, together with the field outcrop observation, show that the rock types developed in the Toutunhe Formation (J_2t) mainly include mudstone, fine, medium and coarse sandstone, gravel-bearing sandstone, glutenite and conglomerate.

The mudstone is commonly interbedded with sandstone with rich variety of colors. Besides the common grayish green mudstone, brownish red and red-dominated varicolored ones also exist (Fig 7A). The structure type of the mudstone is dominated by massive beddings (Figs 3A and B). The sandstone revealed by drilling is normally grayish white or gray (Fig 7B), and the sand yellow and dark purple sandstones occasionally occur on outcrops (Fig 3B). From the perspective of grain size, the medium-coarse sandstone is dominant, followed by the medium sandstone, fine sandstone and siltstone. The depositional structures such as flaser bedding (Fig 3C), parallel bedding (Fig 3A), horizontal bedding (Fig 3B), and small to medium (with the single-layer system < 10 cm) planar cross bedding (Figs 3C and 12B) are commonly developed in the sandstone. The gravel-bearing sandstone, glutenite and conglomerate revealed by drilling are grayish white (Fig 7B), containing a considerable amount of carbonaceous debris and pyrite (Figs 12J-L), with clayization developed (Figs 12~~J,~~ K~~,~~ ~~and L~~). The depositional structures such as large to giant (with the single-layer system > 10 cm) planar cross bedding (Fig 3D), massive bedding (Fig 3E), trough cross bedding (Fig 3F) and surface of scour are developed commonly in the outcrops. The components of the conglomerate mainly include silicite, granite and tuff with poor sorting, and generally being sub-angular to sub-round in roundness.

Fig. 3 Petrographic photographs of Toutunhe Formation (J_2t) outcrops in Louzhuangzi area: (A-C) ~~Sandbody~~Sandstone outcrop of the Upper Member, Toutunhe Formation (J_2t^2), showing fine-medium sandstone with parallel beddings (A), mudstone with massive beddings (B), fine-medium sandstone with horizontal beddings (B), and fine sandstone with flaser beddings and planar cross

beddings (C); (D-F) ~~Sandbody~~Sandstone outcrop of the Lower Member, Toutunhe Formation (J_2t^1), showing sandstone with large to giant planar cross beddings (D), conglomerate with massive beddings (E), and glutenite and coarse sandstone with trough cross beddings (F); (G) Interface between the Lower Member of the Toutunhe Formation (J_2t^1) and the Xishanyao Formation (J_2x); (H) ~~Sandbody~~Sandstone outcrop of the Xishanyao Formation (J_2x), showing siltstone with parallel beddings and coal lines.

Table 1. Property of sandstone samples from Toutunhe Formation (J_2t) of Louzhuangzi area, southern margin of Junggar Basin.

Sample number	Drill hole	Sampling depth / (m)	Lithology description
**23DJA-6**	ZK16-2	654.60	Grey glutenite
**23DJA-9**		705.70	
**22ZGE001**	ZK12-2	672.48	Grey gravel-bearing coarse sandstone
**23DJA-4**		680.70	
**23ZGE016**	ZK12-3	838.64	
**23ZGE028**		798.50	
**23DJA-15**		871.50	Grey glutenite
**23DJA-27**		641.70	
**22ZGE014**	ZK6-2	483.47	Grey gravel-bearing coarse sandstone
**22ZGE015**		479.30	
**22ZGE016**		477.30	
**22ZGE026**		398.80	

**22ZGE063**		478.60	
**23ZGE027**	ZK3-3	753.10	Grey white fine sandstone
**23ZGE026**	ZK4-2	398.20	Grey white coarse sandstone
**23DJA-7**	ZK4-3	710.40	Grey gravel-bearing
**22ZGE033**	ZK5-1	148.50	coarse sandstone
**22ZGE034**	ZK11-2	24.84	Grey coarse sandstone
**23ZGE022**		119.52	Grey white gravel-
	ZK11-3		bearing coarse
**23ZGE024**		79.20	sandstone

The components of the sandstone clasitcs in the Toutunhe Formation (J_2t), Louzhuangzi area have been identified by using a polarizing microscope, the statistical analysis of the area proportions of quartz, feldspar and debris under the field of view has been performed, and the percentage content of each type of clastics components has been determined. When the statistical data are projected into the Folk's ternary diagram for sandstone classification, it can be seen that the type of sandstone is mainly feldspar lithic sandstone, followed by lithic feldspar sandstone, and the feldspar sandstone is just rarely found (Fig 4). Studies on the components of the sandstone clastics show that the percentage contents of the debris and feldspar in the sandstone are relatively high. Specifically, quartz accounts for 18% to 59%, averaging 38%; feldspar accounts for 15% to 50%, averaging 26%; debris accounts for 10% to 60%, averaging 35%. The

compositional maturity index Q/(F+R) of the sandstone ranges from 0.22 to 1.44, averaging 0.65, which is relatively low~~, indicating that the type of the sandstone is greywacke and the sandbodies were formed in a proximal deposit environment~~.

Fig. 4 Classification diagram of sandstones in Toutunhe Formation (J_2t), Louzhuangzi area (According to [41~~23~~]).

The content of sandstone clastics ranges from 70% to 89%, averaging 82%, with quartz, feldspar and debris as the main components. The sorting property of the clastic particles is mostly poor, and the roundness is mainly sub-angular to angular, with only a small amount of debris being sub-round to round (Figs 5 and 6). The clastic particles are relatively dispersed and generally do not contact with each other (Figs 5A-F), with the particle diameters being up to 2600 μm~~um~~, mostly between 500 and 1500 μm~~um~~, averaging about 800 μm~~um~~. The structural type is mainly coarse-giant grained sandy structure, followed by fine-medium grained sandy structure. The interstitial materials between the clastiic particles are mainly composed of matrix and cements, with a content of approximately 20% to 30%. The matrix content is relatively high, with the components being mainly fine silty quartz and feldspar particles (Figs 5B, C, E), followed by a small amount of clay mineral, such as illite, sericite (Fig. 5B), ~~illite/smectite mixed layer material (Fig 10F), ~~and kaolinite, smectite, chlorite (Figs 10E, F and ~~14G-I~~15, 16). In the uranium mineralized sandstone samples, finely dispersed uranium minerals and fine-grained pyrite are commonly observed in the matrix, and associated with clay minerals and carbonaceous debris (Figs 5H, I and 10A-C). The cementing materials are mainly composed of carbonate and clay minerals ~~(such~~

as kaolin and chlorite/smectite mixed layer minerals (Figs 5A and 6F), followed by gelatinous pyrite (Figs 5G and I). The main cementing types are mostly basal cementation, with matrix-supported structures (Figs 5A-F). These clastic particles features indicate that both the compositional maturity and the structural maturity of the sandstone are generally low.

Fig. 5 Petrographic characteristics of sandstone samples from Toutunhe Formation (J_2t) in Louzhuangzi area under microscope: (A) Sharp-edged quartz clasts cemented by basal carbonate cementation, ZK5-1, 22ZGE033, 148.50 m, (+); (B) Clastics and clay matrix, ZK6-2, 22ZGE016, 477.30m, (+); (C) Pink crystalline quartz and feldspar matrix filled among debris particles, ZK6-2, 22ZGE016, 477.30m, (+); (D) Illitizated plagioclase with relatively dirty surface, ZK6-2, 22ZGE063, 478.60m, (+); (E) Lattice twin crystal microcline, ZK6-2, 22ZGE014, 483.47m, (+); (F) Bending and crushing of serimicized biotite, ZK12-2, 22ZGE001, 672.48m, (+); (G) Pyrite cementation, ZK4-2, 23ZGE026, 398.20m (reflected light); (H) Fine-grained pyrite and uranium minerals filled the cell cavities of carbonaceous debris, ZK4-2, 23ZGE026, 398.20 m, (BSE image); (I) Pyrite and uranium minerals filled among the clastic particles, ZK12-3, 23ZGE028, 798.50 m, (BSE image).

The relative content of quartz ranges from 22% to 59%, averaging 40%, with single crystal quartz being the main component, accounting for about 65% of the total amount of quartz, followed by polycrystalline quartz, accounting for about 35%. The quartz is generally characterized by dirty and rough surface, with poor roundness, and commonly shows angular and sub-angular shapes (Figs 5A-D and 6C, D). The feldspar content ranges from 11% to 36%, averaging 21%, with microcline and plagioclase

being the main components. The microcline is mainly the characterized by lattice twin crystal structures, with the twin crystal bands usually having a spindle-shaped lattice pattern under the microscope. (Fig 5E). The plagioclase mainly shows the characteristics of polysynthetic twin texture, with serimicization usually occurring on the surface (Fig 5D), and compaction and crushing of altered biotite occasionally being observed (Fig 5F).

The debris particles have relatively large diameters and good roundness being sub-round and round, with cement and detrital matrix usually observed on the edge. The debris can be classified into the following 3 types according to the rock property in the source area: igneous rock debris, sedimentary rock debris and metamorphic rock debris. The commonly observed tuff debris (Fig 6A), dacite porphyry debris (Fig 6D), and granite debris (Figs 6E, F) are all the products of fragmentation and deposition of the intermediate acidic igneous rocks and intrusive rocks. The sedimentary rock debris is composed mainly by siliceous rock debris (Fig 6C), while flint debris (Fig 6E) and quartz schist debris (Fig 6B) constitute the main types of the metamorphic rock debris. The rhyolite spherulitic texture, almond shaped body, is clearly visible in the igneous rock debris (Fig 6A), indicating that the weathering of the pro-rock is relatively intense. Porphyritic texture is identified in part of the acidic volcanic rock debris (Fig 6D), with felsic mineral as the main composition of the phenocryst and cryptocrystalline quartz as the matrix. In some thin slices of rock samples, the epigranite debris can be found, the granitic texture composed of quartz and feldspar is clearly visible, whereas most of the mica-type minerals are absent (Figs 6E, F). The metamorphic rock is mainly

composed of the quartzite debris that accounts for 90% of the metamorphic rock debris, mainly in the forms of polycrystalline quartz debris (Fig 6C) and flint debris (Fig 6E), followed by the quartz schist debris (Fig 6B), with obvious platy structures.

Fig. 6 Debris characteristics of sandstone samples from Toutunhe Formation (J_2t) in Louzhuangzi area under microscope: (A) Cryptocrystalline tuff debris, ZK6-2, 22ZGE016, 477.30 m, (+); (B) Platy-structure quartz schist debris, ZK6-2, 22ZGE015, 479.30m, (+); (C) Sericitization of sharp edged polycrystalline quartz, ZK6-2, 22ZGE016, 477.30m, (+); (D) Dacite porphyry debris with porphyritic texture, ZK6-2, 22ZGE026, 398.80m, (+); (E) Dissolution pit visible in cryptocrystalline flint debris, ZK6-2, 22ZGE026, 398.80m, (+); (F) Carbonate-cemented granite debris, ZK11-2, 22ZGE034, 24.84 m, (+).

4.2 Sandstone depositional characteristicsDepositional environment and facies interpretation

The Toutunhe Formation (J_2t) can be divided vertically into upper and lower members. The Upper Member (J_2t^2) consists of relatively thick purple-red to dark purple mudstone interbedded with thin grayish-white siltstone and fine sandstone layers (Fig 7A). Depositional microfacies including floodplain, natural levee, point bar, and channel-fill deposits are recognized. Sedimentary structures observed in the Upper Member include massive bedding in mudstone (Fig 3B), flaser bedding in fine sandstone (Fig 3C), horizontal bedding in mudstone and sandstone (Fig 3B), parallel bedding in sandstone (Fig 3A), and small- to medium-scale planar cross-bedding (Figs 3C and 12B).

The color, lithology and depositional facies of sandbodies can play important roles

Formatted: Font: Italic

Formatted: Subscript

Formatted: Font: Italic

Formatted: Superscript

in inferring the depositional environment of the sandbodies. The color can indirectly reflect the changes in paleoclimate and water environment, while the lithology can provide important information about depositional facies.

Vertically, the Toutunhe Formation (J_2t) can be divided into upper and lower members. The Upper Member is composed of relatively thick, purple red and dark purple mudstone layers interbedded with thin, grayish white siltstone and fine sandstone layers (Fig 7A), with the depositional microfacies such as floodplain, natural levee, point bar, and channel fill developed. The mudstone with massive beddings (Fig 3B) is mainly deposited in the floodplain environment or in the abandoned river channels, with its formation being related to the abundant supply of sediments and the weak action of water flow, and is commonly found in the low-energy areas of rivers or the marginal areas of sediment accumulation [23-24]. The fine sandstone with flaser beddings (Fig 3C) is mainly formed by the migration and deposition of the sandy sediments on the bottom of the meandering river channel under the action of water flow [6], with characteristics of fine particle size and clear beddings, reflecting the transportation and redistribution effects of water flow on sediments [25]. The mudstone and sandstone with horizontal beddings (Fig 3B) are mainly deposited in the floodplain or onlap area of the meandering river, where the water flow is usually weak, and the sediments are mainly accumulated in the horizontal direction, forming regular horizontal bedding structures [26]. The sandstone with parallel beddings (Fig 3A) is mainly deposited in a high flow regime planar laminar flow environment, with its bedding direction being consistent with the direction of water flow, reflecting relatively

~~strong dynamic conditions of water flow [27]. The sandstone with small to medium planar cross beddings (Figs 3Cand 12B) is mainly formed by the lateral migration and deposition of sediments, with its bedding structure indicating that the sediment has migrated in a certain direction and accumulated under the action of water flow, generally showing the depositional characteristics of a meandering river environment with slightly weak hydrodynamic force [28].~~

Fig. 7 Photographs of representative core samples from Toutunhe Formation (J_2t) exposed by the ~~industrial~~economic uranium mineralization drill hole of ZK5-7 in Louzhuangzi area: (A) Meandering river facies ' thick mud with thin sand from the Upper Member of the Toutunhe Formation (J_2t^2); (B) Gravelly braided river facies thick sand body from the Lower Member of the Toutunhe Formation (J_2t^1); (C) Sandy braided river facies flood mud and crevasse splay sand from the bottom of the Lower Member of the Toutunhe Formation (J_2t^1) .

The ore-bearing main sandstones in the Lower Member of the Toutunhe Formation (J_2t^1) is composed of the thick, grayish-white coarse sandstone, glutenite, and conglomerate interspersed by the thin layers of grayish-green mudstone on the profile (Fig 7B), with the depositional microfacies such as sandy channel fill, lag deposit and gravel bar developed (Fig 8). The Glutenite with trough cross-bedding (Fig 3F) and conglomerate with massive bedding (Fig 3E) also occur. At the base of the Lower Member (J_2t^1), gray to grayish-white coarse sandstone and medium–fine sandstone interbedded with green mudstone are observed (Fig 7C). Depositional microfacies including floodplain deposits, crevasse splays, and channel bars are identified (Fig 8), and sedimentary structures such as massive bedding, small-scale planar cross-bedding,

Formatted: Subscript
Formatted: Font: Italic
Formatted: Superscript
Formatted: Font color: Accent 1
Formatted: Font color: Accent 1
Formatted: Font: 12 pt
Formatted: Font: Italic
Formatted: Superscript

climbing ripple bedding, and scour surfaces are common (Fig 8). ~~The ore-bearing main sandbody in the Lower Member of the Toutunhe Formation (J_2t^1) is composed of the thick, grayish-white coarse sandstone, glutenite, and conglomerate interspersed by the thin layers of grayish-green mudstone on the profile (Fig 7B), with the depositional microfacies such as sandy channel fill, lag deposit and gravel bar developed (Fig 8). The glutenite with large scale, planar cross beddings (Fig 3D) is mainly formed by downstream aggradational braided flow deposition, with the characteristics of large gravel particle size and regular beddings, reflecting the rapid water flow and rapid sediment accumulation in the braided river depositional environment [29]. The glutenite with trough cross beddings (Fig 3F) and conglomerate with massive beddings (Fig 3E) are mainly formed by the erosion and filling deposition of braided river channels, with the bedding structures showing that the sediments were re-accumulated after being eroded by water flow in the braided river channels, generally presenting the characteristics of gravel braided fluvial facies deposition with strong hydrodynamic force [30]. The sandbody at the bottom of the Lower Member of the Toutunhe Formation (J_2t^1) is composed of gray and grayish-white coarse sandstone and medium-fine sandstone and green mudstone (Fig 7C), with the depositional microfacies such as floodplain, crevasse splay and channel bar developed (Fig 8), and the depositional structures such as massive bedding, small-scale planar cross bedding, climbing ripple bedding and scour surface (Fig 8) commonly observed, presenting the deposition characteristics of a sandy braided river environment with moderate hydrodynamic force.~~

Up-section from the Lower to the Upper Member (J_2t^1), thick conglomerate and coarse sandstone layers decrease, whereas grayish-white siltstone and fine sandstone layers become more abundant. In addition, multiple gypsum layers are present within the succession (Figs 12E, I).

~~The thick conglomerate and coarse sandstones gradually disappear, and the grayish-white siltstone and fine sandstone layers gradually increase in the sandbodies from the Lower Member to the Upper Member of the Toutunhe Formation (J_2t) [6], and meanwhile, multiple layers of gypsum are found in the sandbodies (Figs 12E , I), indicating the sandbodies were formed in a depositional environment where the paleoclimate became increasingly arid, the hydrodynamic force gradually weakened, and the water body became continuously shallower from bottom to up [31].~~

Fig. 8 Interpretation of the core profile of drill hole ZK5-1 in Louzhuangzi area. (Abbreviation: Fm=Formation; DF= Depositional Facies; OD= Overbank Deposit; SCF= Sandy Channel Fill; LD= Lag Deposit; GB= Gravel Bar; FP= Floodplain; CS= Crevasse Splay; CB= Channel Bar; IDB= Interdistributary Bay; SDC= Subaqueous Distributary Channel).

4.3 Uranium mineralization characteristics

The uranium mineralization in the Louzhuangzi area occurs in the ~~sandbodies~~sandstones of the Lower Member of the Toutunhe Formation (J_2t^1). The classification of depositional facies based on outcrops and borehole strata, together with previous studies, shows that the Lower Member of the Toutunhe Formation (J_2t^1) contains sandstones deposited in braided-river delta plain, sandy braided-river, and gravelly braided-river facies (Figs 7-9). ~~The classification studies of the depositional~~

Formatted: Font: Italic

Formatted: Superscript

Formatted: Font color: Accent 1

facies of the outcrops and borehole strata, together with the previous study results, document that the Lower Member of the Toutunhe Formation (J_2t^1) contains the depositional sandbodies of braided river delta plain, sandy braided river and gravelly braided river facies deposits (Figs. 7, 8 and 9). The uranium mineralization zone, large in scale, is distributed along the direction of the sandbodysandstone trend from east to west, being about 5.5 km long from east to west and about 800 m – 1,100 m in width (Fig 1C), controlled mainly by the depositional sandbodiessandstones of braided river delta plain deposition (the main channel of gravelly braided river) (Fig 14). The A-A 'borehole profile (Fig 9) exposes multiple layers of uranium bodies and uranium mineralized bodies in the depths ranging from 32.05 m to 695.55 m, with the depth being deeper in the west and shallower in the east (approaching the eroded surface). The thickness of the industrialeconomic ore bodies is generally between 0.8 m and 5.6 m, controlled by a set of gravelly braided river depositional sandbodiessandstones, which are thick and large, with coarse sandstone, glutenite, and conglomerate being interbedded frequently, and are loose to relatively loose in structure, with good permeability, connectivity and stratification, and rich in carbonized plant detritus. The gravelly braided river channel lag deposits is the main position for the formation of uranium mineralization (Fig 8). The thick mudstone layer deposited in the meandering river environment in the Upper Member of the Toutunhe Formation (J_2t^2) and the mud-bearing sandbodysandstone at the bottom of the Lower Member (J_2t^1) act as the top and bottom plates of the aquiclude, respectively, and form a sandbodysandstone structure favorable for uranium mineralization together with the thick gravel-bearing coarse

sandstone, glutenite, and conglomerate layers in the middle and upper sections of the Lower Member (J_2t^1) (Fig 9).

Fig. 9 Sketch diagram showing the A-A' profile in Louzhuangzi area (In the Lower Member of the Toutunhe Formation (J_2t^1), the top roof of the sandstone has been leveled. The profile position is shown in Figure 1C).

4.4 Uranium occurrence and mineral assemblages

In the uranium ore samples from the Toutunhe Formation (J_2t) of the Louzhuangzi area, uranium occurs mainly in ~~3~~three types of state including the independent uranium mineral, unclassified nano-uranium minerals~~the dispersed adsorbed mineral~~ and the isomorphism substitution mineral.

4.4.1 Independent uranium mineral

The independent uranium minerals mainly include coffinite and pitchblende, with a small amount of brannerite-like.

Electron probe analyses indicate that the diameters of the coffinite particles generally range from 5 μm to 30 μm, with these particles mostly being distributed among the clastic particles in gel-like forms, and coexisting with gelatinous pyrite (Fig 10A); the finely dispersed coffinite particles associated with ~~clay minerals (~~chlorite~~, and chlorite/smectite mixed layer mineral)~~ are commonly observed (Figs 10F and ~~15~~16), and the coffinite particles are also commonly observed occurring in ring-shaped distribution on the quartz surface and coexist with pitchblende (Figs 10D, I); massive coffinite particles (with particle diameter up to 200 μm) can occasionally be observed

in the matrix (Fig 10B). The coffinite is generally rich in Y_2O_3, CaO, P_2O_5 and FeO, with the total amount of these components accounting for 87.51% to 97.14%, averaging 91.81%. Specifically, the contents of SiO_2, UO_2, Y_2O_3, CaO, P_2O_5, FeO range from 9.33% to 18.23%, 52.89% to 66.88%, 0.50% to 7.33%, 0.62% to 7.21%, 1.06% to 6.44%, and 0.09% to 4.59%, respectively, with the average contents of 14.31%, 61.33%., 3.40%., 3.42%, 3.43%, and 1.55%, respectively. Additionally, the coffinite also contains a small amount of impurities such as TiO_2, ZrO_2, PbO, and MnO (Table 2).

Fig. 10 Occurrence characteristics of uranium in sandstones of Toutunhe Formation (J_2t) in Louzhuangzi area (BSE images), including (A) Gelatinous coffinite associated with pyrite, ZK11-3, 23ZGE022, 119.52m; (B) Massive coffinite associated with fine-grained pyrite, ZK12-3, 23ZGE028, 798.50m; (C) Massive pitchblende associated with pyrite, ZK12-3, 23ZGE028, 798.50 m; (D) Baited pitchblende coexisted with coffinite with ring-shaped distribution; ZK4-2, 23ZGE026, 398.20 m; (E) Nanoscale titanium-uranium aggregates associated with chlorite, ZK3-3, 23ZGE027, 753.10m; (F) Nanoscale uranium particles adsorbed in clay minerals, ZK12-3, 23ZGE028, 798.50m; (G) Nanoscale uranium minerals adsorbed at the edge of titanium oxides, ZK4-2, 23ZGe026, 398.20m; (H) Nanoscale uranium minerals adsorbed in the dissolution pores of titanium oxides, ZK12-3, 23ZGE028, 798.50 m; (I) Uranium-rich monazite associated with baited pitchblende, ZK4-2, 23ZGE026, 398.20m; (J) Uranium-rich rutile, ZK4-2, 23ZGE026, 398.20 m; (K-L) Area distribution of U(K) and Ti (L) elements in uranium-rich rutile obtained by electron probe.

Pitchblende particles are relatively fine, generally ranging from 5 μm to 20 μm in diameters, and its content in the independent uranium minerals is lower than that of

coffinite. The composition and appearance characteristics of pitchblende can basically be determined by electron microscope. Under the electron microscope, the massive pitchblende particles can be observed to appear in the matrix among the clastic particles, often associated with clay minerals and fine-grained pyrite (Fig 10C), and the baited pitchblende can also be commonly observed to be wrapped by the in ring-shaped distribution of coffinite in the quartz dissolution pits (Figs 10D, I). The pitchblende is characterized by a lower SiO_2 content and a higher UO_2 content. The total amount of the pitchblende chemical components accounts for 86.29% to 95.38%, with an average of 90.63%. In which, SiO_2 content ranges from 1.05% to 5.76%, averaging 2.36%. and UO_2 content ranges from 75.50% to 86.07%, averaging 81.06%. Additionally, the pitchblende also contains a small amount of impurities such as Y_2O_3, CaO, P_2O_5, and FeO (Table 2).

Fig. 11 Images showing the characteristics of titanium-containing uranium minerals under FIB and TEM, including (A) Backscattered electron (BSE) image of titanium-containing uranium mineral under focused ion beam (FIB) scanning electron microscope; (B) Image of the transparent foil of titanium-containing uranium mineral under transmission electron microscope (The transparent foil was taken vertically downward at the yellow rectangular position in Figure A, with the thickness <100 nm); (C) HAADF-STEM image of titanium-containing uranium mineral; (D-I) STEM-EDS-derived area distribution of Ti-U(D), U(E), Ti(F), Ca(G) and Si(H) elements and energy spectra (I) of the black rectangular area in Figure C.

Besides coffinite and pitchblende, titanium-containing uranium minerals are also commonly found in the independent uranium minerals. This type of uranium mineral

usually fills among the clastic particles in the form of disseminated mineral aggregates (Fig 11A), or associated with chlorite (Fig 10E). Under the transmission electron microscope, it can be observed that the titanium-containing uranium mineral particles present a bright-white, complexly interwoven acicular aggregate morphology (Figs 11B, C). The micro-zone element area distribution image shows that this type of uranium-containing mineral is rich in Ti and U, with the distribution of the two elements being relatively uniform (Figs 11D-F). When combined with the micro-zone area-scanning element energy spectrum image (Fig 11I), it can be confirmed that this type of uranium mineral is a collection of nano-scale brannerite-like aggregate, rather than nanoscale coffinite or pitchblende associated with titanium oxides. The difference between the contents of TiO_2 and UO_2 in the brannerite-like is small and tends to be stable, with the average contents of TiO_2 and UO_2 being 33.20% and 39.62%, respectively. The contents of SiO_2, CaO and ZrO_2 in the brannerite-like are relatively high, with the average contents of SiO_2, CaO, and ZrO_2 being 6.48%, 1.74%, 2.88%, respectively. Additionally, a small amount of impurities such as Al_2O_3 and FeO also exist in the brannerite-like (Table 2).

4.4.2 Unclassified nano-uranium minerals~~Dispersed adsorbed uranium~~

~~The dispersed adsorbed uranium is actually the nano-scale uranium minerals adsorbed at the edges of clastic particles or filling in interstitial materials, whose mineral types cannot be confirmed by means of electron probe and scanning electron microscope.~~

Under scanning electron microscope, the nano-scale uranium minerals adsorbed in ~~the clay minerals (~~chlorite~~, chlorite/smectite mixed layer mineral)~~ are commonly observed (Figs 10F and 16), and the nano-scale uranium mineral are also commonly observed associated with loose and porous titanium oxides (Figs 10G, H). The uranium minerals are adsorbed at the edges or in the dissolution pores of this type of titanium oxides, and eventually formed a mixed mineral phase containing uranium and titanium ~~[32]~~, and occurring in the ~~sandbodies~~sandstones in the form of titanium-uranium "mixture". This titanium-uranium "mixture" phase mineral is rich in the sandstone-type uranium deposit in the southern Songliao Basin, and can be observed under a transmission electron microscope. It is ~~actually~~ the nanoscale uranium mineral (coffinite, pitchblende) adsorbed at the edges or in the dissolution pores of the titanium oxides ~~[33]~~, rather than the brannerite-like described in previous studies. The nano-scale uranium minerals~~The dispersed adsorbed uranium~~ is characterized by relatively high content of TiO_2, with a large difference in UO_2 contents. The content of UO_2 adsorbed in the pores or at the edges of the titanium oxides is relatively low, whereas the content of UO_2 adsorbed in ~~clay minerals (~~chlorite~~, chlorite/smectite mixed layer mineral)~~ is relatively high. The total chemical component amount of the nano-scale uranium minerals~~dispersed adsorbed uranium~~ ranges from 92.05% to 95.65%, with an average of 93.88%. Specifically, the contents of UO_2, TiO_2, FeO, SiO_2 range from 9.15% to 60.68%, 19.05% to 67.00%, 0.13% to 4.21% and 2.25% to 11.57%, respectively, averaging 31.98%, 45.82%, 2.33% and 7.41%, respectively; the average contents of CaO, Al_2O_3 and ZrO_2 are 1.80%, 1.67% and 1.13%, respectively. Additionally, the

Formatted: Font: (Default) Times New Roman, 12 pt

Formatted: Normal, Indent: First line: 2 ch, Line spacing: Double

Formatted: Font: (Default) Times New Roman, 12 pt

Formatted: Font: (Default) Times New Roman, 12 pt

Formatted: Font: (Default) Times New Roman, 12 pt

Formatted: Font: (Default) Times New Roman, 12 pt

nano-scale uranium mineralsthe dispersed adsorbed uranium also contains a small amount of impurities such as Y_2O_3 and P_2O_5 (Table 2).

Formatted: Font: (Default) Times New Roman, 12 pt

4.4.3 Isomorphism substitution mineral

The minerals such as uranium-rich rutile (Fig 10J) and monazite (Fig 10I) can be occasionally observed under the microscope in the uranium ore samples from the Louzhuangzi area. The distribution of uranium in these minerals is extremely uneven (Figs 10K, L). These uranium-containing minerals may have been formed through the isomorphism substitution of Ti^{4+} and REE^{3+} by uranium in the source rocks, and then occurred in the sandbodiessandstones as a result of source rock weathering and crushing, and sediment transportation and deposition. During the uranium mineralization process, uranium-rich rutile and monazite, due to the later-stage fluid alteration effect, can serve as potential uranium sources in depositional sandbodiessandstone units, and can promote the formation of uranium mineralization.

Table 2. Analysis results of chemical components in uranium-rich minerals in Louzhuangzi area by electron probe (Wt %).

Table 2. Analysis results of chemical components in uranium-rich minerals in Louzhuangzi area by electron probe (Wt %).

**Test point**	**SiO_2**	**Al_2O_3**	**MgO**	**Na_2O**	**Y_2O_3**	**CaO**	**P_2O_5**	**FeO**	**TiO_2**	**ZrO_2**	**PbO**	**MnO**	**UO_2**	**Total**	**Mineral type**
23ZGE022-01	18.23	0.22	0.03	0.11	0.50	3.88	1.26	0.09	0.12	0.09	0.05	0.02	66.44	91.03	coffinite
23ZGE022-02	9.33	0.99	0.19	0.11	0.64	4.30	2.12	0.46	2.86	4.43	0.11	0.15	66.88	92.57	coffinite
23ZGE022-03	16.39	1.24	0.19	0.01	0.92	4.88	2.79	0.62	0.28	7.24	0.05	0.16	61.58	96.36	coffinite
23ZGE022-04	11.54	0.06	0.05	0.07	0.60	4.42	3.51	1.47	0.58	3.13	0.05	0.07	66.71	92.26	coffinite
23ZGE028-01	15.98	1.59	0.14	0.03	0.97	4.49	2.60	0.20	0.30	7.17	0.01	0.11	58.27	91.85	coffinite
23ZGE028-02	13.27	1.08	0.13	0.05	0.72	4.26	2.65	0.30	1.73	6.75	0.05	0.01	59.24	90.23	coffinite
23ZGE028-03	14.33	1.21	0.05	0.21	4.93	7.21	5.91	0.21	0.07	0.98	0.00	0.12	54.04	89.27	coffinite
23ZGE028-04	15.09	0.05	0.49	0.03	7.17	0.62	1.31	1.06	0.00	1.16	0.09	0.00	60.54	87.61	coffinite
23ZGE026-01	14.28	0.05	0.39	0.08	6.21	0.65	1.06	2.66	0.00	1.28	0.06	0.02	60.99	87.72	coffinite
23ZGE026-02	16.20	0.28	1.87	0.07	7.33	0.83	1.24	1.00	0.00	0.49	0.18	0.00	58.07	87.56	coffinite
23ZGE026-01	16.54	5.38	0.19	0.17	4.69	3.88	5.53	0.89	0.10	2.22	0.01	0.00	52.89	92.50	coffinite
23ZGE026-03	14.26	0.24	0.09	0.03	3.24	3.39	5.30	4.59	0.21	0.08	0.08	0.00	65.62	97.14	coffinite
23ZGE026-04	11.71	0.20	0.02	0.07	3.20	3.76	4.68	4.56	0.08	0.17	0.09	0.00	65.70	94.23	coffinite
23ZGE026-05	12.42	0.19	0.07	0.01	3.96	2.67	5.25	3.51	0.07	0.05	0.06	0.06	65.86	94.16	coffinite

23ZGE026-06	16.46	1.68	0.21	0.08	4.36	4.21	6.44	1.84	0.48	0.82	0.00	0.06	60.37	97.01	coffinite
23ZGE026-07	13.00	0.39	0.06	0.02	5.03	1.27	3.21	1.33	3.49	1.39	0.20	0.02	58.12	87.51	coffinite
X̄ (averages)	14.31	0.93	0.26	0.07	3.40	3.42	3.43	1.55	0.65	2.34	0.07	0.05	61.33	91.81	
23ZGE028-05	4.34	0.02	0.03	0.19	0.26	3.45	0.36	0.33	1.88	0.69	0.06	0.36	82.87	94.83	pitchblende
23ZGE028-06	1.20	0.00	0.04	0.10	0.00	3.56	0.30	0.32	0.35	0.31	5.21	0.39	83.60	95.38	pitchblende
23ZGE028-07	5.76	1.72	0.24	0.27	0.00	2.79	0.17	0.92	0.64	1.16	0.02	0.47	75.50	89.66	pitchblende
23ZGE028-08	1.95	0.02	0.09	0.28	1.52	2.39	0.05	0.62	0.00	0.03	0.05	0.54	83.33	90.85	pitchblende
23ZGE028-09	2.75	0.00	0.07	0.55	1.81	1.21	0.30	0.45	0.00	0.00	0.00	0.19	84.25	91.58	pitchblende
23ZGE026-08	2.40	0.03	0.06	0.49	1.30	1.74	0.14	0.71	0.00	0.16	0.05	0.38	84.67	92.12	pitchblende
23ZGE026-09	1.73	0.01	0.05	0.26	1.18	1.86	0.08	0.60	0.00	0.05	0.01	0.46	86.07	92.33	pitchblende
23ZGE026-10	1.05	0.00	0.00	0.06	0.36	3.99	0.25	1.18	0.25	0.63	1.26	0.73	76.82	86.57	pitchblende
23ZGE026-11	1.11	0.00	0.00	0.09	0.36	3.55	0.24	1.04	0.57	0.56	1.47	0.67	77.06	86.72	pitchblende
23ZGE026-12	1.30	0.01	0.01	0.07	0.33	3.07	0.22	0.94	0.94	0.79	1.28	0.90	76.44	86.29	pitchblende
X̄ (averages)	2.36	0.18	0.06	0.24	0.71	2.76	0.21	0.71	0.46	0.44	0.94	0.51	81.06	90.63	
23ZGE027-01	6.30	0.83	0.16	0.09	0.02	2.19	0.16	0.41	33.49	2.23	0.01	0.32	39.81	86.01	Brannerite-like
23ZGE027-02	9.18	2.30	0.51	0.08	0.09	2.92	0.16	0.70	31.97	3.37	0.06	0.55	38.01	89.88	Brannerite-like

22ZGE027-03	6.88	0.83	0.06	0.30	0.03	1.07	0.17	2.05	31.67	2.64	0.00	0.24	40.40	86.33	Brannerite-like
22ZGE027-04	5.74	0.36	1.62	0.06	0.43	0.59	0.12	0.45	33.34	1.75	0.03	0.19	39.33	84.01	Brannerite-like
23ZGE026-13	6.62	1.06	0.23	0.06	0.07	3.03	0.16	0.54	32.67	3.43	0.00	0.63	38.72	87.22	Brannerite-like
23ZGE026-14	4.14	0.74	0.09	0.34	0.12	0.65	0.12	3.99	36.09	3.86	0.00	0.24	41.46	91.83	Brannerite-like
X̄ (averages)	6.48	1.02	0.45	0.16	0.13	1.74	0.15	1.36	33.21	2.88	0.02	0.36	39.62	87.55	
23ZGE026-15	8.69	2.37	0.34	0.22	0.15	1.43	0.07	4.21	67.00	0.91	0.05	0.22	9.15	94.81	
23ZGE026-16	4.25	1.07	0.31	0.20	0.00	2.86	0.27	2.43	21.24	2.10	0.00	0.23	60.68	95.65	Unidentified
23ZGE026-17	10.30	0.11	0.02	0.07	2.43	3.60	2.07	2.45	19.05	0.10	0.02	0.10	53.24	93.55	nano-uranium
23ZGE028-10	2.25	0.00	0.38	0.04	0.17	0.63	0.10	0.13	60.33	1.60	0.00	0.12	26.29	92.05	minerals
23ZGE028-11	11.57	4.80	0.82	0.14	0.01	0.46	0.03	2.42	61.47	0.93	0.06	0.09	10.55	93.34	
X̄ (averages)	7.41	1.67	0.37	0.13	0.55	1.80	0.51	2.33	45.82	1.13	0.03	0.15	31.98	93.88	

SiO_2	Al_2O_3	MgO	Na_2O	Y_2O_3	CaO	P_2O_5	FeO	TiO_2	ZrO_2	PbO	MnO	UO_2	Total	Mineral type
18.23	0.22	0.03	0.11	0.50	3.88	1.26	0.09	0.12	0.09	0.05	0.02	66.44	91.03	coffinite
9.33	0.99	0.19	0.11	0.64	4.30	2.12	0.46	2.86	4.43	0.11	0.15	66.88	92.57	coffinite
16.39	1.24	0.19	0.01	0.92	4.88	2.79	0.62	0.28	7.24	0.05	0.16	61.58	96.36	coffinite
11.54	0.06	0.05	0.07	0.60	4.42	3.51	1.47	0.58	3.13	0.05	0.07	66.71	92.26	coffinite
15.98	1.59	0.14	0.03	0.97	4.49	2.60	0.20	0.30	7.17	0.01	0.11	58.27	91.85	coffinite
13.27	1.08	0.13	0.05	0.72	4.26	2.65	0.30	1.73	6.75	0.05	0.01	59.24	90.23	coffinite
14.33	1.21	0.05	0.21	4.93	7.21	5.91	0.21	0.07	0.98	0.00	0.12	54.04	89.27	coffinite
15.09	0.05	0.49	0.03	7.17	0.62	1.31	1.06	0.00	1.16	0.09	0.00	60.54	87.61	coffinite
14.28	0.05	0.39	0.08	6.21	0.65	1.06	2.66	0.00	1.28	0.06	0.02	60.99	87.72	coffinite
16.20	0.28	1.87	0.07	7.33	0.83	1.24	1.00	0.00	0.49	0.18	0.00	58.07	87.56	coffinite
16.54	5.38	0.19	0.17	4.69	3.88	5.53	0.89	0.10	2.22	0.01	0.00	52.89	92.50	coffinite
14.26	0.24	0.09	0.03	3.24	3.39	5.30	4.59	0.21	0.08	0.08	0.00	65.62	97.14	coffinite
11.71	0.20	0.02	0.07	3.20	3.76	4.68	4.56	0.08	0.17	0.09	0.00	65.70	94.23	coffinite
12.42	0.19	0.07	0.01	3.96	2.67	5.25	3.51	0.07	0.05	0.06	0.06	65.86	94.16	coffinite
16.46	1.68	0.21	0.08	4.36	4.21	6.44	1.84	0.48	0.82	0.00	0.06	60.37	97.01	coffinite
13.00	0.39	0.06	0.02	5.03	1.27	3.21	1.33	3.49	1.39	0.20	0.02	58.12	87.51	coffinite
4.34	0.02	0.03	0.19	0.26	3.45	0.36	0.33	1.88	0.69	0.06	0.36	82.87	94.83	pitchblende
1.20	0.00	0.04	0.10	0.00	3.56	0.30	0.32	0.35	0.31	5.21	0.39	83.60	95.38	pitchblende
5.76	1.72	0.24	0.27	0.00	2.79	0.17	0.92	0.64	1.16	0.02	0.47	75.50	89.66	pitchblende
1.95	0.02	0.09	0.28	1.52	2.39	0.05	0.62	0.00	0.03	0.05	0.54	83.33	90.85	pitchblende

Formatted Table

2.75	0.00	0.07	0.55	1.81	1.21	0.30	0.45	0.00	0.00	0.00	0.19	84.25	91.58	pitchblende
2.40	0.03	0.06	0.49	1.30	1.74	0.14	0.71	0.00	0.16	0.05	0.38	84.67	92.12	pitchblende
1.73	0.01	0.05	0.26	1.18	1.86	0.08	0.60	0.00	0.05	0.01	0.46	86.07	92.33	pitchblende
1.05	0.00	0.00	0.06	0.36	3.99	0.25	1.18	0.25	0.63	1.26	0.73	76.82	86.57	pitchblende
1.11	0.00	0.00	0.09	0.36	3.55	0.24	1.04	0.57	0.56	1.47	0.67	77.06	86.72	pitchblende
1.30	0.01	0.01	0.07	0.33	3.07	0.22	0.94	0.94	0.79	1.28	0.90	76.44	86.29	pitchblende
6.30	0.83	0.16	0.09	0.02	2.19	0.16	0.41	33.49	2.23	0.01	0.32	39.81	86.01	brannerite
9.18	2.30	0.51	0.08	0.09	2.92	0.16	0.70	31.97	3.37	0.06	0.55	38.01	89.88	brannerite
6.88	0.83	0.06	0.30	0.03	1.07	0.17	2.05	31.67	2.64	0.00	0.24	40.40	86.33	brannerite
5.74	0.36	1.62	0.06	0.43	0.59	0.12	0.45	33.34	1.75	0.03	0.19	39.33	84.01	brannerite
6.62	1.06	0.23	0.06	0.07	3.03	0.16	0.54	32.67	3.43	0.00	0.63	38.72	87.22	brannerite
4.14	0.74	0.09	0.34	0.12	0.65	0.12	3.99	36.09	3.86	0.00	0.24	41.46	91.83	brannerite
8.69	2.37	0.34	0.22	0.15	1.43	0.07	4.21	67.00	0.91	0.05	0.22	9.15	94.81	
4.25	1.07	0.31	0.20	0.00	2.86	0.27	2.43	21.24	2.10	0.00	0.23	60.68	95.65	
10.30	0.11	0.02	0.07	2.43	3.60	2.07	2.45	19.05	0.10	0.02	0.10	53.24	93.55	Dispersed adsorbed uranium
2.25	0.00	0.38	0.04	0.17	0.63	0.10	0.13	60.33	1.60	0.00	0.12	26.29	92.05	
11.57	4.80	0.82	0.14	0.01	0.46	0.03	2.42	61.47	0.93	0.06	0.09	10.55	93.34	

4.4.3 Isomorphism substitution mineral

The minerals such as uranium-rich rutile (Fig 10J) and monazite (Fig 10I) can be occasionally observed under the microscope in the uranium ore samples from the Louzhuangzi area. The distribution of uranium in these minerals is extremely uneven (Figs 10K, L). These uranium-containing minerals may have been formed through the

isomorphism substitution of Ti^{4+} and REE^{3+} by uranium in the source rocks, and then occurred in the sandbodies as a result of source rock weathering and crushing, and sediment transportation and deposition. During the uranium mineralization process, uranium-rich rutile and monazite, due to the later-stage fluid alteration effect, can serve as potential uranium sources in depositional sandbodies, and can promote the formation of uranium mineralization.

4.5 Alteration features and redox interfaces

4.5.1 Interlayer oxidation

In the Wanjiayao mineral site, located northeast of the Louzhuangzi mineral area, brownish-yellow discoloration is clearly observed on outcrops within sandstones of the Upper Member of the Toutunhe Formation (J_2t^2) (Figs 12A, B). In addition, relatively large fragments of carbonaceous debris are occasionally present and show no obvious color change relative to the surrounding matrix (Fig 12C). Uranium anomalies are detected within brownish-yellow glutenite (Fig 12D), and gypsum layers beneath the glutenite locally display partial discoloration and textural alteration (Fig 12E). At the Hasa Tomb anomaly site, east of the Louzhuangzi mineral area, the sandstone of the Lower Member of the Toutunhe Formation (J_2t^1) exhibits heterogeneous brownish-yellow staining and mottled coloration (Fig 12F). Some stained surfaces are partially covered by Quaternary surficial deposits. After removal of the surficial cover, brownish-yellow staining remains clearly visible and is spatially associated with zones of elevated uranium mineralization (Fig 12G).In the Wanjiayao mineral site in the northeast direction of the Louzhuangzi mineral area, the brownish yellow oxidation

Formatted: Font: Italic

Formatted: Superscript

Formatted: Font: Italic

Formatted: Superscript

~~phenomenon in the sandstone of the Upper Member of the Toutuhhe Formation (J_2t^2) can be clearly observed on the outcrops (Figs 12A, B), and large pieces of carbonaceous debris not being oxidized can be occasionally found (Fig 12C). Uranium anomalies are discovered in the brownish-yellow glutenite (Fig 12D), and the incomplete oxidation of the gypsum layer is observed in sandbodied under the glutenite layer (Fig12E). In the Hasa tomb anomaly site in the east of the Louzhuangzi mineral area, the sandstone in the Lower Member of the Toutunhe Formation (J_2t^1) has undergone uneven oxidation (Fig 12F), with some surface oxidation sites being covered by quaternary floating soil. When the floating soil is moved away, the brownish-yellow oxidation marks can be clearly observed, accompanied by strong uranium mineralization (Fig 12G).~~ Also, in the east of the Louzhuangzi mineral area, an interlayer oxidation zone has been exposed in the Upper Member of the Toutunhe Formation (J_2t^2) by the ~~industrial~~economic uranium mineralization drill hole of ZK11-3, with the buried depth being about 40.9m, and multiple layers of gypsum being present in the oxidized ~~sandbody~~sandstone (Fig 12I). Except for the ~~sandbody~~sandstone exposed by the drill holes of ZK11-2 and ZK11-3, the ~~sandbodies~~sandstones exposed by the other holes drilled to the Toutunhe Formation (J_2t) are all grayish to grayish-white (Figs 7B, 8 and 9) and reducing ~~sandbodies~~sandstones, rich in carbonized plant detritus and pyrite (Figs 12J-L), but the residual traces of ilmenite oxidation and alteration can still be observed under the microscope (Figs 17A, B). ~~In the west the of the Louzhuangzi mineral area, the oxidation type belongs to the post-mineralization phreatic oxidation with a relatively weak oxidation effect [10].~~

Fig. 12 Epigenetic alteration characteristics of ~~sandbodies~~sandstones in Toutunhe Formation (J_2t), Louzhuangzi area, including (A-D) Sand-yellow oxidized ~~sandbody~~sandstone outcrop (A); medium to large planar cross bedding developed in the ~~sandbody~~sandstone (B); incompletely oxidized carbonized plant detritus (C) and layered gypsum (E) occasionally observed; uranium anomaly commonly observed in gravel-bearing coarse sandstone (D) of the Upper Member of the Toutunhe Formation (J_2t^2) at Wanjiayao mineralization site; (F-G) Incompletely oxidized gray residues in the sand-yellow oxidized ~~sandbody~~sandstone (F); uranium anomalies observed in the oxidized ~~sandbody~~sandstone (G) of the Lower Member of the Toutunhe Formation (J_2t^1) at Hasa tomb anomaly site; (H-I) Oxidized ~~sandbody~~sandstone (H) and multiple layers of gypsum (I) of the Upper Member of the Toutunhe Formation (J_2t^2) exposed by drill hole ZK11-3; (J) Laminated carbonized plant detritus and pyrite distribution observed in the grayish-white altered ~~sandbodies~~sandstones in the Lower Member of the Toutunhe Formation (J_2t^1) exposed by drill hole ZK 6-4; (K) Kaolin and pyrite nodules developed in the grayish-white altered ~~sandbodies~~sandstones in the Lower Member of the Toutunhe Formation (J_2t^1) exposed by drill hole ZK 12-3; (L) Laminated carbonized plant detritus and pyrite distribution exposed by drill hole ZK4-3 in depth of 758.24m.

Field geological investigation found that the interlayer oxidation zone in the Louzhuangzi mineral area extends in a belt-like pattern from the Hasa tomb anomaly site in the east of this mineral area continuously to the Wanjiayao, Liuhuang'gou and Qianshuihe mineralization sites, with the total length of the front line of the oxidation zone controlled by outcrops and drill holes being approximately 20km (Fig 14). The drill hole ZK11-2 reveals that the uranium mineralized ~~sandbody~~sandstone in the Lower

Member of the Toutunhe Formation (J_2t^1) is closely related to the oxidized ~~sandbody~~sandstone at the Hasa tomb anomaly site, with every ore layer, every uranium mineralized body, and every uranium anomaly exposed by this drill hole in the Lower Member of the Toutunhe Formation (J_2t^1) having the corresponding interlayer oxidation zone at the Hasa tomb anomaly site in east of this drill hole ~~[10]~~ (Fig 13). The field geological investigation and the analysis of the information revealed by the drill hole show that the uranium mineralization in the Louzhuangzi area is closely related to the interlayer oxidation zone, and specifically, it is mainly controlled by the interlayer oxidation of epigenetic oxygen- and uranium- containing fluids. The development of the interlayer oxidation zone is mainly controlled by the Haojiagou Uplift, with the front line of the oxidation zone being basically parallel to the Haojiagou Uplift zone (Fig 14).

Fig. 13 Profile of interlayer oxidation zones and uranium mineralization zones in ~~sandbodies~~sandstones of Toutunhe Formation (J_2t) in drill hole ZK 11-2 at Hasa tomb anomaly site (with sandstone top roof being leveled).

Fig. 14 Distribution of depositional facies and oxidation zones in the Lower Member of Toutunhe Formation (J_2t^1), Louzhuangzi area (modified from [13~~10~~]).

4.5.2 Clayification

The gray and grayish-white ~~sandbody~~sandstone alteration zone in the Lower Member of the Toutunhe Formation (J_2t^1) of the Louzhuangzi area (Figs 7B, 8, and 9) and the uranium mineralization zone are closely related in space. The ore-bearing ~~sandbodies~~sandstones are mostly glutenite in lithology, and has undergone relatively

Formatted: Font: 10.5 pt

strong clayification (Figs 8, 15 and 16). Large amounts of clay minerals have been identified in the ~~sandbodies~~sandstones occurring in the gray and grayish-white alteration zone by using a scanning electron microscope, with the lithologies being mainly kaolinite (Figs 15A-C), ~~chlorite/~~smectite ~~mixed layer mineral~~ (Figs 15D-F), and ~~rapidolite~~ chlorite (Figs 15G-I), followed by sericite (Figs 5B and 6C). Clay minerals have excellent adsorption capability and high surface activity, and can selectively adsorb the oxygen- and uranium- containing fluids migrating in the sandstone pores, leading to the enrichment and precipitation of uranium elements on the surface of clay minerals, and eventually forming uranium-rich flake-like and massive clay aggregates (Fig 16). ~~Therefore, clay minerals play a role of adsorption and precipitation interfaces during uranium mineralization process, are conducive to the formation and positioning of uranium mineralization distribution space [34], which verifies the genetic connection between clay minerals and uranium mineralization at the microscopic scale.~~

Fig. 15 Characteristics of clay minerals in ~~sandbodies~~sandstones in Lower Member of Toutunhe Formation (J_2t^1), Louzhuangzi area (BSE images), including (A) Intergranular layered kaolinite, ZK16-2, 23DJA-9, 705.70m; (B) Intergranular worm-like kaolinite, ZK16-2, 23 dJA-6, 654.60m; (C) Intergranular plate-like kaolinite, ZK12-3, 23 dJA-27, 641.70m; (D) Spherical ~~chlorite/~~smectite ~~mixed layer mineral~~ on the particle surface , ZK4-3, 23DJA-7, 710.40m; (E) Cellular ~~chlorite/~~smectite ~~mixed layer mineral~~ on the particle surface , ZK12-3, 23 DJA-15,871.50m; (F) The strawberry pyrite Encapsulated by flake-like ~~chlorite/~~smectite ~~mixed layer mineral~~, ZK12-2, 23DJA-4, 680.70m; (G-I) Intergranular flake-like chlorite aggregate, ZK16-2, 23DJA-9, 705.70m.

Fig. 16 Association of uranium minerals and clay minerals in ~~sandbodies~~sandstones in Lower

Member of Toutunhe Formation (J_2t^1), Louzhuangzi area (BSE images), including (A) Uranium mineral adsorbed in the chlorite/smectite mixed layer mineral, ZK3-3, 23ZGe027, 753.10m; (B) Uranium mineral associated with chloritized biotite, ZK4-2, 23ZGE026, 398.20m; (C) Uranium minerals associated with chlorite, ZK12-3, 23ZGE028, 798.50m.

4.5.3 Sulfurization

Titanium oxide particles are commonly observed in thin sections of the gray and grayish-white sandstones of the Lower Member of the Toutunhe Formation (J_2t^1). These particles display clear features of epigenetic alteration and are predominantly angular to sub-angular in morphology (Figs 17A, B). A smaller proportion exhibits circular to sub-circular shapes (Figs 17C, D), suggesting modification by epigenetic processes and indicating that these titanium oxides represent altered products of proximal deposits. Titanium oxide particles can be commonly observed in the slices of the gray and grayish-white sandbodies of the Lower Member of the Toutunhe Formation (J_2t^1) under the microscope, showing obvious characteristics of epigenetic alteration, and the morphology of the particles are mostly angular to sub-angular (Figs 17A, B), with a few being circular to sub-circular (Figs 17C, D) due to epigenetic alteration, indicating that these titanium oxides are altered products of the proximal deposits. The early deposited ilmenite oxides were eventually transformed to the combination assemblage of anatase and magnetite due to the iron exsolution in the epigenetic alteration process, with pyrite enveloped at the edge of the mineral assemblage (Figs 17A, B). The uranium minerals adsorbed at the edge of altered titanium oxides were eventually transformed to the uranium-bearing phases rich in iron, titanium, and sulfur [32], with the periphery

Formatted: Font: Italic

Formatted: Superscript

Formatted: Font color: Accent 1

Formatted: Font color: Accent 1

of these uranium-bearing phases commonly enveloped by gelatinous pyrite (Figs 17C, D). Under the effect of strong sulfurization, the ilmenite was metasomatized by pyrite, and formed the combination assemblage of anatase and pyrite, but the lattice twin structure of ilmenite still remains (Figs 17E, F). ~~The association and combination assemblage of the titanium oxide, uranium mineral, and pyrite indicates that the uranium-bearing sandbodies have undergone intense sulfurization process after uranium mineralization, resulting in the partial or complete replacement of organic matter, ilmenite oxides, hematite and limonite in the sandbodies by pyrite [35].~~

Fig. 17 Alteration characteristics of titanium oxides in ~~sandbodies~~sandstones in Lower Member of Toutunhe Formation (J_2t^1), Louzhuangzi area. (A-C) Anatase and magnetite mineral assemblage enveloped by gelatinous pyrite (reflected light); (D) uranium-bearing oxides enveloped by gelatinous pyrite; (E-F) Interwoven association of anatase and pyrite (BSE image).

In addition, the near surface oxidized ~~sandbodies~~sandstones are only exposed in the eastern of the Louzhuangzi mineral area by drill holes ZK11-2 and ZK11-3 along the trend of the uranium ore body. Within a range of 6km to the west of the Louzhuangzi mineral area, the target ~~sandbodies~~sandstones exposed by drill holes are all gray or grayish white (Figs 7B, 8, 9 and 18). The rock slice identification under the microscope show that the ore-bearing sandstones ~~has~~ have undergone intense sulfurization (Fig 17). Moreover, the petroleum geological information indicates that the oil and gas activities in the deep layers of the study area have been frequent, suggesting~~.~~ that the intense sulfurization alteration in the gray and grayish-white ~~sandbodies~~sandstones were highly likely formed by the reduction and bleaching effects due to the large amount of H_2S

and CH_4 gases released from the deep oil and gas leakage, which eventually determined the spatial distribution of uranium mineralization [15~~12~~].

Fig. 18 Schematic diagram of the B-B ' drill hole profile in Louzhuangzi area. The profile position is shown in Figure 1C (modified from [15-16~~12-13~~]).

5. Discussion

5.1 Depositional environment and facies interpretation

In the Upper Member (J_2t^2), thick mudstone intervals with massive bedding (Fig 3B) are consistent with deposition under low-energy conditions such as floodplains or abandoned channels, where sediment settling dominates under weak flow conditions [42]. Flaser bedding in fine sandstone (Fig 3C) suggests intermittent traction and suspension deposition under fluctuating flow, consistent with sandy sediment migration on channel bottoms [9, 43]. Horizontal bedding in mudstone and sandstone (Fig 3B) is typical of low-energy sheet-like sedimentation in floodplain or onlap settings where flow is weak and deposition is laterally extensive [44]. Parallel bedding in sandstone (Fig 3A) indicates deposition under a higher-flow regime with planar laminar flow conditions [45]. Small- to medium-scale planar cross-bedding (Figs 3C and 12B) reflects bedform migration and lateral sediment transport under persistent flow, compatible with a meandering-river setting characterized by relatively moderate hydrodynamic energy [46].

In the Lower Member (J_2t^1), the dominance of thick coarse sandstone, glutenite, and conglomerate (Fig 7B), together with the development of channel-fill, lag-deposit, and gravel-bar microfacies (Fig 8), indicates deposition in a braided-river system with

Formatted: Font: (Default) Times New Roman, 16 pt, Bold
Formatted: Heading 2
Formatted: Font: (Default) Times New Roman, 12 pt
Formatted: Indent: First line: 2 ch, Line spacing: Double
Formatted: Font: (Default) Times New Roman, 12 pt
Formatted: Font: (Default) Times New Roman, 12 pt, Subscript
Formatted: Font: (Default) Times New Roman, 12 pt, Italic
Formatted: Font: (Default) Times New Roman, 12 pt, Superscript
Formatted: Font: (Default) Times New Roman, 12 pt
Formatted: Font: (Default) Times New Roman, 12 pt
Formatted: Font: (Default) Times New Roman, 12 pt
Formatted: Font: (Default) Times New Roman, 12 pt
Formatted: Font: (Default) Times New Roman, 12 pt
Formatted: Font: (Default) Times New Roman, 12 pt
Formatted: Font: (Default) Times New Roman, 12 pt, Italic
Formatted: Font: (Default) Times New Roman, 12 pt, Superscript
Formatted: Font: (Default) Times New Roman, 12 pt

higher-energy channel processes. Large-scale planar cross-bedding in glutenite (Fig 3D) is consistent with downstream-aggrading braided flow and rapid sediment accumulation [47]. Trough cross-bedding in glutenite (Fig 3F) and massive-bedded conglomerate (Fig 3E) further suggest channel erosion and subsequent infill, typical of gravelly braided fluvial deposition under strong hydrodynamic conditions [48]. The sandy-dominated association at the base of the Lower Member, with climbing ripples and scour surfaces (Fig 8), is consistent with a sandy braided-river environment with moderate hydrodynamic energy.

The upward decrease in conglomerate and coarse sandstone and the increase in siltstone and fine sandstone from the Lower to the Upper Member [9], together with the appearance of multiple gypsum layers (Figs 12E, I), suggest an overall upward shift toward more arid conditions and reduced hydrodynamic energy, accompanied by progressive shallowing of the water body through time [49].

~~5.1~~5.2 Sandstone alteration conditions and the response to uranium mineralization~~Controls on uranium mineralization: sedimentary facies and diagenesis~~

Uranium mineralization in the Toutunhe Formation (J_2t) of the Louzhuangzi area is primarily controlled by braided channel sedimentary architecture and subsequent fluid-induced alteration. Matrix-rich braided channel lag deposits constitute the principal ore- bearing facies. These facies-controlled sandstones are characterized by high primary permeability and enhanced geochemical reactivity along grain–matrix interfaces, thereby providing favorable pathways for the migration of uranium-bearing

Formatted: Font: (Default) Times New Roman, 12 pt

Formatted: Font: (Default) Times New Roman, 12 pt

Formatted: Font: (Default) Times New Roman, 12 pt

Formatted: Normal, Indent: First line: 2 ch

Formatted: Font: (Default) Times New Roman, 12 pt

Formatted: Font: (Default) Times New Roman, 12 pt

Formatted: Font: 12 pt, Not Bold

Formatted: Font: Italic

oxidizing fluids and the development of localized reduction fronts [5, 50].

The ore-bearing sandstones exhibit relatively low compositional maturity and contain abundant unstable or labile components, including feldspar and lithic fragments, which are highly susceptible to chemical weathering and diagenetic alteration [51]. Although burial compaction reduced the initial porosity, reactive detrital grain boundaries were largely preserved, particularly in zones where feldspar and lithic fragments underwent alteration. Labile components—especially feldspar and volcanic or metamorphic lithic fragments—are prone to alteration under oxidizing or acidic conditions, releasing Ca, Na, and K, and potentially providing ion-exchange surfaces capable of temporarily adsorbing or immobilizing uranium during fluid migration [52]. Moreover, alteration of feldspar and lithic fragments may generate secondary porosity and authigenic clay minerals (e.g., kaolinite, montmorillonite, and chlorite), which can serve as adsorption sites for uranium or participate in redox buffering processes [53].

The braided river channel facies (especially lag deposits with gravel lenses and matrix-rich intervals) serve as primary hosts for uranium mineralization. These facies offer both structural porosity and geochemical reactivity. Diagenetic compaction reduced initial porosity but preserved reactive grain–matrix boundaries. Comparison with analogous sandstone-type uranium systems in the Ordos Basin [27], Songliao Basin [36] and Texas [37] reinforces the sedimentary–diagenetic control model.

5.25.3 Reduction–precipitation of uranium and superimposed transformationSandstone compositional maturity and its impact on uranium mobility

A close spatial association is observed between uranium minerals and pyrite, titanium oxides, and organic matter within the ore- bearing sandstones. Pyrite and organic matter act as effective reducing agents, forming localized redox-buffering zones capable of reducing mobile U^{6+} to immobile U^{4+} [16, 54-56]. Titanium oxides exhibit strong surface complexation capacity for U^{4+}. Microstructural and compositional evidence supports their dual role as both adsorption substrates and mineralization nuclei [57], and they may further promote recrystallization under fluctuating redox conditions and temperature variations [31, 58]. The recrystallized Ti–U mineral aggregates are interpreted as nanocrystalline brannerite-like phases. Although further confirmation by X-ray diffraction (XRD) is required, the brannerite-like interpretation is supported by nanoscale mineral structural evidence [31].

The Q/(F+R) ratio, where Q represents quartz, F represents feldspar, and R represents lithic fragments, is commonly employed as an indicator of compositional maturity in sandstones. In this study, the Q/(F+R) values of the Toutunhe Formation (J_2t) sandstones range from 0.22 to 1.44, with an average of 0.65, suggesting overall low compositional maturity. This implies that the sandstones contain a significant proportion of unstable or labile components (feldspar and lithic fragments), which are more susceptible to chemical weathering and diagenetic alteration.

The relatively low maturity of these sandstones has direct implications for uranium

mobility. Labile components, particularly feldspars and volcanic or metamorphic lithic fragments, are prone to alteration under oxidizing or acidic conditions, releasing elements such as Ca, Na, K, and potentially providing ion exchange surfaces that can temporarily adsorb or immobilize uranium during its transport [38]. Moreover, the alteration of feldspar and lithics can generate secondary porosity and clay minerals (e.g., kaolinite, illite, chlorite), which may act as uranium adsorption sites or participate in redox buffering.

Therefore, the presence of feldspar- and lithic-rich sandstones enhances the chemical reactivity of the rock matrix, creating favorable conditions for uranium fixation during fluid-rock interactions, especially in the presence of reducing agents such as organic matter, pyrite, or migrated hydrocarbons.

5.3 5.4 Fluid activity and conceptual modelRedox buffering role of pyrite and organic matter

Uranium mineralization in the Louzhuangzi area is closely related to interlayer oxidation driven by surficial oxygen-bearing, uranium-bearing fluids [13]. However, the occurrence of nano-scale brannerite-like aggregates and pronounced sulfidation features in the analyzed samples suggests that the grayish-white ore- bearing sandstones may have experienced superimposed transformation by thermal fluids. Under the influence of deep-seated reducing fluids, such as H_2S- or CH_4-bearing fluids, localized re-reduction of the sandstones likely occurred, thereby inhibiting the remobilization and oxidative destruction of uranium mineralization [20-21, 23-24, 31, 59-60]. Based on the current understanding, this study proposes a conceptual model of coupled

mineralization control involving braided fluvial sedimentary sandstones and multi-stage metallogenic fluid processes (Fig 19). This model highlights the coupling relationship between sandstone sedimentation, post-depositional alteration, and uranium mineralization. Nevertheless, due to the lack of fluid inclusion microthermometry and stable isotope constraints, the timing, temperature, and sources of the involved fluids remain to be further verified by future investigations.

Petrographic observations, SEM–EDS mappings, and EPMA point analyses indicate that pyrite and organic matter are abundant in the grayish-white alteration zones where uranium is preferentially enriched. The pyrite grains occur as framboidal clusters, anhedral masses, and rim-like aggregates surrounding detrital grains and clay-filled pores. Organic matter appears as diffuse films, vein-like bitumen, and particulate debris closely associated with uranium minerals such as pitchblende and coffinite.

The spatial and textural association between uranium minerals and these reductants suggests that both pyrite and hydrocarbons played important roles as redox buffers during uranium precipitation. Pyrite acts as an effective reducing agent by consuming dissolved oxygen or oxidized uranium species (e.g., U^{6+}), leading to the reduction and immobilization of uranium as U^{4+} minerals [39]. Organic matter, especially bitumen, may further stabilize, reducing microenvironments and contributing H_2S or CH_4 during thermal decomposition, which facilitates uranium reduction [35, 40–42].

Furthermore, the localized accumulation of uranium at clay–pyrite or bitumen–matrix interfaces implies that redox gradients at these boundaries were critical for

nucleation and growth of uranium-bearing phases. This is consistent with models proposed for other sandstone-type uranium deposits globally [41–43].

5.4 Timing and genetic stage of mineral alteration

The Toutunhe Formation (J_2t) exhibits two main types of mineral alteration: (1) chloritization, kaolinitization, and sericitization of feldspars and mafic grains, and (2) uranium-related alterations including clay–pyrite–bitumen assemblages and Ti–U oxide crystallization. These alteration styles are temporally distinct and reflect different diagenetic–epigenetic stages [44].

The early diagenetic alterations include partial chloritization of volcanic lithics and feldspar kaolinitization, which occur at relatively low temperatures during burial compaction and initial fluid interactions. These processes are widespread across both mineralized and barren zones, indicating background diagenesis [45].

In contrast, late-stage epigenetic alterations are spatially associated with uranium mineralization and are concentrated in grayish-white alteration zones. Features such as sericitization rims, framboidal pyrite, amorphous bitumen, and uranium-bearing clay–pyrite intergrowths — combined with FIB–TEM observations of brannerite-like nanoparticles — suggest fluid-mediated transformations during hydrocarbon or thermal fluid ingress.

Thus, distinguishing alteration paragenesis is critical: early diagenetic modifications set the lithochemical framework, while epigenetic fluid overprints are responsible for uranium concentration and ore-grade mineralization.

5.5 Geochemical indicators and mineralogical constraints

In this study, we applied Q/(F+R) ratios as indicators of compositional maturity; however, we recognize that additional geochemical proxies would significantly enhance the interpretive framework. Future work will focus on acquiring major and trace element concentrations (e.g., U, Ti, Fe, Al, Th, V), elemental ratios (e.g., Ti/Zr, Th/U, U/Fe), and rare earth element (REE) patterns to delineate provenance signals and hydrothermal overprints. Discrimination diagrams such as Harker plots and Th/U vs. U/Fe biplots will also be introduced to visualize alteration trends and uranium mobility.

Moreover, while SEM, EPMA, and FIB-TEM provided critical insights into the textural and chemical properties of uranium-bearing minerals, we acknowledge that the lack of X-ray diffraction (XRD) data limits the definitive identification of crystalline versus amorphous phases. In future analyses, XRD will be employed to confirm mineral identifications, particularly for fine-grained clay minerals and titanium–uranium oxides. This will ensure greater accuracy in distinguishing polymorphs such as anatase vs. brookite, and the presence of potentially amorphous or poorly crystalline brannerite phases.

5.6 Origin and nature of thermal fluids

Although this study proposes that thermal fluids have played a role in the superimposed alteration of uranium-bearing sandbodies, the precise origin, composition, and evolution of these fluids remain poorly constrained. Based on the regional tectonic and geothermal background, it is plausible that the thermal fluids were derived from deep basin-centered flow systems driven by compaction, tectonic

compression, or magmatic underplating beneath the Junggar Basin [13]. The presence of extensive clay mineral transformations, sulfurization features, and nanoscale brannerite aggregations observed in the altered grayish-white sandstones suggest fluid activity under moderately elevated temperatures. In addition, the association of brannerite with chlorite and chlorite–smectite mixed layers implies that the fluids were relatively reducing and carried dissolved uranium and titanium species under stable physicochemical conditions. However, due to the lack of direct fluid inclusion or isotopic data, the properties and pathways of these thermal fluids cannot be definitively identified.

5.7 Uranium mineralization model

This study systematically reveals the sedimentary characteristics of the Toutunhe Formation (J_2t) in the study area through integrated field geological investigation, drill core data compilation, and petrographic analysis, combined with microscopic observations of uranium mineralization and analyses of post-depositional alteration of the sandbodies. The coupling relationship between sandbody alteration and uranium mineralization is explored, and the uranium mineralization process is preliminarily reconstructed. The results suggest that uranium mineralization may have been jointly controlled by multiple factors, including gravel lag deposits in braided river channels, overprinting alteration by epigenetic interlayer oxidation and thermal fluids, and reduction by hydrocarbons (Fig 19).

Fig. 19 Schematic diagram of the uranium mineralization model in the Louzhuangzi area, including (A) Types of mineral alteration in different fluid environments; (B) Interlayer oxidizing fluid mineralization; (C) Thermal fluid superposition transformation with oil and gas reduction.

6. Conclusions and implications for uranium exploration

(1) Uranium mineralization in the Louzhuangzi area is predominantly developed within thick, coarse-grained braided channel sandstones of the lower member of the Toutunhe Formation (J_2t^1).

(2) Pyrite, titanium oxides, clay minerals, and organic matter are spatially associated with uranium minerals, acting as local reducing agents and/or adsorption sites that facilitate the reduction of U^{6+} to U^{4+} and subsequent precipitation.

(3) Uranium mainly occurs in the form of pitchblende, coffinite, and Ti–U aggregates, including brannerite-like nano-scale particles.

(4) The alteration of the ore-bearing sandstones reflects at least three stages of fluid activity: (i) interlayer oxidation by surficial oxygen-bearing, uranium-rich fluids; (ii) post-mineralization superimposed transformation by thermal fluids; and (iii) localized reduction induced by hydrocarbon influx.

This study reveals that uranium mineralization in the Toutunhe Formation (J_2t) of the Louzhuangzi area is jointly controlled by braided river channel lag deposits, multi-stage epigenetic fluid alteration, and localized reduction by hydrocarbon-rich fluids. The key findings can be summarized as follows:

(1) Sedimentary Control: Uranium-bearing sandstones are primarily developed in

Formatted: Font: Italic

Formatted: Superscript

thick, coarse-grained braided river channel facies. These facies offer high permeability and favorable accommodation for fluid migration.

(2) Multi-stage Alteration: Petrographic and TEM observations demonstrate at least three stages of fluid interaction: (i) early diagenetic chloritization/kaolinitization, (ii) interlayer oxidation during epigenesis, and (iii) localized reduction due to hydrocarbon influx and thermal fluid overprint.

(3) Uranium Mineral Assemblages: Uranium predominantly occurs as pitchblende, coffinite, and Ti–U aggregates (including brannerite-like nanoparticles), indicating variable physicochemical conditions during mineralization.

(4) Redox Buffering: Pyrite and amorphous bitumen commonly coexist with uranium minerals and act as local reducing agents, promoting U^{6+} to U^{4+} reduction and precipitation.

These findings have important implications for uranium exploration in the Junggar Basin and analogous basins: (i) Priority should be given to regions where braided river channels intersect with hydrocarbon migration pathways or faults; (ii) Attention should be paid to grayish-white alteration zones with abundant pyrite and organic matter; (iii) Targeting Ti–U mineral assemblages and interpreting their alteration features can help infer fluid history and guide ore prediction.

Corresponding Author

Fengjun Nie — School of Earth Sciences, East China University of Technology, Nanchang, Jiangxi, China; Email: niefj@263.net.

Author Information

Qing Wang — School of Earth Sciences, East China University of Technology, Nanchang, Jiangxi, China; Email: destinw@126.com.

Author Contributions

Conceptualization: Qing Wang, Fengjun Nie.

Data curation: Qing Wang, Xin Zhang.

Formal analysis: Fengjun Nie, Qing Wang, Xin Zhang.

Funding acquisition: Fengjun Nie, Dong-guang Yang.

Investigation: Qing Wang, Fengjun Nie, Xin Zhang, Weiwei Jia, Kegai Lu, Xiao Sun.

Methodology: Fengjun Nie, Kegai Lu.

Project administration: Fengjun Nie, Fei Xia.

Resources: Fengjun Nie, Weiwei Jia, Kegai Lu, Xiao Sun.

Writing – original draft: Qing Wang.

Writing – review & editing: Qing Wang, Fei Xia.

Data Availability

All data generated or analyzed during this study are attached.

Declaration of Competing Interest

The authors declare that they have no known competing financial interests or personal relationships that could have appeared to influence the work reported in this

paper.

Funding Projects: In this study, the field geological investigation and sample preparation were financially supported by the National Natural Science Foundation of China (No. U2244205 and No. U2067202). The laboratory analytical work was supported by the Joint Innovation Fund Project of China Uranium Industry Company Limited – State Key Laboratory of Nuclear Resources and Environment, East China University of Technology (2023NRE-LH-10).

Acknowledgments: We thank the PLOS ONE editors for their support, all contributors to fieldwork and laboratory analyses, and the reviewers for their constructive suggestions that significantly enhanced this manuscript.

Images of the author's field geological survey and laboratory analysis work.

Formatted: Font: Bold, (Asian)

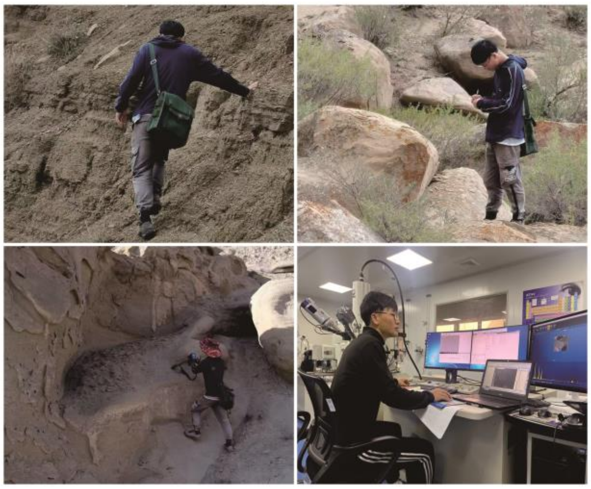

Formatted: Font: Bold, (Asian)

Formatted: Centered

References

1. Cai, Y. Q., Zhang, J. D., Li, Z. Y., Guo, Q. Y., Song, J. Y., Fan, H. H., Liu, W. S., Qi, F. C., Zhang, M. L. 2015. Outline of uranium resources characteristics and metallogenetic regularity in China. Acta Geologica Sinica 89(6):1051–1069. https://kns.cnki.net/KCMS/detail/detail.aspx?dbcode=CJFQ&dbname=CJFDLAST2015&filename=DZXE201506005. [in Chinese]
2. Zhang, J. D. 2016. Innovation and development of metallogenic theory for sandstone type uranium deposit in China. Uranium Geology 32(6):321–332. doi: 10. 3969/j issn 1000-0658. 2016. 06. 001. [in Chinese]
3. Geng, Y. Y., Liu, Z. Y., He, Z. B., Huang, S. H., Guo, Q., Xu, Q., Song, J. Y. 2020. Provenance and ore-forming tectonic setting of sandstone-type uranium deposits in the southern margin of Junggar Basin——Insights from U-Pb ages and Hf isotopes of detrital zircons. Geological Review 66(2):393–409. doi: 10.16509/j.georeview.2020.02.010. [in Chinese]
4. Cheng, Y. H., Jin, R. S., Cuney, M., Petrov, V. A., Miao, P. S. 2024. The strata constraint on large scale sandstone-type uranium mineralization in Meso-Cenozoic basins, northern China. Acta Geologica Sinica 98(7):1953–1976. doi: 10.19762/j.cnki.dizhixuebao.2023299. [in Chinese]
5. Huang, S. J. 1994. Formation conditions and prospecting criteria for sandstone uranium deposits of interlayered oxidation type. Uranium Geology (1):6–13. https://kns.cnki.net/KCMS/detail/detail.aspx?dbcode=CJFQ&dbname=CJFD9495&filename=YKDZ401.001. [in Chinese]
6. Chen, Z. Y. 2002. Regional distribution regularity of sandstone uranium deposits in Asian continent and prospecting strategy for sandstone uranium deposits in China. Uranium Geology (3):129–137. https://kns.cnki.net/KCMS/detail/detail.aspx?dbcode=CJFQ&dbname=CJFD2002&filename=YKDZ200203000. [in Chinese]
7. Wang, G. 2002. Mountain–basin formation and sandstone-type uranium

mineralization in Xinjiang. Xinjiang Geology 20(2):110–114. https://kns.cnki.net/KCMS/detail/detail.aspx?dbcode=CJFQ&dbname=CJFD2002&filename=XJDI200202006. [in Chinese]

8. Chen, D. S., Li, S. X., Cai, Y. Q. 2006. Overview of the researches on sedimentary environment for sandstone-type uranium deposits in the Mesozoic–Cenozoic basins of China. Acta Sedimentologica Sinica 24(2):223–228. https://kns.cnki.net/KCMS/detail/detail.aspx?dbcode=CJFQ&dbname=CJFD2006&filename=CJXB200602008. [in Chinese]

9. Wang, G.R., Jia, W. W., Du, M., Huang, S., Lu, K.G., Sun, X. 2021. Relationship between jurassic braided river depositional system and uranium mineralization in the Southern Margin of Junggar Basin. Uranium Geology 37(3) :342–54. doi: 10.3969/j.issn.1000-0658.2021.37.037. [in Chinese]

10. Geng Y.Y, Liu Z.Y, Huang S.H, He Z.B. Metallogenic environment and prospecting direction of sandstone-type uranium deposits in the Toutunhe area, southern margin of Junggar Basin. Uranium Geology. 2022;38(2):194–206. https://kns.cnki.net/KCMS/detail/detail.aspx?dbcode=CJFQ&dbname=CJFDLAST2022&filename=YKDZ202202004. [in Chinese]

11. Wu Y.P. The uranium mineralization characteristics of the Liuhuang'gou area in the southern margin of Junggar Basin [dissertation]. Beijing: China University of Geosciences (Beijing); 2014. [in Chinese]

12. Chen, Z.L., Lu, K.G., Wang, G., Chen, B.L., Wang, G.R., Zheng, E.J., Cui, L.L., Ding, W.J. 2010. Characteristics of Cenozoic structural movements in southern margin of Junggar basin and its relationship to the mineralization of sandstone-type uranium deposits, Acta Petrologica Sinica, 26(2): 457-470. https://kns.cnki.net/KCMS/detail/detail.aspx?dbcode=CJFQ&dbname=CJFD2010&filename=YSXB201002012. [in Chinese]

13. Lu, K.G., Du, M., Sun, X., Jia, W. W., Wang, S.Y. 2023. Metallogenic controlling factors and genetic analysis of sandstone uranium deposit in the Lower Member of Toutunhe Formation in Louzhuangzi area, Southern Junggar Basin. Uranium Geology 39(4): 507–21. doi: 10.3969/j.issn.1000-0658.2023.39.043. [in Chinese].

14. Huang, S., Jia, W. W., Yan, J. J. 2023. Discussion on the relationship between sand body characteristics and uranium mineralization in the Lower Member of Toutunhe Formation in Louzhuangzi area, Southern Margin of Junggar Basin. Uranium Geology (4): 522–32. doi:10.3969/j.issn.1000-0658.2023.39.044. [in Chinese]

15. Jia W.W., Wang, G.R., Tang, X.F., Huang, S., Yan, J. J. 2023. Discussion on the relationship of different types of alteration zones to the uranium mineralization in Toutunhe Formation of Louzhuangzi area, Southern Margin of Junggar Basin. World Nuclear Geoscience 40(2):152–61. doi:10.3969/j.issn.1672-0636.2023.02.002. [in Chinese]

16. He, Z.B., Hu, B.Q., Qiu, L.F., Wang, Y., Chen, H., Jia, W.W., Li, Y.F., Ji, H.L., Zhu, M.H. 2024. The role of hydrocarbons in the formation of uranium mineralization, Louzhuangzi district, Southern Junggar Basin (China). Minerals 14(7):709. doi: 10.3390/min14070709.

17. Yang, Z. H., Dong, Y., Liu, C., Liu, M., Zhu, Y. S., Zhou, Y. 2022. Study on the occurrence state of uranium in the Toutunhe Formation of Middle Jurassic in Liuhuang'gou region, southern margin of Junggar Basin. Uranium Geology 38(6):1032–1042. doi:10.3969/j.issn.1000-0658.2022.38.091. [in Chinese]

18. Finch, W. I. 1967. Geology of epigenetic uranium deposits in sandstone in the United States. U.S. Geological Survey Professional Paper 538, 121 p. doi: 10.3133/pp538.

19. Reynolds, R. L., Goldhaber, M. B. 1978. Origin of a South Texas roll-type uranium deposit: I. Alteration of iron-titanium oxide minerals. Economic Geology 73(8):1677–1689. doi: 10.2113/gsecongeo.73.8.1677.

20. Nie, F. J., Yan, Z. B., Xia, F., He, J. F., Zhang, C. Y., Feng, Z. B., Zhang, X., Yang, D. G., Chen, M. Y., Tan, S. J., Zhang, J., Kang, S. H., Ning, J., Yang, J. X., Shen, K. F., Cai, J. F. 2021. Two-stage and two-mode uranium mineralization for sandstone-type uranium deposits. Acta Geoscientica Sinica 42(6):823–848. doi: 10.3975/cagsb.2021.112201. [in Chinese]

21. Yan, Z. B., Zhang, W. W., Zhang, C. Y., Xia, F., Nie, F. J., Wu, X. Y. 2023. The

influence of fluid activity from deep basin on the metallogenic process of sandstone type uranium deposits. Acta Geologica Sinica 97(12):4131–4149. doi: 10.19762/j.cnki.dizhixuebao.2023169. [in Chinese]

22. Liu, Y., Zhao, J. H., Jiao, Y. Q., Liu, Z. R., Zhou, M. F. 2023. Constraints of in-situ S-isotopic compositions of pyrite on the genesis of the Bayinqinggeli sandstone-hosted uranium deposit, Ordos Basin, northern China. Ore Geology Reviews 161:105608. doi: 10.1016/j.oregeorev.2023.105608.

23. Liu, C. Y., Zhang, L., Huang, L., Wu, B. L., Wang, J. Q., Zhang, D. D., Tan, C. Q., Ma, Y. P., Zhao, J. S. 2024. Novel metallogenic model of sandstone-type uranium deposits: Mineralization by deep organic fluids. Earth Science Frontiers 31(1):368–383. doi: 10.13745/j.esf.sf.2024.1.7. [in Chinese]

24. Sun, D., Li, H. M., Xia, F., Nie, F. J., Huang, G. W., Zhang, Z. B., Meng, F. M., Pan, J. Y., Hu, Y. J. 2024. Provenance and tectonic setting of the Lower Cretaceous Huanhe Formation in the northwestern Ordos Basin and its implications for uranium mineralization. ACS Omega 9(3):3324–3341. doi: 10.1021/acsomega.3c06163.

25. Nie, F. J., Yan, Z. B., Xia, F., Li, M. G., Lu, Y. Y., Cai, J. F., Guo, F. N., Ning, J. 2017. Hot fluid flows in the sandstone-type uranium deposit in the Kailu basin, Northeast China. Geological Bulletin of China 36(10):1850–1866. [in Chinese]

26. Qin, M. K., Huang, S. H., Liu, J. L., Liu, Z. Y., Guo, Q., Jia, L. C., Jiang, W. J. 2021. Hydrothermal alteration and its superimposed enrichment for the Qianjiadian tabular-type uranium deposit in the southwestern Songliao Basin. Minerals 12(1):52. doi: 10.3390/min12010052.

27. Li, Z. Y., Liu, W. S., Li, W. T., Li, X. D., Qin, M. K., Cai, Y. Q., Zhang, Y. L., et al. 2022. Exudative metallogeny of the Hadatu sandstone-type uranium deposit in the Erlian Basin, Inner Mongolia. Geology in China 49(4):1009–1047. doi: 10.12029/gc20220401. [in Chinese]

28. Liu, B., Hao, P., Zhang, Z. L., Guo, X. J., Zhang, P. F., Shi, Z. Q. 2023. Geology, alteration system and uranium mineralization of Tamusu large uranium deposit, China. Ore Geology Reviews 158:105488. doi: 10.1016/j.oregeorev.2023.105488.

29. Liu, W. S., Li, Z. Y., Liu, C. H., Ji, H. W., Li, X. D. 2024. Exudative metallogeny and model of Saihan Gaobi sandstone-type uranium deposit in Erlian Basin. Uranium Geology 40(1):129–142. Doi: 10.3969/j.issn.1000-0658.2024.40.011. [in Chinese].

30. Tang, C., Xu, Z. L., Duan, M., Meng, L. S., Liu, H. J., Wei, J. L., Zhang, C., Zhao, L. J. 2025. Genetic model of the Luhai sandstone-type uranium deposit in the Erlian Basin, Inner Mongolia. Minerals 15(3):294. doi: 10.3390/min15030294.

31. Wang, Q., Nie, F. J., Yang, Y. P., Xian, H. Y., Xia, F., Yan, Z. B., Zhang, X., Yang, D. G., Jia, W. W., Meng, F. M., Sun, D., Yan, J. J., Huang, S. 2025. Brannerite identification in the Louzhuangzi sedimentary U deposit and implications for deposit genesis or post-ore alteration. Ore Geology Reviews 106908. doi: 10.1016/j.oregeorev.2025.106908.

32. Hu, X. W., Yang, X. Y., Ren, Y. S., Wu, Z. J., Du, G. F., Huang, T. T. 2020. Controls of sedimentary environment and tectonic evolution on sandstone-type uranium mineralization in the Junggar Basin. Geotectonica et Metallogenia 44(4):725–741. doi: 10.16539/j.ddgzyckx.2020.04.013. [in Chinese]

33. Wu, Z.H., Jiang, W., Zhou, J.R., Li, J.S. 2001. Thermal-chronological dating on the thermal history of plutons and tectonic-landform evolution of the central tibetan plateau. Acta Geologica Sinica (4):468–76. [in Chinese]

34. Fang, S.H., Guo, Z. J., Wu, C. D., Zhang, Z. C., Wang, M. N., Yuan, Q. D. 2006. Jurassic clastic composition in the Southern Junggar Basin, Northwest China: implications for basin-range pattern and tectonic attributes. Acta Geologica Sinica (2):196–209. [in Chinese]

35. Fang, S.H., Song, Y., Jia, C.Z., Guo, Z.J., Zhang, Z.C., Liu, L.J. 2007. Timing of Cenozoic intense deformation at the north margin of Tianshan and its implications for petroleum accumulation. Earth Science Frontiers 14(2) :205-214. [in Chinese]

36. Han, B.F., Guo, Z.J., Zhang, Z.Ch., Zhang, L.Z., Chen, J.F., Song, B. 2010. Age, geochemistry, and tectonic implications of a late Paleozoic stitching pluton in the North Tian Shan suture zone, western China. GSA Bulletin 122(3–4):627–40. doi: 10.1130/B26491.1.

37. Li, W.H., Xu, G. Z., Quan, J. P. 2010. Late modification of the intermountain basins of the Tien Shan orogenic belt and sandstone-type uranium mineralization. Mineral Deposits 29(S1):141–42. doi: 10.16111/j.0258-7106.2010.s1.081. [in Chinese]
38. Xiao, X.C., Tang, Y.Q., Wang, J., Gao, J., Zhao, M. 1994. Blueschist belts and their tectonic implications of the S. Tianshan Mts., N. W. China. Acta Geoscientia Sinica (Z2). [in Chinese]
39. Guan, X.T., Wu, C.D., Wu, J., Zhou, J.Q., Jiao, Y., Zhou, R., Yu, Q.S. 2020. Sedimentary sequence and depositional environment evolution of Upper Jurassic-Lower Cretaceous strata in the Southern Margin of Junggar Basin. Xinjiang Petroleum Geology 41(1):67–79. doi: 10.7657/XJPG20200109. [in Chinese]
40. Wang, D.Y., Fu, Y.Q., 1981. Uranium Mineralogy (in Chinese). Atomic Energy Press, Beijing, p. 109.
41. Folk， R.L. 1968. Petrology of Sedimentary Rocks[Z]. U.S.A: Hemphill Publishingc，Austin，1968:170.
42. Yin, Z.Q., Qin, X. G., Wu, J. S., Ning, B. 2009. The multimodal grain-size distribution characteristics of loess desert lake and river sediments in some areas of Northern China. Acta Sedimentologica Sinica 27(2):343–51. Doi: 10.14027/j.cnki.cjxb.2009.02.009. [in Chinese]
43. Wang H.W. Sedimentary microfacies and evolutionary simulation of a meandering river in the NmIII oil group, Yangerzhuang Oilfield [dissertation]. Qingdao: China University of Petroleum (East China); 2024. [in Chinese]
44. Tang, Y.N., Hu, C.L., Dan, S.H., Han, C.C., Liu, Z.M. 2022. Depositional model for the early triassic braided river delta and controls on oil reservoirs in the eastern Junggar Basin, Northwestern China. Minerals 12(11):1409. doi: 10.3390/min12111409.
45. Liu, S., Li, J.B., Jin, F.B., Liu, H.F., Wang, M., Cheng, Y.L., Zhong, X.H., Lu, H. 2024. Genesis of efficient reservoirs in mixed sedimentary setting: A case study of Benxi Formation in the Gaoqiao area, Ordos Basin. Turkish Journal of Earth Sciences 33(2):118–45. doi: 10.55730/1300-0985.1903.
46. Wang, J., Gao, X. J., Zhou, X. M. 2019. Reservoir achitecture characterization and

remaining oil distribution of meandering river. Journal of China University of Petroleum 43(3):13–24. [in Chinese]

47. Zhu, Z.J., Huang, B.H., Guo, F.S., Zheng, H.F., Jiang, Y.B. 2012. Cretaceous braided river facies sediments and Danxia landform development characteristics in Longhushan World Geopark, Jiangxi. Acta Geoscientia Sinica 33(3):379–87. doi: 10.3975/cagsb.2012.03.12. [in Chinese]
48. Liu, Y.M., Chen, R.X., Zhao, D.D., Zhang, Z.Y., Wang, J., Hou, J.G. 2022. Sedimentary characteristics of gravelly braided rivers of the Lower Shihezi Formation in the Jin 72 well area, Dongsheng gas field. Petroleum Science Bulletin 7(4):457–74. doi: 10.3969/j.issn.2096-1693.2022.04.040. [in Chinese]
49. Jolivet, M., Bourquin, S., Heilbronn, G., Robin, C., Barrier, L., Dabard, M.P., Jia, Y.Y., De Pelsmaeker, E., Fu, B.H. 2017. The Upper Jurassic–Lower Cretaceous alluvial-fan deposits of the Kalaza Formation (Central Asia): Tectonic pulse or increased aridity? Geological Society, London, Special Publications 427(1):491–521. doi: 10.1144/SP427.6.
50. Jiao, Y. Q., Wu, L. Q., Rong, H., Zhang, F., Le, L., Song, H., Tao, Z. P., Peng, H., Sun, Y. H., Xiang, Y. 2022. Sedimentation, diagenesis and uranium mineralization: innovative discoveries and cognitive challenges in study of sandstone-type uranium deposits in China. Earth Science 47(10):3580–3602. https://doi.org/10.3799/dqkx.2022.284. [in Chinese]
51. Peng, S. L., Tian, J. P., Guo, X. Y., Jin, Y. P., Sun, B., Dou, H. X., Zhang, F., Hao, J., Hu, Y. L., Chen, L., Xu, W., Tong, Y. 2023. Characteristics and metallogenesis of ore-bearing sandstone in the Tarangaole sandstone type uranium deposit, Ordos Basin. Geology in China 50(1):264–276. doi: 10.12029/gc20200814001. [in Chinese]
52. Castillo-Oliver, M., Melgarejo, J. C., Torró, L., Villanova-de-Benavent, C., Campeny, M., Díaz-Acha, Y., Amores-Casals, S., Xu, J., Proenza, J., & Tauler, E. 2020. Sandstone-Hosted Uranium Deposits as a Possible Source for Critical Elements: The Eureka Mine Case, Castell-Estaó, Catalonia. Minerals, 10(1), 34. https://doi.org/10.3390/min10010034.

53. Song, H., Ni, S. J., Hou, M.C., Zhang, C.J., Shi, Z.Q., Wang, G., Yang, B., Hu, Y., Chen, Y.J. 2016. The characteristic of clay minerals in sandstone-type uranium deposit in the Yili Basin, NW China and its relationship with uranium mineralization. Acta Geologica Sinica 90(12):3352–66. http://www.geojournals.cn/dzxb/ch/index.aspx. [in Chinese]

54. Spirakis, C. S. (1996). The roles of organic matter in the formation of uranium deposits in sedimentary rocks. Ore Geology Reviews, 11(1), 53 ~ 69. https://doi.org/10.1016/0169-1368(95)00015-1.

55. Bonnetti, C., Cuney, M., Michels, R., Truche, L., Malartre, F., Liu, X., Yang, J. 2015. The multiple roles of sulfate-reducing bacteria and Fe-Ti oxides in the genesis of the Bayinwula roll front-type uranium deposit, Erlian Basin, NE China. Economic Geology 110(4):1059–81. doi: 10.2113/econgeo.110.4.1059.

56. Cumberland, S. A., Evans, K., Douglas, G., de Jonge, M., Fisher, L., Howard, D., & Moreau, J. W. (2021). Characterisation of uranium-pyrite associations within organic-rich Eocene sediments using EM, XFM-µXANES and µXRD. Ore Geology Reviews, 133, 104051. https://doi.org/10.1016/j.oregeorev.2021.104051.

57. Yin, S., Yan, Z.B., Fu, J.L., Zhang, W., Liu, H., Xia, F., Wang, Q.F. 2024. Polymorphic transformations of titanium oxides contribute to economic uranium mineralization in sandstone. Geology. doi: 10.1130/G51982.1.

58. Chen, R., Siidra, O. I., Firsova, V. A., Arevalo-Lopez, A., Colmont, M., Ugolkov, V. L., Bocharov, V. N. 2023. The chemistry, recrystallization and thermal expansion of brannerite from Akchatau, Kazakhstan. Materials 16(4):1719. doi: 10.3390/ma16041719.

59. Hall S.M, van Gosen B.S, Zielinski R.A. Sandstone-hosted uranium deposits of the Colorado Plateau, USA. Ore Geology Reviews. 2023;155:105353. doi:10.1016/j.oregeorev.2023.105353.

60. Qiu, L. F., Li, Z. Y., He, F., Wu, Z. Q., Liu, K. P., Zhang, L., Mao, N., Wang, T. T., Wang, L. 2025. Contribution of deep organic fluids to uranium mineralization in Cretaceous sandstones of the southwestern Ordos Basin, China. Hydrogeology

Journal 33(4):925–944. doi: 10.1007/s10040-025-02912-4.

1. Geng Y.Y, Liu Z.Y, He Z.B, Huang S.H, Guo Q, Xu Q, et al. Provenance and ore-forming tectonic setting of sandstone-type uranium deposits in the southern margin of Junggar Basin—Insights from U–Pb ages and Hf isotopes of detrital zircons. Geological Review. 2020;66(2):393–409. doi:10.16509/j.georeview.2020.02.010.
2. Huang S.J. Formation conditions and prospecting criteria for interlayer oxidation sandstone-type uranium deposits. Uranium Geology. 1994;(1):6–13. [in Chinese]
3. Chen Z.Y. Regional distribution of sandstone-type uranium deposits in Asia and prospecting strategy for China. Uranium Geology. 2002;(3):129–37. [in Chinese]
4. Wang G. Mountain–basin formation and sandstone-type uranium mineralization in Xinjiang. Xinjiang Geology. 2002;(2):110–14. [in Chinese]
5. Chen D.S, Li S.X, Cai Y.Q. Overview of sedimentary environments of sandstone-type uranium deposits in the Meso – Cenozoic basins of China. Acta Sedimentologica Sinica. 2006;(2):223–28. [in Chinese]
6. Wang G.R, Jia W.W, Du M, Huang S, Lu K.G, Sun X. Relationship between Jurassic braided river depositional system and uranium mineralization in the southern margin of Junggar Basin. Uranium Geology. 2021;37(3):342–54. [in Chinese]
7. Geng Y.Y, Liu Z.Y, Huang S.H, He Z.B. Metallogenic environment and prospecting direction of sandstone-type uranium deposits in the Toutunhe area, southern margin of Junggar Basin. Uranium Geology. 2022;38(2):194–206. [in Chinese]
8. Wu Y.P. The uranium mineralization characteristics of the Liuhuang'gou area in the southern margin of Junggar Basin [dissertation]. Beijing: China University of Geosciences (Beijing); 2014. [in Chinese]

Formatted: Font: 12 pt, Not Bold

Formatted: List Paragraph, Line spacing: 1.5 lines, Numbered + Level: 1 + Numbering Style: 1, 2, 3, … + Start at: 1 + Alignment: Left + Aligned at: 0" + Indent at: 0.31", Don't allow hanging punctuation

9. Chen Z.L, Lu K.G, Wang G, Chen B.L, Wang G.R, Zheng E.J, et al. Characteristics of Cenozoic structural movements in the southern margin of the Junggar Basin and their relationship to sandstone-type uranium mineralization. Acta Petrologica Sinica. 2010;26(2):457–70. [in Chinese]

10. Lu K.G, Du M, Sun X, Jia W.W, Wang S.Y. Metallogenic controls and genetic analysis of sandstone uranium deposits in the Lower Member of the Toutunhe Formation, Louzhuangzi area, southern Junggar Basin. Uranium Geology. 2023;39(4):507–21. [in Chinese]

11. Huang S, Jia W.W, Yan J.J. Relationship between sandbody characteristics and uranium mineralization in the Lower Member of the Toutunhe Formation, Louzhuangzi area, southern Junggar Basin. Uranium Geology. 2023;39(4):522–32. [in Chinese]

12. Jia W.W, Wang G.R, Tang X.F, Huang S, Yan J.J. Relationship of alteration zone types to uranium mineralization in the Toutunhe Formation, Louzhuangzi area, southern Junggar Basin. World Nuclear Geoscience. 2023;40(2):152 – 61. [in Chinese]

13. He Z.B, Hu B.Q, Qiu L.F, Wang Y, Chen H, Jia W.W, et al. The role of hydrocarbons in the formation of uranium mineralization, Louzhuangzi district, southern Junggar Basin (China). Minerals. 2024;14(7):709. doi:10.3390/min14070709.

14. Yang Z.H, Dong Y, Liu C, Liu M, Zhu Y.S, Zhou Y. Occurrence state of uranium in the Middle Jurassic Toutunhe Formation, Liuhuang'gou region, southern margin of Junggar Basin. Uranium Geology. 2022;38(6):1032–42. [in Chinese]

15. Hu X.W, Yang X.Y, Ren Y.S, Wu Z.J, Du G.F, Huang T.T. Sedimentary environment and tectonic evolution of Junggar Basin: Constraints on sandstone-type uranium mineralization. Geotectonica et Metallogenia. 2020;(4):725 – 41. doi:10.16539/j.ddgzyckx.2020.04.013. [in Chinese]

16. Wu Z.H, Jiang W, Zhou J.R, Li J.S. Thermal chronological dating of pluton thermal history and tectono-geomorphic evolution of the central Tibetan Plateau. Acta Geologica Sinica. 2001;(4):468–76. [in Chinese]

17. Fang S.H, Guo Z.J, Wu C.D, Zhang Z.C, Wang M.N, Yuan Q.D. Jurassic clastic composition in the southern Junggar Basin, NW China: Implications for basin–range pattern and tectonics. Acta Geologica Sinica. 2006;(2):196–209. [in Chinese]

18. Fang S.H, Song Y, Jia C.Z, Guo Z.J, Zhang Z.C, Liu L.J. Timing of Cenozoic intense deformation at the north margin of the Tianshan and implications for petroleum accumulation. Earth Science Frontiers. 2007;14(2):205–14. [in Chinese]

19. Han B.F, Guo Z.J, Zhang Z.C, Zhang L.Z, Chen J.F, Song B. Age, geochemistry, and tectonic implications of a late Paleozoic stitching pluton in the North Tianshan suture zone, western China. Geological Society of America Bulletin. 2010;122(3–4):627–40. doi:10.1130/B26491.1.

20. Li W.H, Xu G.Z, Quan J.P. Late modification of the intermountain basins of the Tien Shan orogenic belt and sandstone-type uranium mineralization. Mineral Deposits. 2010;29(S1):141–42. doi:10.16111/j.0258-7106.2010.s1.081. [in Chinese]

21. Xiao X.C, Tang Y.Q, Wang J, Gao J, Zhao M. Blueschist belts and their tectonic implications in the South Tianshan Mountains, NW China. Acta Geoscientia Sinica. 1994;(Z2). [in Chinese]

22. Guan X.T, Wu C.D, Wu J, Zhou J.Q, Jiao Y, Zhou R, et al. Sedimentary sequence and depositional evolution of Upper Jurassic–Lower Cretaceous strata, southern margin of Junggar Basin. Xinjiang Petroleum Geology. 2020;41(1):67–79. [in Chinese]

23. Folk R.L. Petrology of Sedimentary Rocks. Austin (TX): Hemphill Publishing;

1968. 170 p.

24. Yin Z.Q, Qin X.G, Wu J.S, Ning B. Multimodal grain-size distribution characteristics of loess, desert, lake and river sediments in parts of northern China. Acta Sedimentologica Sinica. 2009;27(2):343–51. [in Chinese]

25. Wang H.W. Sedimentary microfacies and evolutionary simulation of a meandering river in the NmIII oil group, Yangerzhuang Oilfield [dissertation]. Qingdao: China University of Petroleum (East China); 2024. [in Chinese]

26. Tang Y.N, Hu C.L, Dan S.H, Han C.C, Liu Z.M. Depositional model for the Early Triassic braided river delta and controls on oil reservoirs in the eastern Junggar Basin, NW China. Minerals. 2022;12(11):1409. doi:10.3390/min12111409.

27. Liu S, Li J.B, Jin F.B, Liu H.F, Wang M, Cheng Y.L, et al. Genesis of efficient reservoirs in mixed sedimentary settings: Benxi Formation, Gaoqiao area, Ordos Basin. Turkish Journal of Earth Sciences. 2024;33(2):118–45. doi:10.55730/1300-0985.1903.

28. Wang J, Gao X.J, Zhou X.M. Reservoir architecture characterization and remaining oil distribution of meandering rivers. Journal of China University of Petroleum. 2019;43(3):13–24. [in Chinese]

29. Zhu Z.J, Huang B.H, Guo F.S, Zheng H.F, Jiang Y.B. Cretaceous braided-river facies and Danxia landform development in Longhushan World Geopark, Jiangxi. Acta Geoscientia Sinica. 2012;33(3):379–87. [in Chinese]

30. Liu Y.M, Chen R.X, Zhao D.D, Zhang Z.Y, Wang J, Hou J.G. Sedimentary characteristics of gravelly braided rivers, Lower Shihezi Formation, Jin 72 well area, Dongsheng gas field. Petroleum Science Bulletin. 2022;7(4):457–74. [in Chinese]

31. Jolivet M, Bourquin S, Heilbronn G, Robin C, Barrier L, Dabard M.P, et al. The Upper Jurassic–Lower Cretaceous alluvial-fan deposits of the Kalaza Formation (Central Asia): Tectonic pulse or increased aridity? Geological Society, London,

Special Publications. 2017;427(1):491–521. doi:10.1144/SP427.6.

32. Vinokurov S.F, Nesterova M.V. New mechanism for uranium–titanium association formation in paleovalley ores. Doklady Earth Sciences. 2010;431(1):380–85. doi:10.1134/S1028334X10030244.

33. Yin S, Yan Z.B, Fu J.L, Zhang W, Liu H, Xia F, et al. Polymorphic transformations of titanium oxides contribute to economic uranium mineralization in sandstone. Geology. 2024; doi:10.1130/G51982.1.

34. Song H, Ni S.J, Hou M.C, Zhang C.J, Shi Z.Q, Wang G, et al. Clay minerals in sandstone-type uranium deposits of the Yili Basin (NW China) and their relationship to uranium mineralization. Acta Geologica Sinica. 2016;90(12):3352–66. [in Chinese]

35. Bonnetti C, Cuney M, Michels R, Truche L, Malartre F, Liu X, et al. Multiple roles of sulfate-reducing bacteria and Fe–Ti oxides in the Bayinwula roll-front uranium deposit, Erlian Basin, NE China. Economic Geology. 2015;110(4):1059–81. doi:10.2113/econgeo.110.4.1059.

36. Jia J.L, Miao C.F, Xie W.Q, Wu Y.J. Multi-scale sedimentary facies controls on fluvial sandstone-type uranium accumulation in the Songliao Basin, NE China. Ore Geology Reviews. 2023;161:105673. doi:10.1016/j.oregeorev.2023.105673.

37. Reynolds R.L, Goldhaber M.B. Origin of a South Texas roll-type uranium deposit: I. Alteration of iron–titanium oxide minerals. Economic Geology. 1978;73(8):1677–89. doi:10.2113/gsecongeo.73.8.1677.

38. Castillo-Oliver M, Melgarejo J.C, Torró L, Villanova-de-Benavent C, Campeny M, Díaz-Acha Y, et al. Sandstone-hosted uranium deposits as a possible source for critical elements: The Eureka Mine case (Catalonia). Minerals. 2020;10(1):34. doi:10.3390/min10010034.

39. Cumberland S.A, Evans K, Douglas G, de Jonge M, Fisher L, Howard D, et al.

~~Characterisation of uranium - pyrite associations within organic-rich Eocene sediments using EM, XFM - µXANES and µXRD. Ore Geology Reviews. 2021;133:104051. doi:10.1016/j.oregeorev.2021.104051.~~

~~40. Spirakis C.S. The roles of organic matter in the formation of uranium deposits in sedimentary rocks. Ore Geology Reviews. 1996;11(1):53–69. doi:10.1016/0169-1368(96)00007-6.~~

~~41. Hall S.M, van Gosen B.S, Zielinski R.A. Sandstone-hosted uranium deposits of the Colorado Plateau, USA. Ore Geology Reviews. 2023;155:105353. doi:10.1016/j.oregeorev.2023.105353.~~

~~42. Qiu L.F, Li Z.Y, He F, Wu Z.Q, Liu K.P, Zhang L, et al. Contribution of deep organic fluids to uranium mineralization in Cretaceous sandstones, southwestern Ordos Basin, China. Hydrogeology Journal. 2025;33(4):925 – 44. doi:10.1007/s10040-025-02912-4.~~

~~43. Liu Y, Zhao J.H, Jiao Y.Q, Liu Z.R, Zhou M.F. In situ S isotopic compositions of pyrite and the genesis of the Bayinqinggeli sandstone-hosted uranium deposit, Ordos Basin, northern China. Ore Geology Reviews. 2023;161:105608. doi:10.1016/j.oregeorev.2023.105608.~~

~~44. Tang C, Xu Z.L, Duan M, Meng L.S, Liu H.J, Wei J.L, et al. Genetic model of the Luhai sandstone-type uranium deposit, Erlian Basin, Inner Mongolia. Minerals. 2025;15(3):294. doi:10.3390/min15030294.~~

~~45. Liu B, Hao P, Zhang Z.L, Guo X.J, Zhang P.F, Shi Z.Q. Geology, alteration system and uranium mineralization of the Tamusu large uranium deposit, China. Ore Geology Reviews. 2023;158:105488. doi:10.1016/j.oregeorev.2023.105488.~~

Supporting information

S1 Fig. (A) **Sketch map showing tectonic location of Junggar Basin** (modified from [38~~21~~]); (B) **Geological sketch map of Louzhuangzi area** (modified from [15~~12~~]); (C)

Division and distribution of the main tectonic units at southern margin of Junggar Basin (modified from [~~9~~12]).

S2 Fig. **Stratigraphic column of the Louzhuangzi area** (modified after an internal report from Geological Party No. 216, CNNC, 2022)~~**Composite stratigraphic column of Louzhuangzi area** (Modified based on the internal report from the geological party No. 216, CNNC, 2022)~~.

S3 Fig. Petrographic photographs of Toutunhe Formation (J_2t) outcrops in Louzhuangzi area: (A-C) ~~Sandbody~~Sandstone outcrop of the Upper Member, Toutunhe Formation (J_2t^2), showing fine-medium sandstone with parallel beddings (A), mudstone with massive beddings (B), fine-medium sandstone with horizontal beddings (B), and fine sandstone with flaser beddings and planar cross beddings (C); (D-F) ~~Sandbody~~Sandstone outcrop of the Lower Member, Toutunhe Formation (J_2t^1), showing sandstone with large to giant planar cross beddings (D), conglomerate with massive beddings (E), and glutenite and coarse sandstone with trough cross beddings (F); (G) Interface between the Lower Member of the Toutunhe Formation (J_2t^1) and the Xishanyao Formation (J_2x); (H) ~~Sandbody~~Sandstone outcrop of the Xishanyao Formation (J_2x), showing siltstone with parallel beddings and coal lines.

S4 Fig Classification diagram of sandstones in Toutunhe Formation (J_2t), Louzhuangzi area (According to [41~~23~~]).

S5 Fig Petrographic characteristics of sandstone samples from Toutunhe Formation (J_2t) in Louzhuangzi area under microscope: (A) Sharp-edged quartz clasts cemented by basal carbonate cementation, ZK5-1, 22ZGE033, 148.50 m, (+); (B)

Formatted: Font: Not Bold

Clastics and clay matrix, ZK6-2, 22ZGE016, 477.30m, (+); (C) Pink crystalline quartz and feldspar matrix filled among debris particles, ZK6-2, 22ZGE016, 477.30m, (+); (D) Illitizated plagioclase with relatively dirty surface, ZK6-2, 22ZGE063, 478.60m, (+); (E) Lattice twin crystal microcline, ZK6-2, 22ZGE014, 483.47m, (+); (F) Bending and crushing of serimicized biotite, ZK12-2, 22ZGE001, 672.48m, (+); (G) Pyrite cementation, ZK4-2, 23ZGE026, 398.20m (reflected light); (H) Fine-grained pyrite and uranium minerals filled the cell cavities of carbonaceous debris, ZK4-2, 23ZGE026, 398.20 m, (BSE image); (I) Pyrite and uranium minerals filled among the clastic particles, ZK12-3, 23ZGE028, 798.50 m, (BSE image).

S6 Fig. Debris characteristics of sandstone samples from Toutunhe Formation (J_2t) in Louzhuangzi area under microscope: (A) Cryptocrystalline tuff debris, ZK6-2, 22ZGE016, 477.30 m, (+); (B) Platy-structure quartz schist debris, ZK6-2, 22ZGE015, 479.30m, (+); (C) Sericitization of sharp edged polycrystalline quartz, ZK6-2, 22ZGE016, 477.30m, (+); (D) Dacite porphyry debris with porphyritic texture, ZK6-2, 22ZGE026, 398.80m, (+); (E) Dissolution pit visible in cryptocrystalline flint debris, ZK6-2, 22ZGE026, 398.80m, (+); (F) Carbonate-cemented granite debris, ZK11-2, 22ZGE034, 24.84 m, (+).

S7 Fig. Photographs of representative core samples from Toutunhe Formation (J_2t) exposed by the ~~industrial~~economic uranium mineralization drill hole of ZK5-7 in Louzhuangzi area: (A) Meandering river facies ' thick mud with thin sand from the Upper Member of the Toutunhe Formation (J_2t^2); (B) Gravelly braided river facies thick sand body from the Lower Member of the Toutunhe Formation (J_2t^1); (C) Sandy braided

river facies flood mud and crevasse splay sand from the bottom of the Lower Member of the Toutunhe Formation (J_2t^1) .

S8 Fig. Interpretation of the core profile of drill hole ZK5-1 in Louzhuangzi area. (Abbreviation: Fm=Formation; DF= Depositional Facies; OD= Overbank Deposit; SCF= Sandy Channel Fill; LD= Lag Deposit; GB= Gravel Bar; FP= Floodplain; CS= Crevasse Splay; CB= Channel Bar; IDB= Interdistributary Bay; SDC= Subaqueous Distributary Channel).

S9 Fig. Sketch diagram showing the A-A' profile in Louzhuangzi area (In the Lower Member of the Toutunhe Formation (J_2t^1), the top roof of the sandstone has been leveled. The profile position is shown in Figure 1C).

S10 Fig. Occurrence characteristics of uranium in sandstones of Toutunhe Formation (J_2t) in Louzhuangzi area (BSE images), including (A) Gelatinous coffinite associated with pyrite, ZK11-3, 23ZGE022, 119.52m; (B) Massive coffinite associated with fine-grained pyrite, ZK12-3, 23ZGE028, 798.50m; (C) Massive pitchblende associated with pyrite, ZK12-3, 23ZGE028, 798.50 m; (D) Baited pitchblende coexisted with coffinite with ring-shaped distribution; ZK4-2, 23ZGE026, 398.20 m; (E) Nanoscale titanium-uranium aggregates associated with chlorite, ZK3-3, 23ZGE027, 753.10m; (F) Nanoscale uranium particles adsorbed in clay minerals, ZK12-3, 23ZGE028, 798.50m; (G) Nanoscale uranium minerals adsorbed at the edge of titanium oxides, ZK4-2, 23ZGEe026, 398.20m; (H) Nanoscale uranium minerals adsorbed in the dissolution pores of titanium oxides, ZK12-3, 23ZGE028, 798.50 m; (I) Uranium-rich monazite associated with baited pitchblende, ZK4-2, 23ZGE026,

398.20m; (J) Uranium-rich rutile, ZK4-2, 23ZGE026, 398.20 m; (K-L) Area distribution of U(K) and Ti (L) elements in uranium-rich rutile obtained by electron probe.

S11 Fig. Images showing the characteristics of titanium-containing uranium minerals under FIB and TEM (ZK1202, 22ZGE001, 672.48 m), including (A) Backscattered electron (BSE) image of titanium-containing uranium mineral under focused ion beam (FIB) scanning electron microscope; (B) Image of the transparent foil of titanium-containing uranium mineral under transmission electron microscope (The transparent foil was taken vertically downward at the yellow rectangular position in Figure A, with the thickness <100 nm); (C) HAADF-STEM image of titanium-containing uranium mineral; (D-I) STEM-EDS-derived area distribution of Ti-U(D), U(E), Ti(F), Ca(G) and Si(H) elements and energy spectra (I) of the black rectangular area in Figure C.

Formatted: Font: Bold

S12 Fig. Epigenetic alteration characteristics of ~~sandbodies~~sandstones in Toutunhe Formation (J_2t), Louzhuangzi area, including (A-D) Sand-yellow oxidized ~~sandbody~~sandstone outcrop (A); medium to large planar cross bedding developed in the ~~sandbody~~sandstone (B); incompletely oxidized carbonized plant detritus (C) and layered gypsum (E) occasionally observed; uranium anomaly commonly observed in gravel-bearing coarse sandstone (D) of the Upper Member of the Toutunhe Formation (J_2t^2) at Wanjiayao mineralization site; (F-G) Incompletely oxidized gray residues in the sand-yellow oxidized ~~sandbody~~sandstone (F); uranium anomalies observed in the oxidized ~~sandbody~~sandstone (G) of the Lower Member of the Toutunhe Formation (J_2t^1)

at Hasa tomb anomaly site; (H-I) Oxidized ~~sandbody~~sandstone (H) and multiple layers of gypsum (I) of the Upper Member of the Toutunhe Formation (J_2t^2) exposed by drill hole ZK11-3; (J) Laminated carbonized plant detritus and pyrite distribution observed in the grayish-white altered ~~sandbodies~~sandstones in the Lower Member of the Toutunhe Formation (J_2t^1) exposed by drill hole ZK 6-4; (K) Kaolin and pyrite nodules developed in the grayish-white altered ~~sandbodies~~sandstones in the Lower Member of the Toutunhe Formation (J_2t^1) exposed by drill hole ZK 12-3; (L) Laminated carbonized plant detritus and pyrite distribution exposed by drill hole ZK4-3 in depth of 758.24m.

S13 Fig. Profile of interlayer oxidation zones and uranium mineralization zones in ~~sandbodies~~sandstones of Toutunhe Formation (J_2t) in drill hole ZK 11-2 at Hasa tomb anomaly site (with sandstone top roof being leveled).

S14 Fig. Distribution of depositional facies and oxidation zones in the Lower Member of Toutunhe Formation (J_2t^1), Louzhuangzi area (modified from [13~~10~~]).

S15 Fig. Characteristics of clay minerals in ~~sandbodies~~sandstones in Lower Member of Toutunhe Formation (J_2t^1), Louzhuangzi area (BSE images), including (A) Intergranular layered kaolinite, ZK16-2, 23DJA-9, 705.70m; (B) Intergranular worm-like kaolinite, ZK16-2, 23 dJA-6, 654.60m; (C) Intergranular plate-like kaolinite, ZK12-3, 23 dJA-27, 641.70m; (D) Spherical ~~chlorite/~~smectite mixed layer mineral on the particle surface , ZK4-3, 23DJA-7, 710.40m; (E) Cellular ~~chlorite/~~smectite mixed layer mineral on the particle surface , ZK12-3, 23 DJA-15,871.50m; (F) The strawberry pyrite Encapsulated by flake-like ~~chlorite/~~smectite mixed layer mineral, ZK12-2,

23DJA-4, 680.70m; (G-I) Intergranular flake-like chlorite aggregate, ZK16-2, 23DJA-9, 705.70m.

S16 Fig. Association of uranium minerals and clay minerals in ~~sandbodies~~sandstones in Lower Member of Toutunhe Formation (J_2t^1), Louzhuangzi area (BSE images), including (A) Uranium mineral adsorbed in ~~the~~ chlorite~~/smectite mixed layer mineral~~, ZK3-3, 23ZGe027, 753.10m; (B) Uranium mineral associated with chloritized biotite, ZK4-2, 23ZGE026, 398.20m; (C) Uranium minerals associated with chlorite, ZK12-3, 23ZGE028, 798.50m.

S17 Fig. Alteration characteristics of titanium oxides in ~~sandbodies~~sandstones in Lower Member of Toutunhe Formation (J_2t^1), Louzhuangzi area. (A-C) Anatase and magnetite mineral assemblage enveloped by gelatinous pyrite (reflected light); (D) uranium-bearing oxides enveloped by gelatinous pyrite; (E-F) Interwoven association of anatase and pyrite (BSE image).

S18 Fig. Schematic diagram of the B-B ' drill hole profile in Louzhuangzi area. The profile position is shown in Figure 1C (modified from [15-16~~12-13~~]).

S19 Fig. Schematic diagram of the uranium mineralization model in the Louzhuangzi area, including (A) Types of mineral alteration in different fluid environments; (B) Interlayer oxidizing fluid mineralization; (C) Thermal fluid superposition transformation with oil and gas reduction.

S1 Table. Property of sandstone samples from Toutunhe Formation (J_2t) of Louzhuangzi area, southern margin of Junggar Basin.

S2 Table. Analysis results of chemical components in uranium-rich minerals in Louzhuangzi area by electron probe (Wt %).

Editor-in-Chief,

PLOS ONE,
Dear Editor,

Thank you for your constructive comments that were very helpful in improving the quality of this manuscript. We have carefully reviewed the comments and made corresponding revisions to the manuscript. We have submitted the second revised manuscript to PLOS ONE, entitled " **Control of deposition-epigenetic alteration of sandbody on uranium mineralization in Toutunhe Formation, Louzhuangzi area, Southern Margin of Junggar Basin, China** ".

We have comprehensively revised the paper based on the reviewers' comments. The main revisions are marked in red in the manuscript. The most important revisions are as follows:

Reviewer #2:
The authors have addressed the comments appropriately, and the manuscript is now suitable for publication.
Response: Thank you for your constructive suggestions. We sincerely thank the reviewer for the careful evaluation and positive assessment of our manuscript. The comments and suggestions provided during the first round of review were essential in substantially improving the quality of this paper, and we are very grateful for your continued support.

Reviewer #3:
Review of the manuscript titled “Control of deposition-epigenetic alteration of sandbody on uranium mineralization in Toutunhe Formation, Louzhuangzi area, Southern Margin of Junggar Basin, China”, submitted to the journal “Plos One”.
Response: We sincerely thank you for taking the time to review our manuscript and for providing constructive comments. Your suggestions have been crucial in improving the quality of this paper, and we have revised the manuscript accordingly, as detailed below:
Specific modifications:
1. - Line 15: Remove “shows” and use has instead of have: “In recent years, some uranium mineralization with industrial values has been discovered in ….”
Response: Thank you for your constructive suggestions.
Original: In recent years, some uranium mineralization shows with industrial values have been discovered in the Louzhuangzi area, southern margin of the Junggar Basin, and a medium-sized uranium mineral area has been confirmed.
Revised: In recent years, some uranium mineralization with industrial values has been discovered in the Louzhuangzi area on the southern margin of the Junggar Basin, and a medium-sized uranium mineralized area has been confirmed.

2. - Line 21: use "in the Toutunhe Formation (J_2t) of the Louzhuangzi area …", and you can remove "of the Louzhuangzi area" from line 27.

Response: Thank you for your constructive suggestions. We have revised the text accordingly by specifying "in the Toutunhe Formation (J_2t) of the Louzhuangzi area" in the first occurrence and removing the redundant phrase "of the Louzhuangzi area" from the later sentence. These revisions improve clarity and avoid repetition.

Original: In this study, the depositional characteristics of the sandbodies in the Toutunhe Formation (J2t) has been revealed through field geological investigation, collation of drill core data and petrographic analysis; the occurrence state of uranium and alteration characteristics of minerals coexisting with uranium has been explored by using scanning electron microscope (SEM), electron probe (EMPA), and focused ion beam scanning electron microscopy (FIB)- transmission electron microscopy (TEM). Comprehensive studies show that the uranium mineralization in the Toutunhe Formation (J2t) of the Louzhuangzi area is controlled by the coupling of multiple factors such as the gravel braided river channel lag deposits, the multi-stage alteration of epigenetic - thermal fluids, and the reduction of oil and gas.

Revised: In this study, the depositional characteristics of the sandbodies in the Toutunhe Formation (J_2t) of the Louzhuangzi area are investigated through field geological surveys, drill-core data compilation, and petrographic analysis. The modes of uranium occurrence and the alteration characteristics of uranium-associated minerals are examined using scanning electron microscopy (SEM), electron probe microanalysis (EPMA), and focused ion beam scanning electron microscopy – transmission electron microscopy (FIB – TEM). The results indicate that uranium mineralization in the Toutunhe Formation (J_2t) is controlled by the coupling of multiple factors, including gravelly braided-river channel lag deposits, multi-stage epigenetic–hydrothermal fluid alteration, and hydrocarbon-related reduction.

3. - Line 36: Turpan-Hami, Songliao Basins, and the northern⋯

Response: Thank you for your constructive suggestion. We have revised the sentence to improve clarity and consistency in the listing of basins by standardizing basin names and refining the wording. The revised text now clearly indicates that sandstone-type uranium deposits in northern China are mainly distributed in Mesozoic-Cenozoic fore-mountain basins, including the Ili, Turpan-Hami, Songliao, and Ordos basins.

Original: The sandstone-type uranium deposits in northern China are mainly found in the Mesozoic and Cenozoic fore-mountain basins, such as the southern margins of the Ili, Turpan-Hami, and Songliao Basins, and the northern part of the Ordos Basin.

Revised: Sandstone-type uranium deposits in northern China are mainly distributed in Mesozoic-Cenozoic foreland basins, such as Ili, Turpan-Hami, and Songliao basins, as well as the Ordos Basin.

4. - Line 41: mineralizations

Response: Thank you for your constructive suggestion. We have revised the sentence by changing "mineralization" to "mineralizations" to reflect the occurrence of

multiple uranium mineralized bodies, and slightly refined the wording to improve clarity and readability.

Original: Some promising uranium mineralization has been discovered in the Jurassic, Cretaceous and Paleogene sandstones in the Dingshan area of the northern basin, the Kamusite area of the eastern basin and the southern margin of the basin, which has attracted high attention from the industry.

Revised: Some promising uranium mineralizations have been discovered in Jurassic, Cretaceous, and Paleogene sandstones in the Dingshan area of the northern basin, the Kamusite area of the eastern basin, and along the southern margin of the basin, attracting considerable attention from the uranium exploration industry.

5. Lines 49-53: The sentence is very long.

Response: Thank you for your constructive suggestion. We have revised this part by splitting the long sentence into two shorter sentences, which improves clarity and readability while preserving the original meaning.

Original: The uranium-enriched geological bodies of the Northern Tianshan Mountains and the conglomerate layers in the bottom of the Lower Cretaceous Qingshuihe Formation provide uranium source for the uranium mineralization, and the relatively stable structure areas are the favorable locations for the mineralization of uranium.

Revised: The uranium-enriched geological bodies of the Northern Tianshan Mountains, together with conglomerate layers at the base of the Lower Cretaceous Qingshuihe Formation, provide important uranium sources for mineralization. In addition, relatively stable structural areas constitute favorable sites for uranium mineralization.

6. - Line 54: …during the period from the Late Cretaceous to the Paleogene. The Middle and…

Response: Thank you for your constructive suggestion. We have revised the text by restructuring the sentence and separating it into two clearer statements. This revision improves readability and more clearly expresses the temporal relationship between tectonic stability and uranium mineralization.

Original: The southern margin of the Junggar Basin was in a tectonically stable state during the Late Cretaceous to Paleogene period; the Middle and Lower Jurassic series received long-term modification by the uranium- and oxygen- containing water, resulting in the formation of a large amount of uranium mineralization.

Revised: The southern margin of the Junggar Basin remained tectonically stable during the period from the Late Cretaceous to the Paleogene. The Middle and Lower Jurassic strata experienced long-term interaction with uranium- and oxygen-bearing fluids, leading to the formation of significant uranium mineralization.

7. - Line 78: Use the full name for Si, P and Y.

Response: Thank you for your constructive suggestion. We have revised the sentence by replacing Si, P, and Y with their full oxide forms (SiO_2, P_2O_5, and Y_2O_3), which improves clarity and consistency with standard geochemical reporting.

Original: The components of uranium in this ore site are dominated by pitchblende and the unknown uranium minerals rich in elements such as Si, P and Y, with less brannerite and adsorbed uranium.

Revised: Uranium at this ore site is mainly hosted by pitchblende and unidentified uranium minerals enriched in SiO_2, P_2O_5, and Y_2O_3, with subordinate brannerite and adsorbed uranium.

8. - Line 79-82: The sentence is very long. - Line 81: determines that the mineralization…

Response: Thank you for your constructive suggestion. We have revised this part by restructuring the long sentence into two clearer statements and explicitly using "determines that" to improve grammatical correctness and readability. The revised text more clearly explains the genetic control of epigenetic oxidation fluids on uranium mineralization and its implications for in-situ leaching.

Original: The uranium mineralization uniquely derived from epigenetic oxidation fluid alteration and the in-situ leaching mining method of the sandstone-type uranium deposit determine the mineralization and production of uranium are closely related to the deposition-epigenetic alteration of the uranium-bearing sandbodies.

Revised: Uranium mineralization in sandstone-type uranium deposits is primarily derived from epigenetic oxidizing fluid alteration. This genetic characteristic determines that both uranium mineralization and uranium production by in-situ leaching are closely related to the depositional features and subsequent epigenetic alteration of uranium-bearing sandbodies.

9. - Lines 86-94: this is a paragraph that should consist of at least 3 sentences, not one sentence. Avoid long sentences. Same in lines 110-116, divide the paragraph into sentences.

Response: Thank you for your constructive suggestion. We have revised both paragraphs by dividing the original long sentences into multiple shorter sentences and reorganizing the text to improve clarity and readability. The revised paragraphs now consist of several concise sentences that better convey the scientific content and logical relationships.

Original: 86-94: In order to explore the controlling effect of sandbody deposition-epigenetic alteration on uranium mineralization in Toutunhe Formation (J2t) in the southern margin of Junggar Basin, a study on the deposition-epigenetic alteration characteristics of the uranium-bearing sandbodies has been carried out guided by the uranium mineralization theory related to epigenetic oxidation fluid alteration, sedimentology and alteration mineralogy, and the depositional environment of the uranium-bearing sandbodies and the properties of the ore-forming fluids have been determined, providing a reference for determining the future uranium exploration direction in the basin. 110-116: Since the stable block in the Tarim region was located

at the center of the compression, the lithospheric mantle of the stable block gradually thickened under compression, whereas the rock mass of the Tianshan Orogenic Belt, as the buffer and release area of the compressive stress, gradually rose and uplifted, and finally formed the current tectonic and geomorphic pattern of the Junggar Basin after long period of geological evolution, which is separated from the Tuha and Ili Basins by mountains.

Revised: 86-94: To investigate the controlling effects of sandbody deposition and epigenetic alteration on uranium mineralization in the Toutunhe Formation (J_2t) at the southern margin of the Junggar Basin, we conducted a systematic study of the depositional and alteration characteristics of uranium-bearing sandbodies. This work was guided by established theories of uranium mineralization related to epigenetic oxidizing fluid alteration, sedimentology, and alteration mineralogy. The depositional environments of the uranium-bearing sandbodies and the physicochemical properties of the ore-forming fluids were thereby constrained, providing a geological basis for future uranium exploration in the basin. 110-116: During regional compression, the Tarim stable block was located near the center of the compressive regime, resulting in progressive thickening of its lithospheric mantle. In contrast, the Tianshan Orogenic Belt acted as a buffer and stress-release zone, where crustal shortening and uplift gradually intensified. Through prolonged geological evolution, these processes ultimately shaped the present tectonic and geomorphic framework of the Junggar Basin, which is now separated from the Tuha and Ili basins by intervening mountain ranges.

10. - Line 102: …mineralization (Fig 19). Is it Figure 19 or Reference 19?

Response: Thank you for your constructive suggestion. The citation referred to Figure 19. However, considering that figure citations in the Introduction may be potentially misleading to readers, we have removed the figure reference from this sentence in the revised manuscript.

Original: We hypothesize that the formation and preservation of uranium mineralization are controlled by the combined effects of (1) braided channel sandbody architecture favorable for fluid migration, (2) multiple stages of epigenetic fluid alteration involving oxidizing, thermal, and reducing fluids, and (3) localized redox interfaces induced by hydrocarbons and sulfide mineralization (Fig 19).

Revised: We hypothesize that the formation and preservation of uranium mineralization are controlled by the combined effects of (1) braided channel sandbody architecture favorable for fluid migration, (2) multiple stages of epigenetic fluid alteration involving oxidizing, thermal, and reducing fluids, and (3) localized redox interfaces induced by hydrocarbons and sulfide mineralization.

11. - Line 124: the orientation “N-WW” seems incorrect.

Response: Thank you for your constructive suggestion. After rechecking the regional structural data, we confirm that both the Yilianhabirga Mountains and the Bogda Mountain tectonic belt trend NW－SE. The previously used orientations (N-WW and

N-E) were incorrect descriptions in the original manuscript. These errors have now been corrected to NW－SE in the revised version to ensure structural accuracy.

Original: In recent years, the Louzhuangzi uranium mineral area has been discovered in the intersection and subduction site of the N-WW oriented Yilianhabirga Mountains and the western sloping end of the N-E oriented Bogda Mountain tectonic belt (Haojiagou Uplift) (Fig 1B) in the fore-mountain fold-and-thrust belt of the Northern Tianshan Mountains.

Revised: In recent years, the Louzhuangzi uranium mineral area has been discovered at the intersection between the NW－SE－trending Yilianhabirga Mountains and the western plunging end of the Bogda Mountain tectonic belt (Haojiagou Uplift) (Fig. 1B), within the foreland fold-and-thrust belt of the Northern Tianshan Mountains.

12. - The quality of most of your Figures is bad. The content, essentially the texts, is unreadable (e.g., Figures 1, 2, 8, 9, 13, 14? 18). Please provide a high-quality version of your Figures.

Response: Thank you for your constructive suggestion. The reduced image quality observed in the current version is primarily due to compression applied during the manuscript submission and system-generated preview process. In the next (third-round) submission, we will replace all affected figures with uncompressed, high-resolution versions to ensure that all text, symbols, and details are clearly legible. If required by the editorial office, we are also happy to provide the original CorelDRAW source files or other high-resolution figure formats upon request.

13. - Line 150: use “stratigraphic column of Louzhuangzi area…” instead of “Composite stratigraphic column of Louzhuangzi area…”

Response: Thank you for your constructive suggestion. We have revised the figure caption by replacing “Composite stratigraphic column” with “Stratigraphic column of the Louzhuangzi area” to more accurately describe the figure.

Original: Fig. 2 Composite stratigraphic column of Louzhuangzi area (Modified based on the internal report from the geological party No. 216, CNNC, 2022).

Revised: Fig. 2 Stratigraphic column of the Louzhuangzi area (modified after an internal report from Geological Party No. 216, CNNC, 2022).

14. - Methods and Materials: label the subheads “Fieldwork and sampling, Petrographic and mineralogical analysis…” as 3.1; 3.2; …

Response: Thank you for your constructive suggestion. We have revised the Methods and Materials section by adding numbered subheadings, including 3.1 Fieldwork and sampling and 3.2 Petrographic and mineralogical analysis, to improve the structure and clarity of this section.

15. - Adding some field or lab pictures may be useful.

Response: Thank you for your constructive suggestion. We have added photographs documenting the field investigations conducted by the lead author, as well as images illustrating representative laboratory analytical procedures, as Supplementary Material

at the end of the revised manuscript. If the editorial office considers these materials unnecessary, they can be readily removed from the final version.

16. - Lines 191, 193: I ask if the Figures cited are Figures 7A, 7B?
Response: Thank you for your constructive suggestion. We confirm that the figures cited in Lines 191 and 193 indeed refer to Figures 7A and 7B. The citations have been checked and corrected where necessary in the revised manuscript to ensure accuracy and consistency.

17. - Lines 201: I ask if the Figure cited is Figure 12?
Response: Thank you for your constructive suggestion. We confirm that the figure cited in Line 201 indeed refers to Figure 12. In the revised manuscript, we have carefully checked this paragraph and corrected the inaccurate figure citations. The text has been updated to explicitly reference Fig 12B for planar cross-bedding and Figs. 12J–L for the occurrence of carbonaceous debris, pyrite, and clayization, ensuring consistency between the text and the figures.
Original: The depositional structures such as flaser bedding (Fig 3C), parallel bedding (Fig 3A), horizontal bedding (Fig 3B), and small to medium (with the single-layer system < 10 cm) planar cross bedding (Figs 3C and 12B) are commonly developed in the sandstone. The gravel-bearing sandstone, glutenite and conglomerate revealed by drilling are grayish white (Fig 7B), containing a considerable amount of carbonaceous debris and pyrite, with clayization developed (Figs 12J, K, and L).
Revised: The depositional structures such as flaser bedding (Fig 3C), parallel bedding (Fig 3A), horizontal bedding (Fig 3B), and small to medium (with the single-layer system < 10 cm) planar cross bedding (Figs 3C and 12B) are commonly developed in the sandstone. The gravel-bearing sandstone, glutenite and conglomerate revealed by drilling are grayish white (Fig 7B), containing a considerable amount of carbonaceous debris and pyrite（Figs 12 J-L）, with clayization developed (Fig 12 K).

18. - Line 239: use micrometer symbol “µm” not “um” for 2600um, 500 and 1500um,…
Response: Thank you for your constructive suggestion. In the revised manuscript and updated figures, all instances of “um” have been corrected to the proper micrometer symbol “µm” to ensure consistency with standard scientific notation.
Original: The clastic particles are relatively dispersed and generally do not contact with each other (Figs 5A-F), with the particle diameters being up to 2600um, mostly between 500 and 1500um, averaging about 800um.
Revised: The clastic particles are relatively dispersed and generally do not contact with each other (Figs 5A-F), with the particle diameters being up to 2600 µm, mostly between 500 and 1500 µm, averaging about 800 µm.

19. - Lines 340-344: The sentence “The ore-bearing main… bar developed” is very long. Same for the next one. Same for the sentence in lines 609-614.
Response: Thank you for your constructive suggestion.

Original: 340-344: The ore-bearing main sandbody in the Lower Member of the Toutunhe Formation (J_2t^1) is composed of the thick, grayish-white coarse sandstone, glutenite, and conglomerate interspersed by the thin layers of grayish-green mudstone on the profile (Fig 7B), with the depositional microfacies such as sandy channel fill, lag deposit and gravel bar developed (Fig 8) .609-614: Titanium oxide particles can be commonly observed in the slices of the gray and grayish-white sandbodies of the Lower Member of the Toutunhe Formation (J_2t^1) under the microscope, showing obvious characteristics of epigenetic alteration, and the morphology of the particles are mostly angular to sub-angular (Figs 17A, B), with a few being circular to sub-circular (Figs 17C, D) due to epigenetic alteration, indicating that these titanium oxides are altered products of the proximal deposits..

Revised: 340-344: The ore-bearing main sandbody in the Lower Member of the Toutunhe Formation (J_2t_1) consists mainly of thick grayish-white coarse sandstone, glutenite, and conglomerate, with thin interlayers of grayish-green mudstone (Fig 7B). Depositional microfacies including sandy channel fills, lag deposits, and gravel bars are well developed within this sandbody (Fig 8). 609-614: Titanium oxide particles are commonly observed in thin sections of the gray and grayish-white sandbodies of the Lower Member of the Toutunhe Formation (J_2t_1). These particles display clear features of epigenetic alteration and are predominantly angular to sub-angular in morphology (Figs 17A, B). A smaller proportion exhibits circular to sub-circular shapes (Figs 17C, D), suggesting modification by epigenetic processes and indicating that these titanium oxides represent altered products of proximal deposits.

20. - Line 376: “document” ? You want to say “shows” ?

Response: Thank you for your constructive suggestion. We have revised the sentence by replacing “document” with “shows”, which more appropriately conveys the intended meaning. The sentence has also been slightly refined for clarity and consistency.

Original: The classification studies of the depositional facies of the outcrops and borehole strata, together with the previous study results, document that the Lower Member of the Toutunhe Formation (J2t1) contains the depositional sandbodies of braided river delta plain, sandy braided river and gravelly braided river facies deposits (Figs. 7, 8 and 9).

Revised: The classification of depositional facies based on outcrops and borehole strata, together with previous studies, shows that the Lower Member of the Toutunhe Formation (J_2t_1) contains sandbodies deposited in braided-river delta plain, sandy braided-river, and gravelly braided-river facies (Figs 7-9).

21. - It is recommended to discuss the main strengths and limitations of this work.

-Overall, the manuscript must be revised before sending it for the next review.

Response: Thank you for your constructive suggestion. In response to your comments, we have outlined the main strengths and limitations of this study in the discussion section of the revised manuscript. Specifically, we highlighted the advantages of integrating mineralogical methods and nanoscale characterization, while also pointing

out current limitations, such as the limited analytical testing methods, the lack of XRD, Fluid inclusions, Bulk geochemistry, and Isotopic data support, and the need for more comparative datasets from other sandstone-type uranium deposit systems. In addition, the manuscript has undergone a comprehensive revision following your comments and those of the reviewers, including substantial restructuring, clarification of interpretations, and improvements in language, figures, and organization. We believe that these revisions have significantly improved the overall quality and clarity of the manuscript and have prepared it appropriately for the next round of review.

Reviewer #4:

1. The abstract lacks some key aspects, including

Quantify: Add numbers to findings (e.g., " 〖"CO" 〗_2purity >95%").

Clarify: Define the specific "controversy" being addressed.

Response: Thank you for your constructive suggestion. We have substantially revised the abstract of the original manuscript. The revised abstract strengthens the expression of research findings, and the controversies existing in current research have been adjusted to the introduction and discussed in detail.

2. Keywords: The authors may consider changing some of the keywords using one of the suggestions below:

- Sandstone-type uranium deposits (Standardized classification)
- Braided river delta (Specific depositional micro-facies from your abstract)
- Hydrocarbon-mediated reduction (The specific "how" of your mineralization)
- FIB-TEM/SEM analysis (Specific high-end methodology)
- Toutunhe Formation (Necessary stratigraphic marker)
- Redox transition zone (The geochemical environment)
- Northwest China (Broader geographic context to reach a wider audience)

Response: Thank you for your constructive suggestion. We have revised the Keywords section in accordance with your recommendations to better reflect the standardized deposit classification, key depositional micro-facies, principal mineralization mechanisms, applied analytical methods, stratigraphic context, and broader geographic setting. The revised keywords are provided below.

Original: Sedimentary environment; Epigenetic alteration; Uranium mineralization; Toutunhe Formation; Southern margin of Junggar Basin

Revised: Sandstone-type uranium deposits, Braided river delta, Hydrocarbon-mediated reduction, FIB-TEM/SEM analysis, Toutunhe Formation, Redox transition zone, Northwest China.

3. Are all the pictures used here original? If you take a random picture, you must have permission before using it.

Please state what role the funders took in the study. If the funders had no role, please state: "The funders had no role in study design, data collection and analysis, decision to publish, or preparation of the manuscript."

If this statement is not correct, you must amend it as needed. And include it in the cover letter, that will be enough.

Response: Thank you for your constructive suggestion. All figures in the manuscript are original unless specifically indicated otherwise. The roles of the funders in this study have been clearly stated in the "Author Contributions" section and further clarified in the cover letter with the following statement:"The funders made the following contributions during the research process: Conceptualization, Formal analysis, Investigation, Methodology, Project administration, and Resources."

4. Introduction: The text needs to be solidified by adding more references; each statement requires supporting references.

Response: Thank you for your constructive suggestion. In the revised version, we have improved the introduction, added key issues and controversies in current research, and provided relevant references.

5. Methodology: If it's possible, the author can add a graphical abstract.

Response: Thank you for your constructive suggestion. Figure 19 of the paper presents the ore-controlling sand body and mineral alteration characteristics as well as the multi-stage fluid interaction process. We consider using this figure as a graphic summary of this paper.

Reviewer #5:

1. Severe Introduction Deficiencies

The introduction (lines 32-105) lacks:

• Clear knowledge gaps: What specifically is unknown about this deposit type?
• Theoretical framework: No explicit statement of competing hypotheses.
• Literature synthesis: References are listed without critical evaluation of conflicting interpretations.

Response: Thank you for your constructive suggestion. In the revised version of the manuscript, we made significant changes to the introduction. Specifically, we supplemented the introduction with explanations of the problems that had existed in previous studies of this deposit, and clearly articulated the competing hypotheses.

2. Methods Section (lines 152-181)

Critical missing elements even after revision:

• Sample selection criteria and representativenes.
• Sampling Strategy Not Justified.
• Table 1 lists samples from various depths (24.84m to 871.50m) and drill holes.
• No explanation of selection criteria.
• No discussion of spatial representativeness.
• Samples concentrated in specific holes (e.g., 6 samples from ZK6-2, only 1 from ZK3-3).
• Potential sampling bias need to be addressed.
• Quality control procedures.

• Detection limits for EMPA analyses.
• Reproducibility assessment.

Response: Thank you for your constructive suggestion. In the revised version, we describe the sampling strategy and its sample selection criteria and representativeness. We address the criteria and representativeness of sample collection, the sampling strategy, and existing sampling bias issues. We also state the detection limit for EMPA analysis. All sample slides were properly preserved to ensure the reproducibility and verifiability of the analytical results.

EMPA Data Quality Issues (Table 2):
• Totals range from 84.01% to 97.14% - some analyses are poor quality.
• No standard reference materials mentioned.
• Single-point analyses without replication.
• No discussion of analytical uncertainty.
• Sub-optimal totals (e.g., 84.01% for brannerite) suggest volatile loss or inappropriate analytical conditions.

Response: Thank you for your constructive suggestion. In this revised version, we have added standard reference materials and conducted multi-point analyses of the same mineral aggregates, rather than single-point analyses. Furthermore, we have added a discussion of the uncertainties in the analytical results.

Missing Critical Techniques: The authors acknowledge (only after review) absence of:
• XRD - fundamental for phase identification.
• Fluid inclusions - critical for understanding ore-forming fluids.
• Bulk geochemistry - necessary for provenance and alteration assessment.
• Isotopic data - essential for fluid source and timing constraints.

Response: Thank you for your constructive suggestion. We fully understand your concerns regarding the key analytical techniques and agree with your views. We are very grateful to the reviewers for their suggestions on supplementary analytical techniques. However, our research is currently in its initial stages, and we will actively conduct relevant experimental analyses in subsequent studies to further supplement and improve the existing gaps in our research. We look forward to receiving your further guidance in future research.

The Response to Reviewers states: "Due to the limited quantity of samples available for destructive analysis, XRD analysis was not conducted". This is scientifically unacceptable because:
• XRD requires minimal sample (~100mg).
• The study collected 22 samples (Table 1).
• XRD is non-negotiable for validating mineral identifications.
• Claims about "chlorite/smectite mixed layer minerals" cannot be confirmed without XRD.

Response: We fully understand your concerns regarding our previous response. We acknowledge that our previous response was somewhat weak. The truth is that the

samples for experimental analysis were sent to a third-party rock thin-section preparation company. Previously, we only retrieved the sample thin sections for analysis; the remaining solid block samples were not retrieved in time, resulting in a limited number of samples available for destructive analysis. We have now contacted the third-party company and retrieved some of the block samples for XRD analysis in subsequent studies. Furthermore, based on the morphology and energy dispersive spectroscopy characteristics of the clay minerals observed so far, we have initially classified them as kaolinite, montmorillonite, and chlorite. In the revised manuscript, we have revised the uncertain "chlorite/montmorillonite mixed layer minerals" to montmorillonite.

3. Results-Discussion Conflation

Despite revision, interpretive statements remain embedded in Results:

• Lines 314-315: "indicating that the type of the sandstone is greywacke and the sandbodies were formed in a proximal deposit environment" - this is interpretation.

• Lines 345-361: Depositional environment interpretation mixed with descriptive sedimentology.

• Lines 520-528: Genetic interpretations presented as observations.

Response: Thank you for your constructive suggestion. In the revised manuscript, we removed the explanatory statement "indicating that the type of sandstone is greywacke and the sandbodies were formed in a proximal deposit environment." Furthermore, we made significant revisions to the results section, "4.2 Depositional environment and facies interpretation." Specifically, we moved this title and related explanatory statements to the discussion section as subsection 5.1, and renamed the results section as "4.2 Sandbody Depositional Characteristics," retaining the results describing the sandbody characteristics. The related statements in lines 520-528 were also revised accordingly.

Brannerite Identification - Overstated Certainty

The manuscript claims (lines 457-467) definitive brannerite identification, but:

• Evidence presented: Only EDS element mapping showing Ti and U co-distribution.

• Missing: Electron diffraction patterns confirming brannerite crystal structure.

• TEM images (Fig. 11B-C): Show "complexly interwoven acicular aggregates" but lack lattice fringe imaging.

• Chemical composition (Table 2): TiO_2/UO_2 ratios (average 33.20%/39.62%) deviate from stoichiometric UTi_2O_6 (expected ~29.7%/63.3%).

Critical issue: The text contradicts itself - lines 467 states this is "nanoscale brannerite aggregate, rather than nanoscale coffinite or pitchblende associated with titanium oxides," but the evidence for this distinction is not compelling without crystallographic data.

Response: Thank you for your constructive suggestion. In the revised manuscript, we have replaced the overly confident description of "brannerite" with the more cautious term "brannerite -like nanomineral aggregates." Our published paper (DOI: 10.1016/j.oregeorev.2025.106908) provides a detailed discussion of the electron

diffraction patterns and lattice-fringe imaging associated with the crystal structure of brannerite-like phases, as well as the occurrence of nanocrystalline brannerite aggregates (rather than nanoscale uranyl silicates or pitchblende related to Ti oxides). In addition, the discrepancy between the TiO_2/UO_2 ratio of the uranium nitride–like phase in Table 2 (mean = 33.20%/39.62%) and the stoichiometric TiO_2/UO_2 ratio expected for UTi_2O_6 (approximately 29.7%/63.3%) may be attributable to its low crystallinity and/or the presence of amorphous domains.

4. Interpretive Weaknesses

(1) "Thermal Fluids" - Unsupported Speculation

The manuscript repeatedly invokes "thermal fluids" (lines 29-30, 101, 731-744, 754) but:

Evidence provided:

- Clay mineral assemblages (chlorite, chlorite-smectite).
- Brannerite occurrence.
- Sulfurization features.

Problems:

- These features can form at diagenetic temperatures (<100° C).
- No temperature constraints whatsoever.
- No fluid inclusion data.
- No isotopic evidence for deep fluid sources.
- The Discussion (lines 731-744) is essentially an admission that thermal fluids are speculative.

The authors state (Response to Reviewers): "due to the lack of direct fluid inclusion or isotopic data, the properties and pathways of these thermal fluids cannot be definitively identified".

Then why make it a central interpretive element? This represents speculation presented as conclusion.

Response: Thank you for your constructive suggestion. Thank you for this detailed and important critique. We agree that our previous wording and emphasis could be read as treating “thermal fluids” as a firm conclusion, whereas our dataset does not provide direct temperature constraints (e.g., fluid-inclusion microthermometry) or definitive isotopic fingerprints for a deep fluid source. Therefore, we have revised the manuscript to indicate the limitations of the current study, emphasize the inferences and conceptual models regarding fluid action, and ensure that all interpretations match the existing level of evidence.

ii. Hydrocarbon Reduction - Circumstantial Evidence

The manuscript argues (lines 69-71, 641-643, 677-690) that H_2S and CH_4 from hydrocarbon leakage caused reduction, based on:

- Presence of pyrite and bitumen.
- Regional petroleum geology.
- Citation of He et al. (2024).

Missing:

- Direct evidence of hydrocarbon fluid inclusions.

• Organic geochemistry linking bitumen to deep sources.
• Sulfur isotope data to constrain H_2S sources (biological vs. thermochemical).
• Temporal constraints on hydrocarbon migration vs. uranium precipitation.
Reviewer 1 noted: "Additional data, such as fluid inclusions with more analysis, could strengthen the hydrocarbon reduction hypothesis".
Authors' response: Acknowledge limitation and cite He et al. (2024) for supporting evidence.
Problem: This effectively outsources key evidence to another study while maintaining the interpretation as a primary conclusion.
Response: Thank you for your constructive suggestion. Thank you for this careful and constructive critique. We agree that our previous wording could be read as treating hydrocarbon-derived reduction (H_2S/CH_4) as a primary conclusion, whereas the evidence presented in this manuscript is largely indirect (bitumen + pyrite occurrence and regional petroleum context). We have therefore revised the manuscript to ensure that the hydrocarbon-reduction interpretation is presented as a supported but non-exclusive hypothesis, explicitly limited by the absence of several key datasets.

iii. Depositional Facies Analysis - Oversimplified
The depositional environment interpretation (Section 4.2, lines 305-396) suffers from:
• Sedimentary structures described but not systematically analyzed: No paleocurrent data, no grain size distribution analysis, no facies associations diagram.
• Uncritical facies assignment: E.g., "flaser bedding... mainly formed by migration and deposition... in meandering river channel" (lines 317-320) - but flaser bedding forms in tidal settings and other environments.
• Missing quantitative data: No channel dimensions, no sand/mud ratios, no architectural element analysis.
• References misapplied: Folk (1968) cited for sedimentary environments (line 344) when it's a textural classification scheme.
Response: Thank you for your constructive suggestion. Thank you for this detailed and valuable critique. We agree that the original facies discussion in Section 4.2 contained (i) interpretive statements expressed too definitively, (ii) facies assignments that were not sufficiently constrained by systematic facies analysis, and (iii) an inappropriate citation (Folk, 1968) for depositional-environment interpretation. We have revised the manuscript accordingly to improve rigor and to clearly separate descriptive sedimentology (Results) from depositional-environment interpretation (Discussion).

5. DATA PRESENTATION ISSUES
(1) Table 2 - Serious Problems
Compositional Data:
• 35 analyses presented without context (which samples? which grains?).
• Mineral types assigned post-hoc based on composition.
• No clear criteria for distinguishing "dispersed adsorbed uranium" (5 analyses) from coffinite (16 analyses).

• Some "coffinite" analyses have very low SiO_2 (9.33%) approaching pitchblende composition.
Statistical treatment absent:
• No mean ± standard deviation.
• No compositional ranges by deposit location.
• No correlation analysis (e.g., UO_2 vs. TiO_2).

Response: Thank you for your constructive suggestion. Thank you for such detailed and important comments; your suggestions are highly valuable for improving the quality of our manuscript. We agree that the original Table 2 did not provide sufficient contextual information for the 35 analyses and that the statistical treatment of the dataset was incomplete. In the revised version, each analysis in Table 2 is now explicitly linked to its corresponding sample ID, and mean values are provided for each compositional group. Mineral-type assignments were made by integrating the petrographic/textural characteristics of the analyzed spots with their compositional signatures.

The term "dispersed adsorbed uranium" has now been revised to "Unidentified nano-uranium minerals," and these analyses are distinguished from coffinite based on their mineral assemblage context and oxide composition characteristics. We also note that some coffinite analyses display very low SiO_2 contents approaching those of pitchblende; however, their UO_2 contents are generally lower than the overall UO_2 range measured for pitchblende.

Because the number of ore samples analyzed from different sectors of the deposit is currently limited, we did not subdivide compositional ranges by deposit location in order to avoid overinterpretation. In addition, given the clear mineralogical differences among the identified phase groups and the large variability in UO_2 and TiO_2 contents, the present dataset is insufficient to support robust correlation analysis. In future work, we will expand both the number of analyzed ore samples and the total dataset, subdivide results by deposit sector, and conduct correlation analyses to evaluate compositional trends and interrelationships.

6. DISCUSSION SECTION - SUPERFICIAL SYNTHESIS
The Discussion (added/restructured after revision, lines 646-755) has improved structure but remains problematic:
Section 5.1 (Sedimentary control):
• States braided channel facies are primary hosts (correct).
• Comparison with Ordos, Songliao, Texas deposits mentioned but not developed.
• Missing: Why is Toutunhe Formation mineralization similar/different from these analogues?
Section 5.2 (Compositional maturity):
• Q/(F+R) ratios discussed (lines 657-675).
• Links low maturity to uranium mobility – reasonable.
• Missing: Comparison with unmineralized Toutunhe sandstones - are compositional differences significant?
Section 5.3 (Redox buffering):

• Argues pyrite and organics reduced U^{6+} to U^{4+}.
• Problem: This is standard ore-forming process - what's specific to Louzhuangzi?
• References [39-43] cited but not critically evaluated.
Section 5.4 (Alteration timing):
• Distinguishes early diagenetic from epigenetic alteration.
• Major gap: No geochronology, no cross-cutting relationships systematically documented.
• Relies on textural interpretation alone.
Section 5.5 (Future geochemical work):
• This is not discussion - it's an admission of missing data.
• Acknowledges need for major/trace elements, REE, Ti/Zr, Th/U ratios.
• Should have been done before publication.
Section 5.6 (Thermal fluids origin):
• Essentially states "we don't know" (lines 731-744).
• Speculation about basin-centered flow, magmatic underplating.
• No new insight beyond acknowledging uncertainty.
Section 5.7 (Mineralization model):
• Figure 19 presents conceptual model.
• Problem: Model incorporates unvalidated elements (thermal fluids, hydrocarbon timing).
• Three-stage process (oxidation → thermal overprint → hydrocarbon reduction) is plausible but not demonstrated.

Response: Thank you for your detailed and constructive comments. Based on the current revision status of the manuscript, we have substantially revised the Discussion section. In the revised version, we focus primarily on the preliminary findings obtained in this study and the limitations of the current dataset, and we have removed the geochemistry-oriented discussion that cannot be adequately supported by the available data. Accordingly, this manuscript presents our current interpretations while explicitly identifying the remaining uncertainties. Future work by the authors and other researchers in this area will require more comprehensive analytical approaches, including more detailed geochemical investigations, to further test and refine the proposed interpretations.

7. WRITING QUALITY AND PRESENTATION
Pervasive Issues:
i. Jargon and unclear terminology:
• "Deposition-epigenetic alteration" (title) - awkward phrasing.
• "Gravel braided river channel lag deposits" (line 28) - "gravel braided river" is non-standard (should be "gravelly braided river").
• "Uranium-bearing sandbody" - "sandbody" is informal; use "sandstone unit".

Response: Thank you for your constructive suggestion. We revised the awkwardly worded title to Sedimentary architecture and post-depositional alteration of the Toutunhe Formation (J_2t) in the Louzhuangzi area, Southern Junggar Basin:

Implications for uranium mineralization. We also corrected non-standard expressions such as "gravel braided river" and "Uranium-bearing sandbody" in the paper.

ii. Grammatical errors (examples):

• Line 88: "the properties of the ore-forming fluids have been determined" - overstated; properties remain uncertain.

• Line 314: "indicating that the type of the sandstone is greywacke" - awkward construction.

• Line 474: "The dispersed adsorbed uranium is actually the nano-scale uranium minerals" - "is actually" undermines precision.

iii. Redundancy:

• Extensive description of sedimentary structures (lines 196-204, 318-333, 344-361) could be condensed.

• Repeated statements about gray/grayish-white alteration zones (lines 578-583, 632-635, 687-690).

Response: Thank you for your constructive suggestion. Thank you for your constructive suggestions. We have checked the manuscript for grammatical errors. Specifically, we have corrected exaggerated statements, non-standard sentence structures, and redundant expressions, which have greatly improved the readability of the manuscript.

No content in this article has been published elsewhere and is not under consideration for publication. We again thank the reviewers for their valuable comments, suggestions, and recommendations that helped us improve our work and hope that the revised manuscript will meet the requirements for publication in PLOS ONE. Please forward all correspondence concerning this manuscript to me at destinw@126.com.

Sincerely yours,

Qing Wang.
