# Supplementary material for: Depositional architecture and post-depositional alteration of the Toutunhe Formation (J2t) in the Louzhuangzi area, Southern Junggar Basin: Implications for uranium mineralization
Source: PLoS One. 2026 Jun 16;21(6):e0351337. doi: 10.1371/journal.pone.0351337 (PMC13271488; doi:10.1371/journal.pone.0351337)
Supplement: Attachment — Submitted filename: Reviewer Comment_Adepehin.pdf [file pone.0351337.s009.pdf]

Reviewer Report
Manuscript ID: PONE-D-25-34881R2
Title: ***Sedimentary architecture and post-depositional alteration of the Toutunhe Formation (J2t) in the Louzhuangzi area, Southern Junggar Basin: Implications for uranium mineralization***

General Evaluation
This revised manuscript is much readable. The topic is relevant and potentially valuable, particularly given the combination of field observations and micro-analytical techniques (SEM, EPMA, FIB-TEM). However, despite the promising dataset, the manuscript requires **substantial revision**. The principal shortcomings relate to **lack of methodological clarity, weak sedimentological rigor, insufficient linkage between data and interpretation, and structural inconsistencies throughout the manuscript**. In its current form, the study does not yet meet the standards required for publication.

I have some comments on the annotated PDF manuscript, which are amplified here. Authors are advised to consult both materials when revising their manuscript.

Major Comments
1. Abstract and Statement of Purpose
The abstract is overly long, not well structured and does not clearly articulate the research objective.

- Lines 18–22: The abstract emphasizes methodology prematurely rather than clearly stating the **aim of the study**. As noted in the manuscript, *“this sentence needs re-framing… the latter part should establish the purpose of the study rather than highlighting methodology.”*
- The abstract length should also be verified against journal requirements.
-

Recommendation:
Reorganize the abstract to follow a clear structure: background → objective → methods → key findings → implications.

2. Inadequate Literature Context
The introduction lacks sufficient engagement with key literature.

- Line 66: The manuscript discusses uranium mineralization controls but does not cite **landmark studies** (*“Please name some landmark papers relating to this here”*).
- Lines 74–75 and 88–90: Statements are made without citations (*“source??”*).

Recommendation:
Strengthen the literature review by incorporating foundational and recent studies on:

- Sandstone-hosted uranium systems
- Sedimentary controls on mineralization

- Regional geology of the Junggar Basin

3. Geological Background – Conceptual and Factual Issues
The geological background section is problematic.

- Lines 150–154: The tectonic description is **misleading and oversimplified**, as highlighted. How can the Jungar Basin be located on Kazakhstan?
- Line 154: Timing of tectonic evolution is unclear
- Line 161: Citation required but missing.
-

Recommendation:
Reconstruct this section using established regional geological frameworks with proper chronological and tectonic context, supported by appropriate references.

4. Methodology – Major Deficiencies
The Methods section lacks essential detail and reproducibility.
Key omissions:

- Lines 199–206:
  - No clarity on whether cores were **collected by the authors or sourced from a company**
  - Missing details on **number of wells, sampling intervals, and total samples**
- Lines 221–224:
  - No indication of **number of thin sections prepared**
- Lines 225–250:
  - Number of samples analyzed via SEM/EPMA/TEM not specified
- Lines 216–220:
  - Sampling strategy is described qualitatively but not quantitatively

Recommendation:
Provide:

- Total number of samples (field, thin section, SEM, EPMA, TEM)
- Source of core materials
- Sampling rationale and representativeness
- Analytical uncertainties

5. Sedimentary Facies Analysis – Lack of Rigor
This is one of the most critical weaknesses of the manuscript.

- Lines 373–382:
  - Facies interpretations (e.g., floodplain, levee, channel-fill) are presented **without supporting evidence**
- Lines 386–398:
  - No clear criteria used for facies classification
- Lines 408–413:
  - The Results section improperly includes references and interpretations

Recommendation:
Rebuild the sedimentological analysis using a standard workflow:

1. Observations (grain size, structures, geometry)
2. Interpretation (process-based)
3. Facies classification (with explicit criteria)

6. Mixing of Methods and Results

- Lines 289–303:
    - Petrographic quantification (QFL analysis, compositional maturity) appears in Results but lacks methodological explanation

Recommendation:
Move all methodological descriptions (e.g., modal analysis procedures) into the Methods section.

7. Results Section – Lack of Clarity and Structure
The Results section is largely descriptive and lacks analytical depth.

- Lines 253–261:
    - Contains vague and unsupported statements
- Line 263:
    - Content flagged as inappropriate placement

Recommendation:
Please ensure that:

- Results present **only observations**
- Interpretations citations of papers are deferred to the Discussion
- All statements are supported by figures, tables, or data

8. Poor Link Between Data and Uranium Mineralization Model

- The manuscript proposes a conceptual model but does not sufficiently demonstrate:
    - How sedimentary architecture controls permeability
    - How diagenesis influences uranium precipitation
    - How micro-scale observations relate to basin-scale processes

Recommendation:
Strengthen integration between:

- Facies architecture
- Fluid flow pathways
- Redox conditions
- Mineralization processes

9. Overuse of Unsupported Interpretations
Throughout the manuscript, interpretations are made without direct evidence.

Examples include:

- Lines 373–399: Facies assignments without diagnostic criteria
- Lines 408–426: Mineralization controls described without clear data linkage

Recommendation:
All interpretations must be explicitly supported by:

- Observational data
- Figures or tables
- Established sedimentological principles

10. Figures and Supporting Evidence

- Figures are not effectively used to support interpretations
- Lack of explicit cross-referencing between text and figures

Recommendation:

- Improve figure captions (interpretive, not descriptive)
- Explicitly link interpretations to figures

Minor Comments

1. Language and Grammar

- Numerous grammatical and stylistic issues throughout
- Example: Line 389 (*"The ore-bearing main sandstone…"*) requires rephrasing

2. Terminology

- Inconsistent use of sedimentological and mineralogical terminology

3. Referencing

- Multiple missing citations (e.g., lines 74–75, 161)

4. Organization

- Some content appears misplaced or redundant (e.g., petrographic data in Results without methodological context)

Additional Observations

- The manuscript lacks a clear **conceptual framework linking sedimentology, diagenesis, and mineralization**
- Temporal relationships between:
    - Deposition
    - Diagenesis. I feel this control on Uranium mineralization is poorly crafted in the study. I made references to some of my works and the need for authors to discuss paragenetic sequence as it relates to the focus of the study.
    - Mineralization
      remain unclear
- Microanalytical data (SEM/TEM) are not sufficiently integrated with field-scale observations

Recommendation

Major Revision

The manuscript requires **substantial restructuring and methodological clarification** before it can be considered for publication. The dataset is valuable, but significant improvements are necessary in:

- Sedimentological rigor
- Methodological transparency
- Evidence-based interpretation
- Overall organization

Closing Remark

The study has the potential to make a meaningful contribution to the understanding of sandstone-hosted uranium systems. However, this will only be realized after **major revision focused on clarity, rigor, and integration of data and interpretation**. I am happy to look at the paper again.

Ekundayo Adepehin
jadepehin@gmail.com
