# Supplementary material for: Depositional architecture and post-depositional alteration of the Toutunhe Formation (J2t) in the Louzhuangzi area, Southern Junggar Basin: Implications for uranium mineralization
Source: PLoS One. 2026 Jun 16;21(6):e0351337. doi: 10.1371/journal.pone.0351337 (PMC13271488; doi:10.1371/journal.pone.0351337)
Supplement: Attachment — Submitted filename: Review Comments to the Author.docx [file pone.0351337.s005.docx]

***Review Comments to the Author***

**General Evaluation**

The manuscript offers a detailed geological, sedimentological, and mineralogical study of uranium mineralization in the Toutunhe Formation, Junggar Basin, using fieldwork and microanalytical methods. Although it has scientific value and potential to advance knowledge on sandstone-type uranium deposits, significant revisions are needed before it is suitable for publication in PLOS One.

**Major Points for Revision**

1. The introduction should clearly state the research objectives and hypotheses to guide the reader.
2. The manuscript lacks a clear, standalone methodology section, making it hard to separate methods from background and results. A dedicated “Materials and Methods” section with labeled subsections is recommended for clarity.
3. The manuscript lacks detailed geochemical analysis, such as elemental concentrations, ratios, or diagrams (e.g., Harker or discrimination plots) that could support the interpretation of provenance, alteration, and uranium enrichment processes.
4. The study heavily relies on SEM, EMPA, and FIB-TEM observations. However, no XRD or quantitative mineral abundance data are provided. Including XRD would validate the qualitative and semi-quantitative identification of mineralogical phases within crystalline materials.
5. The Results section in the manuscript is informative but not clearly delineated as a standalone section. The findings (e.g., petrography, depositional environment, uranium mineralization) are integrated within broader descriptive sections, making it hard to distinguish between background, methodology, and results.
   5.1 The text mentions the presence of pyrite and organic debris but does not sufficiently explore their role in uranium precipitation. A more focused discussion on their redox buffering capacity is recommended.
   5.2 The manuscript provides Q/ (F+R) ratios to indicate compositional maturity, but the significance of these values in the context of uranium mobility and deposition should be elaborated.
   5.3 In several parts of the manuscript, it is unclear whether certain alterations (e.g., chloritization, sericitization) are of early diagenetic or late-stage epigenetic origin.
6. The manuscript lacks a clearly explained discussion section. To improve scientific rigor, the authors should provide a separate and well-structured discussion that interprets key findings, compares them with previous studies, and explains their implications for uranium mineralization processes.
7. Summarize key findings and explicitly discuss how the study informs future uranium exploration efforts in the Junggar Basin and comparable basins.
8. The manuscript uses author–year (Harvard-style) citations format, but PLOS ONE requires numbered references in Vancouver style. Please revise all in-text citations and the reference list to match the required format.

**Recommendation:** Major Revision
