# Supplementary material for: Depositional architecture and post-depositional alteration of the Toutunhe Formation (J2t) in the Louzhuangzi area, Southern Junggar Basin: Implications for uranium mineralization
Source: PLoS One. 2026 Jun 16;21(6):e0351337. doi: 10.1371/journal.pone.0351337 (PMC13271488; doi:10.1371/journal.pone.0351337)
Supplement: Attachment — Submitted filename: Response to Reviewers.docx [file pone.0351337.s006.docx]

*Editor-in-Chief,*

*PLOS ONE,*

*Dear Editor,*

Thank you for your constructive comments that were very helpful in improving the quality of this manuscript. We have checked the comments carefully and made corresponding modifications in the manuscript. We have submitted a revised manuscript titled “**Control of deposition-epigenetic alteration of sandbody on uranium mineralization in Toutunhe Formation, Louzhuangzi area, Southern Margin of Junggar Basin, China**” for consideration for publication in *PLOS ONE*.

We have conducted a complete revision based on your comments and those from two reviewers. The major changes are marked in red in the manuscript. The most important revisions are:

**Journal requirements:**

When submitting your revision, we need you to address these additional requirements.

1. Please ensure that your manuscript meets PLOS ONE's style requirements, including those for file naming. The PLOS ONE style templates can be found at

https://journals.plos.org/plosone/s/file?id=wjVg/PLOSOne_formatting_sample_main_body.pdf and

<https://journals.plos.org/plosone/s/file?id=ba62/PLOSOne_formatting_sample_title_authors_affiliations.pdf>

**Response:** We appreciate the editor’s reminder regarding manuscript formatting and file naming requirements. In the revised submission, we have carefully formatted the manuscript in accordance with the PLOS ONE style guidelines, including the use of appropriate section headings, reference style, figure and table placement, and file naming conventions, as outlined in the provided templates:

PLOS Manuscript Body Formatting Guidelines

PLOS Affiliations Formatting Guidelines

All manuscript files have been renamed following PLOS ONE’s file naming instructions. We hope the revised version now fully complies with the journal’s formatting requirements.

2. In your Methods section, please provide additional information regarding the permits you obtained for the work. Please ensure you have included the full name of the authority that approved the field site access and, if no permits were required, a brief statement explaining why.

**Response:** This study was conducted with the support of a joint innovation project between East China University of Technology and the No. 216 Geological Party, China National Nuclear Corporation (CNNC). Field access was granted by the No. 216 Geological Party. Both institutions jointly carried out the research, and contributors from each party have been appropriately acknowledged in the author information section. Therefore, no additional fieldwork permission document is required.

3. Thank you for stating the following financial disclosure:

[National Natural Science Foundation of China (No. U2244205 and No. U2067202) and the Joint Innovation Fund Project of China Uranium Industry Company Limited - State Key Laboratory of Nuclear Resources and Environment, East China University of Technology (2023NRE-LH-10).].

Please state what role the funders took in the study. If the funders had no role, please state: "The funders had no role in study design, data collection and analysis, decision to publish, or preparation of the manuscript."

If this statement is not correct you must amend it as needed.

Please include this amended Role of Funder statement in your cover letter; we will change the online submission form on your behalf.

**Response:** Thank you for your guidance regarding the financial disclosure.

In this study, the field geological investigation and sample preparation were financially supported by the National Natural Science Foundation of China (No. U2244205 and No. U2067202). The laboratory analytical work was supported by the Joint Innovation Fund Project of China Uranium Industry Company Limited – State Key Laboratory of Nuclear Resources and Environment, East China University of Technology (2023NRE-LH-10).

This amended Role of Funder statement has been included in the cover letter as requested.

4. If the reviewer comments include a recommendation to cite specific previously published works, please review and evaluate these publications to determine whether they are relevant and should be cited. There is no requirement to cite these works unless the editor has indicated otherwise.

**Response:** Thank you for the clarification regarding reviewer-suggested citations.

We have carefully read reviewers’ comments and have not found any reviewers suggesting citing specific published literature. Those that were not directly relevant to the scope or findings of our study have not been cited, in accordance with the editorial guidance that citation is not mandatory unless explicitly required.

We appreciate the opportunity to critically assess and refine our reference list.

**Reviewer 1:** This manuscript presents a well-executed study on uranium mineralization in the Toutunhe Formation, integrating analysis of depositional environments with epigenetic alteration processes. The research is methodologically robust, employing field surveys, petrographic studies, and advanced microscopy techniques to elucidate the controls on uranium mineralization. The findings are novel and make a meaningful contribution to the understanding of sandstone-type uranium deposits.

The study effectively addresses key questions about the depositional and epigenetic factors influencing uranium mineralization.

The use of SEM, EMPA, and FIB-TEM provides detailed insights into uranium occurrence and mineral associations, where the identification of brannerite and the proposed role of hydrocarbon reduction are notable advantages.

The manuscript is well-organized, with logical flow and clear presentation of results.

**Specific modifications：**

1. Thermal Fluids: Further discussion on the origin and properties of thermal fluids would enhance the understanding of their role in mineralization.

**Response:** Thank you for your constructive suggestions.

We have expanded the discussion on the possible origin and properties of the thermal fluids and clarified the evidentiary basis for our interpretations. At present, we have not conducted fluid inclusion analyses; therefore, our conclusions are preliminary and derive from mineral assemblage and alteration features (e.g., chloritization, clay–pyrite–bitumen associations, and Ti–U aggregates/brane­nerite-like nanoparticles) observed at micro- to nanoscale. These textures are consistent with moderately elevated-temperature, reducing fluids superimposed on earlier oxidation. We now explicitly note this limitation and outline future work (fluid inclusion microthermometry, Raman/GC–MS of inclusions, and stable isotope analyses) that will better constrain fluid sources, temperatures, and timing.

The expanded discussion appears in the revised manuscript at Lines 731-744.

2. Hydrocarbon Evidence: Additional data, such as fluid inclusions with more analysis, could strengthen the hydrocarbon reduction hypothesis.

Some bulk sample analyses are needed to give an overview about the nature of the samples and their matrix.

**Response:** Thank you for your constructive suggestion. We also agree with the reviewer that providing bulk geochemical or mineralogical characterizations would help clarify the nature of the samples and their matrix. In the revised manuscript, we have further elaborated on the hydrocarbon reduction hypothesis by citing supporting evidence from previously published work (He et al., 2024). Although the current dataset is limited, the available evidence suggests a potential link between migrated hydrocarbons and localized reducing conditions favorable for uranium precipitation. We acknowledge that future studies involving Fluid inclusions, Raman spectroscopy or GC–MS analyses are needed to provide more definitive evidence, and we consider this an important direction for ongoing research.

The relevant revisions can be found in Lines 677-690 of the revised manuscript.

**Reviewer 2:** The manuscript offers a detailed geological, sedimentological, and mineralogical study of uranium mineralization in the Toutunhe Formation, Junggar Basin, using fieldwork and microanalytical methods. Although it has scientific value and potential to advance knowledge on sandstone-type uranium deposits, significant revisions are needed before it is suitable for publication in PLOS One.

**Specific modifications：**

1. The introduction should clearly state the research objectives and hypotheses to guide the reader.

**Response:** Thank you for your constructive suggestions. We fully agree that the study objectives and hypotheses should be clearly stated in the introduction to guide the reader. In the revised introduction (lines 95-105), we clarify the overall study objective: to elucidate the sedimentary-epigenetic controls on uranium mineralization in the Toutunhe Formation (J2t) in the Louzhuangzi area using a comprehensive approach of sedimentology, mineralogy, and microanalysis. We also clarify the working hypothesis: the coupling between sedimentary facies, epigenetic fluid alteration, and hydrocarbon reduction controls the observed uranium mineralization.

2. The manuscript lacks a clear, standalone methodology section, making it hard to separate methods from background and results. A dedicated “Materials and Methods” section with labeled subsections is recommended for clarity.

**Response:** Thank you for your constructive suggestions. A new “3. Materials and Methods” section has been created with labeled subsections for clarity. These include: Fieldwork and Sampling, Petrography, SEM–EDS, EPMA, FIB–TEM Sample Preparation, and TEM–EDS Analysis. Methods and instrumental settings (e.g., beam current, accelerating voltage, software) have been clearly described. (Lines 152-181).

3. The manuscript lacks detailed geochemical analysis, such as elemental concentrations, ratios, or diagrams (e.g., Harker or discrimination plots) that could support the interpretation of provenance, alteration, and uranium enrichment processes.

**Response:** Thank you for your constructive suggestion. We agree that detailed geochemical analysis such as element concentration, ratio or graph (e.g., Harker graph or discrimination graph) will provide valuable support for explaining the source, weathering and uranium enrichment processes. However, we note that geologists at our institution have conducted thorough investigations in their work (He, 2025). Since the paper has not been officially published online yet, we avoid citing it to support our current research. Furthermore, we clearly stated in Chapter 5.5 that future work will focus on obtaining main and trace element concentrations (e.g. uranium, titanium, iron, aluminum, thorium, vanadium), element ratios (e.g. titanium/zirconium, thorium/uranium, uranium/ferric), and rare earth element (REE) patterns to depict source signals and hydrothermal superposition. We will also introduce discriminant graphs such as Hakgraph and Thorium/uranium vs. Uranium/iron double-plot to visualize alteration trends and uranium mobility. We appreciate the commenters for their understanding and again for your insights.

4. The study heavily relies on SEM, EMPA, and FIB-TEM observations. However, no XRD or quantitative mineral abundance data are provided. Including XRD would validate the qualitative and semi-quantitative identification of mineralogical phases within crystalline materials.

**Response:** Thank you for your constructive comment. We acknowledge this limitation. Due to the limited quantity of samples available for destructive analysis, XRD analysis was not conducted in this study. We have explicitly noted this limitation in the Discussion section (Lines 722-729), and suggested that future work should include XRD and QEMSCAN analyses to validate the mineralogical identifications.

5. The Results section in the manuscript is informative but not clearly delineated as a standalone section. The findings (e.g., petrography, depositional environment, uranium mineralization) are integrated within broader descriptive sections, making it hard to distinguish between background, methodology, and results.

**Response:** Thank you for your constructive suggestion. We have reorganized the manuscript to establish a standalone “6. Conclusions and Implications for Uranium Exploration” section, which now includes five subsections: sedimentary controls on mineralization, uranium mineral assemblages, multi-stage alteration, redox buffering processes and implications for uranium exploration. This restructuring improves clarity and distinguishes the research findings from the background and methodology (Lines 759-783).

5.1 The text mentions the presence of pyrite and organic debris but does not sufficiently explore their role in uranium precipitation. A more focused discussion on their redox buffering capacity is recommended.

**Response:** Thank you for your constructive suggestions. In the revised manuscript, we have strengthened the discussion of the redox buffering role of pyrite and organic matter in uranium precipitation. Specifically, we discuss their potential to reduce uranium⁶⁺ and promote the precipitation of pitchblende and uraninite. These discussions have been incorporated into the Discussion section (Lines 677-694).

5.2 The manuscript provides Q/ (F+R) ratios to indicate compositional maturity, but the significance of these values in the context of uranium mobility and deposition should be elaborated.

**Response:** Thank you for your constructive suggestions. In the revised manuscript, we have clarified the significance of the Q/(F+R) ratios. We explain that these values (0.22–1.44) reflect lower compositional maturity and are related to porosity evolution, uranium remobilization potential, and cation exchange buffering during mineralization. See the Discussion (Lines 657-675).

5.3 In several parts of the manuscript, it is unclear whether certain alterations (e.g., chloritization, sericitization) are of early diagenetic or late-stage epigenetic origin.

**Response:** We thank the reviewer for pointing this out. We revised the relevant paragraphs to specify timing: e.g., chloritization is interpreted as early diagenetic, whereas sericitization and Ti–U phase replacement are epigenetic. These changes are reflected in the Discussion (Lines 696-713).

6. The manuscript lacks a clearly explained discussion section. To improve scientific rigor, the authors should provide a separate and well-structured discussion that interprets key findings, compares them with previous studies, and explains their implications for uranium mineralization processes.

**Response:** Thank you for your constructive suggestions. We have created a structured "5. Discussion" section with five subheadings: 5.1 Controls on Uranium Mineralization: Sedimentary Facies and Diagenesis, 5.2 Sandstone compositional maturity and its impact on uranium mobility, 5.3 Redox Buffering Role of Pyrite and Organic Matter, 5.4 Timing and Genetic Stage of Mineral Alteration, 5.5 Geochemical Indicators and Mineralogical Constraints, 5.6 Origin and Nature of Thermal Fluids and 5.7 Uranium mineralization model. This has enhanced the depth of our interpretation (Lines 647-755).

7. Summarize key findings and explicitly discuss how the study informs future uranium exploration efforts in the Junggar Basin and comparable basins.

**Response:** Thank you for your constructive suggestions. We have added a new section, "6. Conclusions and Implications for Uranium Exploration," to the revised manuscript. This section summarizes the main findings and outlines their practical implications for future exploration in the Junggar Basin and similar basins (Lines 778-783).

8. The manuscript uses author–year (Harvard-style) citations format, but PLOS ONE requires numbered references in Vancouver style. Please revise all in-text citations and the reference list to match the required format.

**Response:** Thank you for pointing out the formatting error in the manuscript. As requested, we have revised all in-text citations and reference list entries to conform to the Vancouver (numbered) format. For example, "(Geng et al., 2020; Wang et al., 2021)" has now been corrected to "[1,6]".

No content in this article has been published elsewhere and is not under consideration for publication. We again thank the reviewers for their valuable comments, suggestions, and recommendations that helped us improve our work and hope that the revised manuscript will meet the requirements for publication in PLOS ONE. Please forward all correspondence concerning this manuscript to me at [destinw@126.com](mailto:destinw@126.com).

Sincerely yours,

Qing Wang.
