# Supplementary material for: Depositional architecture and post-depositional alteration of the Toutunhe Formation (J2t) in the Louzhuangzi area, Southern Junggar Basin: Implications for uranium mineralization
Source: PLoS One. 2026 Jun 16;21(6):e0351337. doi: 10.1371/journal.pone.0351337 (PMC13271488; doi:10.1371/journal.pone.0351337)
Supplement: Attachment — Submitted filename: Response_to_Reviewers_auresp_2.docx [file pone.0351337.s007.docx]

*Editor-in-Chief,*

*PLOS ONE,*

*Dear Editor,*

Thank you for your constructive comments that were very helpful in improving the quality of this manuscript. We have carefully reviewed the comments and made corresponding revisions to the manuscript. We have submitted the second revised manuscript to PLOS ONE, entitled " **Control of deposition-epigenetic alteration of sandbody on uranium mineralization in Toutunhe Formation, Louzhuangzi area, Southern Margin of Junggar Basin, China** ".

We have comprehensively revised the paper based on the reviewers' comments. The main revisions are marked in red in the manuscript. The most important revisions are as follows:

# Reviewer #2:

The authors have addressed the comments appropriately, and the manuscript is now suitable for publication.

**Response:** Thank you for your constructive suggestions. We sincerely thank the reviewer for the careful evaluation and positive assessment of our manuscript. The comments and suggestions provided during the first round of review were essential in substantially improving the quality of this paper, and we are very grateful for your continued support.

# Reviewer #3:

Review of the manuscript titled “Control of deposition-epigenetic alteration of sandbody on uranium mineralization in Toutunhe Formation, Louzhuangzi area, Southern Margin of Junggar Basin, China”, submitted to the journal “Plos One”.

**Response:** We sincerely thank you for taking the time to review our manuscript and for providing constructive comments. Your suggestions have been crucial in improving the quality of this paper, and we have revised the manuscript accordingly, as detailed below:

**Specific modifications：**

1. - Line 15: Remove “shows” and use has instead of have: “In recent years, some uranium mineralization with industrial values has been discovered in ….”

**Response:** Thank you for your constructive suggestions.

**Original:** In recent years, some uranium mineralization shows with industrial values have been discovered in the Louzhuangzi area, southern margin of the Junggar Basin, and a medium-sized uranium mineral area has been confirmed.

**Revised:** In recent years, some uranium mineralization with industrial values has been discovered in the Louzhuangzi area on the southern margin of the Junggar Basin, and a medium-sized uranium mineralized area has been confirmed.

2. - Line 21: use “in the Toutunhe Formation (J_2_*t*) of the Louzhuangzi area …”, and you can remove “of the Louzhuangzi area” from line 27.

**Response:** Thank you for your constructive suggestions. We have revised the text accordingly by specifying “in the Toutunhe Formation (J₂t) of the Louzhuangzi area” in the first occurrence and removing the redundant phrase “of the Louzhuangzi area” from the later sentence. These revisions improve clarity and avoid repetition.

**Original:** In this study, the depositional characteristics of the sandbodies in the Toutunhe Formation (J2t) has been revealed through field geological investigation, collation of drill core data and petrographic analysis; the occurrence state of uranium and alteration characteristics of minerals coexisting with uranium has been explored by using scanning electron microscope (SEM), electron probe (EMPA), and focused ion beam scanning electron microscopy (FIB)- transmission electron microscopy (TEM). Comprehensive studies show that the uranium mineralization in the Toutunhe Formation (J2t) of the Louzhuangzi area is controlled by the coupling of multiple factors such as the gravel braided river channel lag deposits, the multi-stage alteration of epigenetic - thermal fluids, and the reduction of oil and gas.

**Revised:** In this study, the depositional characteristics of the sandbodies in the Toutunhe Formation (J₂t) of the Louzhuangzi area are investigated through field geological surveys, drill-core data compilation, and petrographic analysis. The modes of uranium occurrence and the alteration characteristics of uranium-associated minerals are examined using scanning electron microscopy (SEM), electron probe microanalysis (EPMA), and focused ion beam scanning electron microscopy–transmission electron microscopy (FIB–TEM). The results indicate that uranium mineralization in the Toutunhe Formation (J₂t) is controlled by the coupling of multiple factors, including gravelly braided-river channel lag deposits, multi-stage epigenetic–hydrothermal fluid alteration, and hydrocarbon-related reduction.

3. - Line 36: Turpan-Hami, Songliao Basins, and the northern…

**Response:** Thank you for your constructive suggestion. We have revised the sentence to improve clarity and consistency in the listing of basins by standardizing basin names and refining the wording. The revised text now clearly indicates that sandstone-type uranium deposits in northern China are mainly distributed in Mesozoic-Cenozoic fore-mountain basins, including the Ili, Turpan-Hami, Songliao, and Ordos basins.

**Original:** The sandstone-type uranium deposits in northern China are mainly found in the Mesozoic and Cenozoic fore-mountain basins, such as the southern margins of the Ili, Turpan-Hami, and Songliao Basins, and the northern part of the Ordos Basin.

**Revised:** Sandstone-type uranium deposits in northern China are mainly distributed in Mesozoic-Cenozoic foreland basins, such as Ili, Turpan-Hami, and Songliao basins, as well as the Ordos Basin.

4. - Line 41: mineralizations

**Response:** Thank you for your constructive suggestion. We have revised the sentence by changing “mineralization” to “mineralizations” to reflect the occurrence of multiple uranium mineralized bodies, and slightly refined the wording to improve clarity and readability.

**Original:** Some promising uranium mineralization has been discovered in the Jurassic, Cretaceous and Paleogene sandstones in the Dingshan area of the northern basin, the Kamusite area of the eastern basin and the southern margin of the basin, which has attracted high attention from the industry.

**Revised:** Some promising uranium mineralizations have been discovered in Jurassic, Cretaceous, and Paleogene sandstones in the Dingshan area of the northern basin, the Kamusite area of the eastern basin, and along the southern margin of the basin, attracting considerable attention from the uranium exploration industry.

5. Lines 49-53: The sentence is very long.

**Response:** Thank you for your constructive suggestion. We have revised this part by splitting the long sentence into two shorter sentences, which improves clarity and readability while preserving the original meaning.

**Original:** The uranium-enriched geological bodies of the Northern Tianshan Mountains and the conglomerate layers in the bottom of the Lower Cretaceous Qingshuihe Formation provide uranium source for the uranium mineralization, and the relatively stable structure areas are the favorable locations for the mineralization of uranium.

**Revised:** The uranium-enriched geological bodies of the Northern Tianshan Mountains, together with conglomerate layers at the base of the Lower Cretaceous Qingshuihe Formation, provide important uranium sources for mineralization. In addition, relatively stable structural areas constitute favorable sites for uranium mineralization.

6. - Line 54: …during the period from the Late Cretaceous to the Paleogene. The Middle and…

**Response:** Thank you for your constructive suggestion. We have revised the text by restructuring the sentence and separating it into two clearer statements. This revision improves readability and more clearly expresses the temporal relationship between tectonic stability and uranium mineralization.

**Original:** The southern margin of the Junggar Basin was in a tectonically stable state during the Late Cretaceous to Paleogene period; the Middle and Lower Jurassic series received long-term modification by the uranium- and oxygen- containing water, resulting in the formation of a large amount of uranium mineralization.

**Revised:** The southern margin of the Junggar Basin remained tectonically stable during the period from the Late Cretaceous to the Paleogene. The Middle and Lower Jurassic strata experienced long-term interaction with uranium- and oxygen-bearing fluids, leading to the formation of significant uranium mineralization.

7. - Line 78: Use the full name for Si, P and Y.

**Response:** Thank you for your constructive suggestion. We have revised the sentence by replacing Si, P, and Y with their full oxide forms (SiO₂, P₂O₅, and Y₂O₃), which improves clarity and consistency with standard geochemical reporting.

**Original:** The components of uranium in this ore site are dominated by pitchblende and the unknown uranium minerals rich in elements such as Si, P and Y, with less brannerite and adsorbed uranium.

**Revised:** Uranium at this ore site is mainly hosted by pitchblende and unidentified uranium minerals enriched in SiO₂, P₂O₅, and Y₂O₃, with subordinate brannerite and adsorbed uranium.

8. - Line 79-82: The sentence is very long. - Line 81: determines that the mineralization…

**Response:** Thank you for your constructive suggestion. We have revised this part by restructuring the long sentence into two clearer statements and explicitly using “determines that” to improve grammatical correctness and readability. The revised text more clearly explains the genetic control of epigenetic oxidation fluids on uranium mineralization and its implications for in-situ leaching.

**Original:** The uranium mineralization uniquely derived from epigenetic oxidation fluid alteration and the in-situ leaching mining method of the sandstone-type uranium deposit determine the mineralization and production of uranium are closely related to the deposition-epigenetic alteration of the uranium-bearing sandbodies.

**Revised:** Uranium mineralization in sandstone-type uranium deposits is primarily derived from epigenetic oxidizing fluid alteration. This genetic characteristic determines that both uranium mineralization and uranium production by in-situ leaching are closely related to the depositional features and subsequent epigenetic alteration of uranium-bearing sandbodies.

9. - Lines 86-94: this is a paragraph that should consist of at least 3 sentences, not one sentence. Avoid long sentences. Same in lines 110-116, divide the paragraph into sentences.

**Response:** Thank you for your constructive suggestion. We have revised both paragraphs by dividing the original long sentences into multiple shorter sentences and reorganizing the text to improve clarity and readability. The revised paragraphs now consist of several concise sentences that better convey the scientific content and logical relationships.

**Original:** 86-94: In order to explore the controlling effect of sandbody deposition-epigenetic alteration on uranium mineralization in Toutunhe Formation (J2t) in the southern margin of Junggar Basin, a study on the deposition-epigenetic alteration characteristics of the uranium-bearing sandbodies has been carried out guided by the uranium mineralization theory related to epigenetic oxidation fluid alteration, sedimentology and alteration mineralogy, and the depositional environment of the uranium-bearing sandbodies and the properties of the ore-forming fluids have been determined, providing a reference for determining the future uranium exploration direction in the basin. 110-116: Since the stable block in the Tarim region was located at the center of the compression, the lithospheric mantle of the stable block gradually thickened under compression, whereas the rock mass of the Tianshan Orogenic Belt, as the buffer and release area of the compressive stress, gradually rose and uplifted, and finally formed the current tectonic and geomorphic pattern of the Junggar Basin after long period of geological evolution, which is separated from the Tuha and Ili Basins by mountains.

**Revised:** 86-94: To investigate the controlling effects of sandbody deposition and epigenetic alteration on uranium mineralization in the Toutunhe Formation (J₂t) at the southern margin of the Junggar Basin, we conducted a systematic study of the depositional and alteration characteristics of uranium-bearing sandbodies. This work was guided by established theories of uranium mineralization related to epigenetic oxidizing fluid alteration, sedimentology, and alteration mineralogy. The depositional environments of the uranium-bearing sandbodies and the physicochemical properties of the ore-forming fluids were thereby constrained, providing a geological basis for future uranium exploration in the basin. 110-116: During regional compression, the Tarim stable block was located near the center of the compressive regime, resulting in progressive thickening of its lithospheric mantle. In contrast, the Tianshan Orogenic Belt acted as a buffer and stress-release zone, where crustal shortening and uplift gradually intensified. Through prolonged geological evolution, these processes ultimately shaped the present tectonic and geomorphic framework of the Junggar Basin, which is now separated from the Tuha and Ili basins by intervening mountain ranges.

10. - Line 102: …mineralization (Fig 19). Is it Figure 19 or Reference 19?

**Response:** Thank you for your constructive suggestion. The citation referred to Figure 19. However, considering that figure citations in the Introduction may be potentially misleading to readers, we have removed the figure reference from this sentence in the revised manuscript.

**Original:** We hypothesize that the formation and preservation of uranium mineralization are controlled by the combined effects of (1) braided channel sandbody architecture favorable for fluid migration, (2) multiple stages of epigenetic fluid alteration involving oxidizing, thermal, and reducing fluids, and (3) localized redox interfaces induced by hydrocarbons and sulfide mineralization (Fig 19).

**Revised:** We hypothesize that the formation and preservation of uranium mineralization are controlled by the combined effects of (1) braided channel sandbody architecture favorable for fluid migration, (2) multiple stages of epigenetic fluid alteration involving oxidizing, thermal, and reducing fluids, and (3) localized redox interfaces induced by hydrocarbons and sulfide mineralization.

11. - Line 124: the orientation “N-WW” seems incorrect.

**Response:** Thank you for your constructive suggestion. After rechecking the regional structural data, we confirm that both the Yilianhabirga Mountains and the Bogda Mountain tectonic belt trend NW–SE. The previously used orientations (N-WW and N-E) were incorrect descriptions in the original manuscript. These errors have now been corrected to NW–SE in the revised version to ensure structural accuracy.

**Original:** In recent years, the Louzhuangzi uranium mineral area has been discovered in the intersection and subduction site of the N-WW oriented Yilianhabirga Mountains and the western sloping end of the N-E oriented Bogda Mountain tectonic belt (Haojiagou Uplift) (Fig 1B) in the fore-mountain fold-and-thrust belt of the Northern Tianshan Mountains.

**Revised:** In recent years, the Louzhuangzi uranium mineral area has been discovered at the intersection between the NW–SE–trending Yilianhabirga Mountains and the western plunging end of the Bogda Mountain tectonic belt (Haojiagou Uplift) (Fig. 1B), within the foreland fold-and-thrust belt of the Northern Tianshan Mountains.

12. - The quality of most of your Figures is bad. The content, essentially the texts, is unreadable (e.g., Figures 1, 2, 8, 9, 13, 14? 18). Please provide a high-quality version of your Figures.

**Response:** Thank you for your constructive suggestion. The reduced image quality observed in the current version is primarily due to compression applied during the manuscript submission and system-generated preview process. In the next (third-round) submission, we will replace all affected figures with uncompressed, high-resolution versions to ensure that all text, symbols, and details are clearly legible. If required by the editorial office, we are also happy to provide the original CorelDRAW source files or other high-resolution figure formats upon request.

13. - Line 150: use “stratigraphic column of Louzhuangzi area…” instead of “Composite stratigraphic column of Louzhuangzi area…”

**Response:** Thank you for your constructive suggestion. We have revised the figure caption by replacing “Composite stratigraphic column” with “Stratigraphic column of the Louzhuangzi area” to more accurately describe the figure.

**Original:** Fig. 2 Composite stratigraphic column of Louzhuangzi area (Modified based on the internal report from the geological party No. 216, CNNC, 2022).

**Revised:** Fig. 2 Stratigraphic column of the Louzhuangzi area (modified after an internal report from Geological Party No. 216, CNNC, 2022).

14. - Methods and Materials: label the subheads “Fieldwork and sampling, Petrographic and mineralogical analysis…” as 3.1; 3.2; …

**Response:** Thank you for your constructive suggestion. We have revised the Methods and Materials section by adding numbered subheadings, including 3.1 Fieldwork and sampling and 3.2 Petrographic and mineralogical analysis, to improve the structure and clarity of this section.

15. - Adding some field or lab pictures may be useful.

**Response:** Thank you for your constructive suggestion. We have added photographs documenting the field investigations conducted by the lead author, as well as images illustrating representative laboratory analytical procedures, as Supplementary Material at the end of the revised manuscript. If the editorial office considers these materials unnecessary, they can be readily removed from the final version.

16. - Lines 191, 193: I ask if the Figures cited are Figures 7A, 7B?

**Response:** Thank you for your constructive suggestion. We confirm that the figures cited in Lines 191 and 193 indeed refer to Figures 7A and 7B. The citations have been checked and corrected where necessary in the revised manuscript to ensure accuracy and consistency.

17. - Lines 201: I ask if the Figure cited is Figure 12?

**Response:** Thank you for your constructive suggestion. We confirm that the figure cited in Line 201 indeed refers to Figure 12. In the revised manuscript, we have carefully checked this paragraph and corrected the inaccurate figure citations. The text has been updated to explicitly reference Fig 12B for planar cross-bedding and Figs. 12J–L for the occurrence of carbonaceous debris, pyrite, and clayization, ensuring consistency between the text and the figures.

**Original:** The depositional structures such as flaser bedding (Fig 3C), parallel bedding (Fig 3A), horizontal bedding (Fig 3B), and small to medium (with the single-layer system < 10 cm) planar cross bedding (Figs 3C and 12B) are commonly developed in the sandstone. The gravel-bearing sandstone, glutenite and conglomerate revealed by drilling are grayish white (Fig 7B), containing a considerable amount of carbonaceous debris and pyrite, with clayization developed (Figs 12J, K, and L).

**Revised:** The depositional structures such as flaser bedding (Fig 3C), parallel bedding (Fig 3A), horizontal bedding (Fig 3B), and small to medium (with the single-layer system < 10 cm) planar cross bedding (Figs 3C and 12B) are commonly developed in the sandstone. The gravel-bearing sandstone, glutenite and conglomerate revealed by drilling are grayish white (Fig 7B), containing a considerable amount of carbonaceous debris and pyrite（Figs 12 J-L）, with clayization developed (Fig 12 K).

18. - Line 239: use micrometer symbol “μm” not “um” for 2600um, 500 and 1500um,…

**Response:** Thank you for your constructive suggestion. In the revised manuscript and updated figures, all instances of “um” have been corrected to the proper micrometer symbol “μm” to ensure consistency with standard scientific notation.

**Original:** The clastic particles are relatively dispersed and generally do not contact with each other (Figs 5A-F), with the particle diameters being up to 2600um, mostly between 500 and 1500um, averaging about 800um.

**Revised:** The clastic particles are relatively dispersed and generally do not contact with each other (Figs 5A-F), with the particle diameters being up to 2600 μm, mostly between 500 and 1500 μm, averaging about 800 μm.

19. - Lines 340-344: The sentence “The ore-bearing main… bar developed” is very long. Same for the next one. Same for the sentence in lines 609-614.

**Response:** Thank you for your constructive suggestion.

**Original:** 340-344: The ore-bearing main sandbody in the Lower Member of the Toutunhe Formation (J_2_*t*^1^) is composed of the thick, grayish-white coarse sandstone, glutenite, and conglomerate interspersed by the thin layers of grayish-green mudstone on the profile (Fig 7B), with the depositional microfacies such as sandy channel fill, lag deposit and gravel bar developed (Fig 8) .609-614: Titanium oxide particles can be commonly observed in the slices of the gray and grayish-white sandbodies of the Lower Member of the Toutunhe Formation (J_2_*t*^1^) under the microscope, showing obvious characteristics of epigenetic alteration, and the morphology of the particles are mostly angular to sub-angular (Figs 17A, B), with a few being circular to sub-circular (Figs 17C, D) due to epigenetic alteration, indicating that these titanium oxides are altered products of the proximal deposits..

**Revised:** 340-344: The ore-bearing main sandbody in the Lower Member of the Toutunhe Formation (J₂t₁) consists mainly of thick grayish-white coarse sandstone, glutenite, and conglomerate, with thin interlayers of grayish-green mudstone (Fig 7B). Depositional microfacies including sandy channel fills, lag deposits, and gravel bars are well developed within this sandbody (Fig 8). 609-614: Titanium oxide particles are commonly observed in thin sections of the gray and grayish-white sandbodies of the Lower Member of the Toutunhe Formation (J₂t₁). These particles display clear features of epigenetic alteration and are predominantly angular to sub-angular in morphology (Figs 17A, B). A smaller proportion exhibits circular to sub-circular shapes (Figs 17C, D), suggesting modification by epigenetic processes and indicating that these titanium oxides represent altered products of proximal deposits.

20. - Line 376: “document”? You want to say “shows”?

**Response:** Thank you for your constructive suggestion. We have revised the sentence by replacing “document” with “shows”, which more appropriately conveys the intended meaning. The sentence has also been slightly refined for clarity and consistency.

**Original:** The classification studies of the depositional facies of the outcrops and borehole strata, together with the previous study results, document that the Lower Member of the Toutunhe Formation (J2t1) contains the depositional sandbodies of braided river delta plain, sandy braided river and gravelly braided river facies deposits (Figs. 7, 8 and 9).

**Revised:** The classification of depositional facies based on outcrops and borehole strata, together with previous studies, shows that the Lower Member of the Toutunhe Formation (J₂t₁) contains sandbodies deposited in braided-river delta plain, sandy braided-river, and gravelly braided-river facies (Figs 7-9).

21. - It is recommended to discuss the main strengths and limitations of this work.

-Overall, the manuscript must be revised before sending it for the next review.

**Response:** Thank you for your constructive suggestion. In response to your comments, we have outlined the main strengths and limitations of this study in the discussion section of the revised manuscript. Specifically, we highlighted the advantages of integrating mineralogical methods and nanoscale characterization, while also pointing out current limitations, such as the limited analytical testing methods, the lack of XRD, Fluid inclusions, Bulk geochemistry, and Isotopic data support, and the need for more comparative datasets from other sandstone-type uranium deposit systems. In addition, the manuscript has undergone a comprehensive revision following your comments and those of the reviewers, including substantial restructuring, clarification of interpretations, and improvements in language, figures, and organization. We believe that these revisions have significantly improved the overall quality and clarity of the manuscript and have prepared it appropriately for the next round of review.

# Reviewer #4:

1. The abstract lacks some key aspects, including

Quantify: Add numbers to findings (e.g., "〖"CO" 〗_2purity >95%").

Clarify: Define the specific "controversy" being addressed.

**Response:** Thank you for your constructive suggestion. We have substantially revised the abstract of the original manuscript. The revised abstract strengthens the expression of research findings, and the controversies existing in current research have been adjusted to the introduction and discussed in detail.

2. Keywords: The authors may consider changing some of the keywords using one of the suggestions below:

• Sandstone-type uranium deposits (Standardized classification)

• Braided river delta (Specific depositional micro-facies from your abstract)

• Hydrocarbon-mediated reduction (The specific "how" of your mineralization)

• FIB-TEM/SEM analysis (Specific high-end methodology)

• Toutunhe Formation (Necessary stratigraphic marker)

• Redox transition zone (The geochemical environment)

• Northwest China (Broader geographic context to reach a wider audience)

**Response:** Thank you for your constructive suggestion. We have revised the Keywords section in accordance with your recommendations to better reflect the standardized deposit classification, key depositional micro-facies, principal mineralization mechanisms, applied analytical methods, stratigraphic context, and broader geographic setting. The revised keywords are provided below.

**Original:** Sedimentary environment; Epigenetic alteration; Uranium mineralization; Toutunhe Formation; Southern margin of Junggar Basin

**Revised:** Sandstone-type uranium deposits, Braided river delta, Hydrocarbon-mediated reduction, FIB-TEM/SEM analysis, Toutunhe Formation, Redox transition zone, Northwest China.

3. Are all the pictures used here original? If you take a random picture, you must have permission before using it.

Please state what role the funders took in the study. If the funders had no role, please state: "The funders had no role in study design, data collection and analysis, decision to publish, or preparation of the manuscript."

If this statement is not correct, you must amend it as needed. And include it in the cover letter, that will be enough.

**Response:** Thank you for your constructive suggestion. All figures in the manuscript are original unless specifically indicated otherwise. The roles of the funders in this study have been clearly stated in the “Author Contributions” section and further clarified in the cover letter with the following statement:“The funders made the following contributions during the research process: Conceptualization, Formal analysis, Investigation, Methodology, Project administration, and Resources.”

4. Introduction: The text needs to be solidified by adding more references; each statement requires supporting references.

**Response:** Thank you for your constructive suggestion. In the revised version, we have improved the introduction, added key issues and controversies in current research, and provided relevant references.

5. Methodology: If it's possible, the author can add a graphical abstract.

**Response:** Thank you for your constructive suggestion. Figure 19 of the paper presents the ore-controlling sand body and mineral alteration characteristics as well as the multi-stage fluid interaction process. We consider using this figure as a graphic summary of this paper.

# Reviewer #5:

1. Severe Introduction Deficiencies

The introduction (lines 32-105) lacks:

• Clear knowledge gaps: What specifically is unknown about this deposit type?

• Theoretical framework: No explicit statement of competing hypotheses.

• Literature synthesis: References are listed without critical evaluation of conflicting interpretations.

**Response:** Thank you for your constructive suggestion. In the revised version of the manuscript, we made significant changes to the introduction. Specifically, we supplemented the introduction with explanations of the problems that had existed in previous studies of this deposit, and clearly articulated the competing hypotheses.

2. Methods Section (lines 152-181)

Critical missing elements even after revision:

• Sample selection criteria and representativenes.

• Sampling Strategy Not Justified.

• Table 1 lists samples from various depths (24.84m to 871.50m) and drill holes.

• No explanation of selection criteria.

• No discussion of spatial representativeness.

• Samples concentrated in specific holes (e.g., 6 samples from ZK6-2, only 1 from ZK3-3).

• Potential sampling bias need to be addressed.

• Quality control procedures.

• Detection limits for EMPA analyses.

• Reproducibility assessment.

**Response:** Thank you for your constructive suggestion. In the revised version, we describe the sampling strategy and its sample selection criteria and representativeness. We address the criteria and representativeness of sample collection, the sampling strategy, and existing sampling bias issues. We also state the detection limit for EMPA analysis. All sample slides were properly preserved to ensure the reproducibility and verifiability of the analytical results.

EMPA Data Quality Issues (Table 2):

• Totals range from 84.01% to 97.14% - some analyses are poor quality.

• No standard reference materials mentioned.

• Single-point analyses without replication.

• No discussion of analytical uncertainty.

• Sub-optimal totals (e.g., 84.01% for brannerite) suggest volatile loss or inappropriate analytical conditions.

**Response:** Thank you for your constructive suggestion. In this revised version, we have added standard reference materials and conducted multi-point analyses of the same mineral aggregates, rather than single-point analyses. Furthermore, we have added a discussion of the uncertainties in the analytical results.

Missing Critical Techniques: The authors acknowledge (only after review) absence of:

• XRD - fundamental for phase identification.

• Fluid inclusions - critical for understanding ore-forming fluids.

• Bulk geochemistry - necessary for provenance and alteration assessment.

• Isotopic data - essential for fluid source and timing constraints.

**Response:** Thank you for your constructive suggestion. We fully understand your concerns regarding the key analytical techniques and agree with your views. We are very grateful to the reviewers for their suggestions on supplementary analytical techniques. However, our research is currently in its initial stages, and we will actively conduct relevant experimental analyses in subsequent studies to further supplement and improve the existing gaps in our research. We look forward to receiving your further guidance in future research.

The Response to Reviewers states: "Due to the limited quantity of samples available for destructive analysis, XRD analysis was not conducted". This is scientifically unacceptable because:

• XRD requires minimal sample (~100mg).

• The study collected 22 samples (Table 1).

• XRD is non-negotiable for validating mineral identifications.

• Claims about "chlorite/smectite mixed layer minerals" cannot be confirmed without XRD.

**Response:** We fully understand your concerns regarding our previous response. We acknowledge that our previous response was somewhat weak. The truth is that the samples for experimental analysis were sent to a third-party rock thin-section preparation company. Previously, we only retrieved the sample thin sections for analysis; the remaining solid block samples were not retrieved in time, resulting in a limited number of samples available for destructive analysis. We have now contacted the third-party company and retrieved some of the block samples for XRD analysis in subsequent studies. Furthermore, based on the morphology and energy dispersive spectroscopy characteristics of the clay minerals observed so far, we have initially classified them as kaolinite, montmorillonite, and chlorite. In the revised manuscript, we have revised the uncertain "chlorite/montmorillonite mixed layer minerals" to montmorillonite.

3. Results-Discussion Conflation

Despite revision, interpretive statements remain embedded in Results:

• Lines 314-315: "indicating that the type of the sandstone is greywacke and the sandbodies were formed in a proximal deposit environment" - this is interpretation.

• Lines 345-361: Depositional environment interpretation mixed with descriptive sedimentology.

• Lines 520-528: Genetic interpretations presented as observations.

**Response:** Thank you for your constructive suggestion. In the revised manuscript, we removed the explanatory statement "indicating that the type of sandstone is greywacke and the sandbodies were formed in a proximal deposit environment." Furthermore, we made significant revisions to the results section, "4.2 Depositional environment and facies interpretation." Specifically, we moved this title and related explanatory statements to the discussion section as subsection 5.1, and renamed the results section as "4.2 Sandbody Depositional Characteristics," retaining the results describing the sandbody characteristics. The related statements in lines 520-528 were also revised accordingly.

Brannerite Identification - Overstated Certainty

The manuscript claims (lines 457-467) definitive brannerite identification, but:

• Evidence presented: Only EDS element mapping showing Ti and U co-distribution.

• Missing: Electron diffraction patterns confirming brannerite crystal structure.

• TEM images (Fig. 11B-C): Show "complexly interwoven acicular aggregates" but lack lattice fringe imaging.

• Chemical composition (Table 2): TiO₂/UO₂ ratios (average 33.20%/39.62%) deviate from stoichiometric UTi₂O₆ (expected ~29.7%/63.3%).

Critical issue: The text contradicts itself - lines 467 states this is "nanoscale brannerite aggregate, rather than nanoscale coffinite or pitchblende associated with titanium oxides," but the evidence for this distinction is not compelling without crystallographic data.

**Response:** Thank you for your constructive suggestion. In the revised manuscript, we have replaced the overly confident description of “brannerite” with the more cautious term “brannerite -like nanomineral aggregates.” Our published paper (DOI: 10.1016/j.oregeorev.2025.106908) provides a detailed discussion of the electron diffraction patterns and lattice-fringe imaging associated with the crystal structure of brannerite-like phases, as well as the occurrence of nanocrystalline brannerite aggregates (rather than nanoscale uranyl silicates or pitchblende related to Ti oxides). In addition, the discrepancy between the TiO₂/UO₂ ratio of the uranium nitride–like phase in Table 2 (mean = 33.20%/39.62%) and the stoichiometric TiO₂/UO₂ ratio expected for UTi₂O₆ (approximately 29.7%/63.3%) may be attributable to its low crystallinity and/or the presence of amorphous domains.

4. Interpretive Weaknesses

(1) "Thermal Fluids" - Unsupported Speculation

The manuscript repeatedly invokes "thermal fluids" (lines 29-30, 101, 731-744, 754) but:

Evidence provided:

• Clay mineral assemblages (chlorite, chlorite-smectite).

• Brannerite occurrence.

• Sulfurization features.

Problems:

• These features can form at diagenetic temperatures (<100°C).

• No temperature constraints whatsoever.

• No fluid inclusion data.

• No isotopic evidence for deep fluid sources.

• The Discussion (lines 731-744) is essentially an admission that thermal fluids are speculative.

The authors state (Response to Reviewers): "due to the lack of direct fluid inclusion or isotopic data, the properties and pathways of these thermal fluids cannot be definitively identified".

Then why make it a central interpretive element? This represents speculation presented as conclusion.

**Response:** Thank you for your constructive suggestion. Thank you for this detailed and important critique. We agree that our previous wording and emphasis could be read as treating “thermal fluids” as a firm conclusion, whereas our dataset does not provide direct temperature constraints (e.g., fluid-inclusion microthermometry) or definitive isotopic fingerprints for a deep fluid source. Therefore, we have revised the manuscript to indicate the limitations of the current study, emphasize the inferences and conceptual models regarding fluid action, and ensure that all interpretations match the existing level of evidence.

ii. Hydrocarbon Reduction - Circumstantial Evidence

The manuscript argues (lines 69-71, 641-643, 677-690) that H₂S and CH₄ from hydrocarbon leakage caused reduction, based on:

• Presence of pyrite and bitumen.

• Regional petroleum geology.

• Citation of He et al. (2024).

Missing:

• Direct evidence of hydrocarbon fluid inclusions.

• Organic geochemistry linking bitumen to deep sources.

• Sulfur isotope data to constrain H₂S sources (biological vs. thermochemical).

• Temporal constraints on hydrocarbon migration vs. uranium precipitation.

Reviewer 1 noted: "Additional data, such as fluid inclusions with more analysis, could strengthen the hydrocarbon reduction hypothesis".

Authors' response: Acknowledge limitation and cite He et al. (2024) for supporting evidence.

Problem: This effectively outsources key evidence to another study while maintaining the interpretation as a primary conclusion.

**Response:** Thank you for your constructive suggestion. Thank you for this careful and constructive critique. We agree that our previous wording could be read as treating hydrocarbon-derived reduction (H₂S/CH₄) as a primary conclusion, whereas the evidence presented in this manuscript is largely indirect (bitumen + pyrite occurrence and regional petroleum context). We have therefore revised the manuscript to ensure that the hydrocarbon-reduction interpretation is presented as a supported but non-exclusive hypothesis, explicitly limited by the absence of several key datasets.

iii. Depositional Facies Analysis - Oversimplified

The depositional environment interpretation (Section 4.2, lines 305-396) suffers from:

• Sedimentary structures described but not systematically analyzed: No paleocurrent data, no grain size distribution analysis, no facies associations diagram.

• Uncritical facies assignment: E.g., "flaser bedding... mainly formed by migration and deposition... in meandering river channel" (lines 317-320) - but flaser bedding forms in tidal settings and other environments.

•Missing quantitative data: No channel dimensions, no sand/mud ratios, no architectural element analysis.

• References misapplied: Folk (1968) cited for sedimentary environments (line 344) when it's a textural classification scheme.

**Response:** Thank you for your constructive suggestion. Thank you for this detailed and valuable critique. We agree that the original facies discussion in Section 4.2 contained (i) interpretive statements expressed too definitively, (ii) facies assignments that were not sufficiently constrained by systematic facies analysis, and (iii) an inappropriate citation (Folk, 1968) for depositional-environment interpretation. We have revised the manuscript accordingly to improve rigor and to clearly separate descriptive sedimentology (Results) from depositional-environment interpretation (Discussion).

5. DATA PRESENTATION ISSUES

(1) Table 2 - Serious Problems

Compositional Data:

• 35 analyses presented without context (which samples? which grains?).

• Mineral types assigned post-hoc based on composition.

• No clear criteria for distinguishing "dispersed adsorbed uranium" (5 analyses) from coffinite (16 analyses).

•Some "coffinite" analyses have very low SiO₂ (9.33%) approaching pitchblende composition.

Statistical treatment absent:

• No mean ± standard deviation.

• No compositional ranges by deposit location.

• No correlation analysis (e.g., UO₂ vs. TiO₂).

**Response:** Thank you for your constructive suggestion. Thank you for such detailed and important comments; your suggestions are highly valuable for improving the quality of our manuscript. We agree that the original Table 2 did not provide sufficient contextual information for the 35 analyses and that the statistical treatment of the dataset was incomplete. In the revised version, each analysis in Table 2 is now explicitly linked to its corresponding sample ID, and mean values are provided for each compositional group. Mineral-type assignments were made by integrating the petrographic/textural characteristics of the analyzed spots with their compositional signatures.

The term “dispersed adsorbed uranium” has now been revised to “Unidentified nano-uranium minerals,” and these analyses are distinguished from coffinite based on their mineral assemblage context and oxide composition characteristics. We also note that some coffinite analyses display very low SiO₂ contents approaching those of pitchblende; however, their UO₂ contents are generally lower than the overall UO₂ range measured for pitchblende.

Because the number of ore samples analyzed from different sectors of the deposit is currently limited, we did not subdivide compositional ranges by deposit location in order to avoid overinterpretation. In addition, given the clear mineralogical differences among the identified phase groups and the large variability in UO₂ and TiO₂ contents, the present dataset is insufficient to support robust correlation analysis. In future work, we will expand both the number of analyzed ore samples and the total dataset, subdivide results by deposit sector, and conduct correlation analyses to evaluate compositional trends and interrelationships.

6. DISCUSSION SECTION - SUPERFICIAL SYNTHESIS

The Discussion (added/restructured after revision, lines 646-755) has improved structure but remains problematic:

Section 5.1 (Sedimentary control):

• States braided channel facies are primary hosts (correct).

• Comparison with Ordos, Songliao, Texas deposits mentioned but not developed.

• Missing: Why is Toutunhe Formation mineralization similar/different from these analogues?

Section 5.2 (Compositional maturity):

• Q/(F+R) ratios discussed (lines 657-675).

• Links low maturity to uranium mobility – reasonable.

• Missing: Comparison with unmineralized Toutunhe sandstones - are compositional differences significant?

Section 5.3 (Redox buffering):

• Argues pyrite and organics reduced U⁶⁺ to U⁴⁺.

• Problem: This is standard ore-forming process - what's specific to Louzhuangzi?

• References [39-43] cited but not critically evaluated.

Section 5.4 (Alteration timing):

• Distinguishes early diagenetic from epigenetic alteration.

• Major gap: No geochronology, no cross-cutting relationships systematically documented.

• Relies on textural interpretation alone.

Section 5.5 (Future geochemical work):

• This is not discussion - it's an admission of missing data.

• Acknowledges need for major/trace elements, REE, Ti/Zr, Th/U ratios.

• Should have been done before publication.

Section 5.6 (Thermal fluids origin):

• Essentially states "we don't know" (lines 731-744).

• Speculation about basin-centered flow, magmatic underplating.

• No new insight beyond acknowledging uncertainty.

Section 5.7 (Mineralization model):

• Figure 19 presents conceptual model.

• Problem: Model incorporates unvalidated elements (thermal fluids, hydrocarbon timing).

• Three-stage process (oxidation → thermal overprint → hydrocarbon reduction) is plausible but not demonstrated.

**Response:** Thank you for your detailed and constructive comments. Based on the current revision status of the manuscript, we have substantially revised the Discussion section. In the revised version, we focus primarily on the preliminary findings obtained in this study and the limitations of the current dataset, and we have removed the geochemistry-oriented discussion that cannot be adequately supported by the available data. Accordingly, this manuscript presents our current interpretations while explicitly identifying the remaining uncertainties. Future work by the authors and other researchers in this area will require more comprehensive analytical approaches, including more detailed geochemical investigations, to further test and refine the proposed interpretations.

7. WRITING QUALITY AND PRESENTATION

Pervasive Issues:

i. Jargon and unclear terminology:

• "Deposition-epigenetic alteration" (title) - awkward phrasing.

• "Gravel braided river channel lag deposits" (line 28) - "gravel braided river" is non-standard (should be "gravelly braided river").

• "Uranium-bearing sandbody" - "sandbody" is informal; use "sandstone unit".

**Response:** Thank you for your constructive suggestion. We revised the awkwardly worded title to Sedimentary architecture and post-depositional alteration of the Toutunhe Formation (J₂t) in the Louzhuangzi area, Southern Junggar Basin: Implications for uranium mineralization. We also corrected non-standard expressions such as "gravel braided river" and "Uranium-bearing sandbody" in the paper.

ii. Grammatical errors (examples):

• Line 88: "the properties of the ore-forming fluids have been determined" - overstated; properties remain uncertain.

• Line 314: "indicating that the type of the sandstone is greywacke" - awkward construction.

• Line 474: "The dispersed adsorbed uranium is actually the nano-scale uranium minerals" - "is actually" undermines precision.

iii. Redundancy:

• Extensive description of sedimentary structures (lines 196-204, 318-333, 344-361) could be condensed.

• Repeated statements about gray/grayish-white alteration zones (lines 578-583, 632-635, 687-690).

**Response:** Thank you for your constructive suggestion. Thank you for your constructive suggestions. We have checked the manuscript for grammatical errors. Specifically, we have corrected exaggerated statements, non-standard sentence structures, and redundant expressions, which have greatly improved the readability of the manuscript.

No content in this article has been published elsewhere and is not under consideration for publication. We again thank the reviewers for their valuable comments, suggestions, and recommendations that helped us improve our work and hope that the revised manuscript will meet the requirements for publication in PLOS ONE. Please forward all correspondence concerning this manuscript to me at [destinw@126.com](mailto:destinw@126.com).

Sincerely yours,

Qing Wang.
