# Supplementary material for: Depositional architecture and post-depositional alteration of the Toutunhe Formation (J2t) in the Louzhuangzi area, Southern Junggar Basin: Implications for uranium mineralization
Source: PLoS One. 2026 Jun 16;21(6):e0351337. doi: 10.1371/journal.pone.0351337 (PMC13271488; doi:10.1371/journal.pone.0351337)
Supplement: Attachment — Submitted filename: Response_to_Reviewers_auresp_3.docx [file pone.0351337.s010.docx]

*Editor-in-Chief,*

*PLOS ONE,*

*Dear Editor,*

Thank you for your constructive comments that were very helpful in improving the quality of this manuscript. We have carefully reviewed the comments and made corresponding revisions to the manuscript. We have submitted the second revised manuscript to PLOS ONE, entitled " **Control of deposition-epigenetic alteration of sandbody on uranium mineralization in Toutunhe Formation, Louzhuangzi area, Southern Margin of Junggar Basin, China** ".

We have comprehensively revised the paper based on the reviewers' comments. The main revisions are marked in red in the manuscript. The most important revisions are as follows:

# Reviewer #2:

**General Comments**

I have carefully evaluated this manuscript according to the PLOS ONE publication criteria, which emphasize scientific rigor and methodological soundness. Overall, the study is technically solid, and the data support the conclusions. The manuscript would benefit from minor revisions, primarily regarding clarity, consistency, and presentation.

**Response:** Thank you for your constructive suggestions. We are grateful for your positive assessment that the study is technically sound and that the data adequately support the conclusions. In response to your suggestions, we have carefully revised the manuscript to improve clarity, consistency, and overall presentation. Specifically, we have refined the language for greater precision and readability, ensured consistency in terminology throughout the text, and improved the organization and formatting of figures and descriptions. We believe these revisions have strengthened the manuscript and enhanced its overall quality. We sincerely appreciate your helpful comments, which have contributed to improving our work.

**Specific Comments.**

1. Abstract

In the Abstract:

The objective of the study should be stated more clearly in the first 2–3 sentences.

Some results are described qualitatively; consider adding key quantitative values (e.g., mineral percentages, geochemical ranges).

The final sentence should better emphasize the main conclusion or implication.

**Response:** Thank you for your constructive suggestions. In response, we have thoroughly revised the Abstract to improve its clarity, structure, and scientific rigor. Specifically, (1) the research objective has been clearly stated within the first two sentences to better define the purpose of the study; (2) key quantitative results, including mineral proportions (e.g., coffinite and pitchblende ~68%, Ti-bearing uranium phases ~16%, and nanoscale uranium minerals ~16%) and geochemical ranges (e.g., UO₂ contents), have been incorporated to replace previously qualitative descriptions; and (3) the final sentence has been rewritten to more explicitly highlight the main conclusion and its broader geological implications. These revisions ensure that the Abstract now follows a clear structure (background–objective–methods–results–implications) and better aligns with the journal’s expectations. We appreciate your suggestions, which have significantly improved the quality of this section.

2. Introduction

The Introduction provides sufficient background, but:

The research gap is not clearly highlighted. Please explicitly state what is missing in previous studies.

The objective(s) of the study should be clearly listed at the end of the section.

**Response:** Thank you for your constructive suggestions. We have carefully revised the introduction to clearly identify research gaps and articulate the objectives of this study.

First, we have added a dedicated paragraph that systematically outlines the limitations of previous research, thus reinforcing the understanding of these gaps.

Specifically, we now emphasize:(1) The microscopic features of sedimentary structures and post-depositional alteration remain insufficiently revealed; (2) The nature and interactions of ore-forming fluids (oxidizing and reducing fluids) remain controversial; and (3) Previous studies have lacked integration of mineralogical evidence at the micrometer to nanometer scale. These additions clearly highlight the shortcomings of previous research and underscore the necessity of this study.

Second, we have clearly articulated the objectives of this study in a structured manner at the end of the introduction. We have added a new paragraph outlining four specific objectives: (i) Analysis of sedimentary tectonic features; (ii) Identification of mineralogical features using multi-scale analysis methods (SEM, electron probe microscopy, focused ion beam-transmission electron microscopy); (iii) Constraining the evolution of ore-forming fluids; and (iv) Constructing a sedimentary-fluid coupled genetic model.

3. Geological Setting

The section is informative, but:

Some lithological descriptions are repetitive- these could be streamlined.

Ensure consistency in terminology (e.g., formation names, stratigraphic units).

**Response:** Thank you for your constructive suggestions. We have carefully revised the Geological Setting section to improve clarity, conciseness, and consistency. Specifically, repetitive lithological descriptions have been streamlined and consolidated to avoid redundancy while preserving essential geological information. In addition, we have standardized the terminology throughout the section, ensuring consistent use of formation names and stratigraphic units.

4. Materials and Methods

This section is generally well prepared and meets PLOS ONE standards.

However:

Please specify sample selection criteria more clearly (why these samples were chosen).

Analytical conditions (e.g., SEM, EPMA settings) are described, but consider grouping them in a more structured format for readability.

If applicable, include uncertainty/error margins for measurements.

**Response:** Thank you for your constructive suggestions. We have revised the Materials and Methods section accordingly:

Sample selection criteria: These have now been clearly defined, including the focus on mineralized grayish-white altered sandstones, selection of fresh and unweathered core intervals, and coverage of both vertical and lateral variability to ensure representativeness.

Analytical conditions: The analytical methods have been reorganized into structured subsections (e.g., SEM, EPMA, TEM), with instrument settings and analytical purposes systematically reported to improve readability.

Analytical uncertainties: Relevant information on analytical conditions, calibration standards, and data quality (e.g., oxide total ranges for EPMA) has been added to better constrain measurement uncertainties.

5. Results

The results are comprehensive and well presented.

Suggestions:

Separate observations from interpretations more clearly (some interpretations appear within the Results section).

Figures and tables: Enhance figures, tables, and their captions

Consider summarizing key findings in a short subsection or table.

**Response:** Thank you for your constructive suggestions. We have revised the manuscript accordingly: Separation of observations and interpretations: The Results section has been carefully revised to retain only observational data, while interpretative content has been moved to the Discussion section where appropriate. Figures and tables: All figures and tables have been improved for clarity and readability, and their captions have been expanded to provide more complete and self-explanatory descriptions. Summary of key findings: A concise summary of the main results has been added to highlight the key findings and improve the overall readability of the Results section.

6. Discussion

The Discussion is scientifically sound and well-reasoned.

Improvements:

Some arguments could be strengthened by comparing results with similar studies.

Avoid repetition of results; focus more on interpretation and implications.

The genetic model is interesting, but it would benefit from:

A clearer step-by-step explanation

Possibly a conceptual diagram

**Response:** Thank you for your constructive suggestions. We have revised the Discussion section accordingly: Comparison with previous studies: Additional comparisons with relevant studies on sandstone-hosted uranium deposits and related systems have been incorporated to strengthen the arguments; Reduction of repetition: Repetitive descriptions of results have been removed, with greater emphasis placed on interpretation and geological implications; Genetic model improvement: The genetic model has been clarified with a more explicit step-by-step explanation, and a conceptual diagram has been added to better illustrate the proposed processes.

7. Conclusions

The Conclusions are appropriate but could be improved:

Avoid repeating detailed results.

Focus on key findings and their implications.

Consider adding 1–2 sentences on future research directions.

**Response:** Thank you for your constructive suggestions. We have revised the Conclusions section accordingly:Reduced repetition of detailed results: The Conclusions have been streamlined to avoid reiterating descriptive results, with greater emphasis placed on synthesizing key findings; Strengthened implications: We now highlight the broader geological significance, including sedimentary control, multi-fluid interactions, and nanoscale mineralization processes in sandstone-hosted uranium systems; Added future research directions: One to two sentences have been included to outline future work, particularly focusing on fluid evolution, quantitative constraints, and the formation mechanisms of Ti–U nanophases.

8. Language and Presentation

The manuscript is understandable, but several minor issues should be addressed:

Grammatical inconsistencies (e.g., verb tense, article usage)

Occasional repetition of phrases

A professional language edit is recommended.

**Response:** Thank you for your constructive suggestions. We have carefully revised the manuscript to improve language quality and presentation: Grammatical issues: Verb tense, article usage, and sentence structure have been systematically corrected throughout the manuscript; Repetition: Redundant phrases and repetitive expressions have been removed or refined to enhance clarity and conciseness; Language editing: The manuscript has undergone thorough language polishing to ensure clarity, consistency, and readability.

# Reviewer #5:

General Evaluation

This revised manuscript is much readable. The topic is relevant and potentially valuable, particularly given the combination of field observations and micro-analytical techniques (SEM, EPMA, FIB-TEM). However, despite the promising dataset, the manuscript requires substantial revision. The principal shortcomings relate to lack of methodological clarity, weak sedimentological rigor, insufficient linkage between data and interpretation, and structural inconsistencies throughout the manuscript. In its current form, the study does not yet meet the standards required for publication. I have some comments on the annotated PDF manuscript, which are amplified here. Authors are advised to consult both materials when revising their manuscript.

**Response:** Thank you for your constructive suggestions. We appreciate your recognition that the manuscript has improved in readability and that the dataset, integrating field observations with micro-analytical techniques (SEM, EPMA, and FIB–TEM), is valuable.

We also acknowledge your concerns regarding methodological clarity, sedimentological rigor, the linkage between data and interpretation, and structural consistency. In response, we have undertaken a comprehensive revision of the manuscript. Specifically, we have: (1) substantially clarified the Methods section by providing detailed descriptions of analytical procedures, instrumentation, standards, and uncertainties; (2) strengthened the sedimentological analysis by refining facies interpretations and improving the description of depositional architecture; (3) enhanced the integration between observational data and genetic interpretations to ensure that all conclusions are directly supported by the presented evidence; and (4) reorganized the manuscript to improve overall structure, logical flow, and consistency across sections.

In addition, we have carefully addressed all comments provided in the annotated PDF, and corresponding revisions have been incorporated throughout the manuscript. We believe these improvements have significantly strengthened the scientific rigor and clarity of the study. We sincerely thank you for your detailed and valuable feedback, which has greatly contributed to enhancing the quality of our work

**Major Comments**

1. Abstract and Statement of Purpose

The abstract is overly long, not well structured and does not clearly articulate the research objective.

Lines 18–22: The abstract emphasizes methodology prematurely rather than clearly stating the aim of the study. As noted in the manuscript, “this sentence needs re-framing… the latter part should establish the purpose of the study rather than highlighting methodology.”

The abstract length should also be verified against journal requirements.

Recommendation: Reorganize the abstract to follow a clear structure: background → objective → methods → key findings → implications.

**Response:** Thank you for your constructive suggestions. In response, we have comprehensively revised the Abstract to improve its structure, clarity, and alignment with journal requirements. Specifically, (1) the Abstract has been reorganized to follow a clear and logical structure of background → objective → methods → key findings → implications; (2) the research objective is now explicitly stated in the opening sentences to clearly articulate the purpose of the study; (3) the previously noted sentences (Lines 18–22) have been rephrased to avoid prematurely emphasizing methodology, with the focus shifted toward establishing the study aim; and (4) the overall length of the Abstract has been reduced to comply with the journal’s word limit. These revisions ensure that the Abstract more effectively communicates the scientific objectives, key results, and broader implications of the study. We appreciate your constructive suggestions, which have significantly improved the presentation and readability of this section.

2. Inadequate Literature Context

The introduction lacks sufficient engagement with key literature.

Line 66: The manuscript discusses uranium mineralization controls but does not cite landmark studies (“Please name some landmark papers relating to this here”).

Lines 74–75 and 88–90: Statements are made without citations (“source??”).

Recommendation: Strengthen the literature review by incorporating foundational and recent studies on:

Sandstone-hosted uranium systems

Sedimentary controls on mineralization

Regional geology of the Junggar Basin

**Response:** Thank you for your constructive suggestions. We have thoroughly revised the Introduction to strengthen the literature context by incorporating both foundational and recent studies, and by adding appropriate citations to all previously unsupported statements.

3. Geological Background – Conceptual and Factual Issues

The geological background section is problematic.

Lines 150–154: The tectonic description is misleading and oversimplified, as highlighted. How can the Jungar Basin be located on Kazakhstan?

Line 154: Timing of tectonic evolution is unclear

Line 161: Citation required but missing.

Recommendation: Reconstruct this section using established regional geological frameworks with proper chronological and tectonic context, supported by appropriate references.

**Response:** Thank you for your constructive suggestions. We acknowledge that the previous version contained oversimplified and potentially misleading descriptions of the regional tectonic framework.

(1) Tectonic framework clarification (Lines 150–154):

We have revised the description to avoid ambiguity regarding the tectonic position of the Junggar Basin. The updated text now clearly states that the basin is located within the eastern segment of the Kazakhstan Plate and is bounded by the Siberian Plate to the north and the Tarim Plate to the south, rather than being “located on Kazakhstan.” This revision aligns with widely accepted regional tectonic frameworks (e.g., Cheng , 2001; Windley et al., 2007; Hendrix et al., 1992; Carroll et al., 1995; Jolivet et al., 2010).

(2) Timing of tectonic evolution (Line 154):

We have substantially revised this part to present a clearer chronological framework. The tectonic evolution is now explicitly divided into key stages, including:

(i) Late Paleozoic ocean closure and terrane accretion,

(ii) Early Mesozoic reactivation of Paleozoic structural frameworks, and

(iii) Cenozoic intracontinental deformation related to the India–Eurasia collision, which led to the uplift of the Tianshan Mountains and development of the foreland fold–thrust system.

This restructuring improves both temporal clarity and geological accuracy.

(3) Missing citation (Line 161):

The missing reference has now been added, and all statements in this section are supported by appropriate and up-to-date literature. We have also reviewed the entire Geological Background section to ensure that all key interpretations are properly referenced.

(4) Overall revision of Geological Background:

Following the reviewer’s recommendation, we have comprehensively reconstructed this section using established regional geological models. The revised version emphasizes: a clear tectonic framework; a coherent chronological evolution, and stronger linkage between basin evolution, sedimentation, and uranium mineralization.

These revisions significantly improve the scientific rigor and reliability of the Geological Background section.

4. Methodology – Major Deficiencies

The Methods section lacks essential detail and reproducibility.

Key omissions:

Lines 199–206: No clarity on whether cores were collected by the authors or sourced from a company

Missing details on number of wells, sampling intervals, and total samples

Lines 221–224: No indication of number of thin sections prepared

Lines 225–250: Number of samples analyzed via SEM/EPMA/TEM not specified

Lines 216–220:Sampling strategy is described qualitatively but not quantitatively

Recommendation: Provide:

Total number of samples (field, thin section, SEM, EPMA, TEM)

Source of core materials

Sampling rationale and representativeness

Analytical uncertainties

**Response:** Thank you for your constructive suggestions. We have substantially revised the Materials and Methods section to improve clarity, detail, and reproducibility.

Specifically, we have: Clarified the source of core materials, stating that all samples were obtained from exploration drill holes provided by the CNNC No. 216 Geological Party; Provided quantitative sampling information, including the number of drill holes, sampling depth range, and total number of samples collected; Specified the number of samples used in each analytical method, including thin sections, SEM, EPMA, and TEM analyses; Refined the sampling strategy, describing it in a quantitative framework that integrates both lateral (west–central–east sectors) and vertical (24.84–871.50 m) coverage to ensure representativeness; Added analytical details and uncertainty-related information, including instrument conditions and calibration standards.These revisions significantly enhance the transparency, reproducibility, and methodological rigor of the study.

5. Sedimentary Facies Analysis – Lack of Rigor

This is one of the most critical weaknesses of the manuscript.

Lines 373–382: Facies interpretations (e.g., floodplain, levee, channel-fill) are presented without supporting evidence

Lines 386–398: No clear criteria used for facies classification

Lines 408–413: The Results section improperly includes references and interpretations

Recommendation: Rebuild the sedimentological analysis using a standard workflow:

1.Observations (grain size, structures, geometry)

2.Interpretation (process-based)

3.Facies classification (with explicit criteria)

**Response:** Thank you for your constructive suggestions. We agree that the original sedimentological analysis lacked sufficient rigor and clarity.

Observations: We now present detailed, evidence-based descriptions of grain size, sedimentary structures (e.g., cross-bedding, parallel bedding), and sand body geometry derived from core and thin-section observations. Interpretation: Process-based interpretations (e.g., channel flow, overbank deposition) are explicitly linked to the observed sedimentary features. Facies classification: Facies types (e.g., channel-fill, levee, floodplain) are redefined using clear and widely accepted diagnostic criteria, which are now explicitly stated in the text. In addition, all unsupported interpretations have been either removed or justified with direct observational evidence, and inappropriate references and interpretative statements previously included in the Results section have been relocated to the Discussion section.

6. Mixing of Methods and Results

Lines 289–303: Petrographic quantification (QFL analysis, compositional maturity) appears in Results but lacks methodological explanation

Recommendation: Move all methodological descriptions (e.g., modal analysis procedures) into the Methods section.

**Response:** Thank you for your constructive suggestions. We agree that the methodological description of petrographic quantification was insufficiently presented.

In the revised manuscript, all procedures related to modal analysis (including QFL point-counting methods and compositional maturity calculations) have been clearly described in the Methods section. The Results section has been revised accordingly to present only the outcomes of the analysis, without methodological details. These changes improve the logical structure and ensure a clear separation between Methods and Results, in line with journal standards.

7. Results Section – Lack of Clarity and Structure

The Results section is largely descriptive and lacks analytical depth.

Lines 253–261: Contains vague and unsupported statements

Line 263: Content flagged as inappropriate placement

Recommendation: Please ensure that:

Results present only observations

Interpretations citations of papers are deferred to the Discussion

All statements are supported by figures, tables, or data

**Response:** Thank you for your constructive suggestions. We agree that the original Results section lacked clarity and structural rigor. In the revised manuscript, the Results section has been thoroughly reorganized to ensure that it presents only direct observations, with all interpretative statements and literature citations moved to the Discussion section. Vague or unsupported statements have been either removed or rewritten based on explicit petrographic and analytical evidence, and the content previously flagged has been relocated to a more appropriate section. In addition, all descriptions in the Results are now explicitly supported by corresponding figures, tables, or analytical data. These revisions significantly improve the clarity, logical structure, and scientific robustness of the Results section.

8. Poor Link Between Data and Uranium Mineralization Model

The manuscript proposes a conceptual model but does not sufficiently demonstrate:

How sedimentary architecture controls permeability

How diagenesis influences uranium precipitation

How micro-scale observations relate to basin-scale processes

Recommendation: Strengthen integration between:

Facies architecture

Fluid flow pathways

Redox conditions

Mineralization processes

**Response:** Thank you for your constructive suggestions. We agree that the linkage between data and the uranium mineralization model required further strengthening.

In the revised manuscript, we have improved the integration between multi-scale observations and the genetic model by: (1) explicitly relating sedimentary facies architecture to permeability heterogeneity and fluid flow pathways; (2) clarifying the role of diagenetic processes (e.g., pyrite formation, clay alteration, and organic matter evolution) in establishing redox conditions favorable for uranium precipitation; and (3) linking micro-scale evidence (e.g., Ti–U associations, pyrite textures, and nanoparticle occurrences) to basin-scale fluid evolution and mineralization processes. These revisions establish a clearer and more coherent connection between facies architecture, fluid migration, redox interfaces, and uranium mineralization, thereby significantly strengthening the conceptual model.

9. Overuse of Unsupported Interpretations

Throughout the manuscript, interpretations are made without direct evidence.

Examples include:

Lines 373–399: Facies assignments without diagnostic criteria

Lines 408–426: Mineralization controls described without clear data linkage

Recommendation: All interpretations must be explicitly supported by:

Observational data

Figures or tables

Established sedimentological principles

**Response:** Thank you for your constructive suggestions. We agree that several interpretations in the original manuscript were insufficiently supported by direct evidence. In the revised manuscript, all interpretative statements have been carefully re-evaluated. Unsupported or speculative interpretations have been either removed or revised to ensure they are explicitly grounded in observational data. Facies assignments are now based on clearly defined diagnostic criteria, and interpretations of mineralization controls are directly linked to petrographic evidence. In addition, all key interpretations are now supported by corresponding figures, tables, or established sedimentological principles. These revisions significantly improve the scientific rigor and reliability of the manuscript.

10. Figures and Supporting Evidence

Figures are not effectively used to support interpretations

Lack of explicit cross-referencing between text and figures

Recommendation:

Improve figure captions (interpretive, not descriptive)

Explicitly link interpretations to figures

**Response:** Thank you for your constructive suggestions. We agree that the linkage between figures and interpretations needs improvement. In the revised manuscript, all figures have been carefully re-evaluated and optimized to support the scientific interpretations better. Figure captions have been rewritten to include key interpretive information rather than purely descriptive content. In addition, explicit cross-referencing between the text and figures has been strengthened throughout the manuscript, ensuring that all major interpretations are directly supported by corresponding visual evidence.

Minor Comments

1. Language and Grammar

Numerous grammatical and stylistic issues throughout

Example: Line 389 (“The ore-bearing main sandstone…”) requires rephrasing

2. Terminology

Inconsistent use of sedimentological and mineralogical terminology

3. Referencing

Multiple missing citations (e.g., lines 74–75, 161)

4. Organization

Some content appears misplaced or redundant (e.g., petrographic data in Results without methodological context)

**Response:** Thank you for these helpful comments.

(1) Language and Grammar: The manuscript has been thoroughly revised for grammar and style, and problematic sentences (e.g., Line 389) have been rephrased. A professional language edit has also been applied.

(2) Terminology: Sedimentological and mineralogical terminology has been standardized throughout the manuscript to ensure consistency.

(3) Referencing: Missing citations (e.g., Lines 74–75, 161) have been added, and the reference list has been carefully checked and updated.

(4) Organization: The manuscript structure has been improved by relocating misplaced or redundant content (e.g., petrographic data and methodological descriptions) to appropriate sections, ensuring a clear separation between Methods, Results, and Discussion.

These revisions improve the overall clarity, consistency, and organization of the manuscript.

Additional Observations

The manuscript lacks a clear conceptual framework linking sedimentology, diagenesis, and mineralization

Temporal relationships between:

Deposition

Diagenesis. I feel this control on Uranium mineralization is poorly crafted in the study. I made references to some of my works and the need for authors to discuss paragenetic sequence as it relates to the focus of the study.

Mineralization remain unclear

Microanalytical data (SEM/TEM) are not sufficiently integrated with field-scale observations

Recommendation:

Major Revision

The manuscript requires substantial restructuring and methodological clarification before it can be considered for publication. The dataset is valuable, but significant improvements are necessary in:

Sedimentological rigor

Methodological transparency

Evidence-based interpretation

Overall organization

**Response:** Thank you for your constructive suggestions. Thank you for your comprehensive and insightful review. We fully understand the questions you raised and have made significant revisions to the manuscript accordingly.

In the discussion, we established a clearer conceptual framework that explicitly links sedimentology, diagenesis, and uranium mineralization to clarify the temporal relationships between sedimentary, diagenetic, and mineralization processes, and how these factors collectively influence uranium precipitation.

Furthermore, we have more systematically integrated micro-area analysis data (SEM, EPMA, FIB-TEM) with field-scale and petrographic observations to enhance multi-scale interpretation.

Simultaneously, we have significantly restructured the manuscript to improve sedimentological rigor, enhance methodological transparency, and ensure that all interpretations are supported by clear observational evidence.

No content in this article has been published elsewhere and is not under consideration for publication. We again thank the reviewers for their valuable comments, suggestions, and recommendations that helped us improve our work and hope that the revised manuscript will meet the requirements for publication in PLOS ONE. Please forward all correspondence concerning this manuscript to me at [destinw@126.com](mailto:destinw@126.com).

Sincerely yours,

Qing Wang.
