# Supplementary material for: Depositional architecture and post-depositional alteration of the Toutunhe Formation (J2t) in the Louzhuangzi area, Southern Junggar Basin: Implications for uranium mineralization
Source: PLoS One. 2026 Jun 16;21(6):e0351337. doi: 10.1371/journal.pone.0351337 (PMC13271488; doi:10.1371/journal.pone.0351337)
Supplement: Attachment — Submitted filename: Commets to the Author.docx [file pone.0351337.s011.docx]

The revised manuscript shows clear and substantial improvement, with better organization, clearer methods, and stronger scientific coherence. Key sections such as the Abstract, Introduction, Geological Background, and Methods are now more clearly written and better structured. The addition of quantitative data, improved sampling description, and integrated analytical techniques (petrography, SEM, EPMA, and FIB–TEM) significantly strengthens the study and enhances its contribution to understanding sandstone-hosted uranium mineralization in the southern Junggar Basin.

However, several important issues still require further attention before the manuscript can be considered fully suitable for publication:

1. The sedimentological interpretation has improved, but the overall sedimentological rigor remains moderate. Several depositional facies interpretations would benefit from clearer diagnostic criteria and stronger support from sedimentary structures, grain-size characteristics, and architectural relationships. A more process-based facies analysis would strengthen this section considerably.
2. Some interpretative statements are still included within the Results section. Separating observational data more clearly from interpretation/discussion would improve both the scientific clarity and the structure of the manuscript.
3. Although the Discussion section is improved, some interpretations are still not sufficiently supported by the presented petrographic, mineralogical, and sedimentological data. The discussion would benefit from stronger and more explicit connections between the analytical results and the proposed geological interpretations.
4. The proposed genetic model is interesting and has improved significantly; however, the relationships among sediment deposition, diagenesis, oxidation, hydrothermal alteration, fluid evolution, and uranium precipitation are still not fully constrained.
5. The integration between micro-scale mineralogical observations and basin-scale geological processes remains somewhat inferential. Additional discussion linking permeability architecture, fluid migration pathways, redox evolution, alteration processes, and uranium mineralization would improve the robustness of the proposed model.
6. The language quality has improved considerably, but the manuscript still contains occasional grammatical inconsistencies, awkward sentence construction, repetitive wording, and several overly long sentences. A final professional English language edit is recommended before acceptance.

Overall, with additional revisions focused on strengthening the evidence-based discussion, improving sedimentological interpretation, clarifying the linkage between results and interpretations/discussion, and refining the language, the manuscript could become suitable for publication in PLOS ONE.Comments
