# Supplementary material for: Depositional architecture and post-depositional alteration of the Toutunhe Formation (J2t) in the Louzhuangzi area, Southern Junggar Basin: Implications for uranium mineralization
Source: PLoS One. 2026 Jun 16;21(6):e0351337. doi: 10.1371/journal.pone.0351337 (PMC13271488; doi:10.1371/journal.pone.0351337)
Supplement: Peer review history

# Peer review: Decision Letter 0

**DOI:** 10.1371/journal.pone.0351337.r001

**Authors:** Mohamed Y.M. Hanfi

31 Jul 2025

PONE-D-25-34881

Control of deposition-epigenetic alteration of sandbody on uranium mineralization in Toutunhe Formation, Louzhuangzi area, Southern Margin of Junggar Basin, China

PLOS ONE

Dear Dr. Wang,

Thank you for submitting your manuscript to PLOS ONE. After careful consideration, we feel that it has merit but does not fully meet PLOS ONE’s publication criteria as it currently stands. Therefore, we invite you to submit a revised version of the manuscript that addresses the points raised during the review process.

Please submit your revised manuscript by Sep 14 2025 11:59PM. If you will need more time than this to complete your revisions, please reply to this message or contact the journal office at plosone@plos.org. When you're ready to submit your revision, log on to https://www.editorialmanager.com/pone/ and select the 'Submissions Needing Revision' folder to locate your manuscript file.

Please include the following items when submitting your revised manuscript:

- A rebuttal letter that responds to each point raised by the academic editor and reviewer(s). You should upload this letter as a separate file labeled 'Response to Reviewers'.
- A marked-up copy of your manuscript that highlights changes made to the original version. You should upload this as a separate file labeled 'Revised Manuscript with Track Changes'.
- An unmarked version of your revised paper without tracked changes. You should upload this as a separate file labeled 'Manuscript'.

If you would like to make changes to your financial disclosure, please include your updated statement in your cover letter. Guidelines for resubmitting your figure files are available below the reviewer comments at the end of this letter.

If applicable, we recommend that you deposit your laboratory protocols in protocols.io to enhance the reproducibility of your results. Protocols.io assigns your protocol its own identifier (DOI) so that it can be cited independently in the future. For instructions see: https://journals.plos.org/plosone/s/submission-guidelines#loc-laboratory-protocols. Additionally, PLOS ONE offers an option for publishing peer-reviewed Lab Protocol articles, which describe protocols hosted on protocols.io. Read more information on sharing protocols at https://plos.org/protocols?utm_medium=editorial-email&utm_source=authorletters&utm_campaign=protocols.

We look forward to receiving your revised manuscript.

Kind regards,

Mohamed Y.M. Hanfi

Academic Editor

PLOS ONE

Journal requirements:

When submitting your revision, we need you to address these additional requirements.

1. Please ensure that your manuscript meets PLOS ONE's style requirements, including those for file naming. The PLOS ONE style templates can be found at

https://journals.plos.org/plosone/s/file?id=wjVg/PLOSOne_formatting_sample_main_body.pdf and

https://journals.plos.org/plosone/s/file?id=ba62/PLOSOne_formatting_sample_title_authors_affiliations.pdf

2. In your Methods section, please provide additional information regarding the permits you obtained for the work. Please ensure you have included the full name of the authority that approved the field site access and, if no permits were required, a brief statement explaining why.

3. Thank you for stating the following financial disclosure:

[National Natural Science Foundation of China (No. U2244205 and No. U2067202) and the Joint Innovation Fund Project of China Uranium Industry Company Limited - State Key Laboratory of Nuclear Resources and Environment, East China University of Technology (2023NRE-LH-10).].

Please state what role the funders took in the study.  If the funders had no role, please state: "The funders had no role in study design, data collection and analysis, decision to publish, or preparation of the manuscript."

If this statement is not correct you must amend it as needed.

Please include this amended Role of Funder statement in your cover letter; we will change the online submission form on your behalf.

4.If the reviewer comments include a recommendation to cite specific previously published works, please review and evaluate these publications to determine whether they are relevant and should be cited. There is no requirement to cite these works unless the editor has indicated otherwise.

Additional Editor Comments (if provided):

[Note: HTML markup is below. Please do not edit.]

Reviewers' comments:

Reviewer's Responses to Questions

**Comments to the Author**

1. Is the manuscript technically sound, and do the data support the conclusions?

The manuscript must describe a technically sound piece of scientific research with data that supports the conclusions. Experiments must have been conducted rigorously, with appropriate controls, replication, and sample sizes. The conclusions must be drawn appropriately based on the data presented.

Reviewer #1: Yes

Reviewer #2: Yes

**********

2. Has the statistical analysis been performed appropriately and rigorously?

Reviewer #1: Yes

Reviewer #2: No

**********

3. Have the authors made all data underlying the findings in their manuscript fully available?

The PLOS Data policy requires authors to make all data underlying the findings described in their manuscript fully available without restriction, with rare exception (please refer to the Data Availability Statement in the manuscript PDF file). The data should be provided as part of the manuscript or its supporting information, or deposited to a public repository. For example, in addition to summary statistics, the data points behind means, medians and variance measures should be available. If there are restrictions on publicly sharing data—e.g. participant privacy or use of data from a third party—those must be specified.

Reviewer #1: Yes

Reviewer #2: Yes

**********

4. Is the manuscript presented in an intelligible fashion and written in standard English?

PLOS ONE does not copyedit accepted manuscripts, so the language in submitted articles must be clear, correct, and unambiguous. Any typographical or grammatical errors should be corrected at revision, so please note any specific errors here.

Reviewer #1: Yes

Reviewer #2: Yes

**********

5. Review Comments to the Author

Please use the space provided to explain your answers to the questions above. You may also include additional comments for the author, including concerns about dual publication, research ethics, or publication ethics. (Please upload your review as an attachment if it exceeds 20,000 characters)

Reviewer #1: This manuscript presents a well-executed study on uranium mineralization in the Toutunhe Formation, integrating analysis of depositional environments with epigenetic alteration processes. The research is methodologically robust, employing field surveys, petrographic studies, and advanced microscopy techniques to elucidate the controls on uranium mineralization. The findings are novel and make a meaningful contribution to the understanding of sandstone-type uranium deposits.

The study effectively addresses key questions about the depositional and epigenetic factors influencing uranium mineralization.

The use of SEM, EMPA, and FIB-TEM provides detailed insights into uranium occurrence and mineral associations, where the identification of brannerite and the proposed role of hydrocarbon reduction are notable advantages.

The manuscript is well-organized, with logical flow and clear presentation of results.

Some Suggestions for Improvement:

Thermal Fluids: Further discussion on the origin and properties of thermal fluids would enhance the understanding of their role in mineralization.

Hydrocarbon Evidence: Additional data, such as fluid inclusions with more analysis, could strengthen the hydrocarbon reduction hypothesis.

Some bulk sample analyses are needed to give an overview about the nature of the samples and their matrix.

Reviewer #2: General Evaluation

The manuscript offers a detailed geological, sedimentological, and mineralogical study of uranium mineralization in the Toutunhe Formation, Junggar Basin, using fieldwork and microanalytical methods. Although it has scientific value and potential to advance knowledge on sandstone-type uranium deposits, significant revisions are needed before it is suitable for publication in PLOS One.

Major Points for Revision

1. The introduction should clearly state the research objectives and hypotheses to guide the reader.

2. The manuscript lacks a clear, standalone methodology section, making it hard to separate methods from background and results. A dedicated “Materials and Methods” section with labeled subsections is recommended for clarity.

3. The manuscript lacks detailed geochemical analysis, such as elemental concentrations, ratios, or diagrams (e.g., Harker or discrimination plots) that could support the interpretation of provenance, alteration, and uranium enrichment processes.

4. The study heavily relies on SEM, EMPA, and FIB-TEM observations. However, no XRD or quantitative mineral abundance data are provided. Including XRD would validate the qualitative and semi-quantitative identification of mineralogical phases within crystalline materials.

5. The Results section in the manuscript is informative but not clearly delineated as a standalone section. The findings (e.g., petrography, depositional environment, uranium mineralization) are integrated within broader descriptive sections, making it hard to distinguish between background, methodology, and results.

5.1 The text mentions the presence of pyrite and organic debris but does not sufficiently explore their role in uranium precipitation. A more focused discussion on their redox buffering capacity is recommended.

5.2 The manuscript provides Q/ (F+R) ratios to indicate compositional maturity, but the significance of these values in the context of uranium mobility and deposition should be elaborated.

5.3 In several parts of the manuscript, it is unclear whether certain alterations (e.g., chloritization, sericitization) are of early diagenetic or late-stage epigenetic origin.

6. The manuscript lacks a clearly explained discussion section. To improve scientific rigor, the authors should provide a separate and well-structured discussion that interprets key findings, compares them with previous studies, and explains their implications for uranium mineralization processes.

7. Summarize key findings and explicitly discuss how the study informs future uranium exploration efforts in the Junggar Basin and comparable basins.

8. The manuscript uses author–year (Harvard-style) citations format, but PLOS ONE requires numbered references in Vancouver style. Please revise all in-text citations and the reference list to match the required format.

Recommendation: Major Revision

**********

6. PLOS authors have the option to publish the peer review history of their article (what does this mean?). If published, this will include your full peer review and any attached files.

If you choose “no”, your identity will remain anonymous but your review may still be made public.

**Do you want your identity to be public for this peer review?**   For information about this choice, including consent withdrawal, please see our Privacy Policy.

Reviewer #1: **Yes:**   WAEL ABDELWAHAB IBRAHEM

Reviewer #2: **Yes:**   Hussien Yessuf Ahmed

**********

[NOTE: If reviewer comments were submitted as an attachment file, they will be attached to this email and accessible via the submission site. Please log into your account, locate the manuscript record, and check for the action link "View Attachments". If this link does not appear, there are no attachment files.]

While revising your submission, please upload your figure files to the Preflight Analysis and Conversion Engine (PACE) digital diagnostic tool, https://pacev2.apexcovantage.com/. PACE helps ensure that figures meet PLOS requirements. To use PACE, you must first register as a user. Registration is free. Then, login and navigate to the UPLOAD tab, where you will find detailed instructions on how to use the tool. If you encounter any issues or have any questions when using PACE, please email PLOS at figures@plos.org. Please note that Supporting Information files do not need this step.

Attachment Submitted filename: Review Comments to the Author.docx

# Author comment: Author response to Decision Letter 1

**DOI:** 10.1371/journal.pone.0351337.r002

8 Oct 2025

Journal requirements:

When submitting your revision, we need you to address these additional requirements.

1. Please ensure that your manuscript meets PLOS ONE's style requirements, including those for file naming. The PLOS ONE style templates can be found at

https://journals.plos.org/plosone/s/file?id=wjVg/PLOSOne_formatting_sample_main_body.pdf and

https://journals.plos.org/plosone/s/file?id=ba62/PLOSOne_formatting_sample_title_authors_affiliations.pdf

Response: We appreciate the editor’s reminder regarding manuscript formatting and file naming requirements. In the revised submission, we have carefully formatted the manuscript in accordance with the PLOS ONE style guidelines, including the use of appropriate section headings, reference style, figure and table placement, and file naming conventions, as outlined in the provided templates:

PLOS Manuscript Body Formatting Guidelines

PLOS Affiliations Formatting Guidelines

All manuscript files have been renamed following PLOS ONE’s file naming instructions. We hope the revised version now fully complies with the journal’s formatting requirements.

2. In your Methods section, please provide additional information regarding the permits you obtained for the work. Please ensure you have included the full name of the authority that approved the field site access and, if no permits were required, a brief statement explaining why.

Response: This study was conducted with the support of a joint innovation project between East China University of Technology and the No. 216 Geological Party, China National Nuclear Corporation (CNNC). Field access was granted by the No. 216 Geological Party. Both institutions jointly carried out the research, and contributors from each party have been appropriately acknowledged in the author information section. Therefore, no additional fieldwork permission document is required.

3. Thank you for stating the following financial disclosure:

[National Natural Science Foundation of China (No. U2244205 and No. U2067202) and the Joint Innovation Fund Project of China Uranium Industry Company Limited - State Key Laboratory of Nuclear Resources and Environment, East China University of Technology (2023NRE-LH-10).].

Please state what role the funders took in the study. If the funders had no role, please state: "The funders had no role in study design, data collection and analysis, decision to publish, or preparation of the manuscript."

If this statement is not correct you must amend it as needed.

Please include this amended Role of Funder statement in your cover letter; we will change the online submission form on your behalf.

Response: Thank you for your guidance regarding the financial disclosure.

In this study, the field geological investigation and sample preparation were financially supported by the National Natural Science Foundation of China (No. U2244205 and No. U2067202). The laboratory analytical work was supported by the Joint Innovation Fund Project of China Uranium Industry Company Limited – State Key Laboratory of Nuclear Resources and Environment, East China University of Technology (2023NRE-LH-10).

This amended Role of Funder statement has been included in the cover letter as requested.

4. If the reviewer comments include a recommendation to cite specific previously published works, please review and evaluate these publications to determine whether they are relevant and should be cited. There is no requirement to cite these works unless the editor has indicated otherwise.

Response: Thank you for the clarification regarding reviewer-suggested citations.

We have carefully read reviewers’ comments and have not found any reviewers suggesting citing specific published literature. Those that were not directly relevant to the scope or findings of our study have not been cited, in accordance with the editorial guidance that citation is not mandatory unless explicitly required.

We appreciate the opportunity to critically assess and refine our reference list.

Reviewer 1: This manuscript presents a well-executed study on uranium mineralization in the Toutunhe Formation, integrating analysis of depositional environments with epigenetic alteration processes. The research is methodologically robust, employing field surveys, petrographic studies, and advanced microscopy techniques to elucidate the controls on uranium mineralization. The findings are novel and make a meaningful contribution to the understanding of sandstone-type uranium deposits.

The study effectively addresses key questions about the depositional and epigenetic factors influencing uranium mineralization.

The use of SEM, EMPA, and FIB-TEM provides detailed insights into uranium occurrence and mineral associations, where the identification of brannerite and the proposed role of hydrocarbon reduction are notable advantages.

The manuscript is well-organized, with logical flow and clear presentation of results.

Specific modifications：

1. Thermal Fluids: Further discussion on the origin and properties of thermal fluids would enhance the understanding of their role in mineralization.

Response: Thank you for your constructive suggestions.

We have expanded the discussion on the possible origin and properties of the thermal fluids and clarified the evidentiary basis for our interpretations. At present, we have not conducted fluid inclusion analyses; therefore, our conclusions are preliminary and derive from mineral assemblage and alteration features (e.g., chloritization, clay–pyrite–bitumen associations, and Ti–U aggregates/branenerite-like nanoparticles) observed at micro- to nanoscale. These textures are consistent with moderately elevated-temperature, reducing fluids superimposed on earlier oxidation. We now explicitly note this limitation and outline future work (fluid inclusion microthermometry, Raman/GC–MS of inclusions, and stable isotope analyses) that will better constrain fluid sources, temperatures, and timing.

The expanded discussion appears in the revised manuscript at Lines 731-744.

2. Hydrocarbon Evidence: Additional data, such as fluid inclusions with more analysis, could strengthen the hydrocarbon reduction hypothesis.

Some bulk sample analyses are needed to give an overview about the nature of the samples and their matrix.

Response: Thank you for your constructive suggestion. We also agree with the reviewer that providing bulk geochemical or mineralogical characterizations would help clarify the nature of the samples and their matrix. In the revised manuscript, we have further elaborated on the hydrocarbon reduction hypothesis by citing supporting evidence from previously published work (He et al., 2024). Although the current dataset is limited, the available evidence suggests a potential link between migrated hydrocarbons and localized reducing conditions favorable for uranium precipitation. We acknowledge that future studies involving Fluid inclusions, Raman spectroscopy or GC–MS analyses are needed to provide more definitive evidence, and we consider this an important direction for ongoing research.

The relevant revisions can be found in Lines 677-690 of the revised manuscript.

Reviewer 2: The manuscript offers a detailed geological, sedimentological, and mineralogical study of uranium mineralization in the Toutunhe Formation, Junggar Basin, using fieldwork and microanalytical methods. Although it has scientific value and potential to advance knowledge on sandstone-type uranium deposits, significant revisions are needed before it is suitable for publication in PLOS One.

Specific modifications：

1. The introduction should clearly state the research objectives and hypotheses to guide the reader.

Response: Thank you for your constructive suggestions. We fully agree that the study objectives and hypotheses should be clearly stated in the introduction to guide the reader. In the revised introduction (lines 95-105), we clarify the overall study objective: to elucidate the sedimentary-epigenetic controls on uranium mineralization in the Toutunhe Formation (J2t) in the Louzhuangzi area using a comprehensive approach of sedimentology, mineralogy, and microanalysis. We also clarify the working hypothesis: the coupling between sedimentary facies, epigenetic fluid alteration, and hydrocarbon reduction controls the observed uranium mineralization.

2. The manuscript lacks a clear, standalone methodology section, making it hard to separate methods from background and results. A dedicated “Materials and Methods” section with labeled subsections is recommended for clarity.

Response: Thank you for your constructive suggestions. A new “3. Materials and Methods” section has been created with labeled subsections for clarity. These include: Fieldwork and Sampling, Petrography, SEM–EDS, EPMA, FIB–TEM Sample Preparation, and TEM–EDS Analysis. Methods and instrumental settings (e.g., beam current, accelerating voltage, software) have been clearly described. (Lines 152-181).

3. The manuscript lacks detailed geochemical analysis, such as elemental concentrations, ratios, or diagrams (e.g., Harker or discrimination plots) that could support the interpretation of provenance, alteration, and uranium enrichment processes.

Response: Thank you for your constructive suggestion. We agree that detailed geochemical analysis such as element concentration, ratio or graph (e.g., Harker graph or discrimination graph) will provide valuable support for explaining the source, weathering and uranium enrichment processes. However, we note that geologists at our institution have conducted thorough investigations in their work (He, 2025). Since the paper has not been officially published online yet, we avoid citing it to support our current research. Furthermore, we clearly stated in Chapter 5.5 that future work will focus on obtaining main and trace element concentrations (e.g. uranium, titanium, iron, aluminum, thorium, vanadium), element ratios (e.g. titanium/zirconium, thorium/uranium, uranium/ferric), and rare earth element (REE) patterns to depict source signals and hydrothermal superposition. We will also introduce discriminant graphs such as Hakgraph and Thorium/uranium vs. Uranium/iron double-plot to visualize alteration trends and uranium mobility. We appreciate the commenters for their understanding and again for your insights.

4. The study heavily relies on SEM, EMPA, and FIB-TEM observations. However, no XRD or quantitative mineral abundance data are provided. Including XRD would validate the qualitative and semi-quantitative identification of mineralogical phases within crystalline materials.

Response: Thank you for your constructive comment. We acknowledge this limitation. Due to the limited quantity of samples available for destructive analysis, XRD analysis was not conducted in this study. We have explicitly noted this limitation in the Discussion section (Lines 722-729), and suggested that future work should include XRD and QEMSCAN analyses to validate the mineralogical identifications.

5. The Results section in the manuscript is informative but not clearly delineated as a standalone section. The findings (e.g., petrography, depositional environment, uranium mineralization) are integrated within broader descriptive sections, making it hard to distinguish between background, methodology, and results.

Response: Thank you for your constructive suggestion. We have reorganized the manuscript to establish a standalone “6. Conclusions and Implications for Uranium Exploration” section, which now includes five subsections: sedimentary controls on mineralization, uranium mineral assemblages, multi-stage alteration, redox buffering processes and implications for uranium exploration. This restructuring improves clarity and distinguishes the research findings from the background and methodology (Lines 759-783).

5.1 The text mentions the presence of pyrite and organic debris but does not sufficiently explore their role in uranium precipitation. A more focused discussion on their redox buffering capacity is recommended.

Response: Thank you for your constructive suggestions. In the revised manuscript, we have strengthened the discussion of the redox buffering role of pyrite and organic matter in uranium precipitation. Specifically, we discuss their potential to reduce uranium⁶⁺ and promote the precipitation of pitchblende and uraninite. These discussions have been incorporated into the Discussion section (Lines 677-694).

5.2 The manuscript provides Q/ (F+R) ratios to indicate compositional maturity, but the significance of these values in the context of uranium mobility and deposition should be elaborated.

Response: Thank you for your constructive suggestions. In the revised manuscript, we have clarified the significance of the Q/(F+R) ratios. We explain that these values (0.22–1.44) reflect lower compositional maturity and are related to porosity evolution, uranium remobilization potential, and cation exchange buffering during mineralization. See the Discussion (Lines 657-675).

5.3 In several parts of the manuscript, it is unclear whether certain alterations (e.g., chloritization, sericitization) are of early diagenetic or late-stage epigenetic origin.

Response: We thank the reviewer for pointing this out. We revised the relevant paragraphs to specify timing: e.g., chloritization is interpreted as early diagenetic, whereas sericitization and Ti–U phase replacement are epigenetic. These changes are reflected in the Discussion (Lines 696-713).

6. The manuscript lacks a clearly explained discussion section. To improve scientific rigor, the authors should provide a separate and well-structured discussion that interprets key findings, compares them with previous studies, and explains their implications for uranium mineralization processes.

Response: Thank you for your constructive suggestions. We have created a structured "5. Discussion" section with five subheadings: 5.1 Controls on Uranium Mineralization: Sedimentary Facies and Diagenesis, 5.2 Sandstone compositional maturity and its impact on uranium mobility, 5.3 Redox Buffering Role of Pyrite and Organic Matter, 5.4 Timing and Genetic Stage of Mineral Alteration, 5.5 Geochemical Indicators and Mineralogical Constraints, 5.6 Origin and Nature of Thermal Fluids and 5.7 Uranium mineralization model. This has enhanced the depth of our interpretation (Lines 647-755).

7. Summarize key findings and explicitly discuss how the study informs future uranium exploration efforts in the Junggar Basin and comparable basins.

Response: Thank you for your constructive suggestions. We have added a new section, "6. Conclusions and Implications for Uranium Exploration," to the revised manuscript. This section summarizes the main findings and outlines their practical implications for future exploration in the Junggar Basin and similar basins (Lines 778-783).

8. The manuscript uses author–year (Harvard-style) citations format, but PLOS ONE requires numbered references in Vancouver style. Please revise all in-text citations and the reference list to match the required format.

Response: Thank you for pointing out the formatting error in the manuscript. As requested, we have revised all in-text citations and reference list entries to conform to the Vancouver (numbered) format. For example, "(Geng et al., 2020; Wang et al., 2021)" has now been corrected to "[1,6]".

Attachment Submitted filename: Response to Reviewers.docx

# Peer review: Decision Letter 1

**DOI:** 10.1371/journal.pone.0351337.r003

**Authors:** Mohamed Y.M. Hanfi

20 Jan 2026

PONE-D-25-34881R1

Control of deposition-epigenetic alteration of sandbody on uranium mineralization in Toutunhe Formation, Louzhuangzi area, Southern Margin of Junggar Basin, China

PLOS One

Dear Dr. Qing Wang,

Thank you for submitting your manuscript to PLOS ONE. After careful consideration, we feel that it has merit but does not fully meet PLOS ONE’s publication criteria as it currently stands. Therefore, we invite you to submit a revised version of the manuscript that addresses the points raised during the review process.

Please submit your revised manuscript by Mar 06 2026 11:59PM. If you will need more time than this to complete your revisions, please reply to this message or contact the journal office at plosone@plos.org. When you're ready to submit your revision, log on to https://www.editorialmanager.com/pone/ and select the 'Submissions Needing Revision' folder to locate your manuscript file.

Please include the following items when submitting your revised manuscript:

- A letter that responds to each point raised by the academic editor and reviewer(s). You should upload this letter as a separate file labeled 'Response to Reviewers'.
- A marked-up copy of your manuscript that highlights changes made to the original version. You should upload this as a separate file labeled 'Revised Manuscript with Track Changes'.
- An unmarked version of your revised paper without tracked changes. You should upload this as a separate file labeled 'Manuscript'.

If you would like to make changes to your financial disclosure, please include your updated statement in your cover letter. Guidelines for resubmitting your figure files are available below the reviewer comments at the end of this letter.

If applicable, we recommend that you deposit your laboratory protocols in protocols.io to enhance the reproducibility of your results. Protocols.io assigns your protocol its own identifier (DOI) so that it can be cited independently in the future. For instructions see: https://journals.plos.org/plosone/s/submission-guidelines#loc-laboratory-protocols. Additionally, PLOS ONE offers an option for publishing peer-reviewed Lab Protocol articles, which describe protocols hosted on protocols.io. Read more information on sharing protocols at https://plos.org/protocols?utm_medium=editorial-email&utm_source=authorletters&utm_campaign=protocols.

We look forward to receiving your revised manuscript.

Kind regards,

Mohamed Y.M. Hanfi

Academic Editor

PLOS One

Journal Requirements:

If the reviewer comments include a recommendation to cite specific previously published works, please review and evaluate these publications to determine whether they are relevant and should be cited. There is no requirement to cite these works unless the editor has indicated otherwise.

[Note: HTML markup is below. Please do not edit.]

Reviewers' comments:

Reviewer's Responses to Questions

**Comments to the Author**

1. If the authors have adequately addressed your comments raised in a previous round of review and you feel that this manuscript is now acceptable for publication, you may indicate that here to bypass the “Comments to the Author” section, enter your conflict of interest statement in the “Confidential to Editor” section, and submit your "Accept" recommendation.

Reviewer #2: All comments have been addressed

Reviewer #3: All comments have been addressed

Reviewer #4: All comments have been addressed

Reviewer #5: (No Response)

**********

2. Is the manuscript technically sound, and do the data support the conclusions?

The manuscript must describe a technically sound piece of scientific research with data that supports the conclusions. Experiments must have been conducted rigorously, with appropriate controls, replication, and sample sizes. The conclusions must be drawn appropriately based on the data presented.

Reviewer #2: Yes

Reviewer #3: Yes

Reviewer #4: Yes

Reviewer #5: Partly

**********

3. Has the statistical analysis been performed appropriately and rigorously?

Reviewer #2: Yes

Reviewer #3: Yes

Reviewer #4: Yes

Reviewer #5: No

**********

4. Have the authors made all data underlying the findings in their manuscript fully available?

The PLOS Data policy requires authors to make all data underlying the findings described in their manuscript fully available without restriction, with rare exception (please refer to the Data Availability Statement in the manuscript PDF file). The data should be provided as part of the manuscript or its supporting information, or deposited to a public repository. For example, in addition to summary statistics, the data points behind means, medians and variance measures should be available. If there are restrictions on publicly sharing data—e.g. participant privacy or use of data from a third party—those must be specified.

Reviewer #2: Yes

Reviewer #3: Yes

Reviewer #4: Yes

Reviewer #5: No

**********

5. Is the manuscript presented in an intelligible fashion and written in standard English?

PLOS ONE does not copyedit accepted manuscripts, so the language in submitted articles must be clear, correct, and unambiguous. Any typographical or grammatical errors should be corrected at revision, so please note any specific errors here.

Reviewer #2: Yes

Reviewer #3: Yes

Reviewer #4: Yes

Reviewer #5: No

**********

6. Review Comments to the Author

Please use the space provided to explain your answers to the questions above. You may also include additional comments for the author, including concerns about dual publication, research ethics, or publication ethics. (Please upload your review as an attachment if it exceeds 20,000 characters)

Reviewer #2: The authors have addressed the comments appropriately, and the manuscript is now suitable for publication.

Reviewer #3: Review of the manuscript titled “Control of deposition-epigenetic alteration of sandbody on uranium mineralization in Toutunhe Formation, Louzhuangzi area, Southern Margin of Junggar Basin, China”, submitted to the journal “Plos One”.

- Line 15: Remove “shows” and use has instead of have: “In recent years, some uranium mineralization with industrial values has been discovered in ….”

- Line 21: use “in the Toutunhe Formation (J2t) of the Louzhuangzi area …”, and you can remove “of the Louzhuangzi area” from line 27.

- Line 36: Turpan-Hami, Songliao Basins, and the northern…

- Line 41: mineralizations

- Lines 49-53: The sentence is very long.

- Line 54: …during the period from the Late Cretaceous to the Paleogene. The Middle and…

- Line 78: Use the full name for Si, P and Y.

- Line 79-82: The sentence is very long.

- Line 81: determines that the mineralization…

- Lines 86-94: this is a paragraph that should consist of at least 3 sentences, not one sentence. Avoid long sentences. Same in lines 110-116, divide the paragraph into sentences.

- Line 102: …mineralization (Fig 19). Is it Figure 19 or Reference 19?

- Line 124: the orientation “N-WW” seems incorrect.

- The quality of most of your Figures is bad. The content, essentially the texts, is unreadable (e.g., Figures 1, 2, 8, 9, 13, 14? 18). Please provide a high-quality version of your Figures.

- Line 150: use “stratigraphic column of Louzhuangzi area…” instead of “Composite stratigraphic column of Louzhuangzi area…”

- Methods and Materials: label the subheads “Fieldwork and sampling, Petrographic and mineralogical analysis…” as 3.1; 3.2; …

- Adding some field or lab pictures may be useful.

- Lines 191, 193: I ask if the Figures cited are Figures 7A, 7B?

- Lines 201: I ask if the Figure cited is Figure 12?

- Please revise the citation of your figures, and organize them following the order of their appearance in the text.

- Line 239: use micrometer symbol “μm” not “um” for 2600um, 500 and 1500um,…

- Lines 340-344: The sentence “The ore-bearing main… bar developed” is very long. Same for the next one. Same for the sentence in lines 609-614.

- Line 376: “document”? You want to say “shows”?

- It is recommended to discuss the main strengths and limitations of this work.

- Overall, the manuscript must be revised before sending it for the next review.

Reviewer #4: Reviewer #1:

Comments

The abstract lacks some key aspects, including

Quantify: Add numbers to findings (e.g., "〖"CO" 〗_2purity >95%").

Clarify: Define the specific "controversy" being addressed.

Comments

Keywords: The authors may consider changing some of the keywords using one of the suggestions below:

• Sandstone-type uranium deposits (Standardized classification)

• Braided river delta (Specific depositional micro-facies from your abstract)

• Hydrocarbon-mediated reduction (The specific "how" of your mineralization)

• FIB-TEM/SEM analysis (Specific high-end methodology)

• Toutunhe Formation (Necessary stratigraphic marker)

• Redox transition zone (The geochemical environment)

• Northwest China (Broader geographic context to reach a wider audience)

Comments

Are all the pictures used here original? If you take a random picture, you must have permission before using it.

Please state what role the funders took in the study. If the funders had no role, please state: "The funders had no role in study design, data collection and analysis, decision to publish, or preparation of the manuscript."

If this statement is not correct, you must amend it as needed. And include it in the cover letter, that will be enough.

Comments

Introduction: The text needs to be solidified by adding more references; each statement requires supporting references.

5. Comments

Methodology: If it's possible, the author can add a graphical abstract.

Reviewer #5: Comments on: Control of deposition–epigenetic alteration of sandstone units on uranium mineralization in the Toutunhe Formation, Louzhuangzi area, Southern Margin of the Junggar Basin, China

This manuscript addresses an important and enduring question in sandstone-type uranium geology: how depositional architecture interacts with later epigenetic processes to control uranium mineralization. The Louzhuangzi area is, in principle, a suitable case study, and the authors have assembled a substantial dataset that includes petrography, SEM-EDS, EPMA, and TEM observations. These efforts are commendable. However, in its current form, the manuscript is weakened by several significant technical limitations, interpretive overreach, and unresolved procedural concerns. As a result, the conclusions extend beyond what the available data can reliably support.

At the most basic level, the sedimentological framework is not sufficiently developed to underpin the interpretations that follow. The manuscript asserts a transition from a gravelly braided river system in the lower Toutunhe Formation to a meandering river system in the upper part, yet this distinction is largely stated rather than demonstrated. Although sedimentary structures such as cross-bedding and erosional contacts are described in detail, key architectural elements that distinguish braided from meandering systems—such as channel geometry, accretion styles, facies proportions, paleocurrent patterns, and systematic grain-size trends—are not presented. In the absence of paleocurrent data, fining-upward successions, or facies architecture diagrams, the depositional interpretations remain ambiguous.

This limitation becomes particularly important when uranium mineralization is later linked to specific “lag deposits” and depositional microfacies. While several microfacies are listed, they are neither mapped nor quantified, and it is unclear which facies host uranium preferentially, or how consistently this relationship holds across the study area. Consequently, the central claim that depositional controls govern uranium distribution lacks a clearly demonstrated sedimentological basis.

More serious concerns arise with the mineralogical characterization of uranium occurrence states. The manuscript distinguishes among coffinite, pitchblende, brannerite, and “dispersed adsorbed uranium,” yet these categories are not supported by sufficiently rigorous mineralogical criteria. Coffinite and pitchblende are differentiated primarily on the basis of SiO₂ content, despite substantial compositional overlap and the absence of X-ray diffraction data. Several analyses attributed to coffinite approach pitchblende compositions, rendering phase identification uncertain.

This uncertainty carries over into the classification of “dispersed adsorbed uranium,” which is defined as nanoscale uranium whose mineral type cannot be confirmed. If the mineralogical identity is unknown, it is difficult to justify excluding poorly crystalline or nanoscale coffinite and pitchblende, particularly when the high TiO₂ contents of these analyses suggest association with titanium oxides rather than true adsorption. Indeed, the manuscript later acknowledges that such uranium may occur as nanoscale minerals attached to Ti-oxide dissolution pores, which directly conflicts with its earlier treatment as a distinct occurrence state.

The identification of brannerite is presented as a key novel contribution, yet it is among the least convincing aspects of the study. Reported compositions deviate from ideal brannerite stoichiometry, and no crystallographic confirmation is provided. Without selected-area electron diffraction or clear lattice-fringe imaging demonstrating the UTi₂O₆ structure, alternative interpretations—such as intergrowths of uranium phases with titanium oxides—cannot be excluded. Given the prominence of this claim, its evidentiary basis needs to be significantly strengthened.

Interpretations of alteration zonation and redox evolution are similarly overstated. Figures showing the spatial relationships among oxidation fronts, depositional facies, and mineralization combine multiple data types without indicating data density or confidence levels. In several areas, particularly in the western part of the study area, interpretations appear to rely on sparse drilling, yet are presented as regionally robust. The interpretation of extensive interlayer oxidation is based largely on color changes and uranium anomalies, without supporting geochemical redox indicators, systematic documentation of alteration mineral assemblages, or mass-balance considerations.

As a result, the conclusion that interlayer oxidation controlled uranium mineralization becomes circular: oxidation is inferred from visual criteria associated with uranium enrichment, and the spatial coincidence is then taken as evidence of genetic control. Alternative scenarios, such as post-mineralization oxidation, are not explored in depth, and no temporal constraints are provided to distinguish cause from effect.

A similar pattern appears in the discussion of sulfurization and hydrocarbon-related reduction. The alteration of titanium oxides and the presence of pyrite are convincingly documented, and the regional context of hydrocarbon systems is appropriately noted. However, the inference that H₂S and CH₄ derived from deep oil and gas leakage were the primary reducing agents responsible for uranium precipitation is not directly supported by the data presented. No sulfur isotope analyses, organic geochemical data, or temporal relationships between hydrocarbon migration and uranium mineralization are provided. Instead, the argument relies on regional analogies and external studies, while acknowledging limitations in the current dataset. If hydrocarbon reduction is to be treated as a central genetic mechanism, it requires primary evidence within this study.

In a broader scientific context, the manuscript does not clearly articulate what insights from Louzhuangzi can be generalized to other sandstone-type uranium systems. Concepts such as multi-stage alteration, facies control on mineralization, and redox-driven uranium precipitation are well established. The proposed novelty lies in their specific combination and sequence at Louzhuangzi, yet these stages are neither independently dated nor constrained by geochemical or isotopic data. Thermal fluids are invoked without temperature or compositional constraints, hydrocarbons without diagnostic geochemical fingerprints, and basin-scale flow systems without hydrological evidence. Consequently, the proposed model reads more as a plausible narrative than a tested framework.

Several procedural and ethical issues further compound these scientific concerns. Changes in authors are not explained and should be transparently addressed in the cover letter and authorship declarations. I noticed that two additional authors have been added to the revised manuscript. In addition, reliance on unpublished or forthcoming institutional studies to support key interpretations is inappropriate when such evidence is central to the manuscript’s conclusions. Interpretations should stand on data that are accessible and peer-reviewed.

In summary, this manuscript contains valuable observations and has the potential to contribute meaningfully to the understanding of sandstone-type uranium mineralization. However, substantial revisions are required. Core mineralogical identifications must be validated using XRD and crystallographic TEM techniques; alteration models must be supported by geochemical and temporal constraints; sedimentological interpretations must be grounded in quantitative facies and architectural analyses; and speculative elements must be clearly separated from evidence-based conclusions. Until these issues are addressed, the manuscript’s central claims remain insufficiently supported, limiting its reliability and broader applicability. Below I point attention to some of these issues for authors’ and editor’s reference.

1. Severe Introduction Deficiencies

The introduction (lines 32-105) lacks:

• Clear knowledge gaps: What specifically is unknown about this deposit type?

• Theoretical framework: No explicit statement of competing hypotheses

• Literature synthesis: References are listed without critical evaluation of conflicting interpretations

The manuscript will benefit from a more robust introduction, if authors can address the concerns above.

2. Methods Section (lines 152-181)

Critical missing elements even after revision:

• Sample selection criteria and representativenes.

• Sampling Strategy Not Justified

• Table 1 lists samples from various depths (24.84m to 871.50m) and drill holes.

• No explanation of selection criteria

• No discussion of spatial representativeness

• Samples concentrated in specific holes (e.g., 6 samples from ZK6-2, only 1 from ZK3-3)

• Potential sampling bias need to be addressed

• Quality control procedures

• Detection limits for EMPA analyses

• Reproducibility assessment

EMPA Data Quality Issues (Table 2):

• Totals range from 84.01% to 97.14% - some analyses are poor quality

• No standard reference materials mentioned

• Single-point analyses without replication

• No discussion of analytical uncertainty

• Sub-optimal totals (e.g., 84.01% for brannerite) suggest volatile loss or inappropriate analytical conditions

Missing Critical Techniques: The authors acknowledge (only after review) absence of:

• XRD - fundamental for phase identification

• Fluid inclusions - critical for understanding ore-forming fluids

• Bulk geochemistry - necessary for provenance and alteration assessment

• Isotopic data - essential for fluid source and timing constraints

The Response to Reviewers states: "Due to the limited quantity of samples available for destructive analysis, XRD analysis was not conducted". This is scientifically unacceptable because:

• XRD requires minimal sample (~100mg)

• The study collected 22 samples (Table 1)

• XRD is non-negotiable for validating mineral identifications

• Claims about "chlorite/smectite mixed layer minerals" cannot be confirmed without XRD

3. Results-Discussion Conflation

Despite revision, interpretive statements remain embedded in Results:

• Lines 314-315: "indicating that the type of the sandstone is greywacke and the sandbodies were formed in a proximal deposit environment" - this is interpretation

• Lines 345-361: Depositional environment interpretation mixed with descriptive sedimentology

• Lines 520-528: Genetic interpretations presented as observations

Brannerite Identification - Overstated Certainty

The manuscript claims (lines 457-467) definitive brannerite identification, but:

• Evidence presented: Only EDS element mapping showing Ti and U co-distribution

• Missing: Electron diffraction patterns confirming brannerite crystal structure

• TEM images (Fig. 11B-C): Show "complexly interwoven acicular aggregates" but lack lattice fringe imaging

• Chemical composition (Table 2): TiO₂/UO₂ ratios (average 33.20%/39.62%) deviate from stoichiometric UTi₂O₆ (expected ~29.7%/63.3%)

Critical issue: The text contradicts itself - lines 467 states this is "nanoscale brannerite aggregate, rather than nanoscale coffinite or pitchblende associated with titanium oxides," but the evidence for this distinction is not compelling without crystallographic data.

4. INTERPRETIVE WEAKNESSES

i. "Thermal Fluids" - Unsupported Speculation

The manuscript repeatedly invokes "thermal fluids" (lines 29-30, 101, 731-744, 754) but:

Evidence provided:

• Clay mineral assemblages (chlorite, chlorite-smectite)

• Brannerite occurrence

• Sulfurization features

Problems:

• These features can form at diagenetic temperatures (<100°C)

• No temperature constraints whatsoever

• No fluid inclusion data

• No isotopic evidence for deep fluid sources

• The Discussion (lines 731-744) is essentially an admission that thermal fluids are speculative

The authors state (Response to Reviewers): "due to the lack of direct fluid inclusion or isotopic data, the properties and pathways of these thermal fluids cannot be definitively identified"

Then why make it a central interpretive element? This represents speculation presented as conclusion.

ii. Hydrocarbon Reduction - Circumstantial Evidence

The manuscript argues (lines 69-71, 641-643, 677-690) that H₂S and CH₄ from hydrocarbon leakage caused reduction, based on:

• Presence of pyrite and bitumen

• Regional petroleum geology

• Citation of He et al. (2024)

Missing:

• Direct evidence of hydrocarbon fluid inclusions

• Organic geochemistry linking bitumen to deep sources

• Sulfur isotope data to constrain H₂S sources (biological vs. thermochemical)

• Temporal constraints on hydrocarbon migration vs. uranium precipitation

Reviewer 1 noted: "Additional data, such as fluid inclusions with more analysis, could strengthen the hydrocarbon reduction hypothesis"

Authors' response: Acknowledge limitation and cite He et al. (2024) for supporting evidence

Problem: This effectively outsources key evidence to another study while maintaining the interpretation as a primary conclusion.

iii. Depositional Facies Analysis - Oversimplified

The depositional environment interpretation (Section 4.2, lines 305-396) suffers from:

• Sedimentary structures described but not systematically analyzed: No paleocurrent data, no grain size distribution analysis, no facies associations diagram

• Uncritical facies assignment: E.g., "flaser bedding... mainly formed by migration and deposition... in meandering river channel" (lines 317-320) - but flaser bedding forms in tidal settings and other environments

• Missing quantitative data: No channel dimensions, no sand/mud ratios, no architectural element analysis

• References misapplied: Folk (1968) cited for sedimentary environments (line 344) when it's a textural classification scheme

5. DATA PRESENTATION ISSUES

1. Table 2 - Serious Problems

Compositional Data:

• 35 analyses presented without context (which samples? which grains?)

• Mineral types assigned post-hoc based on composition

• No clear criteria for distinguishing "dispersed adsorbed uranium" (5 analyses) from coffinite (16 analyses)

• Some "coffinite" analyses have very low SiO₂ (9.33%) approaching pitchblende composition

Statistical treatment absent:

• No mean ± standard deviation

• No compositional ranges by deposit location

• No correlation analysis (e.g., UO₂ vs. TiO₂)

6. DISCUSSION SECTION - SUPERFICIAL SYNTHESIS

The Discussion (added/restructured after revision, lines 646-755) has improved structure but remains problematic:

Section 5.1 (Sedimentary control):

• States braided channel facies are primary hosts (correct)

• Comparison with Ordos, Songliao, Texas deposits mentioned but not developed

• Missing: Why is Toutunhe Formation mineralization similar/different from these analogues?

Section 5.2 (Compositional maturity):

• Q/(F+R) ratios discussed (lines 657-675)

• Links low maturity to uranium mobility - reasonable

• Missing: Comparison with unmineralized Toutunhe sandstones - are compositional differences significant?

Section 5.3 (Redox buffering):

• Argues pyrite and organics reduced U⁶⁺ to U⁴⁺

• Problem: This is standard ore-forming process - what's specific to Louzhuangzi?

• References [39-43] cited but not critically evaluated

Section 5.4 (Alteration timing):

• Distinguishes early diagenetic from epigenetic alteration

• Major gap: No geochronology, no cross-cutting relationships systematically documented

• Relies on textural interpretation alone

Section 5.5 (Future geochemical work):

• This is not discussion - it's an admission of missing data

• Acknowledges need for major/trace elements, REE, Ti/Zr, Th/U ratios

• Should have been done before publication

Section 5.6 (Thermal fluids origin):

• Essentially states "we don't know" (lines 731-744)

• Speculation about basin-centered flow, magmatic underplating

• No new insight beyond acknowledging uncertainty

Section 5.7 (Mineralization model):

• Figure 19 presents conceptual model

• Problem: Model incorporates unvalidated elements (thermal fluids, hydrocarbon timing)

• Three-stage process (oxidation → thermal overprint → hydrocarbon reduction) is plausible but not demonstrated

7. WRITING QUALITY AND PRESENTATION

Pervasive Issues:

i. Jargon and unclear terminology:

o "Deposition-epigenetic alteration" (title) - awkward phrasing

o "Gravel braided river channel lag deposits" (line 28) - "gravel braided river" is non-standard (should be "gravelly braided river")

o "Uranium-bearing sandbody" - "sandbody" is informal; use "sandstone unit"

ii. Grammatical errors (examples):

o Line 88: "the properties of the ore-forming fluids have been determined" - overstated; properties remain uncertain

o Line 314: "indicating that the type of the sandstone is greywacke" - awkward construction

o Line 474: "The dispersed adsorbed uranium is actually the nano-scale uranium minerals" - "is actually" undermines precision

iii. Redundancy:

o Extensive description of sedimentary structures (lines 196-204, 318-333, 344-361) could be condensed

o Repeated statements about gray/grayish-white alteration zones (lines 578-583, 632-635, 687-690)

**********

7. PLOS authors have the option to publish the peer review history of their article (what does this mean?). If published, this will include your full peer review and any attached files.

If you choose “no”, your identity will remain anonymous but your review may still be made public.

**Do you want your identity to be public for this peer review?**   For information about this choice, including consent withdrawal, please see our Privacy Policy.

Reviewer #2: **Yes:**   Hussien Yessuf Ahmed

Reviewer #3: **Yes:**   Abderrahim Ayad

Reviewer #4: No

Reviewer #5: No

**********

[NOTE: If reviewer comments were submitted as an attachment file, they will be attached to this email and accessible via the submission site. Please log into your account, locate the manuscript record, and check for the action link "View Attachments". If this link does not appear, there are no attachment files.]

To ensure your figures meet our technical requirements, please review our figure guidelines: https://journals.plos.org/plosone/s/figures

You may also use PLOS’s free figure tool, NAAS, to help you prepare publication quality figures: https://journals.plos.org/plosone/s/figures#loc-tools-for-figure-preparation.

NAAS will assess whether your figures meet our technical requirements by comparing each figure against our figure specifications.

# Author comment: Author response to Decision Letter 2

**DOI:** 10.1371/journal.pone.0351337.r004

13 Mar 2026

Dear Reviewers, Thank you very much for your constructive suggestions on our submitted manuscript. Your feedback has been invaluable in improving its quality. In the resubmitted manuscript, we have made the corresponding revisions based on your suggestions, which are explained in the Cover Letter. Detailed revisions are included in the Revised Manuscript with Track Changes. Specific responses to the revisions are provided in the Response to Reviewers.

Attachment Submitted filename: Response_to_Reviewers_auresp_2.docx

# Peer review: Decision Letter 2

**DOI:** 10.1371/journal.pone.0351337.r005

**Authors:** Mohamed Y.M. Hanfi

10 Apr 2026

PONE-D-25-34881R2

Sedimentary architecture and post-depositional alteration of the Toutunhe Formation (J2t) in the Louzhuangzi area, Southern Junggar Basin: Implications for uranium mineralization

PLOS One

Dear Dr. Wang,

Thank you for submitting your manuscript to PLOS ONE. After careful consideration, we feel that it has merit but does not fully meet PLOS ONE’s publication criteria as it currently stands. Therefore, we invite you to submit a revised version of the manuscript that addresses the points raised during the review process.

Please submit your revised manuscript by May 25 2026 11:59PM. If you will need more time than this to complete your revisions, please reply to this message or contact the journal office at plosone@plos.org. When you're ready to submit your revision, log on to https://www.editorialmanager.com/pone/ and select the 'Submissions Needing Revision' folder to locate your manuscript file.

Please include the following items when submitting your revised manuscript:

- A letter that responds to each point raised by the academic editor and reviewer(s). You should upload this letter as a separate file labeled 'Response to Reviewers'.
- A marked-up copy of your manuscript that highlights changes made to the original version. You should upload this as a separate file labeled 'Revised Manuscript with Track Changes'.
- An unmarked version of your revised paper without tracked changes. You should upload this as a separate file labeled 'Manuscript'.

If you would like to make changes to your financial disclosure, please include your updated statement in your cover letter. Guidelines for resubmitting your figure files are available below the reviewer comments at the end of this letter.

If applicable, we recommend that you deposit your laboratory protocols in protocols.io to enhance the reproducibility of your results. Protocols.io assigns your protocol its own identifier (DOI) so that it can be cited independently in the future. For instructions see: https://journals.plos.org/plosone/s/submission-guidelines#loc-laboratory-protocols. Additionally, PLOS ONE offers an option for publishing peer-reviewed Lab Protocol articles, which describe protocols hosted on protocols.io. Read more information on sharing protocols at https://plos.org/protocols?utm_medium=editorial-email&utm_source=authorletters&utm_campaign=protocols.

As the corresponding author, your ORCID iD is verified in the submission system and will appear in the published article. PLOS supports the use of ORCID, and we encourage all coauthors to register for an ORCID iD and use it as well. Please encourage your coauthors to verify their ORCID iD within the submission system before final acceptance, as unverified ORCID iDs will not appear in the published article. *Only*   the individual author can complete the verification step; PLOS staff *cannot*   verify ORCID iDs on behalf of authors.

We look forward to receiving your revised manuscript.

Kind regards,

Mohamed Y.M. Hanfi

Academic Editor

PLOS One

Journal Requirements:

If the reviewer comments include a recommendation to cite specific previously published works, please review and evaluate these publications to determine whether they are relevant and should be cited. There is no requirement to cite these works unless the editor has indicated otherwise.

[Note: HTML markup is below. Please do not edit.]

Reviewers' comments:

Reviewer's Responses to Questions

**Comments to the Author**

1. If the authors have adequately addressed your comments raised in a previous round of review and you feel that this manuscript is now acceptable for publication, you may indicate that here to bypass the “Comments to the Author” section, enter your conflict of interest statement in the “Confidential to Editor” section, and submit your "Accept" recommendation.

Reviewer #2: All comments have been addressed

Reviewer #4: All comments have been addressed

Reviewer #5: (No Response)

**********

2. Is the manuscript technically sound, and do the data support the conclusions?

The manuscript must describe a technically sound piece of scientific research with data that supports the conclusions. Experiments must have been conducted rigorously, with appropriate controls, replication, and sample sizes. The conclusions must be drawn appropriately based on the data presented.

Reviewer #2: Yes

Reviewer #4: Yes

Reviewer #5: Partly

**********

3. Has the statistical analysis been performed appropriately and rigorously?

Reviewer #2: Yes

Reviewer #4: Yes

Reviewer #5: No

**********

4. Have the authors made all data underlying the findings in their manuscript fully available?

The PLOS Data policy requires authors to make all data underlying the findings described in their manuscript fully available without restriction, with rare exception (please refer to the Data Availability Statement in the manuscript PDF file). The data should be provided as part of the manuscript or its supporting information, or deposited to a public repository. For example, in addition to summary statistics, the data points behind means, medians and variance measures should be available. If there are restrictions on publicly sharing data—e.g. participant privacy or use of data from a third party—those must be specified.

Reviewer #2: Yes

Reviewer #4: Yes

Reviewer #5: Yes

**********

5. Is the manuscript presented in an intelligible fashion and written in standard English?

PLOS ONE does not copyedit accepted manuscripts, so the language in submitted articles must be clear, correct, and unambiguous. Any typographical or grammatical errors should be corrected at revision, so please note any specific errors here.

Reviewer #2: Yes

Reviewer #4: (No Response)

Reviewer #5: No

**********

6. Review Comments to the Author

Please use the space provided to explain your answers to the questions above. You may also include additional comments for the author, including concerns about dual publication, research ethics, or publication ethics. (Please upload your review as an attachment if it exceeds 20,000 characters)

Reviewer #2: Review Comments to the Author

General Comments

I have carefully evaluated this manuscript according to the PLOS ONE publication criteria, which emphasize scientific rigor and methodological soundness. Overall, the study is technically solid, and the data support the conclusions. The manuscript would benefit from minor revisions, primarily regarding clarity, consistency, and presentation.

Specific Comments

1. Abstract

• In the Abstract:

o The objective of the study should be stated more clearly in the first 2–3 sentences.

o Some results are described qualitatively; consider adding key quantitative values (e.g., mineral percentages, geochemical ranges).

o The final sentence should better emphasize the main conclusion or implication.

2. Introduction

• The Introduction provides sufficient background, but:

o The research gap is not clearly highlighted. Please explicitly state what is missing in previous studies.

o The objective(s) of the study should be clearly listed at the end of the section.

3. Geological Setting

• The section is informative, but:

o Some lithological descriptions are repetitive- these could be streamlined.

• Ensure consistency in terminology (e.g., formation names, stratigraphic units).

4. Materials and Methods

• This section is generally well prepared and meets PLOS ONE standards.

• However:

o Please specify sample selection criteria more clearly (why these samples were chosen).

o Analytical conditions (e.g., SEM, EPMA settings) are described, but consider grouping them in a more structured format for readability.

o If applicable, include uncertainty/error margins for measurements.

5. Results

• The results are comprehensive and well presented.

• Suggestions:

o Separate observations from interpretations more clearly (some interpretations appear within the Results section).

o Figures and tables:

• Enhance figures, tables, and their captions

o Consider summarizing key findings in a short subsection or table.

6. Discussion

• The Discussion is scientifically sound and well-reasoned.

• Improvements:

o Some arguments could be strengthened by comparing results with similar studies.

o Avoid repetition of results; focus more on interpretation and implications.

o The genetic model is interesting, but it would benefit from:

• A clearer step-by-step explanation

• Possibly a conceptual diagram

7. Conclusions

• The Conclusions are appropriate but could be improved:

o Avoid repeating detailed results.

o Focus on key findings and their implications.

o Consider adding 1–2 sentences on future research directions.

8. Language and Presentation

• The manuscript is understandable, but several minor issues should be addressed:

o Grammatical inconsistencies (e.g., verb tense, article usage)

o Occasional repetition of phrases

� A professional language edit is recommended.

• Overall Recommendation: Minor Revision

Reviewer #4: (No Response)

Reviewer #5: This revised manuscript is much readable. The topic is relevant and potentially valuable, particularly given the combination of field observations and micro-analytical techniques (SEM, EPMA, FIB-TEM). However, despite the promising dataset, the manuscript requires substantial revision. The principal shortcomings relate to lack of methodological clarity, weak sedimentological rigor, insufficient linkage between data and interpretation, and structural inconsistencies throughout the manuscript. In its current form, the study does not yet meet the standards required for publication.

I have some comments on the annotated PDF manuscript, which are amplified here. Authors are advised to consult both materials when revising their manuscript.

**********

7. PLOS authors have the option to publish the peer review history of their article (what does this mean?). If published, this will include your full peer review and any attached files.

If you choose “no”, your identity will remain anonymous but your review may still be made public.

**Do you want your identity to be public for this peer review?**   For information about this choice, including consent withdrawal, please see our Privacy Policy.

Reviewer #2: **Yes:**   Hussien Yessuf Ahmed

Reviewer #4: No

Reviewer #5: **Yes:**   Ekundayo Adepehin

**********

[NOTE: If reviewer comments were submitted as an attachment file, they will be attached to this email and accessible via the submission site. Please log into your account, locate the manuscript record, and check for the action link "View Attachments". If this link does not appear, there are no attachment files.]

To ensure your figures meet our technical requirements, please review our figure guidelines: https://journals.plos.org/plosone/s/figures

You may also use PLOS’s free figure tool, NAAS, to help you prepare publication quality figures: https://journals.plos.org/plosone/s/figures#loc-tools-for-figure-preparation.

NAAS will assess whether your figures meet our technical requirements by comparing each figure against our figure specifications.

Attachment Submitted filename: PONE-D-25-34881_R2_reviewer_EJA.pdf

Attachment Submitted filename: Reviewer Comment_Adepehin.pdf

# Author comment: Author response to Decision Letter 3

**DOI:** 10.1371/journal.pone.0351337.r006

10 May 2026

Editor-in-Chief,

PLOS ONE,

Dear Editor,

Thank you for your constructive comments that were very helpful in improving the quality of this manuscript. We have carefully reviewed the comments and made corresponding revisions to the manuscript. We have submitted the second revised manuscript to PLOS ONE, entitled " Control of deposition-epigenetic alteration of sandbody on uranium mineralization in Toutunhe Formation, Louzhuangzi area, Southern Margin of Junggar Basin, China ".

We have comprehensively revised the paper based on the reviewers' comments. The main revisions are marked in red in the manuscript. The most important revisions are as follows:

Reviewer #2:

General Comments

I have carefully evaluated this manuscript according to the PLOS ONE publication criteria, which emphasize scientific rigor and methodological soundness. Overall, the study is technically solid, and the data support the conclusions. The manuscript would benefit from minor revisions, primarily regarding clarity, consistency, and presentation.

Response: Thank you for your constructive suggestions. We are grateful for your positive assessment that the study is technically sound and that the data adequately support the conclusions. In response to your suggestions, we have carefully revised the manuscript to improve clarity, consistency, and overall presentation. Specifically, we have refined the language for greater precision and readability, ensured consistency in terminology throughout the text, and improved the organization and formatting of figures and descriptions. We believe these revisions have strengthened the manuscript and enhanced its overall quality. We sincerely appreciate your helpful comments, which have contributed to improving our work.

Specific Comments.

1. Abstract

In the Abstract:

The objective of the study should be stated more clearly in the first 2–3 sentences.

Some results are described qualitatively; consider adding key quantitative values (e.g., mineral percentages, geochemical ranges).

The final sentence should better emphasize the main conclusion or implication.

Response: Thank you for your constructive suggestions. In response, we have thoroughly revised the Abstract to improve its clarity, structure, and scientific rigor. Specifically, (1) the research objective has been clearly stated within the first two sentences to better define the purpose of the study; (2) key quantitative results, including mineral proportions (e.g., coffinite and pitchblende ~68%, Ti-bearing uranium phases ~16%, and nanoscale uranium minerals ~16%) and geochemical ranges (e.g., UO₂ contents), have been incorporated to replace previously qualitative descriptions; and (3) the final sentence has been rewritten to more explicitly highlight the main conclusion and its broader geological implications. These revisions ensure that the Abstract now follows a clear structure (background–objective–methods–results–implications) and better aligns with the journal’s expectations. We appreciate your suggestions, which have significantly improved the quality of this section.

2. Introduction

The Introduction provides sufficient background, but:

The research gap is not clearly highlighted. Please explicitly state what is missing in previous studies.

The objective(s) of the study should be clearly listed at the end of the section.

Response: Thank you for your constructive suggestions. We have carefully revised the introduction to clearly identify research gaps and articulate the objectives of this study.

First, we have added a dedicated paragraph that systematically outlines the limitations of previous research, thus reinforcing the understanding of these gaps.

Specifically, we now emphasize:(1) The microscopic features of sedimentary structures and post-depositional alteration remain insufficiently revealed; (2) The nature and interactions of ore-forming fluids (oxidizing and reducing fluids) remain controversial; and (3) Previous studies have lacked integration of mineralogical evidence at the micrometer to nanometer scale. These additions clearly highlight the shortcomings of previous research and underscore the necessity of this study.

Second, we have clearly articulated the objectives of this study in a structured manner at the end of the introduction. We have added a new paragraph outlining four specific objectives: (i) Analysis of sedimentary tectonic features; (ii) Identification of mineralogical features using multi-scale analysis methods (SEM, electron probe microscopy, focused ion beam-transmission electron microscopy); (iii) Constraining the evolution of ore-forming fluids; and (iv) Constructing a sedimentary-fluid coupled genetic model.

3. Geological Setting

The section is informative, but:

Some lithological descriptions are repetitive- these could be streamlined.

Ensure consistency in terminology (e.g., formation names, stratigraphic units).

Response: Thank you for your constructive suggestions. We have carefully revised the Geological Setting section to improve clarity, conciseness, and consistency. Specifically, repetitive lithological descriptions have been streamlined and consolidated to avoid redundancy while preserving essential geological information. In addition, we have standardized the terminology throughout the section, ensuring consistent use of formation names and stratigraphic units.

4. Materials and Methods

This section is generally well prepared and meets PLOS ONE standards.

However:

Please specify sample selection criteria more clearly (why these samples were chosen).

Analytical conditions (e.g., SEM, EPMA settings) are described, but consider grouping them in a more structured format for readability.

If applicable, include uncertainty/error margins for measurements.

Response: Thank you for your constructive suggestions. We have revised the Materials and Methods section accordingly:

Sample selection criteria: These have now been clearly defined, including the focus on mineralized grayish-white altered sandstones, selection of fresh and unweathered core intervals, and coverage of both vertical and lateral variability to ensure representativeness.

Analytical conditions: The analytical methods have been reorganized into structured subsections (e.g., SEM, EPMA, TEM), with instrument settings and analytical purposes systematically reported to improve readability.

Analytical uncertainties: Relevant information on analytical conditions, calibration standards, and data quality (e.g., oxide total ranges for EPMA) has been added to better constrain measurement uncertainties.

5. Results

The results are comprehensive and well presented.

Suggestions:

Separate observations from interpretations more clearly (some interpretations appear within the Results section).

Figures and tables: Enhance figures, tables, and their captions

Consider summarizing key findings in a short subsection or table.

Response: Thank you for your constructive suggestions. We have revised the manuscript accordingly: Separation of observations and interpretations: The Results section has been carefully revised to retain only observational data, while interpretative content has been moved to the Discussion section where appropriate. Figures and tables: All figures and tables have been improved for clarity and readability, and their captions have been expanded to provide more complete and self-explanatory descriptions. Summary of key findings: A concise summary of the main results has been added to highlight the key findings and improve the overall readability of the Results section.

6. Discussion

The Discussion is scientifically sound and well-reasoned.

Improvements:

Some arguments could be strengthened by comparing results with similar studies.

Avoid repetition of results; focus more on interpretation and implications.

The genetic model is interesting, but it would benefit from:

A clearer step-by-step explanation

Possibly a conceptual diagram

Response: Thank you for your constructive suggestions. We have revised the Discussion section accordingly: Comparison with previous studies: Additional comparisons with relevant studies on sandstone-hosted uranium deposits and related systems have been incorporated to strengthen the arguments; Reduction of repetition: Repetitive descriptions of results have been removed, with greater emphasis placed on interpretation and geological implications; Genetic model improvement: The genetic model has been clarified with a more explicit step-by-step explanation, and a conceptual diagram has been added to better illustrate the proposed processes.

7. Conclusions

The Conclusions are appropriate but could be improved:

Avoid repeating detailed results.

Focus on key findings and their implications.

Consider adding 1–2 sentences on future research directions.

Response: Thank you for your constructive suggestions. We have revised the Conclusions section accordingly:Reduced repetition of detailed results: The Conclusions have been streamlined to avoid reiterating descriptive results, with greater emphasis placed on synthesizing key findings; Strengthened implications: We now highlight the broader geological significance, including sedimentary control, multi-fluid interactions, and nanoscale mineralization processes in sandstone-hosted uranium systems; Added future research directions: One to two sentences have been included to outline future work, particularly focusing on fluid evolution, quantitative constraints, and the formation mechanisms of Ti–U nanophases.

8. Language and Presentation

The manuscript is understandable, but several minor issues should be addressed:

Grammatical inconsistencies (e.g., verb tense, article usage)

Occasional repetition of phrases

A professional language edit is recommended.

Response: Thank you for your constructive suggestions. We have carefully revised the manuscript to improve language quality and presentation: Grammatical issues: Verb tense, article usage, and sentence structure have been systematically corrected throughout the manuscript; Repetition: Redundant phrases and repetitive expressions have been removed or refined to enhance clarity and conciseness; Language editing: The manuscript has undergone thorough language polishing to ensure clarity, consistency, and readability.

Reviewer #5:

General Evaluation

This revised manuscript is much readable. The topic is relevant and potentially valuable, particularly given the combination of field observations and micro-analytical techniques (SEM, EPMA, FIB-TEM). However, despite the promising dataset, the manuscript requires substantial revision. The principal shortcomings relate to lack of methodological clarity, weak sedimentological rigor, insufficient linkage between data and interpretation, and structural inconsistencies throughout the manuscript. In its current form, the study does not yet meet the standards required for publication. I have some comments on the annotated PDF manuscript, which are amplified here. Authors are advised to consult both materials when revising their manuscript.

Response: Thank you for your constructive suggestions. We appreciate your recognition that the manuscript has improved in readability and that the dataset, integrating field observations with micro-analytical techniques (SEM, EPMA, and FIB–TEM), is valuable.

We also acknowledge your concerns regarding methodological clarity, sedimentological rigor, the linkage between data and interpretation, and structural consistency. In response, we have undertaken a comprehensive revision of the manuscript. Specifically, we have: (1) substantially clarified the Methods section by providing detailed descriptions of analytical procedures, instrumentation, standards, and uncertainties; (2) strengthened the sedimentological analysis by refining facies interpretations and improving the description of depositional architecture; (3) enhanced the integration between observational data and genetic interpretations to ensure that all conclusions are directly supported by the presented evidence; and (4) reorganized the manuscript to improve overall structure, logical flow, and consistency across sections.

In addition, we have carefully addressed all comments provided in the annotated PDF, and corresponding revisions have been incorporated throughout the manuscript. We believe these improvements have significantly strengthened the scientific rigor and clarity of the study. We sincerely thank you for your detailed and valuable feedback, which has greatly contributed to enhancing the quality of our work

Major Comments

1. Abstract and Statement of Purpose

The abstract is overly long, not well structured and does not clearly articulate the research objective.

Lines 18–22: The abstract emphasizes methodology prematurely rather than clearly stating the aim of the study. As noted in the manuscript, “this sentence needs re-framing… the latter part should establish the purpose of the study rather than highlighting methodology.”

The abstract length should also be verified against journal requirements.

Recommendation: Reorganize the abstract to follow a clear structure: background → objective → methods → key findings → implications.

Response: Thank you for your constructive suggestions. In response, we have comprehensively revised the Abstract to improve its structure, clarity, and alignment with journal requirements. Specifically, (1) the Abstract has been reorganized to follow a clear and logical structure of background → objective → methods → key findings → implications; (2) the research objective is now explicitly stated in the opening sentences to clearly articulate the purpose of the study; (3) the previously noted sentences (Lines 18–22) have been rephrased to avoid prematurely emphasizing methodology, with the focus shifted toward establishing the study aim; and (4) the overall length of the Abstract has been reduced to comply with the journal’s word limit. These revisions ensure that the Abstract more effectively communicates the scientific objectives, key results, and broader implications of the study. We appreciate your constructive suggestions, which have significantly improved the presentation and readability of this section.

2. Inadequate Literature Context

The introduction lacks sufficient engagement with key literature.

Line 66: The manuscript discusses uranium mineralization controls but does not cite landmark studies (“Please name some landmark papers relating to this here”).

Lines 74–75 and 88–90: Statements are made without citations (“source??”).

Recommendation: Strengthen the literature review by incorporating foundational and recent studies on:

Sandstone-hosted uranium systems

Sedimentary controls on mineralization

Regional geology of the Junggar Basin

Response: Thank you for your constructive suggestions. We have thoroughly revised the Introduction to strengthen the literature context by incorporating both foundational and recent studies, and by adding appropriate citations to all previously unsupported statements.

3. Geological Background – Conceptual and Factual Issues

The geological background section is problematic.

Lines 150–154: The tectonic description is misleading and oversimplified, as highlighted. How can the Jungar Basin be located on Kazakhstan?

Line 154: Timing of tectonic evolution is unclear

Line 161: Citation required but missing.

Recommendation: Reconstruct this section using established regional geological frameworks with proper chronological and tectonic context, supported by appropriate references.

Response: Thank you for your constructive suggestions. We acknowledge that the previous version contained oversimplified and potentially misleading descriptions of the regional tectonic framework.

(1) Tectonic framework clarification (Lines 150–154):

We have revised the description to avoid ambiguity regarding the tectonic position of the Junggar Basin. The updated text now clearly states that the basin is located within the eastern segment of the Kazakhstan Plate and is bounded by the Siberian Plate to the north and the Tarim Plate to the south, rather than being “located on Kazakhstan.” This revision aligns with widely accepted regional tectonic frameworks (e.g., Cheng , 2001; Windley et al., 2007;

Attachment Submitted filename: Response_to_Reviewers_auresp_3.docx

# Peer review: Decision Letter 3

**DOI:** 10.1371/journal.pone.0351337.r007

**Authors:** Mohamed Y.M. Hanfi

26 May 2026

Depositional architecture and post-depositional alteration of the Toutunhe Formation (J2t) in the Louzhuangzi area, Southern Junggar Basin: Implications for uranium mineralization

PONE-D-25-34881R3

Dear Dr. Qing Wang,

We’re pleased to inform you that your manuscript has been judged scientifically suitable for publication and will be formally accepted for publication once it meets all outstanding technical requirements.

Within one week, you’ll receive an e-mail detailing the required amendments. When these have been addressed, you’ll receive a formal acceptance letter and your manuscript will be scheduled for publication.

An invoice will be generated when your article is formally accepted. Please note, if your institution has a publishing partnership with PLOS and your article meets the relevant criteria, all or part of your publication costs will be covered. Please make sure your user information is up-to-date by logging into Editorial Manager at Editorial Manager® and clicking the ‘Update My Information' link at the top of the page. For questions related to billing, please contact billing support.

If your institution or institutions have a press office, please notify them about your upcoming paper to help maximize its impact. If they’ll be preparing press materials, please inform our press team as soon as possible -- no later than 48 hours after receiving the formal acceptance. Your manuscript will remain under strict press embargo until 2 pm Eastern Time on the date of publication. For more information, please contact onepress@plos.org.

Kind regards,

Mohamed Y.M. Hanfi

Academic Editor

PLOS One

Additional Editor Comments (optional):

Reviewers' comments:

Reviewer's Responses to Questions

**Comments to the Author**

1. If the authors have adequately addressed your comments raised in a previous round of review and you feel that this manuscript is now acceptable for publication, you may indicate that here to bypass the “Comments to the Author” section, enter your conflict of interest statement in the “Confidential to Editor” section, and submit your "Accept" recommendation.

Reviewer #2: All comments have been addressed

Reviewer #5: All comments have been addressed

**********

2. Is the manuscript technically sound, and do the data support the conclusions?

The manuscript must describe a technically sound piece of scientific research with data that supports the conclusions. Experiments must have been conducted rigorously, with appropriate controls, replication, and sample sizes. The conclusions must be drawn appropriately based on the data presented.

Reviewer #2: Yes

Reviewer #5: Yes

**********

3. Has the statistical analysis been performed appropriately and rigorously?

Reviewer #2: Yes

Reviewer #5: N/A

**********

4. Have the authors made all data underlying the findings in their manuscript fully available?

The PLOS Data policy requires authors to make all data underlying the findings described in their manuscript fully available without restriction, with rare exception (please refer to the Data Availability Statement in the manuscript PDF file). The data should be provided as part of the manuscript or its supporting information, or deposited to a public repository. For example, in addition to summary statistics, the data points behind means, medians and variance measures should be available. If there are restrictions on publicly sharing data—e.g. participant privacy or use of data from a third party—those must be specified.

Reviewer #2: Yes

Reviewer #5: (No Response)

**********

5. Is the manuscript presented in an intelligible fashion and written in standard English?

PLOS ONE does not copyedit accepted manuscripts, so the language in submitted articles must be clear, correct, and unambiguous. Any typographical or grammatical errors should be corrected at revision, so please note any specific errors here.

Reviewer #2: Yes

Reviewer #5: Yes

**********

6. Review Comments to the Author

Please use the space provided to explain your answers to the questions above. You may also include additional comments for the author, including concerns about dual publication, research ethics, or publication ethics. (Please upload your review as an attachment if it exceeds 20,000 characters)

Reviewer #2: The revised manuscript shows clear and substantial improvement, with better organization, clearer methods, and stronger scientific coherence. Key sections such as the Abstract, Introduction, Geological Background, and Methods are now more clearly written and better structured. The addition of quantitative data, improved sampling description, and integrated analytical techniques (petrography, SEM, EPMA, and FIB–TEM) significantly strengthens the study and enhances its contribution to understanding sandstone-hosted uranium mineralization in the southern Junggar Basin.

However, several important issues still require further attention before the manuscript can be considered fully suitable for publication:

1. The sedimentological interpretation has improved, but the overall sedimentological rigor remains moderate. Several depositional facies interpretations would benefit from clearer diagnostic criteria and stronger support from sedimentary structures, grain-size characteristics, and architectural relationships. A more process-based facies analysis would strengthen this section considerably.

2. Some interpretative statements are still included within the Results section. Separating observational data more clearly from interpretation/discussion would improve both the scientific clarity and the structure of the manuscript.

3. Although the Discussion section is improved, some interpretations are still not sufficiently supported by the presented petrographic, mineralogical, and sedimentological data. The discussion would benefit from stronger and more explicit connections between the analytical results and the proposed geological interpretations.

4. The proposed genetic model is interesting and has improved significantly; however, the relationships among sediment deposition, diagenesis, oxidation, hydrothermal alteration, fluid evolution, and uranium precipitation are still not fully constrained.

5. The integration between micro-scale mineralogical observations and basin-scale geological processes remains somewhat inferential. Additional discussion linking permeability architecture, fluid migration pathways, redox evolution, alteration processes, and uranium mineralization would improve the robustness of the proposed model.

6. The language quality has improved considerably, but the manuscript still contains occasional grammatical inconsistencies, awkward sentence construction, repetitive wording, and several overly long sentences. A final professional English language edit is recommended before acceptance.

Overall, with additional revisions focused on strengthening the evidence-based discussion, improving sedimentological interpretation, clarifying the linkage between results and interpretations/discussion, and refining the language, the manuscript could become suitable for publication in PLOS ONE.

Reviewer #5: (No Response)

**********

7. PLOS authors have the option to publish the peer review history of their article (what does this mean?). If published, this will include your full peer review and any attached files.

If you choose “no”, your identity will remain anonymous but your review may still be made public.

**Do you want your identity to be public for this peer review?**   For information about this choice, including consent withdrawal, please see our Privacy Policy.

Reviewer #2: **Yes:**   hussien yessuf Ahmed

Reviewer #5: **Yes:**   Ekundayo Joseph Adepehin

**********

Attachment Submitted filename: Commets to the Author.docx

# Editor report: Acceptance letter

**DOI:** 10.1371/journal.pone.0351337.r008

**Authors:** Mohamed Y.M. Hanfi

PONE-D-25-34881R3

PLOS One

Dear Dr. Wang,

I'm pleased to inform you that your manuscript has been deemed suitable for publication in PLOS One. Congratulations! Your manuscript is now being handed over to our production team.

At this stage, our production department will prepare your paper for publication. This includes ensuring the following:

* All references, tables, and figures are properly cited

* All relevant supporting information is included in the manuscript submission,

* There are no issues that prevent the paper from being properly typeset

You will receive further instructions from the production team, including instructions on how to review your proof when it is ready. Please keep in mind that we are working through a large volume of accepted articles, so please give us a few days to review your paper and let you know the next and final steps.

Lastly, if your institution or institutions have a press office, please let them know about your upcoming paper now to help maximize its impact. If they'll be preparing press materials, please inform our press team within the next 48 hours. Your manuscript will remain under strict press embargo until 2 pm Eastern Time on the date of publication. For more information, please contact onepress@plos.org.

You will receive an invoice from PLOS for your publication fee after your manuscript has reached the completed accept phase. If you receive an email requesting payment before acceptance or for any other service, this may be a phishing scheme. Learn how to identify phishing emails and protect your accounts at https://explore.plos.org/phishing.

If we can help with anything else, please email us at customercare@plos.org.

Thank you for submitting your work to PLOS ONE and supporting open access.

Kind regards,

PLOS ONE Editorial Office Staff

on behalf of

Dr. Mohamed Y.M. Hanfi

Academic Editor

PLOS One
